# Growth and survival relationships of 71 tree species with nitrogen and sulfur deposition across the conterminous U.S.

**DOI:** 10.1371/journal.pone.0205296

**Published:** 2018-10-18

**Authors:** Kevin J. Horn, R. Quinn Thomas, Christopher M. Clark, Linda H. Pardo, Mark E. Fenn, Gregory B. Lawrence, Steven S. Perakis, Erica A. H. Smithwick, Douglas Baldwin, Sabine Braun, Annika Nordin, Charles H. Perry, Jennifer N. Phelan, Paul G. Schaberg, Samuel B. St. Clair, Richard Warby, Shaun Watmough

**Affiliations:** 1 Department of Forest Resources and Environmental Conservation, Virginia Tech, Cheatham Hall, Blacksburg, VA, United States of America; 2 United States Environmental Protection Agency, National Center for Environmental Assessment, Washington, DC, United States of America; 3 United States Department of Agriculture Forest Service, Northern Research Station, Burlington, VT, United States of America; 4 United States Department of Agriculture Forest Service, Pacific Southwest Research Station, Riverside, CA, United States of America; 5 New York Water Science Center, U.S. Geological Survey, Troy, NY, United States of America; 6 Forest and Rangeland Ecosystem Science Center, US Geological Survey, Corvallis, OR, United States of America; 7 Department of Geography, The Pennsylvania State University, University Park, PA, United States of America; 8 Institute for Applied Plant Biology, Witterswil, Switzerland; 9 Umeå Plant Science Centre, Swedish University of Agricultural Sciences, Umeå, Sweden; 10 United States Department of Agriculture Forest Service, Northern Research Station, St. Paul, MN, United States of America; 11 Research Triangle Institute (RTI) International, Research Triangle Park, NC, United States of America; 12 Department of Plant and Wildlife Sciences, Brigham Young University, Provo, UT, United States of America; 13 The Warby Group LLC, North Attleboro, MA, United States of America; 14 Trent School of the Environment, Trent University, Peterborough, ON, Canada; INRA, FRANCE

## Abstract

Atmospheric deposition of nitrogen (N) influences forest demographics and carbon (C) uptake through multiple mechanisms that vary among tree species. Prior studies have estimated the effects of atmospheric N deposition on temperate forests by leveraging forest inventory measurements across regional gradients in deposition. However, in the United States (U.S.), these previous studies were limited in the number of species and the spatial scale of analysis, and did not include sulfur (S) deposition as a potential covariate. Here, we present a comprehensive analysis of how tree growth and survival for 71 species vary with N and S deposition across the conterminous U.S. Our analysis of 1,423,455 trees from forest plots inventoried between 2000 and 2016 reveals that the growth and/or survival of the vast majority of species in the analysis (n = 66, or 93%) were significantly affected by atmospheric deposition. Species co-occurred across the conterminous U.S. that had decreasing and increasing relationships between growth (or survival) and N deposition, with just over half of species responding negatively in either growth or survival to increased N deposition somewhere in their range (42 out of 71). Averaged across species and conterminous U.S., however, we found that an increase in deposition above current rates of N deposition would coincide with a small net increase in tree growth (1.7% per Δ kg N ha^-1^ yr^-1^), and a small net decrease in tree survival (-0.22% per Δ kg N ha^-1^ yr^-1^), with substantial regional and among-species variation. Adding S as a predictor improved the overall model performance for 70% of the species in the analysis. Our findings have potential to help inform ecosystem management and air pollution policy across the conterminous U.S., and suggest that N and S deposition have likely altered forest demographics in the U.S.

## Introduction

Elevated atmospheric nitrogen (N) deposition influences the growth and survival of trees and the terrestrial carbon (C) sink through complex and simultaneous mechanisms, such as reducing nutrient limitation, acidifying soils, and altering competitive interactions [1–3]. Unresolved complexity in geographic, biotic, and other factors have led to non-uniform responses of individual tree species to N deposition, as seen in both gradient studies [4–8] and ecosystem-scale N addition experiments [2, 9–11]. Evaluating how tree species differ in their response to N deposition is critical for assessing the resilience of forests to chronic N deposition and the resulting impacts on forest composition [3, 12].

Species-specific empirical relationships between atmospheric N deposition and tree demographic rates are being used to guide the development of critical loads (numerical thresholds above which negative ecological effects begin to occur according to present knowledge [13]) in the United States (U.S.). However, prior work developing these relationships has been limited in geographic range and in the number of tree species assessed. Such empirical relationships are based on a space-for-time substitution in which the patterns in growth and survival of species are analyzed across atmospheric deposition gradients. In a key study, Thomas et al. [6] leveraged the N deposition gradient across the Northeastern U.S. to develop growth and survival relationships of different tree species with N deposition, after accounting for climate and tree size. Others have performed similar gradient-based analyses at the plant functional type (rather than species) scale [14], for smaller regions in the conterminous U.S. [15], or in Europe [5]. However, there are two important limitations to the empirical approach presented by Thomas et al. [6] that prevent its use in developing critical loads for forests of the conterminous U.S. First, that study’s scope included only the effects of N deposition on the 24 most common species in the Northeastern U.S. Second, since N deposition can covary with sulfur (S) deposition at the landscape- to regional-scale, the relationships reported in Thomas et al. [6] did not control for the effects of S and may be implicitly biased by forest sensitivity to S deposition.

The effects of atmospheric S deposition on forest ecosystems are generally negative as a result of soil acidification, increased aluminum mobility in soils, cation leaching, and subsequent foliar nutrient perturbations [16, 17]. Atmospheric S deposition can eventually lead to mortality and reduced tree regeneration, ultimately impacting tree growth [18, 19]. However, tree species differ widely in sensitivity to S deposition [20, 21], potentially based on differences in physiology, local soil conditions, or other factors. Even though S deposition over much of the U.S. has significantly decreased in the past few decades [16], it remains elevated over pre-industrial levels [22]. Consequently, it is critical to disentangle the separate effects of N and S deposition on trees, if critical loads are developed for both N and S deposition.

**Fig 1:**
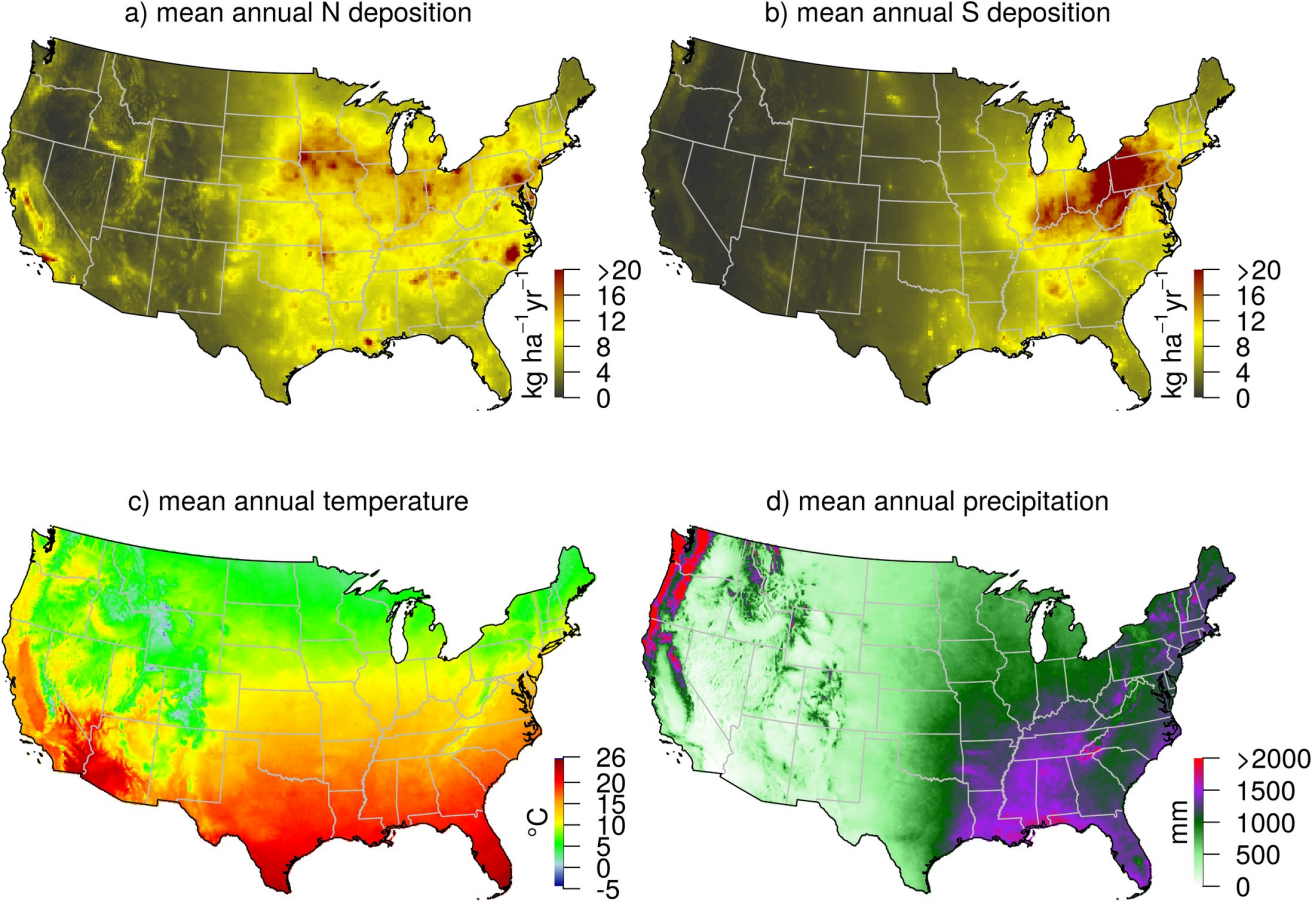
Gradients of N deposition, S deposition, mean annual temperature, and mean annual precipitation across the conterminous U.S. Panels are the a) mean total N deposition from 2000–2012, b) mean total S deposition from 2000–2012, c) mean annual temperature from 2000–2014, and d) mean annual precipitation form 2000–2014. Deposition data are from the TDEP product [23] and climate data are from PRISM (http://prism.oregonstate.edu). The averages for each tree in the analysis were a subset of these datasets that coincided with its measurement interval. The values used in the species-level empirical models correspond to the spatial distribution of the species.

Here we build on Thomas et al. [6] by expanding the analysis of growth and survival relationships with N deposition to the conterminous U.S., characterizing the responses of a large number of temperate tree species (71 of the 94 most common species that pass a covariate collinearity threshold), and including both climate and S deposition as covariates. Specifically, we used remeasured tree inventory data from the extensive U.S. Forest Service’s Forest Inventory and Analysis (FIA) program for which growth and survival measurements for some states have only recently become available. We linked these growth and survival rates with spatially explicit estimates of atmospheric N and S deposition (Fig 1A and 1B) [23] and with historical climate data [24] (Fig 1C and 1D) to evaluate whether atmospheric deposition improves empirical models that describe growth and survival for each tree species as a function of climate, tree size, and competitive environment. Our primary objective was to understand how N deposition (and its covariates) affects forest growth and survival through the contribution of individual tree species. A secondary objective was to understand tree responses to S deposition, both to adequately account for a stressor that can be correlated with N deposition, and to explore differential sensitivities among species to this important stressor. Finally, unique to this analysis, we place the results in the context of covariation between N deposition, S deposition, and climate, thus identifying where the strength of evidence from gradient analysis is strongest.

## Methods

### Forest inventory data

Tree growth, survival, and basal area data were compiled from the FIA program database (accessed on January 24, 2017). The FIA program routinely collects and calculates multiple tree and site values including tree diameter, height, and survival status for all trees that are at least 12.7 cm in diameter (FIA phase 2 manual version 6.1; http://www.fia.fs.fed.us/). Tree biomass was estimated from tree diameter measurements [25] and then multiplied by 0.5 to estimate aboveground C (FIA phase 2 manual version 6.1; http://www.fia.fs.fed.us/). Tree growth rates were calculated from the difference in aboveground C between the latest and first live measurement available of every tree and divided by the elapsed time between measurements to the day. For growth analysis, we used only the first and last live measurement of each tree inventoried using the national design plot (KINDCD = 1, 2, or 3; approximately years 2000–2016 varying by state and inventory cycle with the most common remeasurement interval at approximately 10 years) to limit the pseudo-replication of trees and minimize measurement error by calculating the largest difference in aboveground biomass for each tree. The growth analysis included trees that were on private, state, and federal lands and without regard to disturbance history. In an effort to reduce the amount of noise in the data, trees with growth rates beyond the 95% quantile were excluded. We removed all trees with negative growth values from the data for the growth analysis only as reductions in diameter are highly improbable after 10 years. Tree species that had at least 2,000 individuals in both growth and survival datasets after the data filters were applied were retained for growth and survival analyses (see S1 Table). As a result of the filters, 94 tree species were included in the analysis

The probability of tree survival was calculated from the first live measurement to the last live measurement or to the first measurement recorded as dead for each tree inventoried. Trees that were recorded as dead at both measurement inventories and trees that were harvested were excluded from the survival analysis.

### Climate and atmospheric deposition data

To obtain N and S deposition rates for each tree, we used spatially modeled N and S deposition data from the U.S. National Atmospheric Deposition Program's Total Deposition Science Committee (Fig 1A and 1B, TDEP) [23]. Gridded deposition data were obtained from the U.S. Environmental Protection Agency's FTP server for years 2000 to 2013 (tdep; ftp://ftp.epa.gov/castnet/tdep/grids/ and ftp://ftp.epa.gov/castnet/tdep/images/). Total N and S deposition grids were bilinearly sampled with the publicly available FIA plot locations in R (v3.2.2, Vienna, Austria) and were of sufficiently coarse spatial resolution (4 x 4 km pixels) to not require the actual FIA plot locations [26]. Annual deposition rates were then averaged from the first year of measurement to the last year of measurement for every tree so that each tree had an individualized average N and S deposition that reflects the location and remeasurement period of the tree. Consequently, since our analysis was at the species-level, each species had a unique range of N and S deposition that is determined by the species distribution (i.e., some species have individual trees in regions of higher deposition). Monthly mean temperature and precipitation values were obtained in a gridded (4 x 4 km) format from the PRISM Climate Group at Oregon State University [24] for the conterminous U.S. and sampled in a similar manner to the atmospheric deposition data, resulting in individualized average temperature and annual precipitation values that correspond to the interval between measurement years (Fig 1C and 1D).

### Estimating individual tree growth by species

Our growth model, following Thomas et al. [6], assumes that there is a maximum potential growth rate (that is a function of tree size) modified by competition and climate, whose terms have values between 0 and 1. We then used model selection (described below) to determine whether models with N and/or S deposition terms added explanatory power beyond tree size, climate, and competition. This approach accounts for known drivers of growth and survival (size, climate, and competition) before testing for additional influences of N and S deposition. If the model with N and/or S deposition was selected over the model without the deposition terms, we used the parameterized shape of deposition terms to quantify the relationship between deposition and growth. This approach was equivalent to the methods used by Thomas et al. [6] except that we included two additional covariates (competition based on stand basal area and S deposition) as well as alternative size function terms from Canham and Murphy [7].

Specifically, to predict growth, we first considered each tree species to have an optimal growth rate that was a power function of its size [27] (Eq 1).

$$potential(size)=a\times {size}^{z}$$ (1)

where *size* was in units of aboveground C (kg C/individual), a is a fitted parameter, and z is a fitted parameter. Competition between trees was modeled as a function of plot basal area (*BA*) and the basal area of trees larger than that of the tree of interest (*BAL*) similar to the methods of Pukkala et al. [28] (Eq 2),

$$competition\;term=e^{\left[a_{2}BAL+a_{3}ln(BA)\right]}$$ (2)

where a_2_ and a_3_ are fitted parameters. Environmental conditions of mean annual temperature, mean annual precipitation, and mean annual N and S deposition attenuate these optimal growth rates using Eq 3. Each climate or deposition term was modeled using a modified lognormal function (Eq 3),

$$environment\;term=e^{-0.5{\left(\frac{ln[\frac{X}{x_{1}}]}{x_{2}}\right)}^{2}}$$ (3)

where *X* is the environmental variable and *x*_*1*_ and *x*_*2*_ are the fitted parameters for that variable; *t*_*1*_ and *t*_*2*_ are used for the temperature term, *p*_*1*_ and *p*_*2*_ are used for the precipitation term *n*_*1*_ and *n*_*2*_ are used for the N deposition term, and *s*_*1*_ and *s*_*2*_ are used for the S deposition term. Growth rates (*G*) were then calculated as the product of the size, competition, and environmental terms

$$\begin{array}{l} G=\\ potential(size)\times competition\;term\times temperature\;term\times precipitation\;term\times \\ nitrogen\;deposition\;term\times sulfur\;deposition\;term \end{array}$$ (4)

We examined a total of five different growth models: 1) a full model with the size, competition, climate, S deposition, and N deposition terms; 2) a model with all terms except the N deposition term; 3) a model with all terms except the S deposition term; 4) a model with all terms but without S and N deposition terms; and 5) a null model that estimated a single parameter for the mean growth parameter (a in Eq 5).

G=a (5)

See S2 Table for a full list of growth models.

### Estimating individual tree survival

Similar to the growth analysis, the annual probability of survival (*P(s)*) was estimated from the mathematical product of a maximum survival for the species (*a*), and functions for tree size, competition, climate, and deposition, all raised to the exponent of the elapsed time between the measurements in units of years (*time*) (Eq 6).

$$\begin{array}{l} P(s)=[a\times size\times competition\times temperature\;term\times precipitation\;term\times \\ \;nitrogen\;deposition\;term\times sulphur\;deposition\;term{]}^{time} \end{array}$$ (6)

The maximum survival rate (*a*) is an estimated parameter while the other terms are functions described below. We explored different functional forms with alternative representations of the influence of tree size and competition on survival. In the first set of survival models, size and competition variables were modeled after the methods of Canham and Murphy [7] (Eqs 7 and 8). Size was modeled with an exponential function:

$$size\;term=\left(1-zc_{1}e^{-zc_{2}size}\right)\times e^{-zc_{3}size^{zc_{4}}}$$ (7)

where *zc*_*1-4*_ are fitted parameters and *size* is the aboveground tree C in kg C/individual. Competition was modeled as a function of plot basal area (*BA*) and the ratio of the basal area of the tree of interest to the mean basal area of the plot (*BA*_*ratio*_; Eq 8)

$$competition=e^{-br_{1}\left(BA_{ratio}{}^{br_{2}}\right)\times \left(BA^{br_{3}}\right)}$$ (8)

where *br*_*1-3*_ are fitted parameters. Climate and deposition variables were modeled in the same modified lognormal form given in Eq 3.

The second set of survival models modeled size in the modified lognormal form (Eq 3) and competition in a combined modified lognormal form (Eq 9),

$$competition\_size\_combined=e^{-0.5{\left(\frac{\ln\left[\frac{BA}{ba_{1}}\right]}{ba_{2}}\right)}^{2}-0.5{\left(\frac{\ln\left(\frac{\left(BAL+1\right)}{\left(bl_{1}+1\right)}\right)}{bl_{2}}\right)}^{2}}$$ (9)

where ba_1_, ba_2_, bl_1_, and bl_2_ are fitted parameters. Similar to the growth models, described above, we examined a set of four models that all included climate, competition, size terms but differed in the inclusion of an N and/or S deposition term. We evaluated alternative size and competition terms (Eqs 8 and 9), thus doubling this set of models. Finally, we also fit a null survival model in which a mean annual estimate of survival (*a*) was raised to the exponent of the elapsed time (Eq 10).

$$P(s)=a^{time}$$ (10)

In total we fit nine survival models. See S2 Table for a full list of survival models.

### Fitting parameters and model selection

To estimate the parameters for each of the growth and survival models, we used simulated annealing (100,000 iterations) in the *likelihood* package (v2.1.1) in R to maximize the likelihood functions described below. For the S deposition relationship, we assumed that S deposition would not increase growth or survival, thus we restricted the parameters s_1_ to be below the minimum S deposition for each species. This was necessary to prevent too much flexibility in models with both N and S deposition and is consistent with common role S plays in soil acidification [29, 30]. Following Thomas et al. [6], error estimates for the growth models were normally distributed but with the standard deviation (*σ*) related to the predicted growth of the individual (*G*) (Eqs 11 and 12):

ln(likelikhood)∼ln(Normal(G,σ)) (11)

$$\sigma =\alpha \times G^{\beta }$$ (12)

where *α* and *β* are parameters estimated by the simulated annealing algorithm.

The log likelihood function for survival was a probabilistic model, following Thomas et al. [6] (Eq 13):

$$\ln\left(likelihood\right)=\sum_{i=1}^{n}\left[\begin{array}{cc} \ln\left(P{\left(s\right)}_{i}\right) & if\;the\;individual\;survived\\ \ln\left(1-P{\left(s\right)}_{i}\right) & if\;the\;individual\;died \end{array}\right]$$ (13)

where *P(s)*_*i*_ is the predicted probability of survival of the *i*^*th*^ individual.

We selected the best growth and survival model for each species using AIC [31]. Following Thomas et al. [6], we selected the final model for a species as the simplest model that had an AIC that was within 2.0 AIC units of the best overall model (i.e., the one with the lowest AIC). Since models that were within 2.0 AIC units of the best model were statistically indistinguishable but differing in which terms were included, we opted to use the rule of parsimony to select the best overall model with the fewest parameters in order to maintain consistency with the approach of Thomas et al. [6]. However, we recognize that the best overall model as measured by the lowest AIC is also a common and defensible choice [31]. As a result, we only considered growth or survival to have a relationship with atmospheric deposition (N and/or S) if the model that included the atmospheric deposition covariate had an AIC that was greater than 2.0 units lower that the model with only climate, competition, and tree size covariates. The full list of models, AIC values, and parameters are in S4 Table.

### Estimating tree-level growth and survival rates of change at current N and S deposition rates

To evaluate how the tree species relationships with N and S deposition combined with species distributions and spatial patterns of deposition across the conterminous U.S., we calculated the slope of the growth and survival relationships with N and S deposition at the current level of deposition experienced by each tree in the FIA census. The slope represents the change in growth or survival in response to a small change in N or S deposition from current rates. The slope of aboveground C growth rate (*∂G/∂N*) and survival (*∂P(s)/∂N*) were calculated from the first partial derivatives of the most parsimonious model with respect to N deposition (Eqs 14 and 15),

$$\frac{\partial G}{\partial N}=G\;\left(\frac{-\ln\left(N\right)-\ln\left(n_{1}\right)}{N{\left(n_{2}\right)}^{2}}\right)$$ (14)

$$\frac{\partial P\left(s\right)}{\partial N}=P\left(s\right)\;\left(\frac{-\ln\left(N\right)-\ln\left(n_{1}\right)}{N{\left(n_{2}\right)}^{2}}\right)$$ (15)

and likewise for S deposition (Eqs 16 and 17). (Note: parameter values are different between growth and survival models; e.g. n_1_ and n_2_ in Eq 14 and Eq 15)

$$\frac{\partial G}{\partial S}=G\;\left(\frac{-\ln\left(S\right)-\ln\left(s_{1}\right)}{S{\left(s_{2}\right)}^{2}}\right)$$ (16)

$$\frac{\partial P\left(s\right)}{\partial S}=P\left(s\right)\;\left(\frac{-\ln\left(S\right)-\ln\left(s_{1}\right)}{S{\left(s_{2}\right)}^{2}}\right)$$ (17)

Observed values were then used for the other variables (i.e. size, precipitation, N deposition, etc.) in the derivatives to determine the slope associated with N or S deposition for every tree in the filtered data. The slope of survival rate with N (*∂P(s)/∂N*) or S (*∂P(s)/∂S*) deposition was estimated for 10-year survival rates as this was the median remeasurement interval for all trees. Trees for which the species relationships with N or S deposition were not significant were assigned partial derivatives of 0. The slope for each tree was converted to a percent change by dividing the slope by the modeled growth or survival rate for the tree. For visualization purposes, these tree level slopes were then averaged over a 20 x 20 km raster grid.

### Quantifying collinearity in covariates

To quantify the amount of collinearity of N and S deposition against the other environmental variables of climate and deposition, we calculated variance inflation factors (VIF) of N and S deposition against all other environmental variables (S1 Table). This was done separately for each tree species and for both growth and survival datasets. To calculate the VIF, we ran an ordinary least square regression in R where the spatial pattern in N was regressed against the spatial pattern of mean annual temperature (*T*), mean annual precipitation (*P*), and S deposition (Eq 18).

$$N=\beta_{0}+\beta_{t}T+\beta_{p}P+\beta_{s}S$$ (18)

And where S was regressed against the spatial pattern of mean annual temperature (*T*), mean annual precipitation (*P*), and N deposition (Eq 19).

$$S=\beta_{0}+\beta_{t}T+\beta_{p}P+\beta_{s}N$$ (19)

We calculated the VIF of N deposition and S deposition for both growth and survival data separately for each tree species with Eq 20 in which *R*^*2*^ is the coefficient of determination from linear regression models (Eqs 18 and 19).

$$VIF=\frac{1}{1-R^{2}}$$ (20)

VIF values of 3–10 have been presented in the literature and statistic textbooks as a threshold for high collinearity [32, 33]. We analyzed all 94 species regardless of the VIF or correlation between N and S (S1 Table), but report in the main text species with VIF ≤ 3 for only four responses (growth and survival responses to N and S deposition). The presence of a high correlation or VIF does not mean the assumed causality is erroneous, it simply means that in our dataset we cannot assume independence of stressors.

## Results and discussion

### Species-level relationships with N and S deposition

Our analysis examined the relationship between N deposition and tree growth and survival for 1,423,455 trees from 94 species representing 36 different genera across the conterminous U.S. Of the 94 species analyzed, 71 species had VIF values for growth and survival responses to N and S deposition less or equal to 3. While we focus on these 71 to limit the collinearity among covariates in our analysis, results from all 94 species can be found in S1 Table.

**Fig 2:**
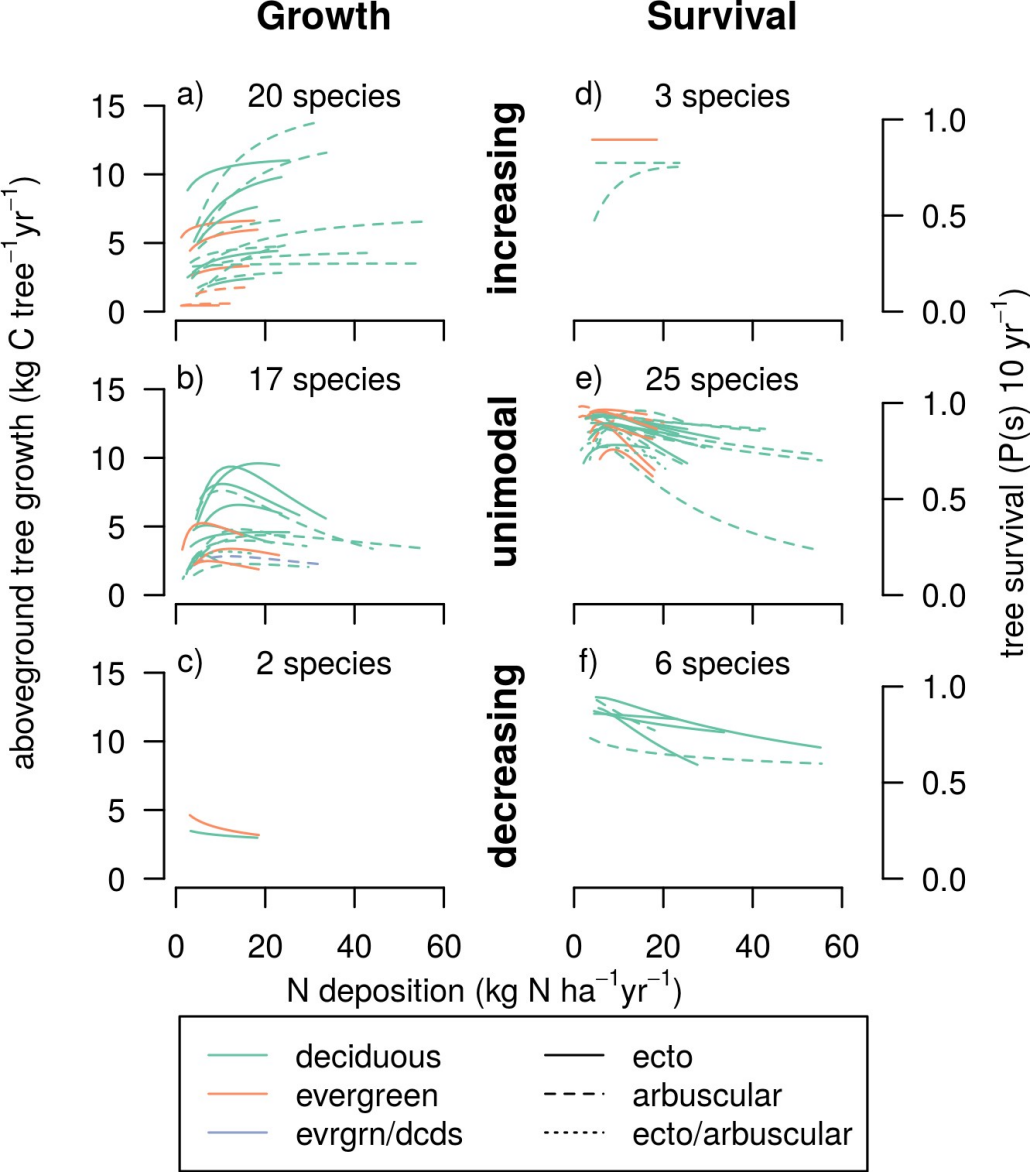
Tree growth and survival versus N deposition by species and colored by ecological attributes. Each curve represents the average tree growth (a-c) or survival (d-f) across the N deposition gradient for a single species. The relationships are separated by general curve shape; The (a,d) increasing, (b,e) unimodal, and (c,f) decreasing relationships with N deposition are shown. (For individual species and growth and survival curves by species and the curves associated with S deposition see S1 Fig). Note that two of the five species in panel d are functionally flat (< 0.01% change over deposition range) and are classified as flat in Table 1.

Of the 71 species with VIF ≤ 3, 39 species exhibited significant relationships between their growth and N deposition (with significant defined as having N deposition in the most parsimonious model with the lowest AIC), while the growth of 32 species did not vary with N deposition (Table 1; Fig 2). Of the 39 species with significant relationships, 20 species exhibited growth rates that increased across the full range of N deposition experienced by that species; the magnitude of the growth increase over the range of N deposition went from a 4% (*Pinus monophylla*) to a 77% change (*Robinia pseudoacaia*) (S1 Table). The group of species with increasing relationships with N deposition included 5 of the 9 nationally common species (defined by > = 2% of sampled trees): *Acer rubrum*, *Liriodendron tulipifera*, *Liquidambar styraciflua*, *Pseudotsuga menziesii*, and *Quercus rubra*.

**Table 1:** Summary of tree species’ relationships with N deposition. Numbers in columns and rows indicate the number of species with a monotonically decreasing, flat, monotonically increasing, or unimodal growth or survival association with N deposition across the range of deposition exposure. The letters denote classes based on the shapes of the combined growth and survival relationships, as described in the text.

	Survival relationship					
		Increase	Unimodal	Decrease	Flat	Sum
Growth relationship	Increase	1 (A)	8 (B)	1 (C)	10 (A)	20
	Unimodal	0 (B)	4 (D)	3 (E)	10 (D)	17
	Decrease	0 (C)	1 (E)	0 (F)	1 (F)	2
	Flat	0 (A)	12 (D)	2 (F)	18 (G)	32
	Sum	1	25	6	39	

Seventeen species displayed humped (or unimodal) relationships between growth and N deposition (Table 1; Fig 2). These are species in which growth was positively related to N deposition at lower levels of deposition but negatively related to N deposition at higher levels of N deposition. The N deposition level at which tree growth rates peaked ranged among species from 6 to 21 kg N ha^-1^ yr^-1^ (S1 Table; Fig 2). Nearly a quarter of species (4 of 17) with unimodal relationships exhibited peak growth rates at N deposition levels less than 10 kg N ha^-1^ yr^-1^ (S1 Table). The only nationally common species with a unimodal relationship was *Populus tremuloides* (peak at 11 kg N ha^-1^ yr^-1^).

Two species exhibited growth rates that decreased across the full range of N deposition experienced by the species (Table 1; Fig 2), the magnitude of change was 14% for *Betula alleghaniensis* and 31% for *Tsuga canadensis* (S1 Table). Neither of the species with consistent declines in growth due to N deposition were common at the national level. However, these two species are regionally important. It is possible that these species could have a positive response to N deposition associated with a very low level of N deposition, but we are unable to estimate where the peak would occur since that value would necessarily be below the current lowest observed value of N deposition.

Thirty-two tree species had significant relationships between survival and N deposition, while the survival of 39 species did not vary with N deposition. Of the species with relationships with N deposition, a unimodal response, where the 10-year survival rate increased to a peak before declining, was the most common (25 species). (Table 1; Fig 2). The N deposition rate at which survival rates peaked ranged from 2 to 14 kg N ha^-1^ yr^-1^, with all but two species displaying peaks at less than 10 kg N ha^-1^ yr^-1^ (S1 Table; Fig 2). Therefore, given current levels of N deposition, many of the tree species with humped shaped relationships may be on the declining side of their survival relationships. The common species with unimodal relationships were *Acer rubrum* (peak at 5.5 kg N ha^-1^ yr^-1^), *Liquidambar styraciflua* (peak at 5.2 kg N ha^-1^ yr^-1^), *Populus tremuloides* (peak at 4.2 kg N ha^-1^ yr^-1^), *Pseudotsuga menziesii* (peak at 5.2 kg N ha^-1^ yr^-1^), *Quercus alba* (peak at 6.8 kg N ha^-1^ yr^-1^), and *Quercus rubra* (peak at 5.6 kg N ha^-1^ yr^-1^).

Six species had consistently declining relationships between N deposition and survival (Table 1; Fig 2). The magnitude of decline for these species ranged from a relative reduction in 10-year survival rate of 3% (*Quercus coccinea*) to 33% (*Quercus falcata*) over the full range of N deposition experienced by that species (S1 Table). As with growth, none of the species with monotonically negative survival-N deposition relationships represented more than 2% of trees sampled, though many are regionally important. All of the species with declining survival as N deposition increased were hardwood species from the *Carya*, *Quercus*, and *Ulmus* genera, and were ectomycorrhizal symbionts—supporting previous studies that found arbuscular mycorrhizal symbionts fared better than ectomycorrhizal symbionts under elevated N deposition [6, 34, 35].

Only one species had increasing relationships between survival and N deposition (Table 1; Fig 2); the magnitude of change was a 37% (*Robinia pseudoacacia)*. Two additional species had relationship with N deposition that were statistically increasing (i.e., N deposition was in the best model and parameters results in an increase in survival across the deposition range) but the relationships with N deposition was numerically flat (less than 0.01% change in survival over the range of N deposition; *Pinus rigida* and *Sassafras albidum*).

The 71 tree species can be broadly classified into seven classes or “response syndromes” based on the shapes of their combined growth and survival relationships (superscripts in Table 1). The most common class (26 species) are species with a net positive effect at low deposition but a net negative effect at high deposition (class D in Table 1). These are species with unimodal relationship in both growth and survival or a unimodal relationship in one and a flat relationship in the other. The second most common class are species with no relationships with N deposition across the entire gradient (18 species, class G in Table 1). The third most common class are species with net positive relationships (11 species). These are species that exhibit an increasing relationship with N deposition in one response (growth or survival) and do not display a negative response in the other response. This class includes one species with positive relationships with N deposition for both growth and survival (*Robinia pseudoacaica*).

The less common classes include thirteen species that had contrasting relationships that depend on the rate of N deposition. There are three ways a species could have offsetting responses: (1) a monotonic increase in one response and a monotonic decrease in the other (1 species; class C in Table 1), whereby the net result depends on the relative magnitude of the growth response compared to the survival response; (2) a unimodal relationship with one response and a monotonic increase in the other, whereby the growth and survival increase with increasing N deposition at low rates but diverge in direction at higher rates of deposition (8 species; class B in Table 1); and (3) a unimodal relationship with one response and a monotonic decrease in the other, whereby growth and survival have opposite relationships at low deposition but both have decreasing relationships at high deposition rates (4 species; class E in Table 1),.

Finally, the less common classes also include species (3) that had clear net negative relationships with N deposition with decreasing relationship in one response (growth or survival) and do not have a positive relationship in the other response (i.e., have flat or decreasing relationships in the other response)(class F in Table 1). Importantly, no species exhibited both decreasing growth and decreasing survival in response to N deposition. Therefore, this class is exclusively species that have the combination of decreasing and flat relationships with N deposition.

While our study’s focus was primarily on the relationship between N deposition and tree growth and survival rates, we also examined the individual effects of S deposition on tree growth and survival. Of the 71 species with VIF ≤ 3, 31 exhibited decreasing growth with S deposition while the growth of the other 40 species did not vary with S deposition. Similarly, the survival of 40 species decreased with S deposition while the survival of 31 species did not vary with S deposition (S1 Table). No species exhibited increased growth or survival with S deposition because the structure of the models allowed only flat or negative relationships—an assumption that is consistent with the common role S plays in soil acidification and its uncommon role as a limiting micronutrient and is necessary to prevent too much flexibility in models with both N and S deposition [29, 30]. Only five species (*Carya cordiformis*, *Carya ovata*, *Celtis laevigata*, *Platanus occidentalis*, and *Quercus phellos*) showed no relationship with N or S deposition in either their growth or survival.

Overall, we found a wide variety of species-level growth and survival responses to N and S deposition. This suggests that the simple expectation of N deposition as beneficial at low levels and harmful at higher levels [1], though accurate for 38 of the 71 species, is not supported across all species within the range of deposition observed across the conterminous U.S. The growth and/or survival of some species declined at even very low levels of N (i.e., the species that decreased with N deposition), while others did not exhibit a threshold at which negative effects occurred (i.e., the species that increased with N deposition), or were unaffected by increased availability of this often limiting nutrient. It is unclear whether monotonic declines in some species were due to indirect negative effects via competition with neighbors that benefited more from N deposition, direct negative effects from soil acidification from increased N, or some combinations of these mechanisms. Species that were not responsive to increasing inputs of N may be already receiving sufficient N from soil processes, and/or immobilization rates in the soil may be high; in either case, additional N from deposition may not have a discernable effect. Similarly, the simplifying assumption that S deposition is harmful to all species also is not consistent with our results, suggesting that some species may be tolerant to this potential stressor. Thus, sensitivity to N and/or S deposition appears highly species- and/or context-specific, requiring further study.

### Scaling-up and mapping the rates of change with N and S deposition

**Fig 3:**
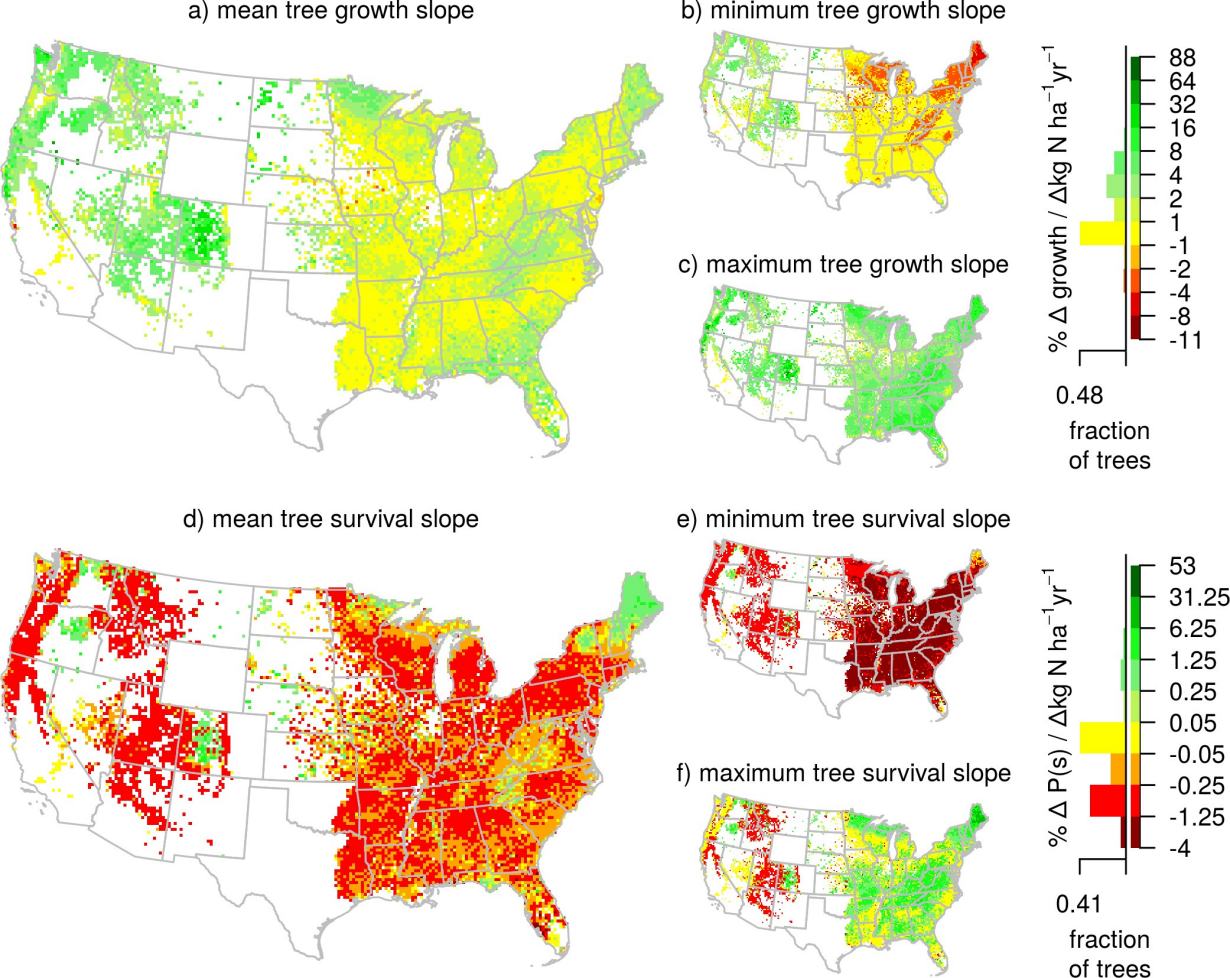
Relative change in growth and survival with N deposition of individual trees across the conterminous U.S. Maps show the relative mean, minimum, and maximum growth (a-c) and survival (d-f) responsiveness (percent change in growth or survival per change in annual N deposition rate) of trees within each 20 x 20 km pixel. Values were derived from the instantaneous rate of change in growth or survival with N deposition of the most parsimonious, species-specific models that allowed both N and S deposition (but did not necessarily contain N or S deposition). Red areas indicate where small increases in N deposition are associated with relatively large decreases in growth or survival while green areas indicate where small increases in N deposition are associated with relatively large increases in growth or survival. White areas are where no tree growth or survival data was available. Histograms indicate the fraction of individual trees by responsiveness to N deposition. Sample size for growth was 1,183,931 trees and for survival was 1,423,455 trees.

Averaged across inventoried trees in conterminous U.S., the slope of the relationship between tree growth and N deposition, at current rates of N deposition, was positive (1.7% per Δ kg N ha^-1^ yr^-1^; Fig 3). This positive rate of change reflects our finding above that a positive relationship with N deposition is the most common non-flat relationship at the species level. However, there is considerable variation; the 95% quantile interval for growth from all trees in the conterminous U.S. ranges from -2.0 to 8.1% per Δ kg N ha^-1^ yr^-1^ (Fig 3B and 3C). Spatially, the slope of the growth relationship was highest in the Pacific Northwest, central Appalachian Mountains, parts of the central Rocky Mountains, and the Upper Midwest (Fig 3). No region has a negative averaged slope throughout (i.e., only a few grid-cells are negative across the entire conterminous U.S.), though much of the conterminous U.S. has an average slope near zero. However, nearly all locations in the conterminous U.S., particularly in the eastern U.S., include trees with positive and negative slopes to N deposition at current rates of N deposition (minimum and maximum rates in Fig 3), highlighting that species with increasing and decreasing relationships co-occur with one another across the country.

In contrast, the slope of the relationship between ten-year tree survival and N deposition was negative, -0.22% per Δ kg N ha^-1^ yr^-1^ (Fig 3), with individual trees ranging from a -1.8 to 1.0% per Δ kg N ha^-1^ yr^-1^ across conterminous U.S. The decrease in survival rates reflects that most species with non-flat relationships had either decreasing relationships with N deposition or unimodal relationships with peaks in survival at relatively low values of N deposition. Regionally, survival slopes vary from an increase in the New England states, parts of the Pacific Northwest, and central Rocky Mountains to a decrease throughout most of the Eastern U.S (Fig 3). Similar to the slopes of the growth relationship, species with positive and negative slopes are co-located, indicating that changes in N deposition could be having an influence on tree biodiversity.

**Fig 4:**
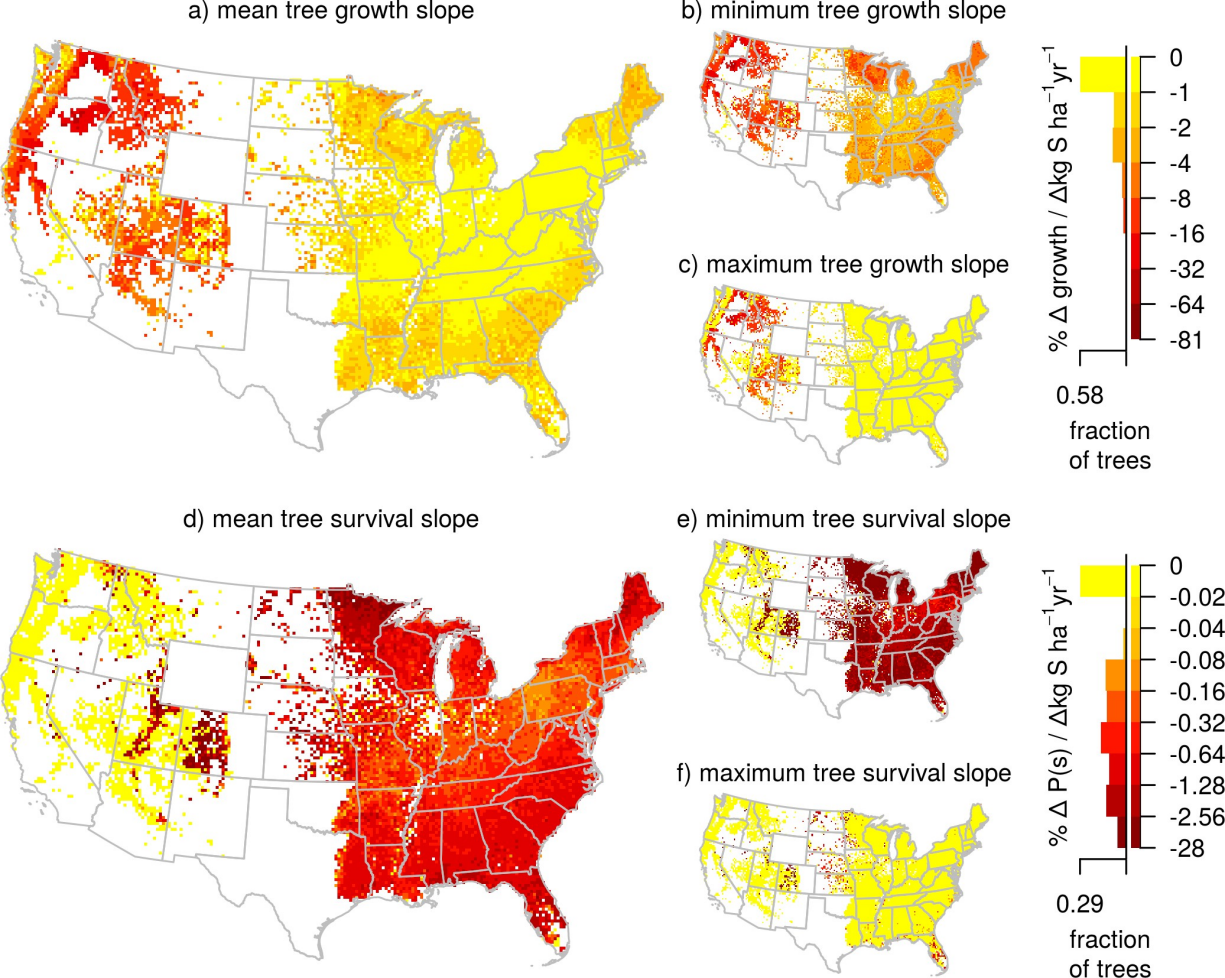
Relative change in growth and survival with S deposition of individual trees across the conterminous U.S. Maps show the relative mean, minimum, and maximum growth (a-c) and survival (d-f) responsiveness (percent change in growth or survival per change in annual S deposition rate) of trees within each 20 x 20 km pixel. Values were derived from the instantaneous rate of change in growth or survival with S deposition of the most parsimonious, species-specific models that allowed both N and S deposition (but did not necessarily contain N or S deposition). Red areas indicate where small increases in S deposition are associated with relatively large decreases in growth or survival. White areas are where no tree growth or survival data were available. Histograms indicate the fraction of individual trees by responsiveness to N deposition. Sample size for growth was 1,183,931 trees and for survival was 1,423,455 trees.

Most regions across the conterminous U.S. contained species that exhibited a negative slope between growth and/or survival with S deposition at current rates of S deposition (Fig 4). Tree growth and survival decreased in relation to S deposition at average relative rates of -1.6% per Δ kg S ha^-1^ yr^-1^ and -0.66% per Δ kg S ha^-1^ yr^-1^ respectively (Fig 4). The regions that showed the strongest negative relationship between growth and S deposition were in the western U.S. In contrast, the western U.S., outside the Central Rocky Mountains, contained no species that passed the VIF collinearity threshold for which S deposition was associated with changes in survival (Fig 4).

While the slope of the responses to N and S deposition give information on tree relationships to changes in deposition, it is important to note that the prediction is the slope at the current levels of atmospheric deposition. Therefore, a predicted future change in growth or survival depends on the direction of change in atmospheric S and/or N deposition; regions with positive instantaneous growth responses would have a predicted decrease in growth if atmosphere deposition decreases in the future, as observed in some regions of the conterminous U.S. [22].

### Correlations among predictors

While our analysis is comprehensive in its geographic coverage and diversity of temperate North American tree species, any gradient, space-for-time, analytic approach is associated with caveats. First, there are factors that could influence growth and survival that we did not account for in the analysis and that should be the focus of future efforts. These include, but are not limited to, ozone deposition and environmental variation, such as drought, that is not captured in the decadal mean annual temperature and precipitation covariates used here.

Another important caveat is the potential collinearity among predictors included and not included in the model due to spatial covariance. High collinearity presents challenges when using gradient analysis to quantify growth and survival associations with N and S deposition. Here we explicitly accounted for the influence of S deposition on growth and survival by including a S deposition term in the empirical equation and by using model selection criteria to determine whether including S deposition improved the explanatory power of the model. To provide context for the strength of evidence for individual species associations with atmospheric deposition, we assessed the collinearity between N deposition, S deposition, and climate by calculating the variance inflation factor (VIF), a multivariate measure of correlation.

**Fig 5:**
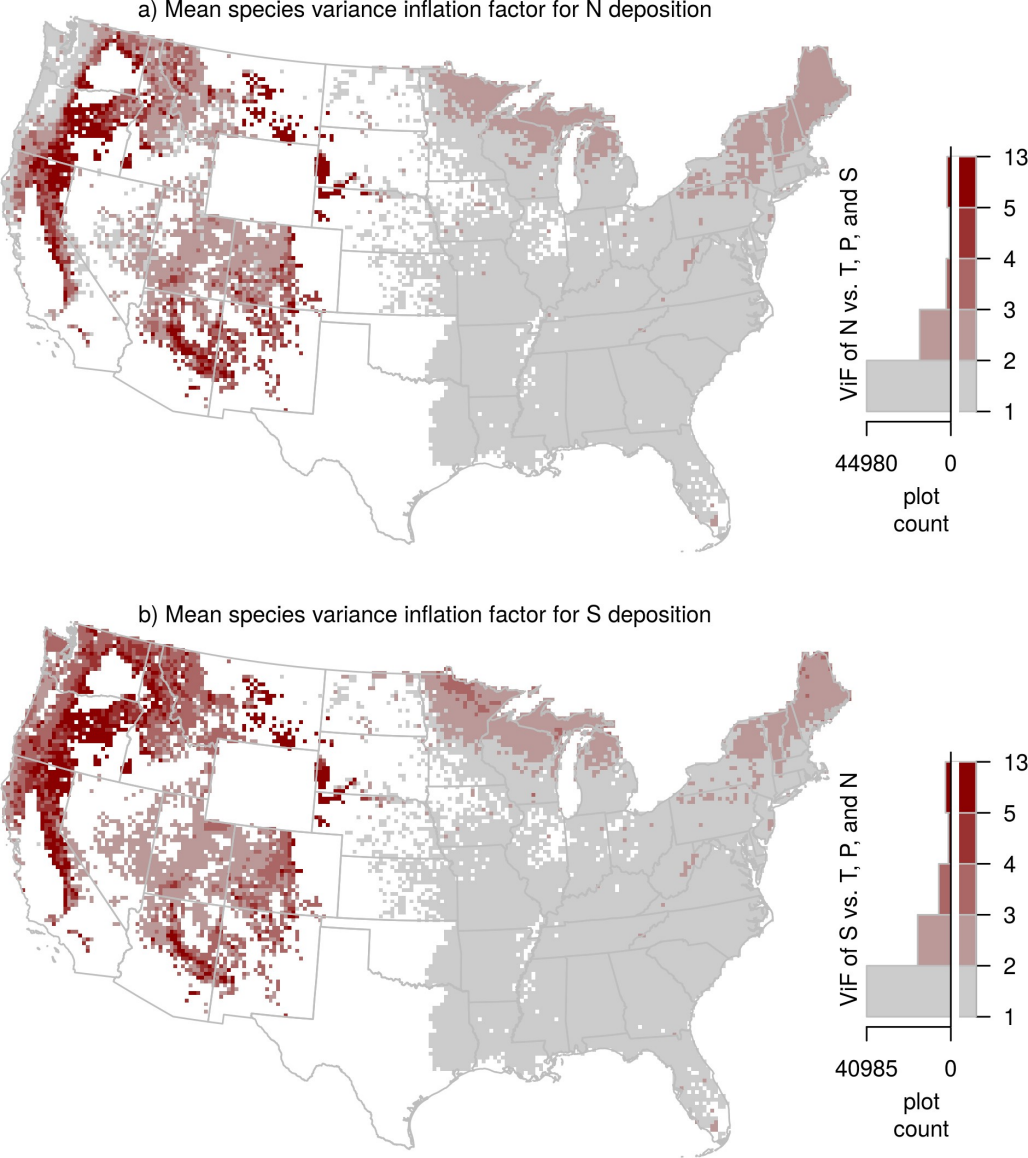
Spatial map of variance inflation factors (VIF): (a) VIF of the relationship between nitrogen (N) deposition and mean annual, temperature, mean annual precipitation, and sulfur (S) deposition; (b) VIF of the relationship between S deposition and mean annual, temperature, mean annual precipitation, and N deposition. VIF for N and S deposition was calculated for each of the 94 tree species, assigned to each tree in the FIA inventory based on species identity, and averaged across inventoried trees in each 20 x 20 pixel. Higher VIF values correspond to regions where the species present in the grid-cell have stronger covariation among predicters in the growth and survival models.

The vast majority of species (71 of 94) had VIF values for both N and S deposition that were less than or equal to 3 (the threshold for high collinearity used in Zuur et al. [33]), and all except two species (*Calocedrus decurrent and Juniperus occidentalis)* had VIF values below the rule of thumb (VIF < 10) in O’brien [32] (S1 Table). By providing the VIF factors for each species (S1 Table), the strength of evidence for separating N and S deposition influences can be compared among species. For example, the highest VIF values were in the western U.S., presumably due to shorter deposition gradients and similar sources for both N and S, and lower species richness (Fig 5). Therefore, the gradient approach likely provides more robust evidence for species responses in the eastern than western U.S. However, there were some western species for which the VIF and correlations were not problematic. It is important to note, as reiterated by O’brien [32], a high VIF score by itself does not mean that the relationship is incorrect, rather, it means that statistically we cannot be certain that we are able separate the influences of the different covariances with our dataset.

## Conclusions

Overall, our study is the first to analyze the relationship between N and S deposition and forest demographic rates across the conterminous U.S., a study area that includes high temperate tree species diversity. Previous efforts have focused on the Northeastern U.S. [6] or Eastern U.S. [14]. Our key findings are as follows: first, nearly all species displayed relationships between atmospheric N and/or S deposition and either growth or survival rates (66 of 71 species had significant relationships). Second, the most common response to N deposition involved a unimodal relationship whereby growth or survival peaked at rates of N deposition currently experienced by that species. However, growth relationships had peaks at much higher deposition rates than the survival responses, resulting in an average increase in growth and decrease in survival across the conterminous U.S. Third, there were species with increasing and decreasing relationships with N deposition that co-occurred across the conterminous U.S., thus potentially influencing tree biodiversity. Fourth, including S deposition improved the fit of the model 70% of species examined, thus advancing the work of Thomas et al. [6] that only examined N deposition. Finally, our confidence in the ability to disentangle the relationship between N deposition and demographic rates from relationships with other environmental covariates is strongest in the Eastern U.S.

Our findings have the potential to help inform ecosystem management and air pollution policy across the conterminous U.S. First, the species-specific relationships between atmospheric deposition and tree growth and survival can help set critical loads of atmospheric deposition designed to protect focal species. Prior critical loads for forest ecosystems have been largely set by either empirical studies at individual sites that are extrapolated to entire ecoregions [3], or studies of charge-balance of N and S to examine forest soil acidification [36]. Second, the species-specific responses across the conterminous U.S. can help guide the development of air pollution secondary standards by identifying which species and regions are most vulnerable to N deposition. In particular, development of response curves allows for a full dose response to N deposition that can support the development of air pollution standards, rather than point estimates of thresholds with no reported underlying dose-response curve. Finally, our results suggest that forest demographics likely have been changing in the U.S. from N and S deposition for decades, in combination with other factors such as pests, fire, and climate change.

## Supporting information

S1 Table Species-level sample characteristics and summarized results. (XLSX)

S2 Table Full growth equations. (PDF)

S3 Table Full survival equations. (PDF)

S4 Table Parameters for each species model. (XLSX)

S1 Fig Species relationships between atmospheric deposition and growth and survival. (PDF)
