# Correction: Growth and survival relationships of 71 tree species with nitrogen and sulfur deposition across the conterminous U.S.

**DOI:** 10.1371/journal.pone.0212984

**Published:** 2019-02-22

**Authors:** Kevin J. Horn, R. Quinn Thomas, Christopher M. Clark, Linda H. Pardo, Mark E. Fenn, Gregory B. Lawrence, Steven S. Perakis, Erica A. H. Smithwick, Douglas Baldwin, Sabine Braun, Annika Nordin, Charles H. Perry, Jennifer N. Phelan, Paul G. Schaberg, Samuel B. St. Clair, Richard Warby, Shaun Watmough

In the Species-level relationships with N and S deposition subsection of the Results and Discussion, there is an error in the final sentence of the sixth paragraph. The correct sentence is: All six of the species with declining survival as N deposition increased were hardwood species from the *Carya*, *Quercus*, and *Ulmus* genera. Four of the six species were ectomycorrhizal symbionts—supporting previous studies that found arbuscular mycorrhizal symbionts on average fared better than ectomycorrhizal symbionts under elevated N deposition [6, 34, 35].

There are errors in the Funding statement. The correct Funding statement is as follows: Funding for this effort was provided by U.S. Environmental Protection Agency (Contract EP-W- 11–029; Work Assignment No. 4–16), U.S. Geological Survey (U.S. Geological Survey Powell Center Program), and the National Park Service (Task Agreement Number P18AC01685). CMC (EPA) and GBL (USGS) contributed to the study design, interpretation now the analysis, decision to publish, and preparation of the manuscript. The Warby Group LLC provided support in the form of salaries for author WB, but did not have any additional role in the study design, data collection and analysis, decision to publish, or preparation of the manuscript. The specific roles of these authors are articulated in the ‘author contributions’ section.
