# Supplementary material for: Growth and survival relationships of 71 tree species with nitrogen and sulfur deposition across the conterminous U.S
Source: PLoS One. 2018 Oct 18;13(10):e0205296. doi: 10.1371/journal.pone.0205296 (PMC6193662; doi:10.1371/journal.pone.0205296)
Supplement: S2 Table — (PDF) [file pone.0205296.s002.pdf]

Table S2. Tree growth equations. Growth equations used to estimate the spatial relationship between aboveground tree growth and atmospheric deposition. *Note: BAL is the basal area of all trees greater than the tree of interest within the subplot divided by 4.*

$$G = a \times size^{z} \times e^{\left[a_2 BAL + a_3 \ln(BA)\right]} \times e^{\frac{-1}{2}\left(\frac{\ln(T/t_1)}{t_2}\right)^2} \times e^{\frac{-1}{2}\left(\frac{\ln(P/p_1)}{p_2}\right)^2} \times e^{\frac{-1}{2}\left(\frac{\ln(S/s_1)}{s_2}\right)^2} \times e^{\frac{-1}{2}\left(\frac{\ln(N/n_1)}{n_2}\right)^2} \quad (1)$$

$$G = a \times size^{z} \times e^{\left[a_2 BAL + a_3 \ln(BA)\right]} \times e^{\frac{-1}{2}\left(\frac{\ln(T/t_1)}{t_2}\right)^2} \times e^{\frac{-1}{2}\left(\frac{\ln(P/p_1)}{p_2}\right)^2} \times e^{\frac{-1}{2}\left(\frac{\ln(S/s_1)}{s_2}\right)^2} \quad (2)$$

$$G = a \times size^{z} \times e^{\left[a_2 BAL + a_3 \ln(BA)\right]} \times e^{\frac{-1}{2}\left(\frac{\ln(T/t_1)}{t_2}\right)^2} \times e^{\frac{-1}{2}\left(\frac{\ln(P/p_1)}{p_2}\right)^2} \times e^{\frac{-1}{2}\left(\frac{\ln(N/n_1)}{n_2}\right)^2} \quad (3)$$

$$G = a \times size^{z} \times e^{\left[a_2 BAL + a_3 \ln(BA)\right]} \times e^{\frac{-1}{2}\left(\frac{\ln(T/t_1)}{t_2}\right)^2} \times e^{\frac{-1}{2}\left(\frac{\ln(P/p_1)}{p_2}\right)^2} \quad (4)$$

$$G = a \quad (5)$$
