# Supplementary material for: Growth and survival relationships of 71 tree species with nitrogen and sulfur deposition across the conterminous U.S
Source: PLoS One. 2018 Oct 18;13(10):e0205296. doi: 10.1371/journal.pone.0205296 (PMC6193662; doi:10.1371/journal.pone.0205296)
Supplement: S3 Table — (PDF) [file pone.0205296.s003.pdf]

Table S3. Tree survival equations. Survival equations used to estimate the spatial relationship between tree survival and atmospheric deposition. *Note: BAL is the basal area of all trees greater than the tree of interest within the subplot divided by 4.*

$$P(s)=\left[a\times\left(1-zc_1\cdot e^{-zc_2\cdot size}\right)\times e^{-zc_3\cdot size^{zc_4}}\times e^{-br_1\left(BA_{ratio}^{br_2}\right)\cdot\left(BA^{br_3}\right)}\times e^{\frac{-1}{2}\left(\frac{\ln(T/t_1)}{t_2}\right)^2}\times e^{\frac{-1}{2}\left(\frac{\ln(P/p_1)}{p_2}\right)^2}\times e^{\frac{-1}{2}\left(\frac{\ln(S/s_1)}{s_2}\right)^2}\times e^{\frac{-1}{2}\left(\frac{\ln(N/n_1)}{n_2}\right)^2}\right]^{\text{time}} \quad (1)$$

$$P(s)=\left[a\times\left(1-zc_1\cdot e^{-zc_2\cdot size}\right)\times e^{-zc_3\cdot size^{zc_4}}\times e^{-br_1\left(BA_{ratio}^{br_2}\right)\cdot\left(BA^{br_3}\right)}\times e^{\frac{-1}{2}\left(\frac{\ln(T/t_1)}{t_2}\right)^2}\times e^{\frac{-1}{2}\left(\frac{\ln(P/p_1)}{p_2}\right)^2}\times e^{\frac{-1}{2}\left(\frac{\ln(S/s_1)}{s_2}\right)^2}\right]^{\text{time}} \quad (2)$$

$$P(s)=\left[a\times\left(1-zc_1\cdot e^{-zc_2\cdot size}\right)\times e^{-zc_3\cdot size^{zc_4}}\times e^{-br_1\left(BA_{ratio}^{br_2}\right)\cdot\left(BA^{br_3}\right)}\times e^{\frac{-1}{2}\left(\frac{\ln(T/t_1)}{t_2}\right)^2}\times e^{\frac{-1}{2}\left(\frac{\ln(P/p_1)}{p_2}\right)^2}\times e^{\frac{-1}{2}\left(\frac{\ln(N/n_1)}{n_2}\right)^2}\right]^{\text{time}} \quad (3)$$

$$P(s)=\left[a\times\left(1-zc_1\cdot e^{-zc_2\cdot size}\right)\times e^{-zc_3\cdot size^{zc_4}}\times e^{-br_1\left(BA_{ratio}^{br_2}\right)\cdot\left(BA^{br_3}\right)}\times e^{\frac{-1}{2}\left(\frac{\ln(T/t_1)}{t_2}\right)^2}\times e^{\frac{-1}{2}\left(\frac{\ln(P/p_1)}{p_2}\right)^2}\right]^{\text{time}} \quad (4)$$

$$P(s)=\left[a\times e^{\frac{-1}{2}\left(\frac{\ln(\text{size}/z_1)}{z_2}\right)^2}\times e^{\frac{-1}{2}\left(\frac{\ln(BA/ba_1)}{ba_2}\right)^2+\frac{-1}{2}\left(\frac{\ln((BAL+1)/(bl_1+1))}{bl_2}\right)^2}\times e^{\frac{-1}{2}\left(\frac{\ln(T/t_1)}{t_2}\right)^2}\times e^{\frac{-1}{2}\left(\frac{\ln(P/p_1)}{p_2}\right)^2}\times e^{\frac{-1}{2}\left(\frac{\ln(S/s_1)}{s_2}\right)^2}\times e^{\frac{-1}{2}\left(\frac{\ln(N/n_1)}{n_2}\right)^2}\right]^{\text{time}} \quad (5)$$

$$P(s)=\left[a\times e^{\frac{-1}{2}\left(\frac{\ln(\text{size}/z_1)}{z_2}\right)^2}\times e^{\frac{-1}{2}\left(\frac{\ln(BA/ba_1)}{ba_2}\right)^2+\frac{-1}{2}\left(\frac{\ln((BAL+1)/(bl_1+1))}{bl_2}\right)^2}\times e^{\frac{-1}{2}\left(\frac{\ln(T/t_1)}{t_2}\right)^2}\times e^{\frac{-1}{2}\left(\frac{\ln(P/p_1)}{p_2}\right)^2}\times e^{\frac{-1}{2}\left(\frac{\ln(S/s_1)}{s_2}\right)^2}\right]^{\text{time}} \quad (6)$$

$$P(s)=\left[a\times e^{\frac{-1}{2}\left(\frac{\ln(\text{size}/z_1)}{z_2}\right)^2}\times e^{\frac{-1}{2}\left(\frac{\ln(BA/ba_1)}{ba_2}\right)^2+\frac{-1}{2}\left(\frac{\ln((BAL+1)/(bl_1+1))}{bl_2}\right)^2}\times e^{\frac{-1}{2}\left(\frac{\ln(T/t_1)}{t_2}\right)^2}\times e^{\frac{-1}{2}\left(\frac{\ln(P/p_1)}{p_2}\right)^2}\times e^{\frac{-1}{2}\left(\frac{\ln(N/n_1)}{n_2}\right)^2}\right]^{\text{time}} \quad (7)$$

$$P(s)=\left[a\times e^{\frac{-1}{2}\left(\frac{\ln(\text{size}/z_1)}{z_2}\right)^2}\times e^{\frac{-1}{2}\left(\frac{\ln(BA/ba_1)}{ba_2}\right)^2+\frac{-1}{2}\left(\frac{\ln((BAL+1)/(bl_1+1))}{bl_2}\right)^2}\times e^{\frac{-1}{2}\left(\frac{\ln(T/t_1)}{t_2}\right)^2}\times e^{\frac{-1}{2}\left(\frac{\ln(P/p_1)}{p_2}\right)^2}\right]^{\text{time}} \quad (8)$$

$$P(s)=a^{\text{time}} \quad (9)$$
