# Supplementary material for: Growth and survival relationships of 71 tree species with nitrogen and sulfur deposition across the conterminous U.S
Source: PLoS One. 2018 Oct 18;13(10):e0205296. doi: 10.1371/journal.pone.0205296 (PMC6193662; doi:10.1371/journal.pone.0205296)
Supplement: S1 Fig — (PDF) [file pone.0205296.s005.pdf]

Fig S1. Tree growth and survival association curves with nitrogen and sulfur deposition by species. Each curve represents the annual growth (top graphs) or 10 year survival (bottom graphs) associations with nitrogen (N; left panels) or sulfur (S, right panels) deposition across the conterminous United States. Sample sizes and Pearson's correlation coefficient between N and S deposition for growth and survival analyses are given above the upper and lower panel respectively. Shaded areas represent the 2 factor support interval for the response curves.

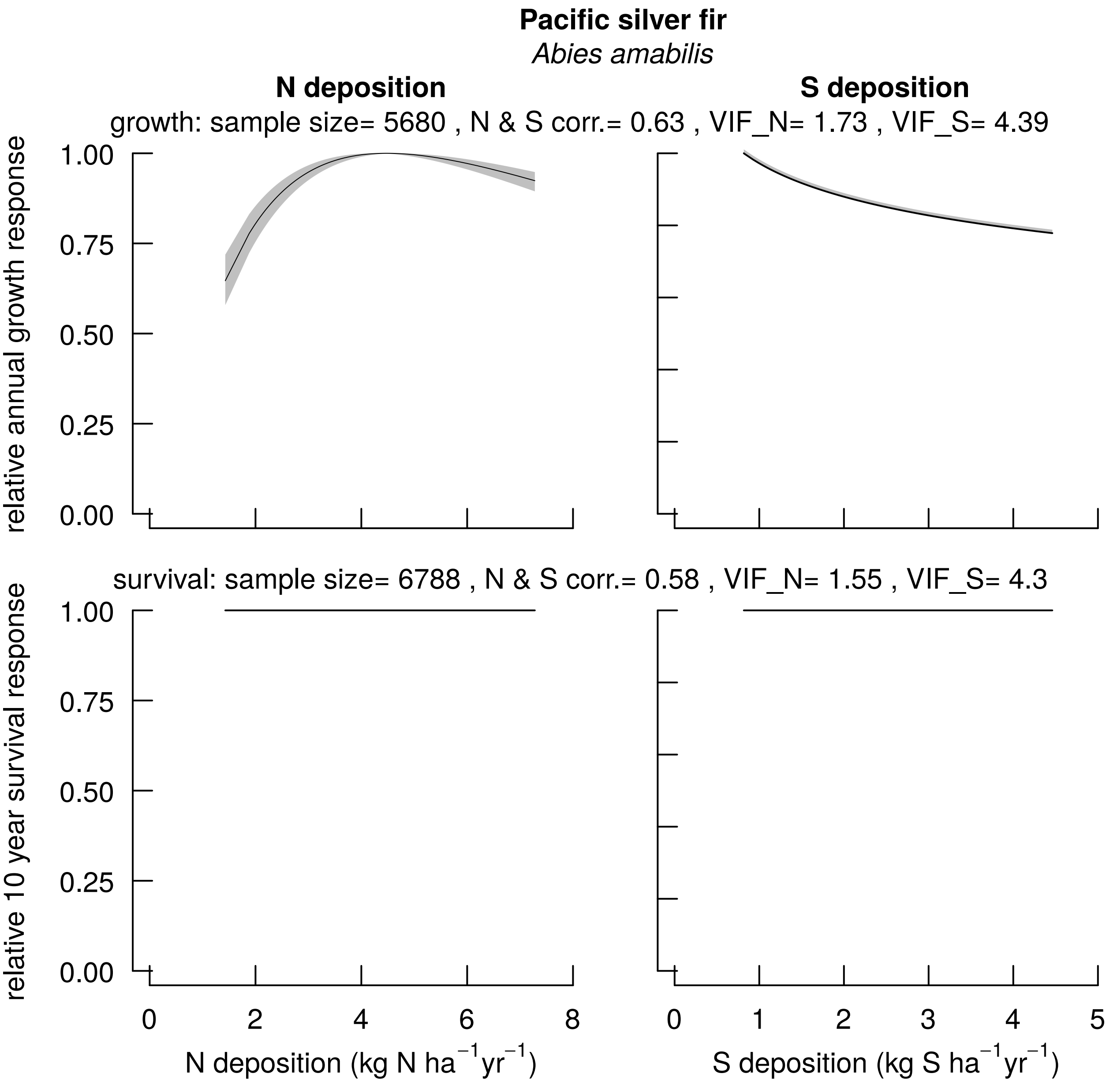
Pacific silver fir
Abies amabilis
N deposition
S deposition
growth: sample size= 5680 , N & S corr.= 0.63 , VIF_N= 1.73 , VIF_S= 4.39
survival: sample size= 6788 , N & S corr.= 0.58 , VIF_N= 1.55 , VIF_S= 4.3
relative annual growth response
relative 10 year survival response
1.00
0.75
0.50
0.25
0.00
0
2
4
6
8
1
3
5
N deposition (kg N ha−1yr−1)
S deposition (kg S ha−1yr−1)

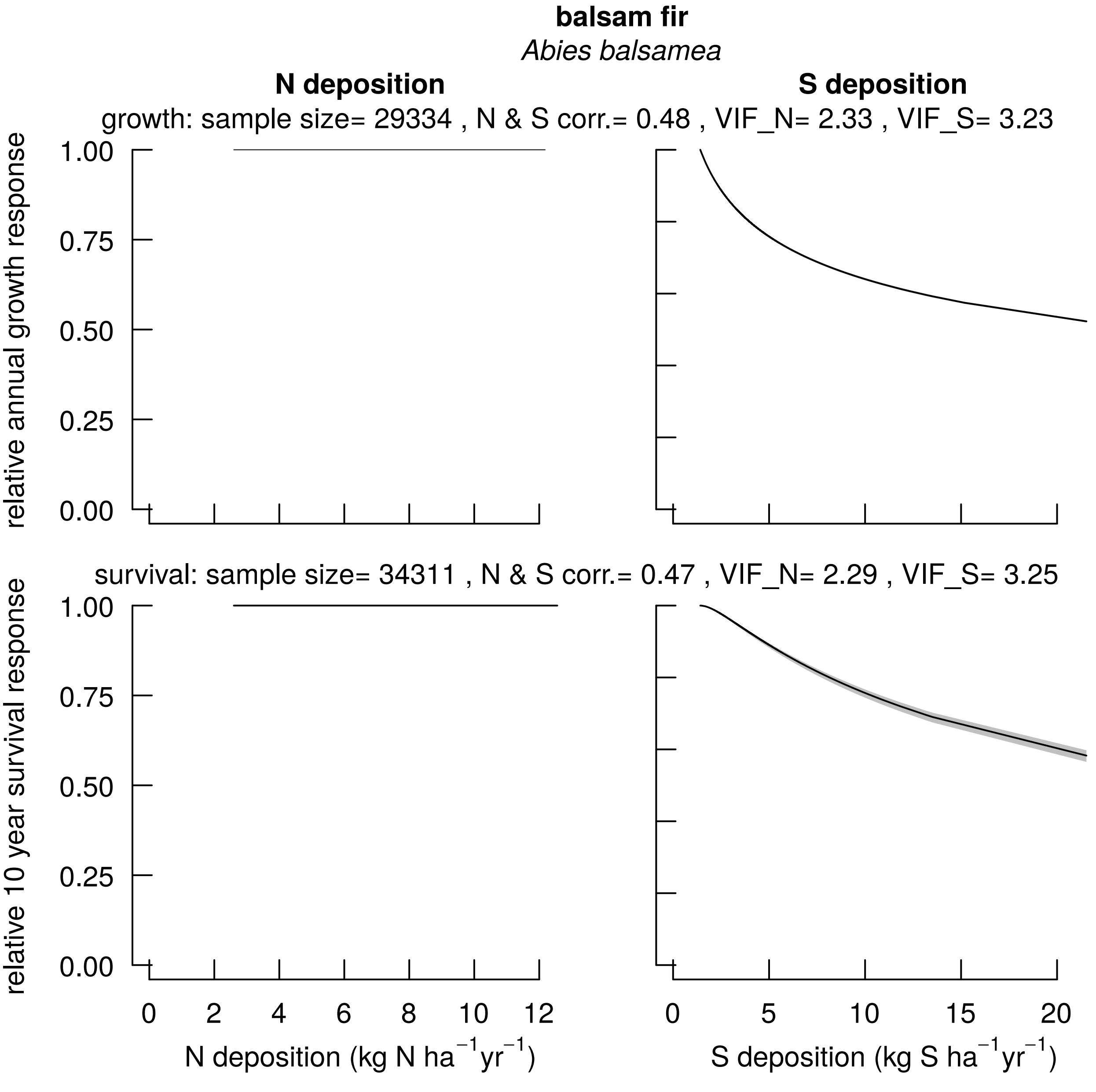
balsam fir
Abies balsamea
N deposition
S deposition
growth: sample size= 29334 , N & S corr.= 0.48 , VIF_N= 2.33 , VIF_S= 3.23
relative annual growth response
1.00
0.75
0.50
0.25
0.00
survival: sample size= 34311 , N & S corr.= 0.47 , VIF_N= 2.29 , VIF_S= 3.25
relative 10 year survival response
1.00
0.75
0.50
0.25
0.00
0 2 4 6 8 10 12
0 5 10 15 20
N deposition (kg N ha^{-1}yr^{-1})
S deposition (kg S ha^{-1}yr^{-1})

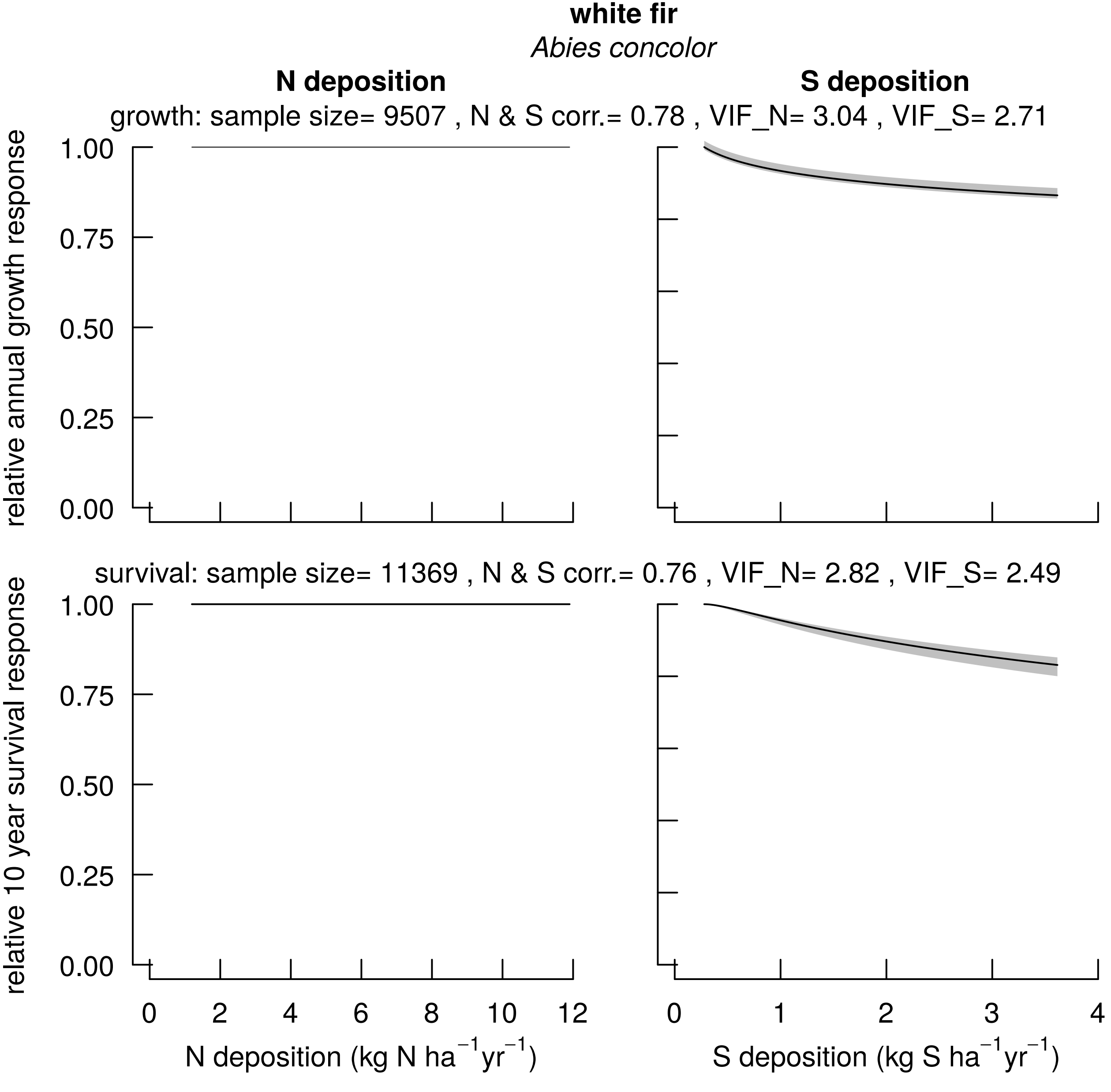
white fir
Abies concolor
N deposition
S deposition
growth: sample size= 9507 , N & S corr.= 0.78 , VIF_N= 3.04 , VIF_S= 2.71
relative annual growth response
1.00
0.75
0.50
0.25
0.00
survival: sample size= 11369 , N & S corr.= 0.76 , VIF_N= 2.82 , VIF_S= 2.49
relative 10 year survival response
1.00
0.75
0.50
0.25
0.00
0 2 4 6 8 10 12
N deposition (kg N ha^{-1}yr^{-1})
0 1 2 3 4
S deposition (kg S ha^{-1}yr^{-1})

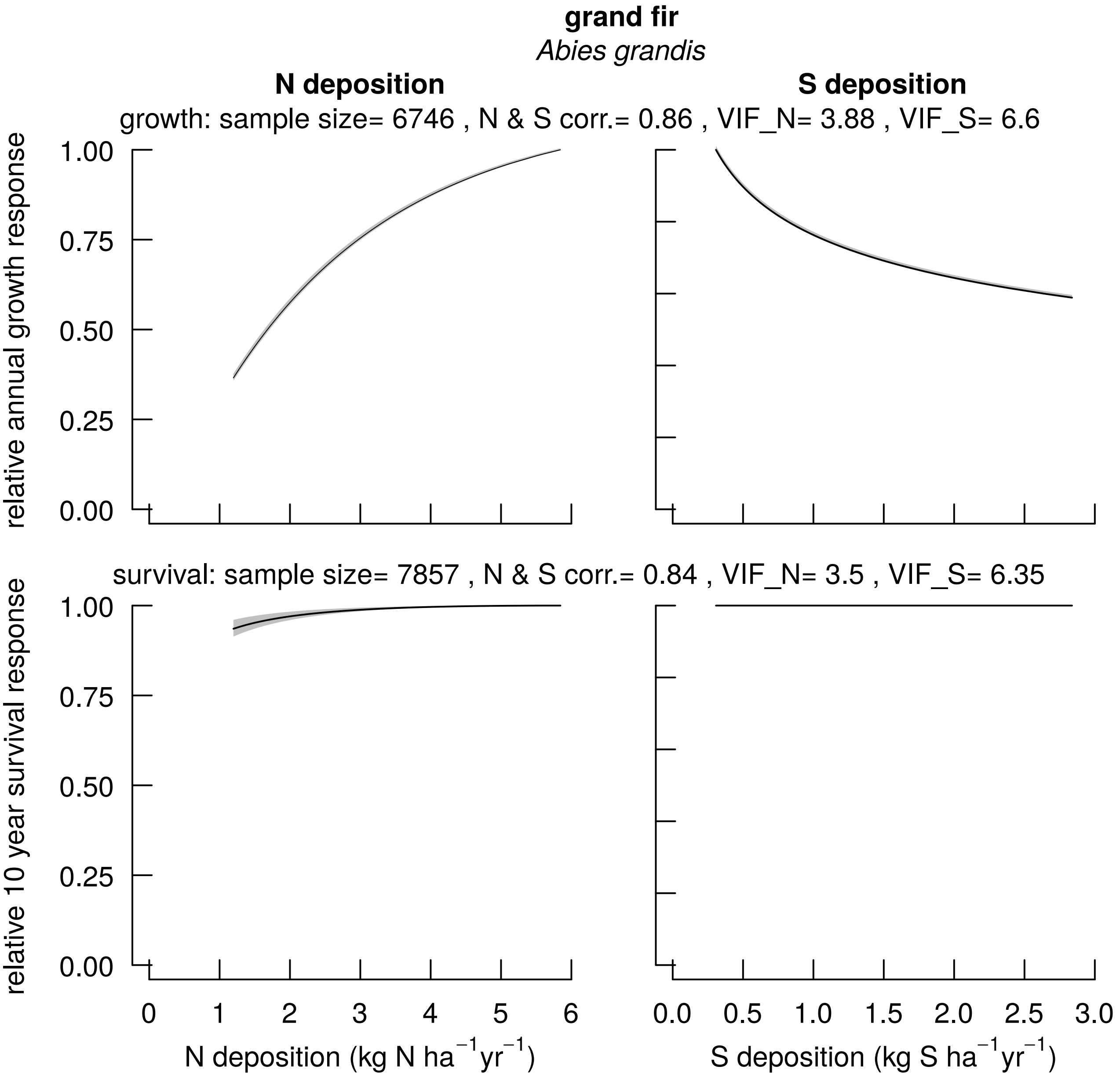
grand fir
Abies grandis
N deposition
S deposition
growth: sample size= 6746 , N & S corr.= 0.86 , VIF_N= 3.88 , VIF_S= 6.6
survival: sample size= 7857 , N & S corr.= 0.84 , VIF_N= 3.5 , VIF_S= 6.35
relative annual growth response
relative 10 year survival response
1.00
0.75
0.50
0.25
0.00
0 1 2 3 4 5 6
0.0 0.5 1.0 1.5 2.0 2.5 3.0
N deposition (kg N ha^{-1}yr^{-1})
S deposition (kg S ha^{-1}yr^{-1})

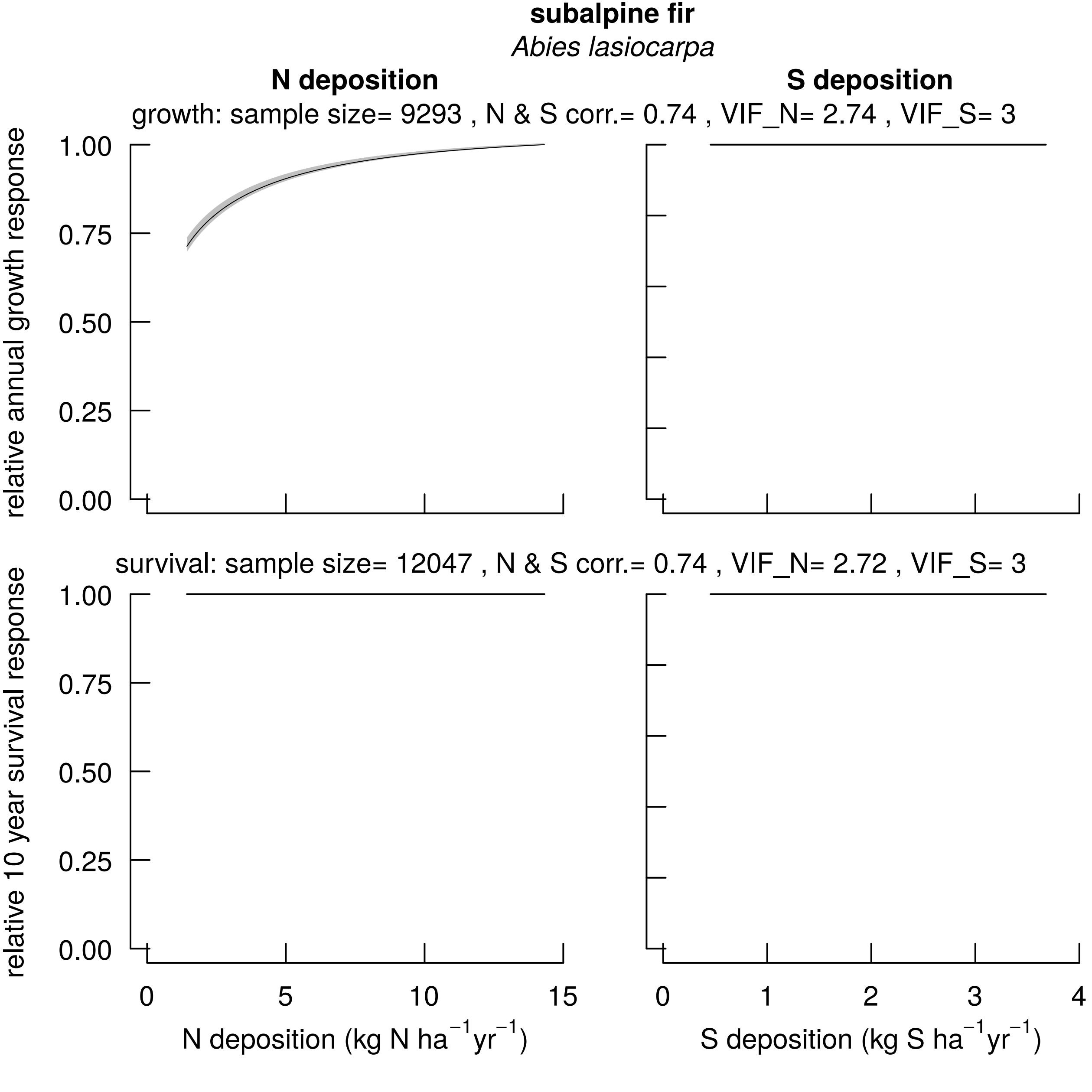

subalpine fir
Abies lasiocarpa
N deposition
S deposition
growth: sample size= 9293 , N & S corr.= 0.74 , VIF_N= 2.74 , VIF_S= 3
survival: sample size= 12047 , N & S corr.= 0.74 , VIF_N= 2.72 , VIF_S= 3
relative annual growth response
relative 10 year survival response
1.00
0.75
0.50
0.25
0.00
0
5
10
15
0
1
2
3
4
N deposition (kg N ha^{-1}yr^{-1})
S deposition (kg S ha^{-1}yr^{-1})

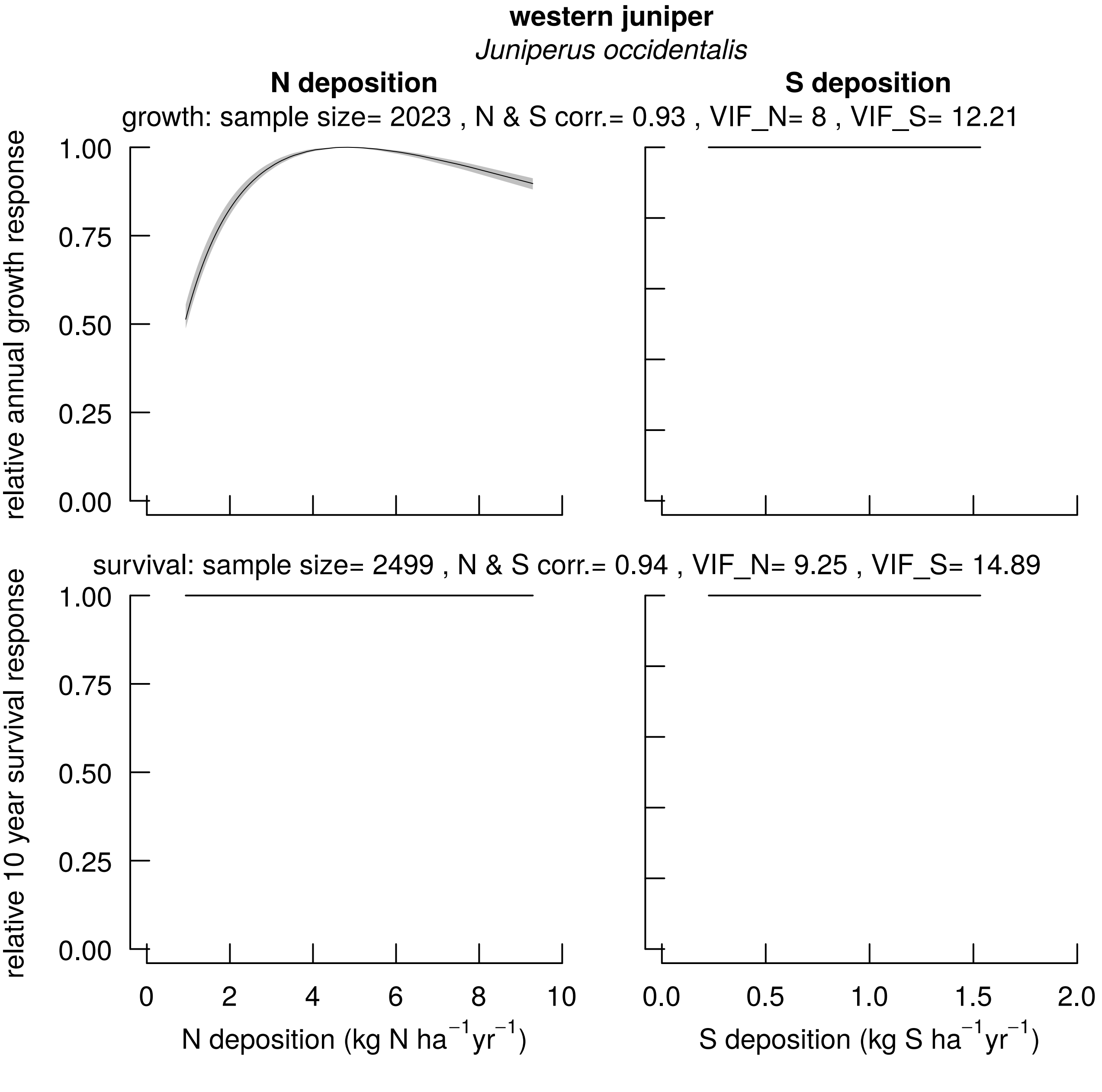

western juniper
Juniperus occidentalis
N deposition
S deposition
growth: sample size= 2023 , N & S corr.= 0.93 , VIF_N= 8 , VIF_S= 12.21
relative annual growth response
1.00
0.75
0.50
0.25
0.00
survival: sample size= 2499 , N & S corr.= 0.94 , VIF_N= 9.25 , VIF_S= 14.89
relative 10 year survival response
1.00
0.75
0.50
0.25
0.00
0
2
4
6
8
10
0.0
0.5
1.0
1.5
2.0
N deposition (kg N ha^{-1}yr^{-1})
S deposition (kg S ha^{-1}yr^{-1})

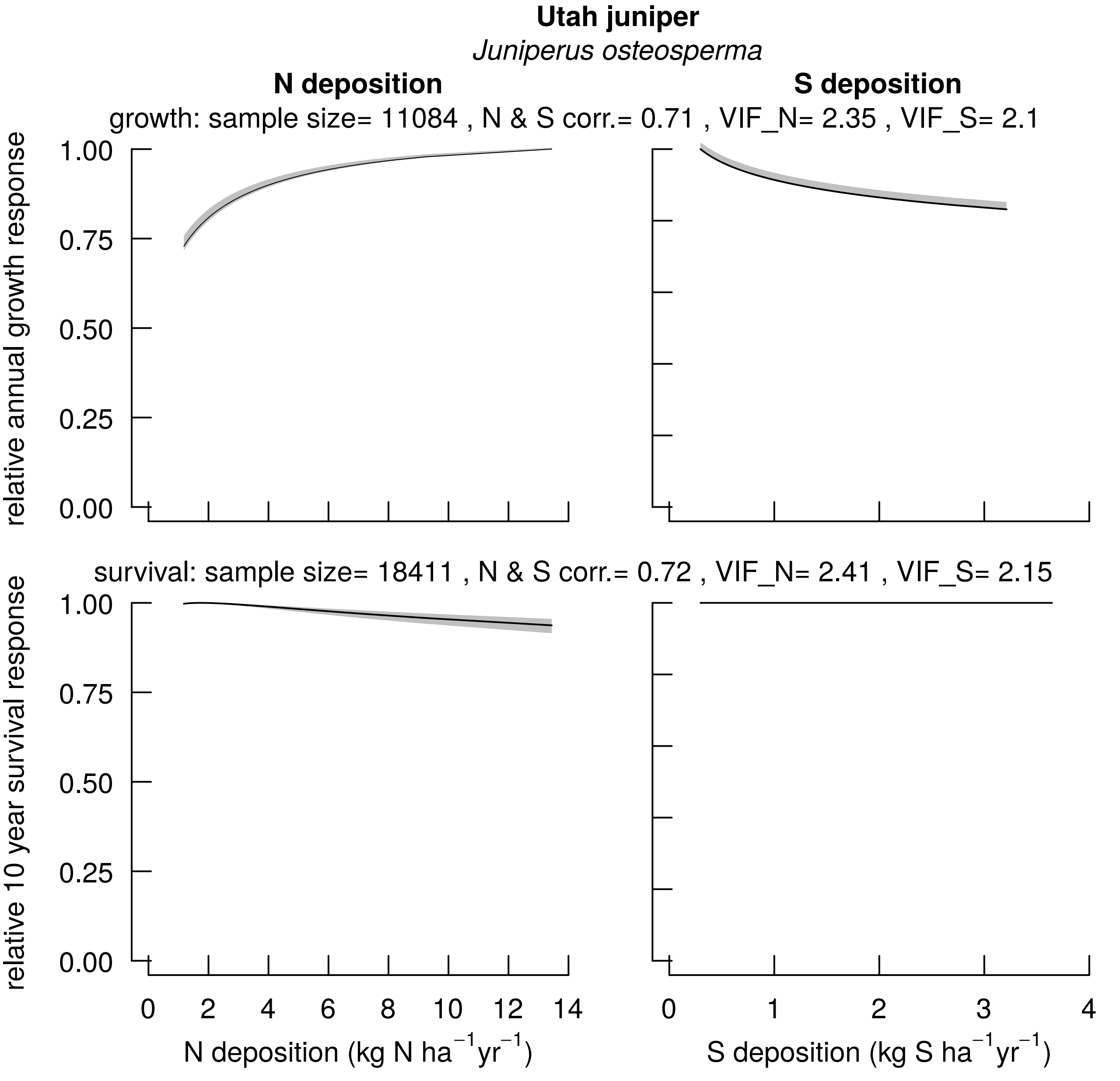

Utah juniper
Juniperus osteosperma
N deposition
S deposition
growth: sample size= 11084 , N & S corr.= 0.71 , VIF_N= 2.35 , VIF_S= 2.1
relative annual growth response
1.00
0.75
0.50
0.25
0.00
survival: sample size= 18411 , N & S corr.= 0.72 , VIF_N= 2.41 , VIF_S= 2.15
relative 10 year survival response
1.00
0.75
0.50
0.25
0.00
0 2 4 6 8 10 12 14
0 1 2 3 4
N deposition (kg N ha^{-1}yr^{-1})
S deposition (kg S ha^{-1}yr^{-1})

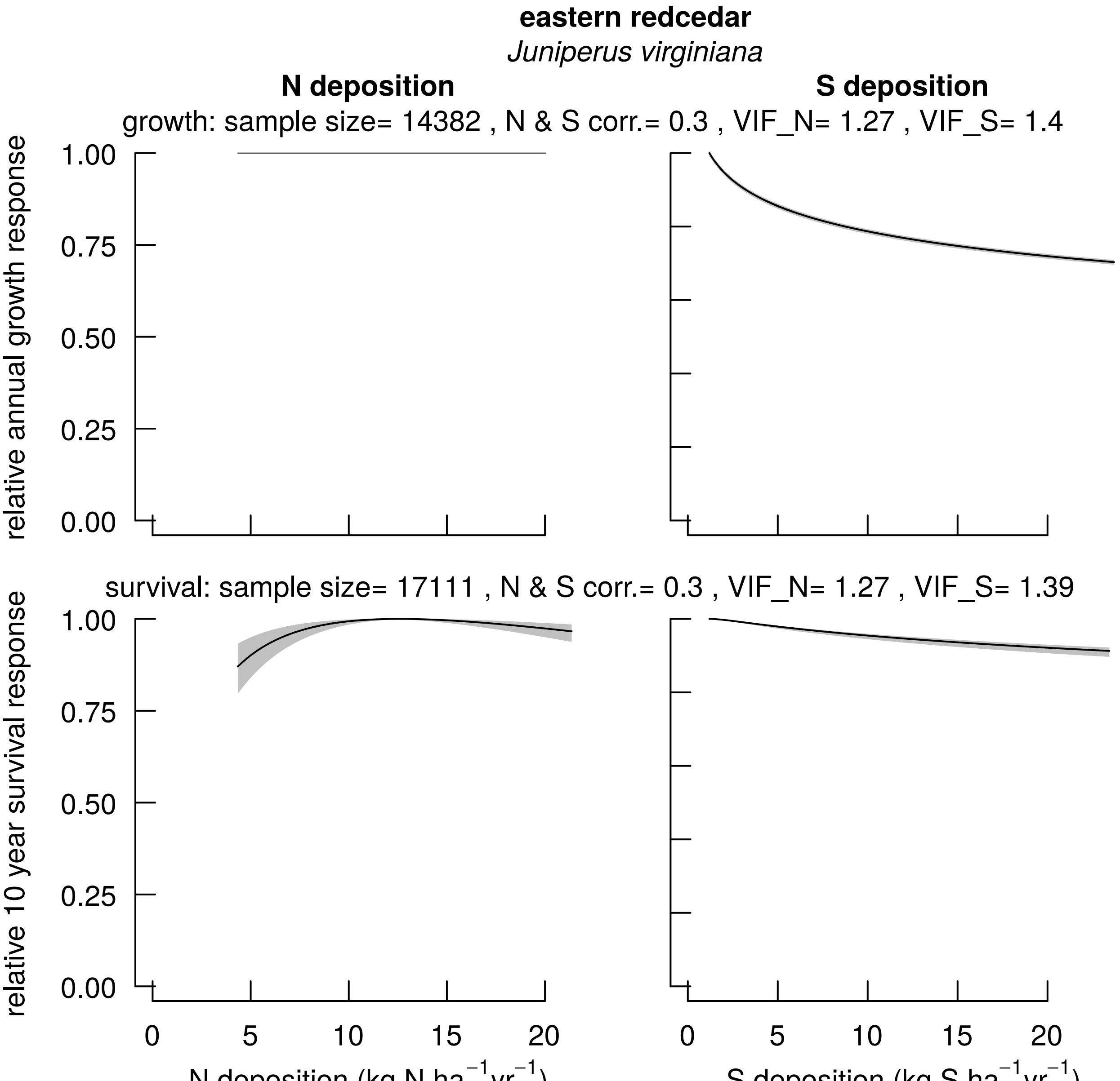
eastern redcedar
Juniperus virginiana
N deposition
S deposition
growth: sample size= 14382 , N & S corr.= 0.3 , VIF_N= 1.27 , VIF_S= 1.4
relative annual growth response
1.00
0.75
0.50
0.25
0.00
survival: sample size= 17111 , N & S corr.= 0.3 , VIF_N= 1.27 , VIF_S= 1.39
relative 10 year survival response
1.00
0.75
0.50
0.25
0.00
0
5
10
15
20
N deposition (kg N ha−1yr−1)
0
5
10
15
20
S deposition (kg S ha−1yr−1)

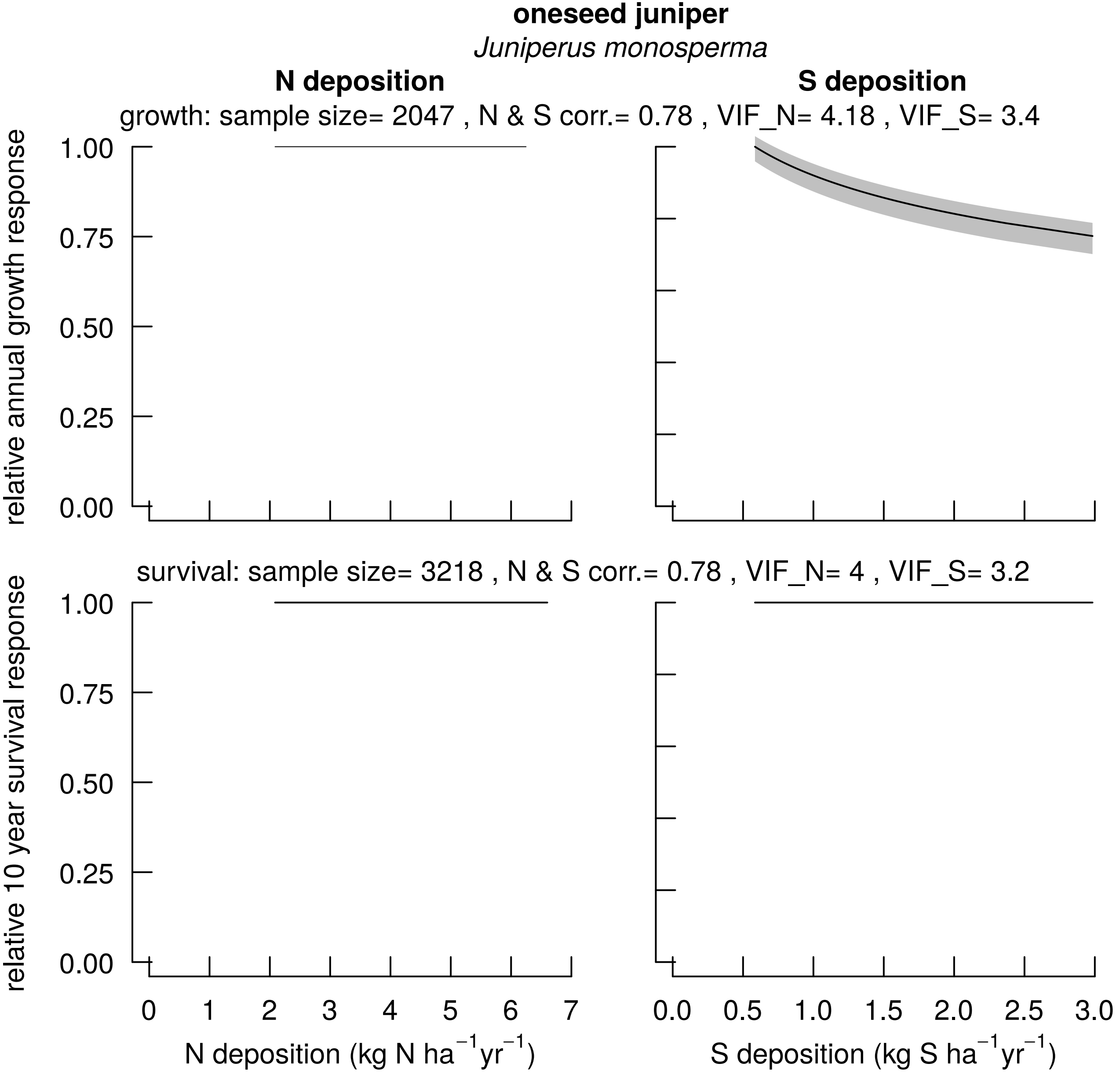

oneseed juniper
Juniperus monosperma
N deposition
S deposition
growth: sample size= 2047 , N & S corr.= 0.78 , VIF_N= 4.18 , VIF_S= 3.4
survival: sample size= 3218 , N & S corr.= 0.78 , VIF_N= 4 , VIF_S= 3.2
relative annual growth response
relative 10 year survival response
1.00
0.75
0.50
0.25
0.00
0 1 2 3 4 5 6 7
0.0 0.5 1.0 1.5 2.0 2.5 3.0
N deposition (kg N ha^{-1}yr^{-1})
S deposition (kg S ha^{-1}yr^{-1})

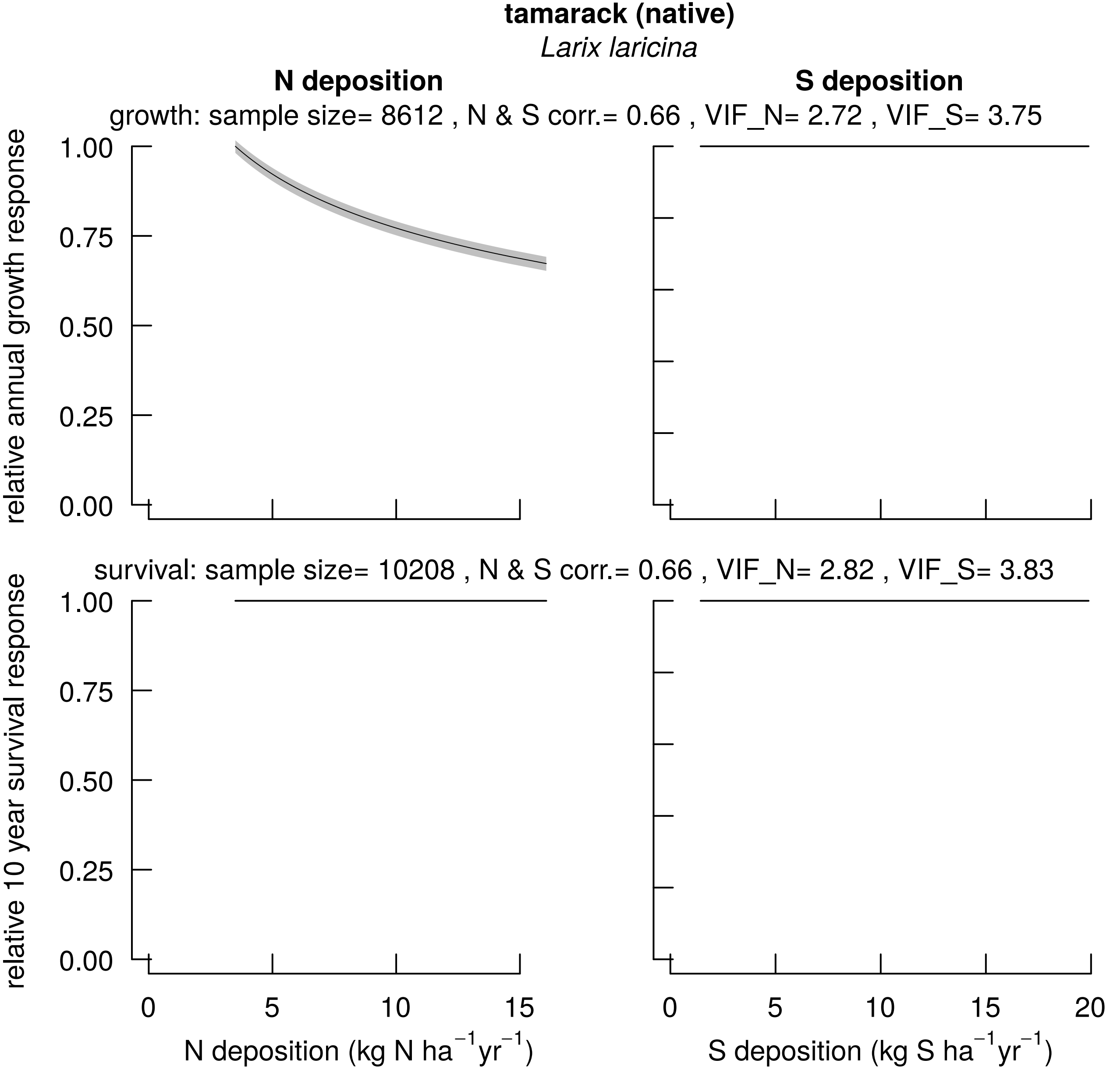
tamarack (native)
Larix laricina
N deposition
S deposition
growth: sample size= 8612 , N & S corr.= 0.66 , VIF_N= 2.72 , VIF_S= 3.75
relative annual growth response
1.00
0.75
0.50
0.25
0.00
survival: sample size= 10208 , N & S corr.= 0.66 , VIF_N= 2.82 , VIF_S= 3.83
relative 10 year survival response
0
5
10
15
20
N deposition (kg N ha^{-1}yr^{-1})
S deposition (kg S ha^{-1}yr^{-1})

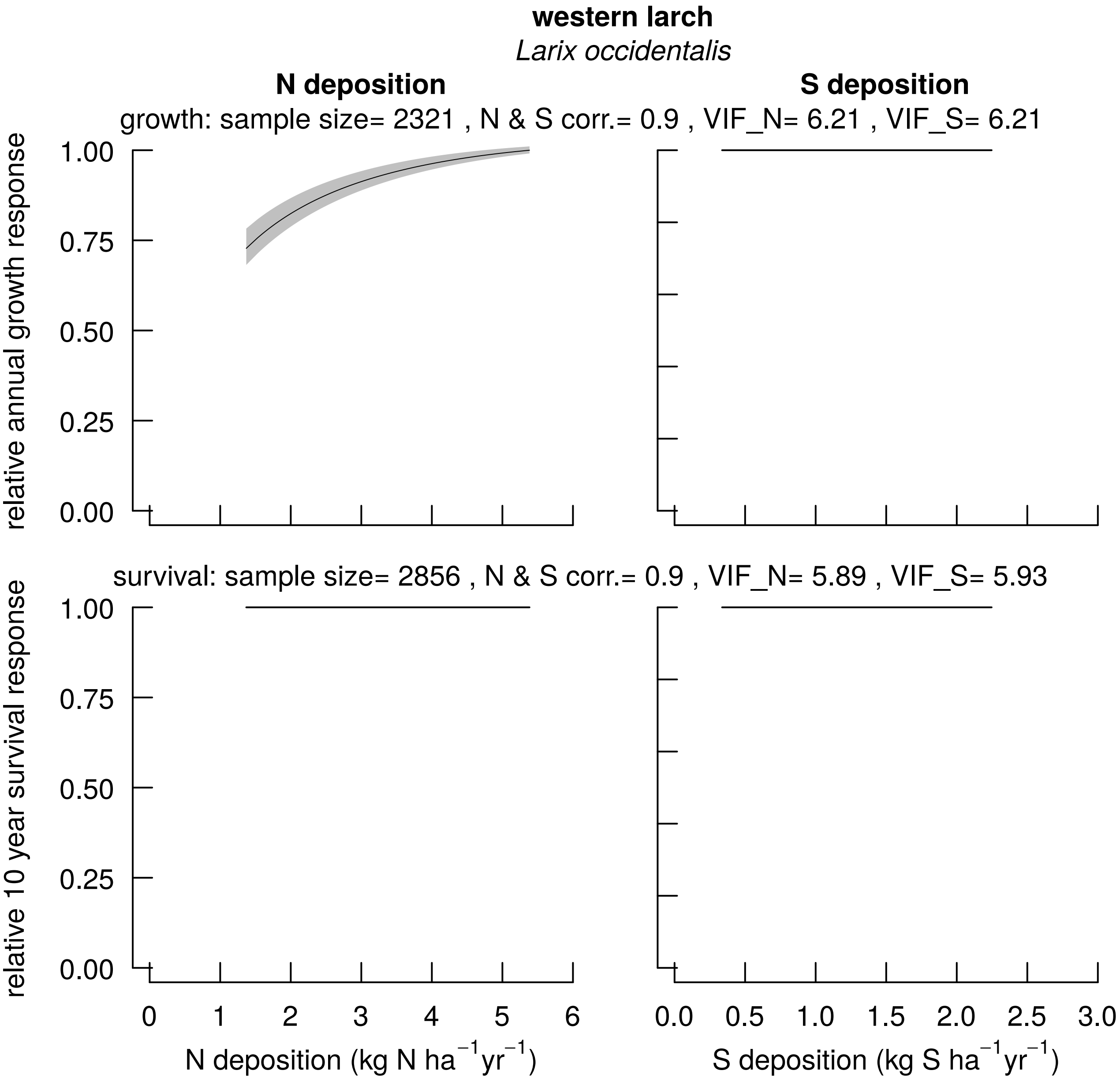
western larch
Larix occidentalis
N deposition
S deposition
growth: sample size= 2321 , N & S corr.= 0.9 , VIF_N= 6.21 , VIF_S= 6.21
relative annual growth response
1.00
0.75
0.50
0.25
0.00
survival: sample size= 2856 , N & S corr.= 0.9 , VIF_N= 5.89 , VIF_S= 5.93
relative 10 year survival response
1.00
0.75
0.50
0.25
0.00
0 1 2 3 4 5 6
0.0 0.5 1.0 1.5 2.0 2.5 3.0
N deposition (kg N ha^{-1}yr^{-1})
S deposition (kg S ha^{-1}yr^{-1})

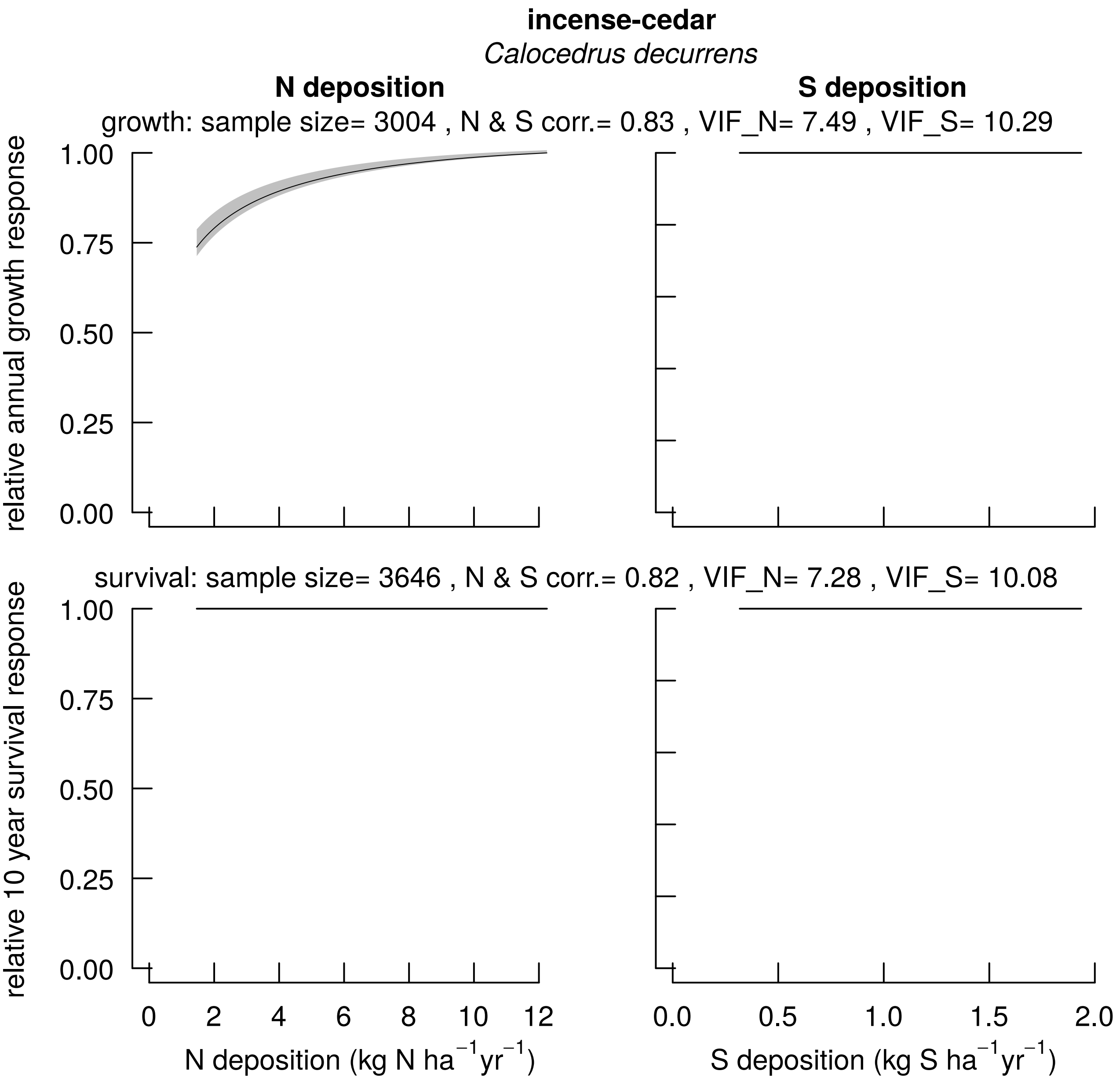

incense-cedar
Calocedrus decurrens
N deposition
S deposition
growth: sample size= 3004 , N & S corr.= 0.83 , VIF_N= 7.49 , VIF_S= 10.29
relative annual growth response
1.00
0.75
0.50
0.25
0.00
survival: sample size= 3646 , N & S corr.= 0.82 , VIF_N= 7.28 , VIF_S= 10.08
relative 10 year survival response
1.00
0.75
0.50
0.25
0.00
0
2
4
6
8
10
12
0.0
0.5
1.0
1.5
2.0
N deposition (kg N ha⁻¹yr⁻¹)
S deposition (kg S ha⁻¹yr⁻¹)

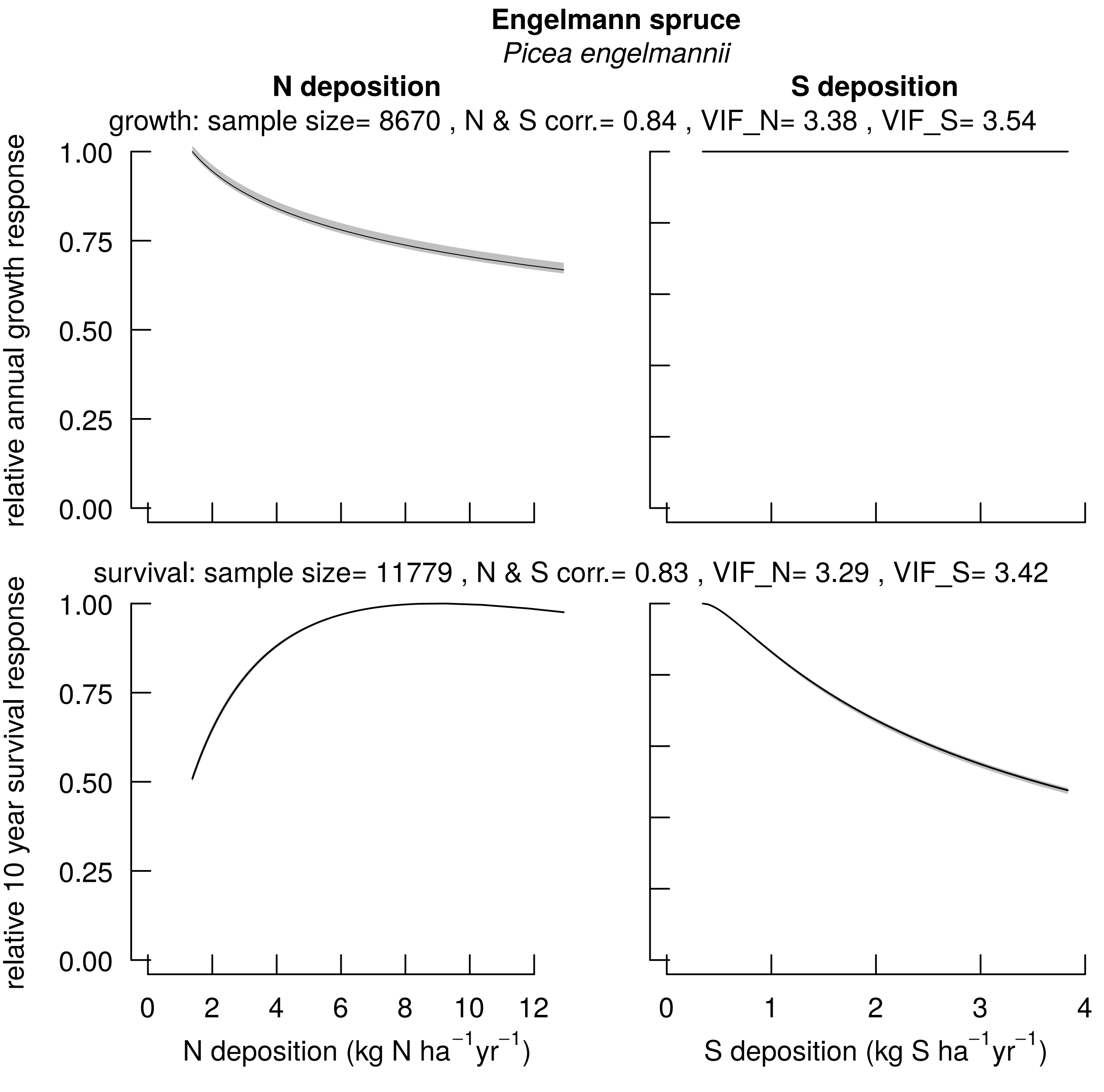

Engelmann spruce
Picea engelmannii
N deposition
S deposition
growth: sample size= 8670 , N & S corr.= 0.84 , VIF_N= 3.38 , VIF_S= 3.54
relative annual growth response
1.00
0.75
0.50
0.25
0.00
survival: sample size= 11779 , N & S corr.= 0.83 , VIF_N= 3.29 , VIF_S= 3.42
relative 10 year survival response
0 2 4 6 8 10 12
0 1 2 3 4
N deposition (kg N ha−1yr−1)
S deposition (kg S ha−1yr−1)

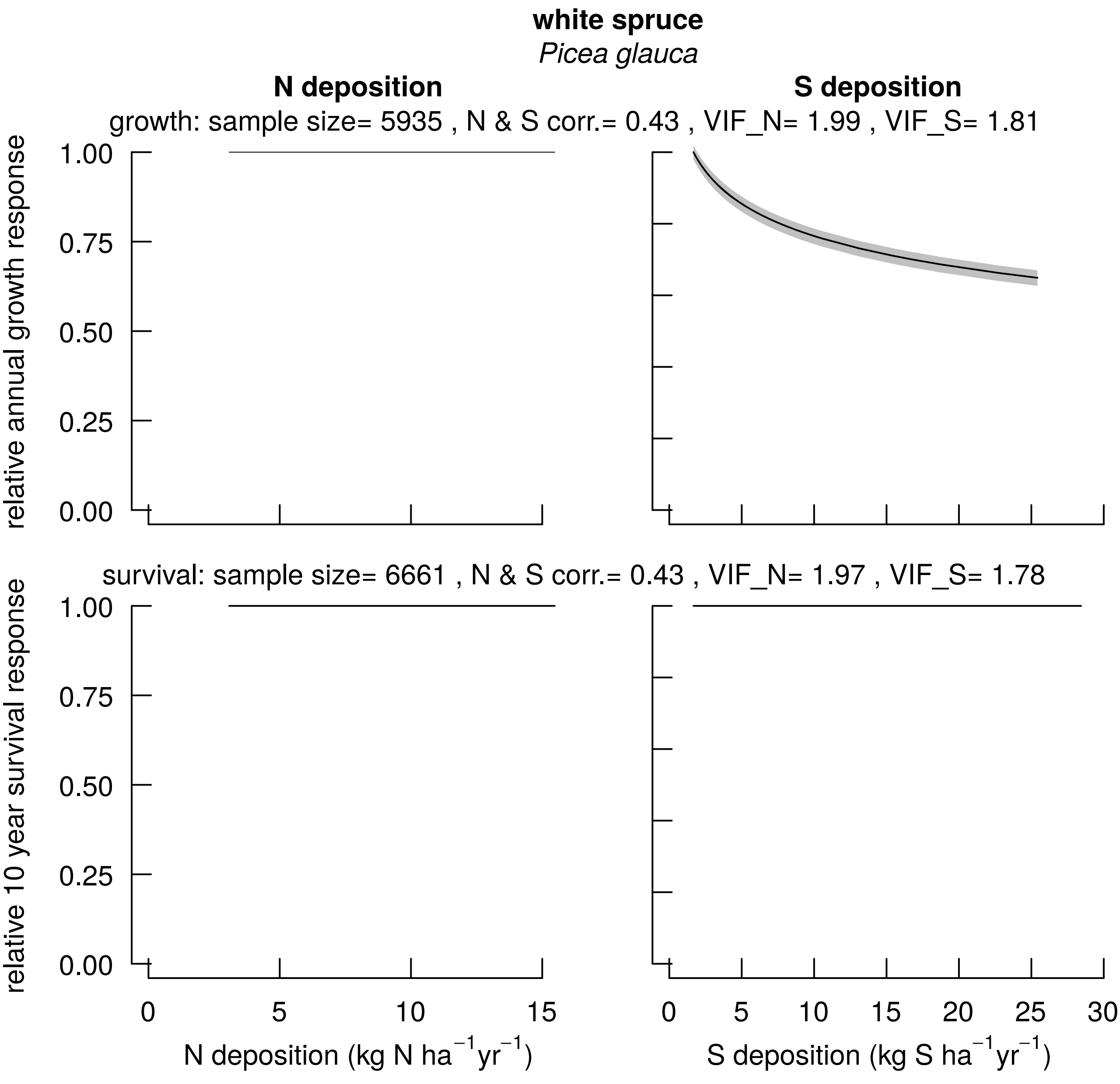

white spruce
Picea glauca
N deposition
S deposition
growth: sample size= 5935 , N & S corr.= 0.43 , VIF_N= 1.99 , VIF_S= 1.81
relative annual growth response
1.00
0.75
0.50
0.25
0.00
survival: sample size= 6661 , N & S corr.= 0.43 , VIF_N= 1.97 , VIF_S= 1.78
relative 10 year survival response
1.00
0.75
0.50
0.25
0.00
0
5
10
15
0
5
10
15
20
25
30
N deposition (kg N ha^{-1}yr^{-1})
S deposition (kg S ha^{-1}yr^{-1})

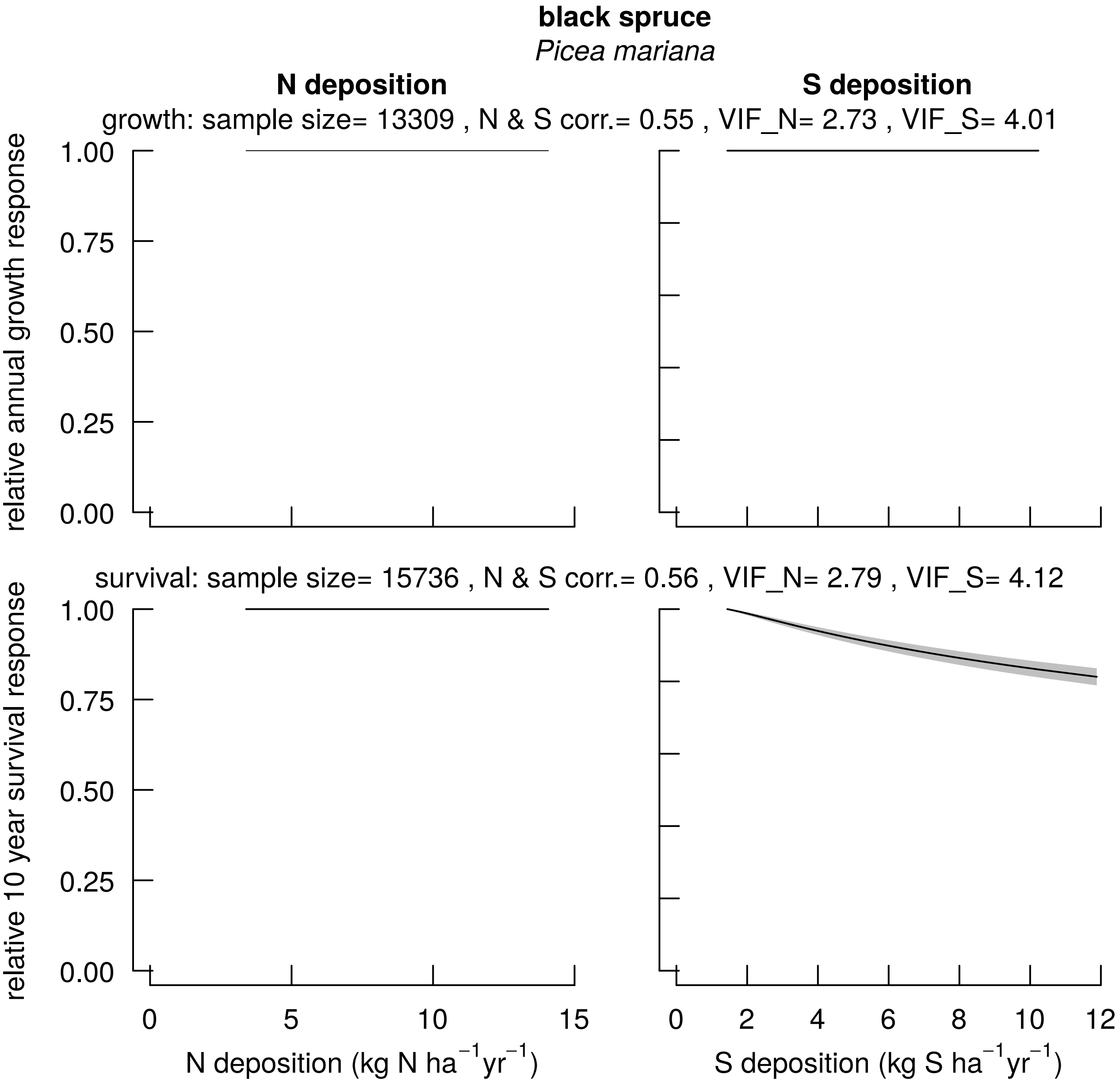

black spruce
Picea mariana
N deposition
S deposition
growth: sample size= 13309 , N & S corr.= 0.55 , VIF_N= 2.73 , VIF_S= 4.01
relative annual growth response
1.00
0.75
0.50
0.25
0.00
survival: sample size= 15736 , N & S corr.= 0.56 , VIF_N= 2.79 , VIF_S= 4.12
relative 10 year survival response
0
5
10
15
0
2
4
6
8
10
12
N deposition (kg N ha^{-1}yr^{-1})
S deposition (kg S ha^{-1}yr^{-1})

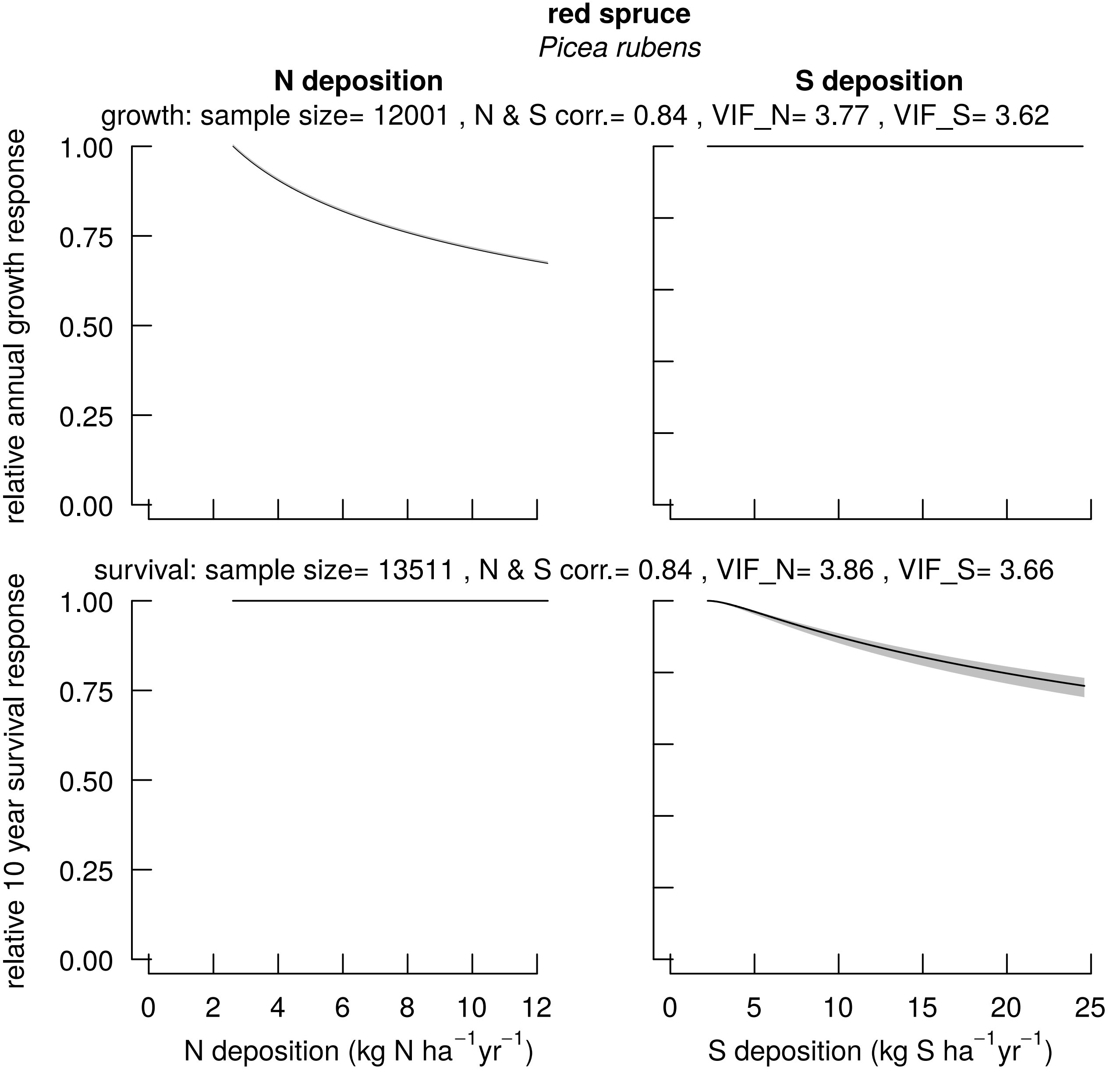
red spruce
Picea rubens
N deposition
S deposition
growth: sample size= 12001 , N & S corr.= 0.84 , VIF_N= 3.77 , VIF_S= 3.62
survival: sample size= 13511 , N & S corr.= 0.84 , VIF_N= 3.86 , VIF_S= 3.66
relative annual growth response
relative 10 year survival response
1.00
0.75
0.50
0.25
0.00
0
2
4
6
8
10
12
5
15
20
25
N deposition (kg N $ha^{-1}yr^{-1}$)
S deposition (kg S $ha^{-1}yr^{-1}$)

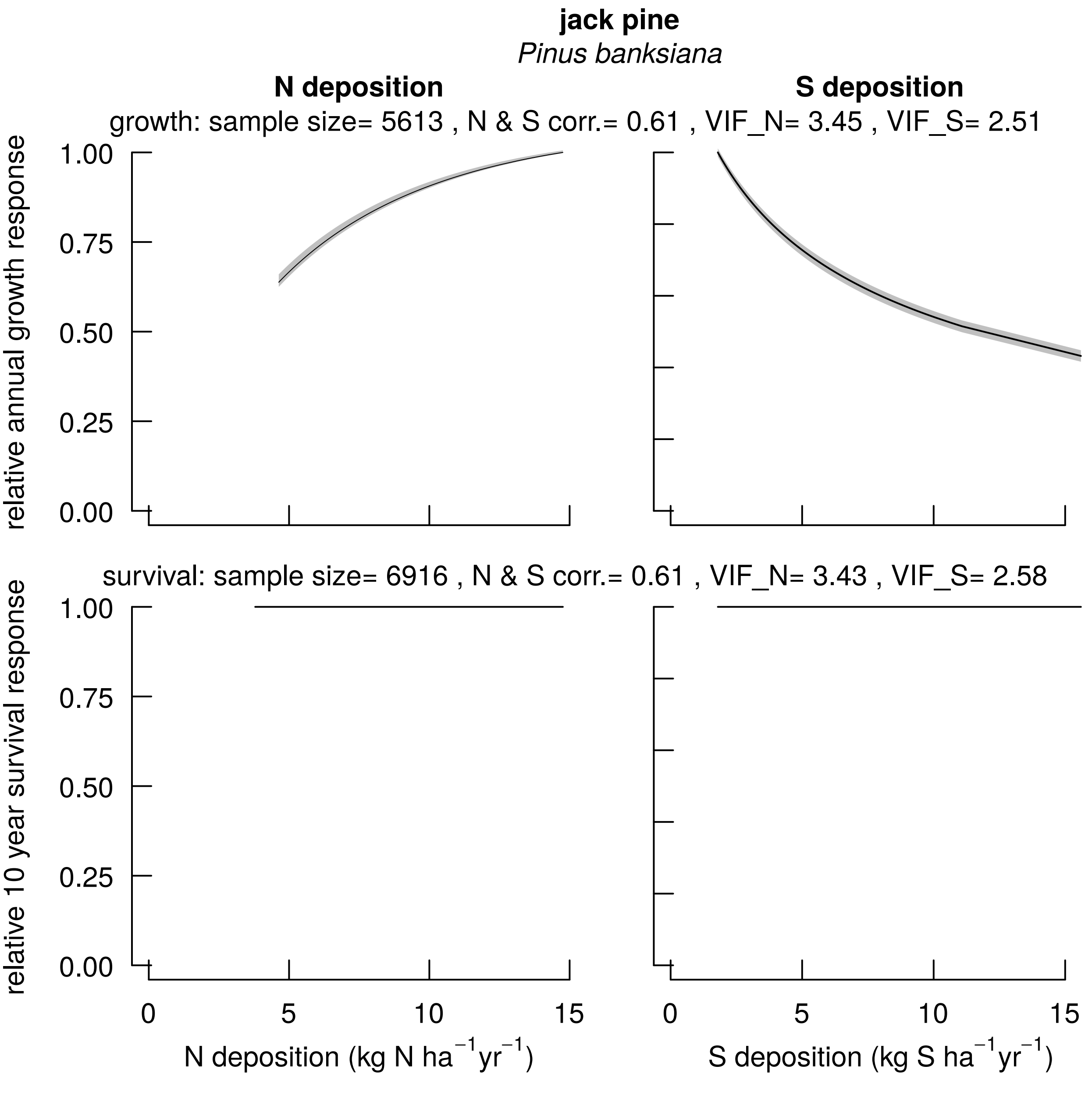
jack pine
Pinus banksiana
N deposition
S deposition
growth: sample size= 5613 , N & S corr.= 0.61 , VIF_N= 3.45 , VIF_S= 2.51
survival: sample size= 6916 , N & S corr.= 0.61 , VIF_N= 3.43 , VIF_S= 2.58
relative annual growth response
relative 10 year survival response
1.00
0.75
0.50
0.25
0.00
0
5
10
15
N deposition (kg N ha⁻¹yr⁻¹)
S deposition (kg S ha⁻¹yr⁻¹)

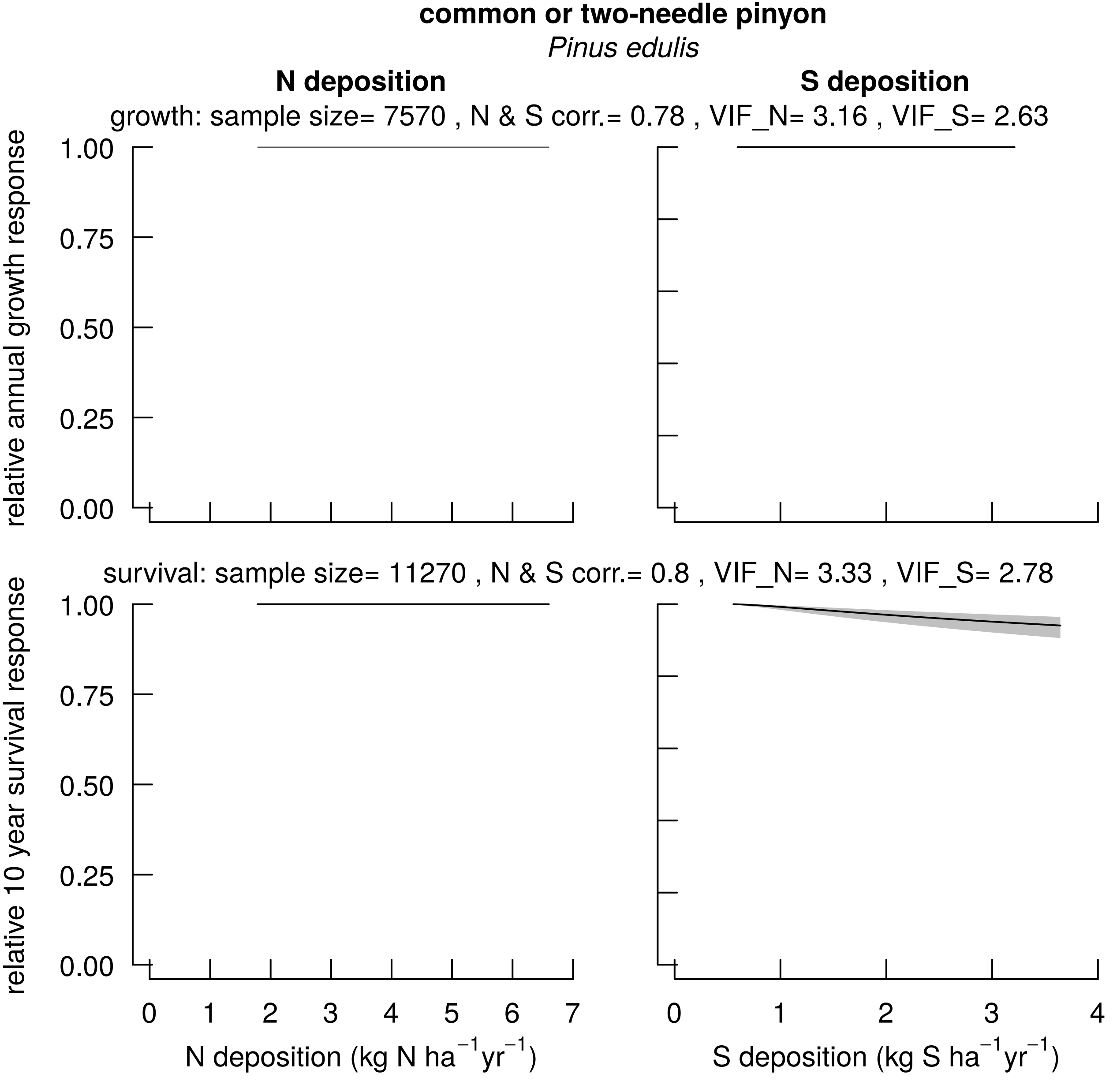

common or two-needle pinyon
Pinus edulis
N deposition
S deposition
growth: sample size= 7570 , N & S corr.= 0.78 , VIF_N= 3.16 , VIF_S= 2.63
relative annual growth response
1.00
0.75
0.50
0.25
0.00
survival: sample size= 11270 , N & S corr.= 0.8 , VIF_N= 3.33 , VIF_S= 2.78
relative 10 year survival response
1.00
0.75
0.50
0.25
0.00
0 1 2 3 4 5 6 7
N deposition (kg N ha^-1 yr^-1)
0 1 2 3 4
S deposition (kg S ha^-1 yr^-1)

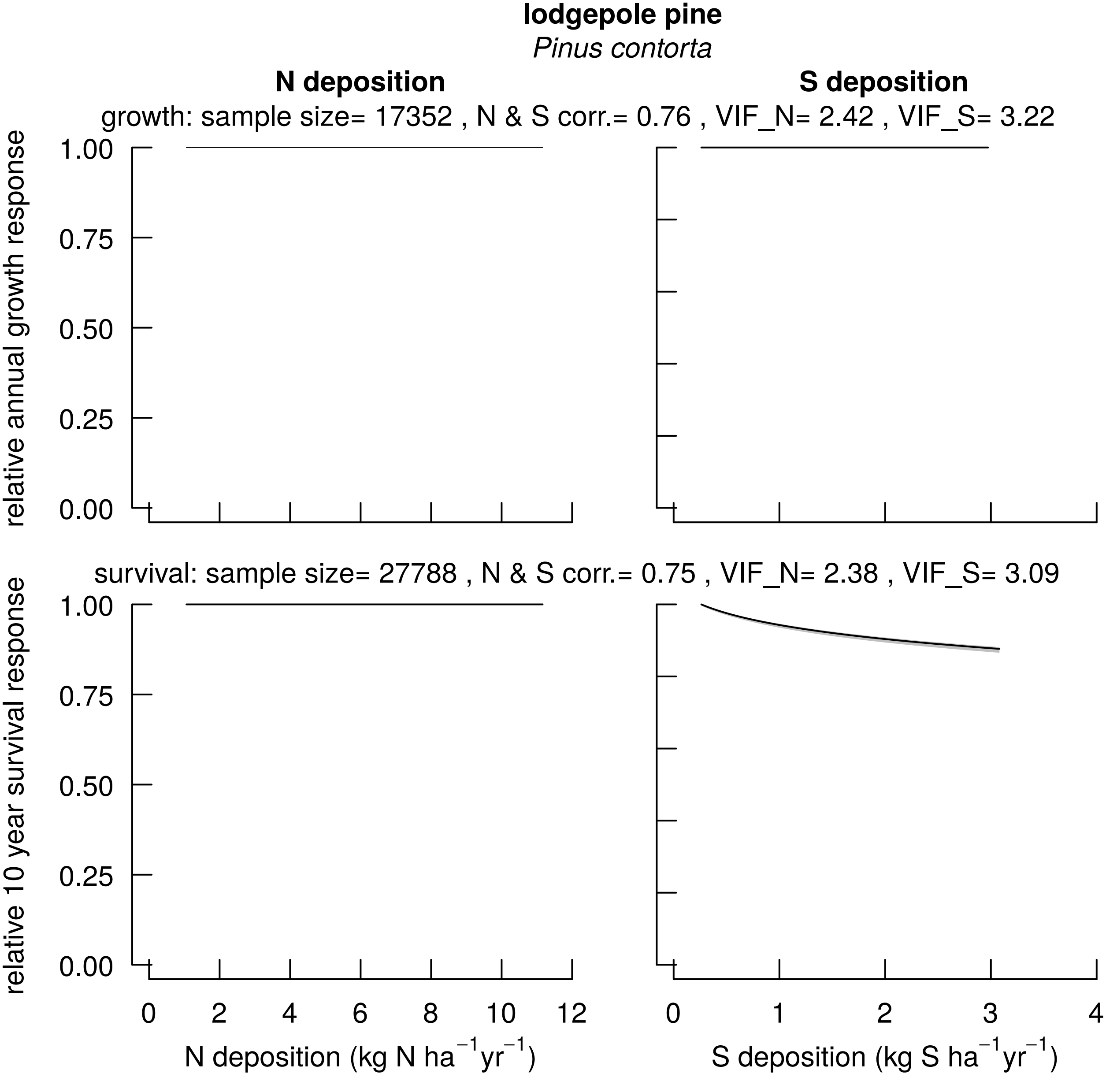

lodgepole pine
Pinus contorta
N deposition
S deposition
growth: sample size= 17352 , N & S corr.= 0.76 , VIF_N= 2.42 , VIF_S= 3.22
relative annual growth response
1.00
0.75
0.50
0.25
0.00
survival: sample size= 27788 , N & S corr.= 0.75 , VIF_N= 2.38 , VIF_S= 3.09
relative 10 year survival response
1.00
0.75
0.50
0.25
0.00
0 2 4 6 8 10 12
0 1 2 3 4
N deposition (kg N ha^{-1}yr^{-1})
S deposition (kg S ha^{-1}yr^{-1})

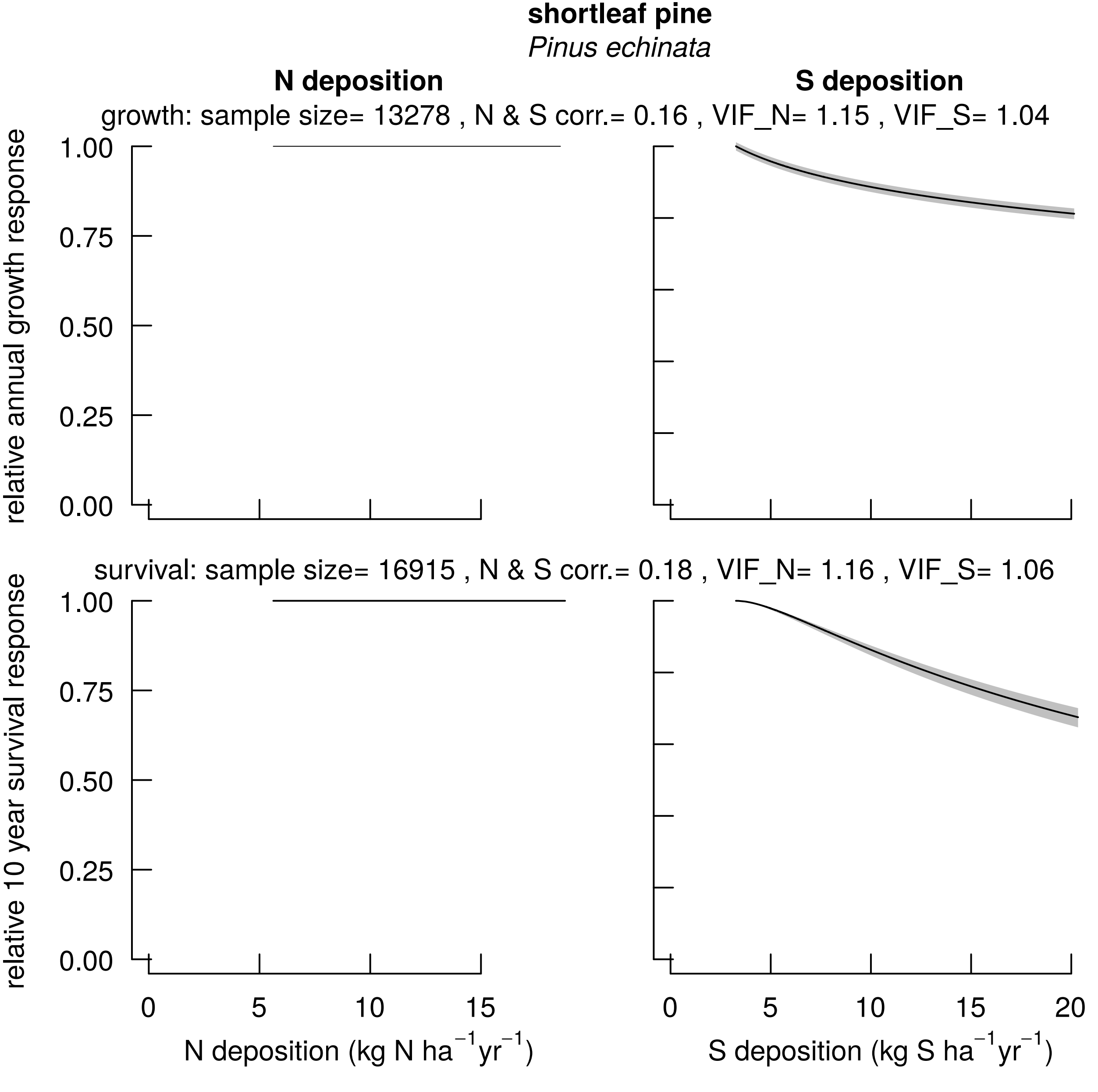

shortleaf pine
Pinus echinata
N deposition
S deposition
growth: sample size= 13278 , N & S corr.= 0.16 , VIF_N= 1.15 , VIF_S= 1.04
relative annual growth response
1.00
0.75
0.50
0.25
0.00
survival: sample size= 16915 , N & S corr.= 0.18 , VIF_N= 1.16 , VIF_S= 1.06
relative 10 year survival response
1.00
0.75
0.50
0.25
0.00
0
5
10
15
0
5
10
15
20
N deposition (kg N ha^{-1}yr^{-1})
S deposition (kg S ha^{-1}yr^{-1})

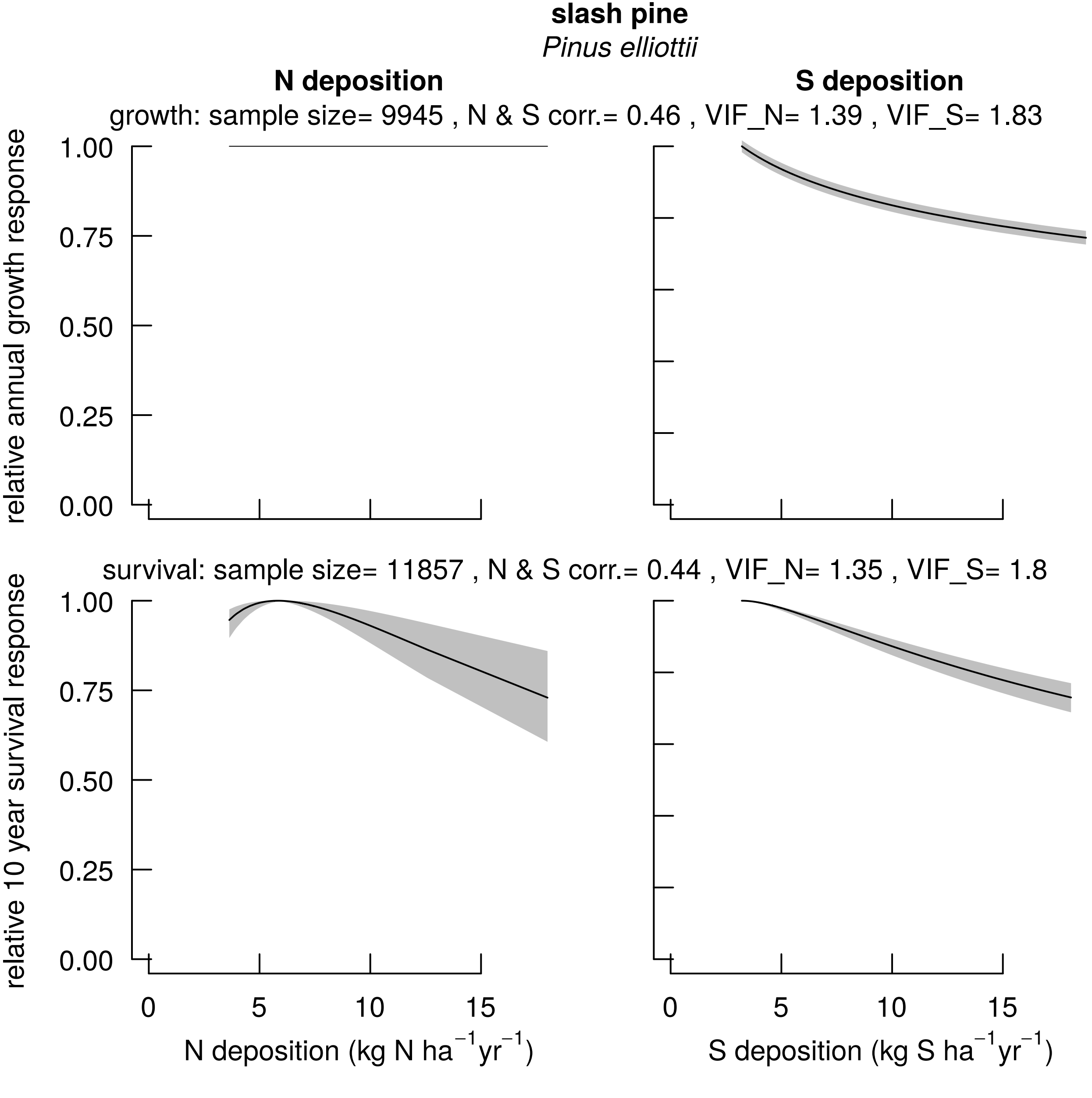
slash pine
Pinus elliottii
N deposition
S deposition
growth: sample size= 9945 , N & S corr.= 0.46 , VIF_N= 1.39 , VIF_S= 1.83
relative annual growth response
1.00
0.75
0.50
0.25
0.00
survival: sample size= 11857 , N & S corr.= 0.44 , VIF_N= 1.35 , VIF_S= 1.8
relative 10 year survival response
0
5
10
15
N deposition (kg N ha⁻¹yr⁻¹)
S deposition (kg S ha⁻¹yr⁻¹)

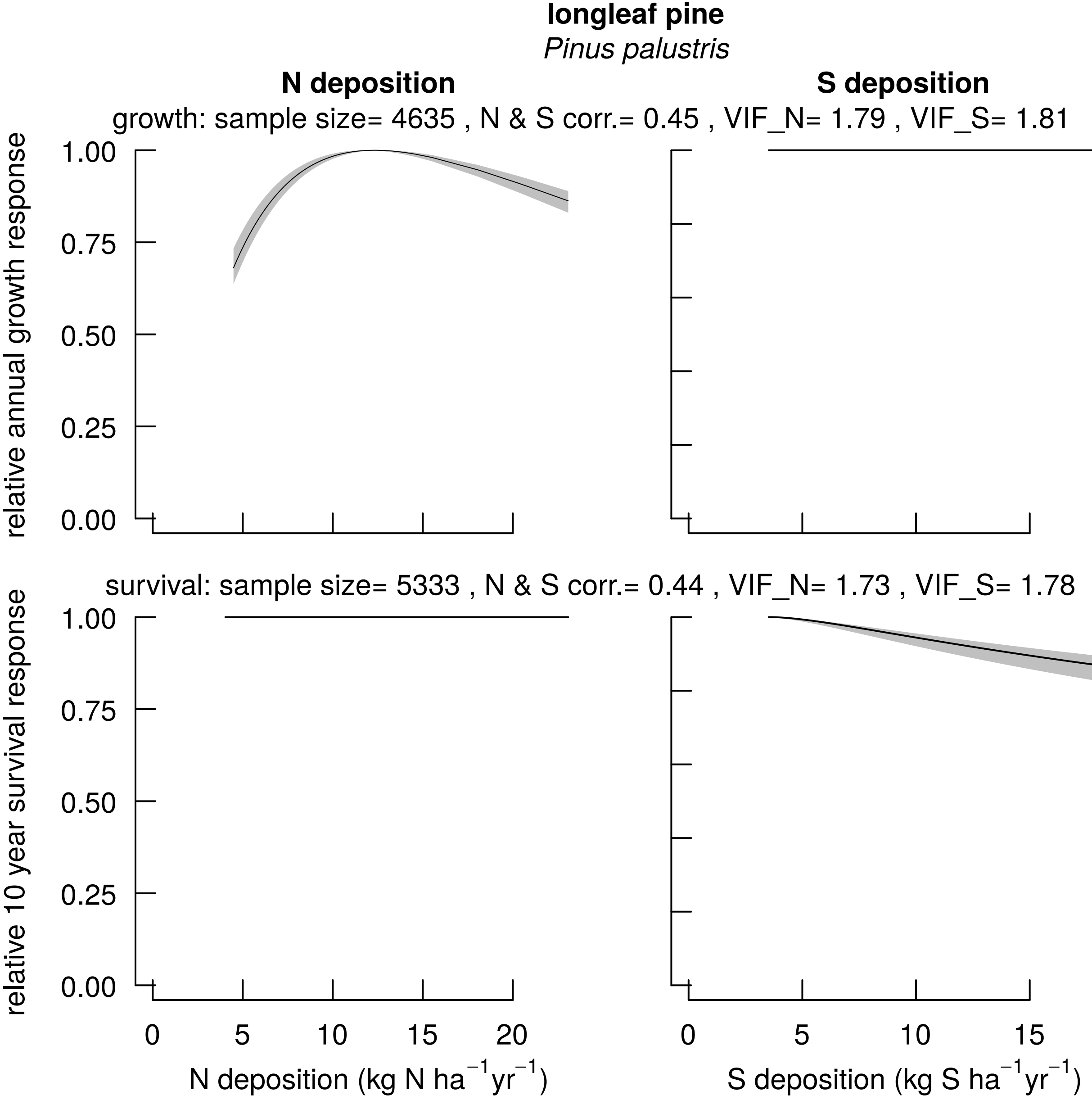

longleaf pine
Pinus palustris
N deposition
S deposition
growth: sample size= 4635 , N & S corr.= 0.45 , VIF_N= 1.79 , VIF_S= 1.81
relative annual growth response
1.00
0.75
0.50
0.25
0.00
survival: sample size= 5333 , N & S corr.= 0.44 , VIF_N= 1.73 , VIF_S= 1.78
relative 10 year survival response
0 5 10 15 20
0 5 10 15
N deposition (kg N ha^{-1}yr^{-1})
S deposition (kg S ha^{-1}yr^{-1})

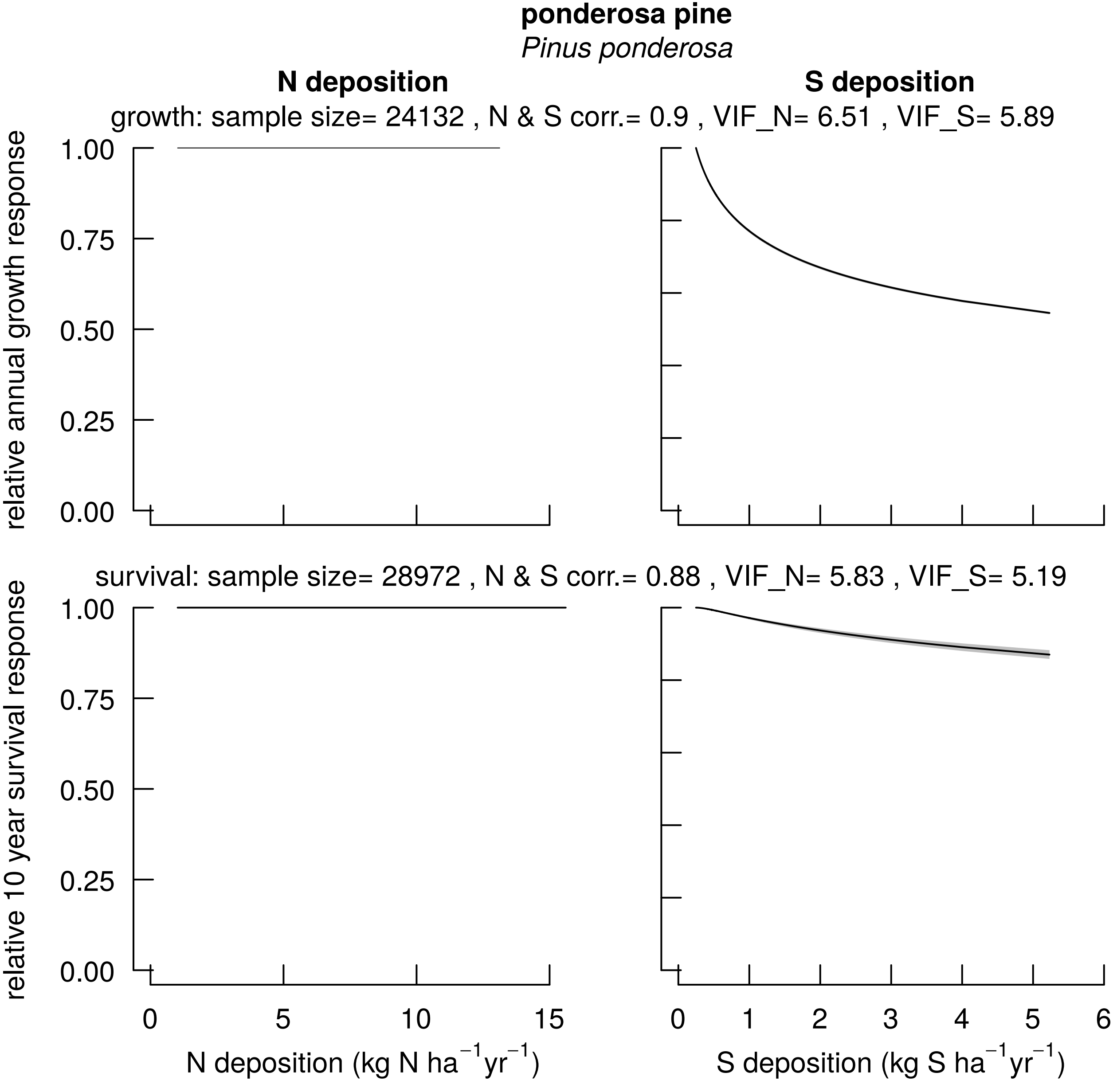

ponderosa pine
Pinus ponderosa
N deposition
S deposition
growth: sample size= 24132 , N & S corr.= 0.9 , VIF_N= 6.51 , VIF_S= 5.89
relative annual growth response
1.00
0.75
0.50
0.25
0.00
survival: sample size= 28972 , N & S corr.= 0.88 , VIF_N= 5.83 , VIF_S= 5.19
relative 10 year survival response
1.00
0.75
0.50
0.25
0.00
0
5
10
15
0
1
2
3
4
5
6
N deposition (kg N ha^{-1}yr^{-1})
S deposition (kg S ha^{-1}yr^{-1})

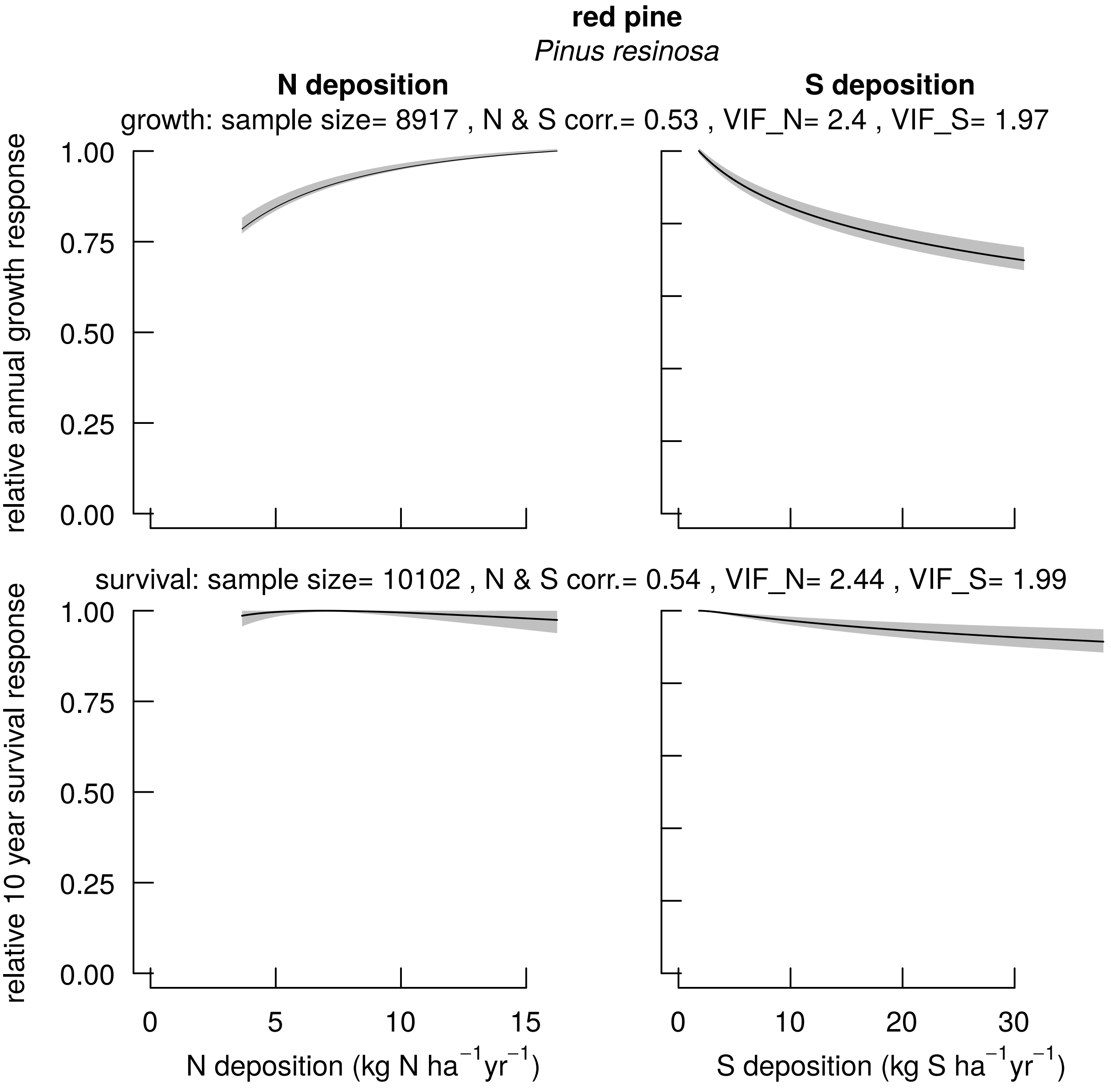
red pine
Pinus resinosa
N deposition
S deposition
growth: sample size= 8917 , N & S corr.= 0.53 , VIF_N= 2.4 , VIF_S= 1.97
relative annual growth response
1.00
0.75
0.50
0.25
0.00
survival: sample size= 10102 , N & S corr.= 0.54 , VIF_N= 2.44 , VIF_S= 1.99
relative 10 year survival response
1.00
0.75
0.50
0.25
0.00
0
5
10
15
0
10
20
30
N deposition (kg N ha^{-1}yr^{-1})
S deposition (kg S ha^{-1}yr^{-1})

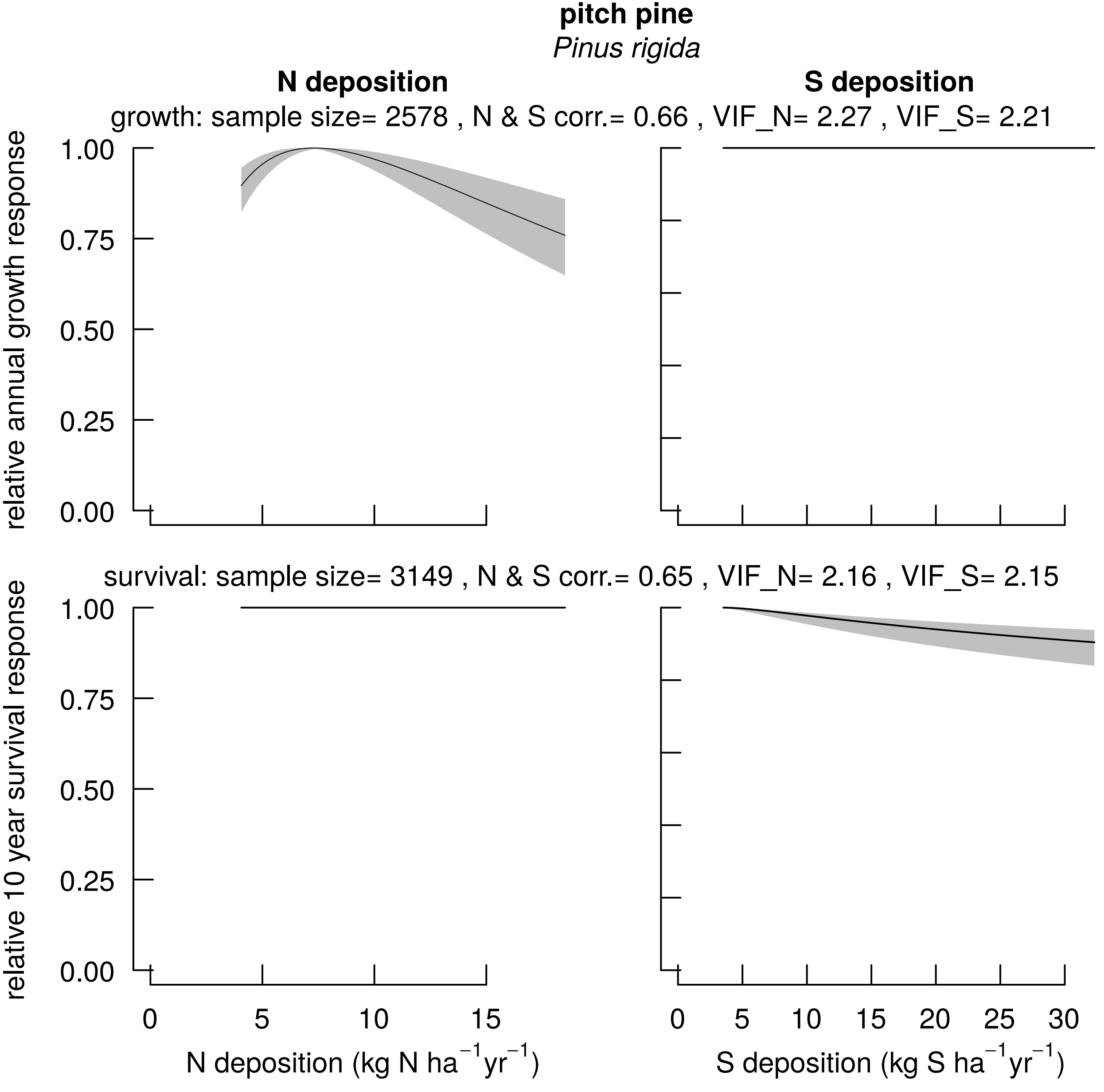
pitch pine
Pinus rigida
N deposition
S deposition
growth: sample size= 2578 , N & S corr.= 0.66 , VIF_N= 2.27 , VIF_S= 2.21
relative annual growth response
1.00
0.75
0.50
0.25
0.00
survival: sample size= 3149 , N & S corr.= 0.65 , VIF_N= 2.16 , VIF_S= 2.15
relative 10 year survival response
1.00
0.75
0.50
0.25
0.00
0
5
10
15
N deposition (kg N ha^{-1}yr^{-1})
0
5
10
15
20
25
30
S deposition (kg S ha^{-1}yr^{-1})

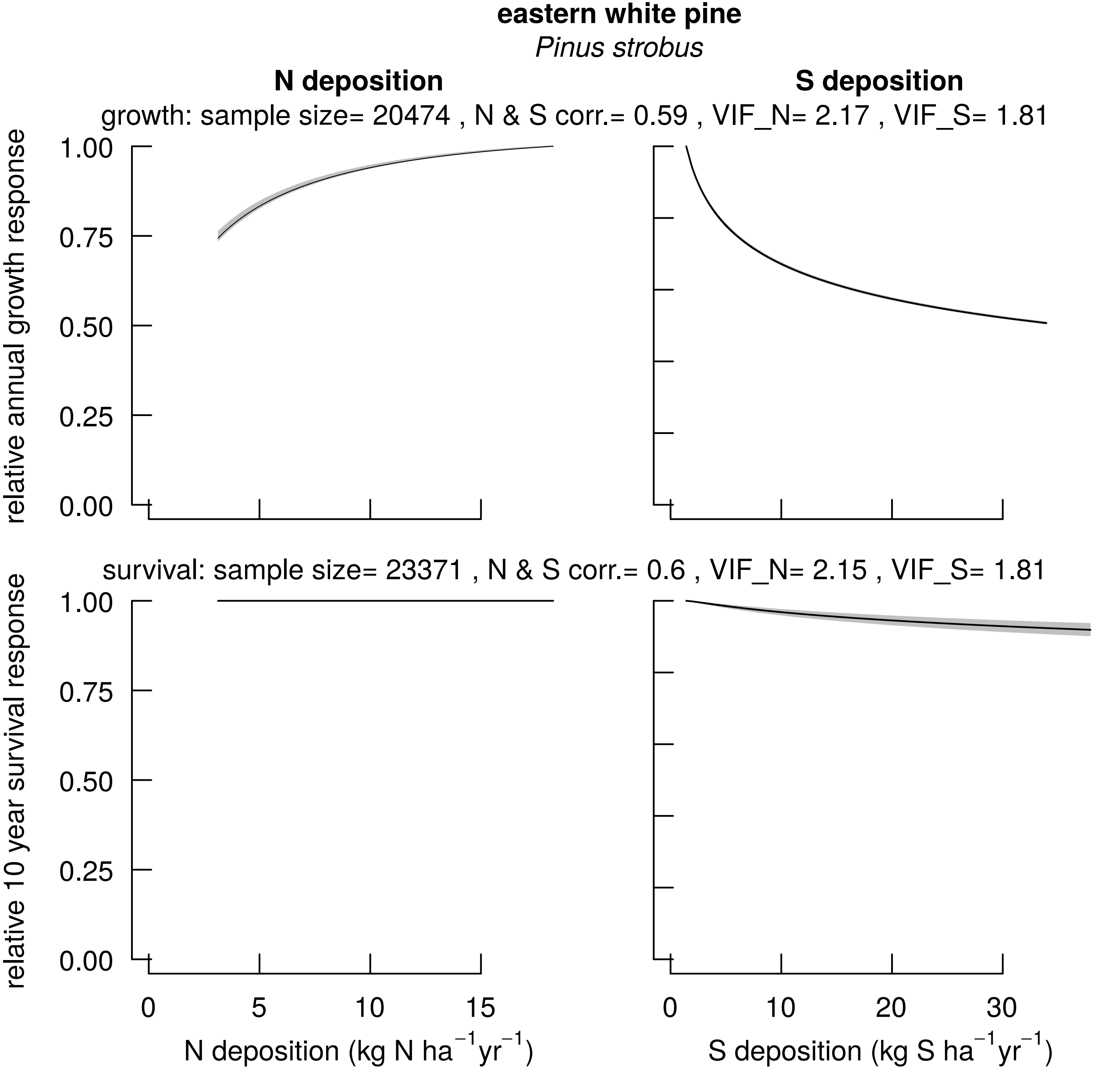
eastern white pine
Pinus strobus
N deposition
S deposition
growth: sample size= 20474 , N & S corr.= 0.59 , VIF_N= 2.17 , VIF_S= 1.81
survival: sample size= 23371 , N & S corr.= 0.6 , VIF_N= 2.15 , VIF_S= 1.81
relative annual growth response
relative 10 year survival response
1.00
0.75
0.50
0.25
0.00
0
5
10
15
20
30
N deposition (kg N ha^{-1}yr^{-1})
S deposition (kg S ha^{-1}yr^{-1})

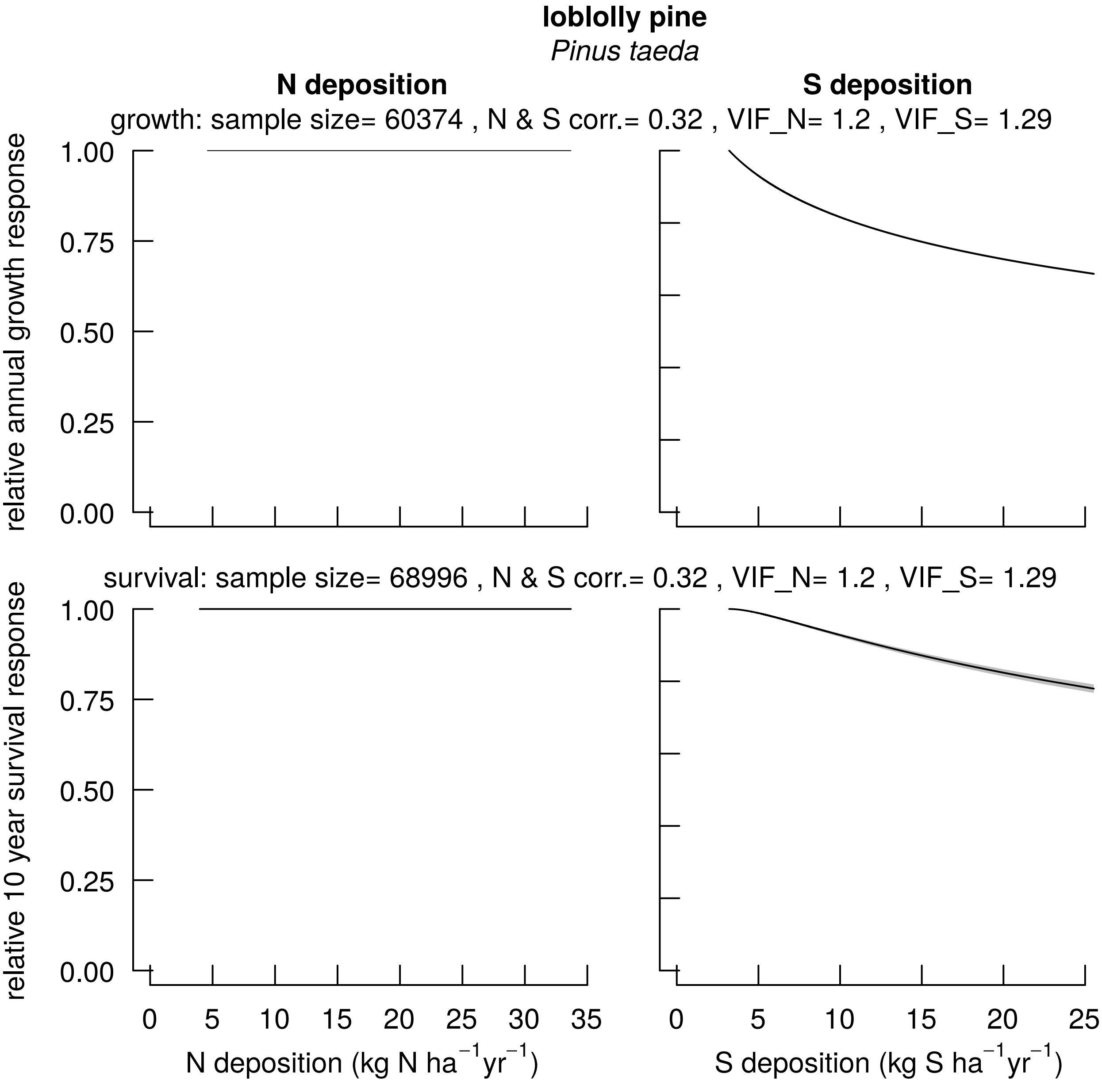

loblolly pine
Pinus taeda
N deposition
S deposition
growth: sample size= 60374 , N & S corr.= 0.32 , VIF_N= 1.2 , VIF_S= 1.29
survival: sample size= 68996 , N & S corr.= 0.32 , VIF_N= 1.2 , VIF_S= 1.29
relative annual growth response
relative 10 year survival response
1.00
0.75
0.50
0.25
0.00
0 5 10 15 20 25 30 35
0 5 10 15 20 25
N deposition (kg N ha−1yr−1)
S deposition (kg S ha−1yr−1)

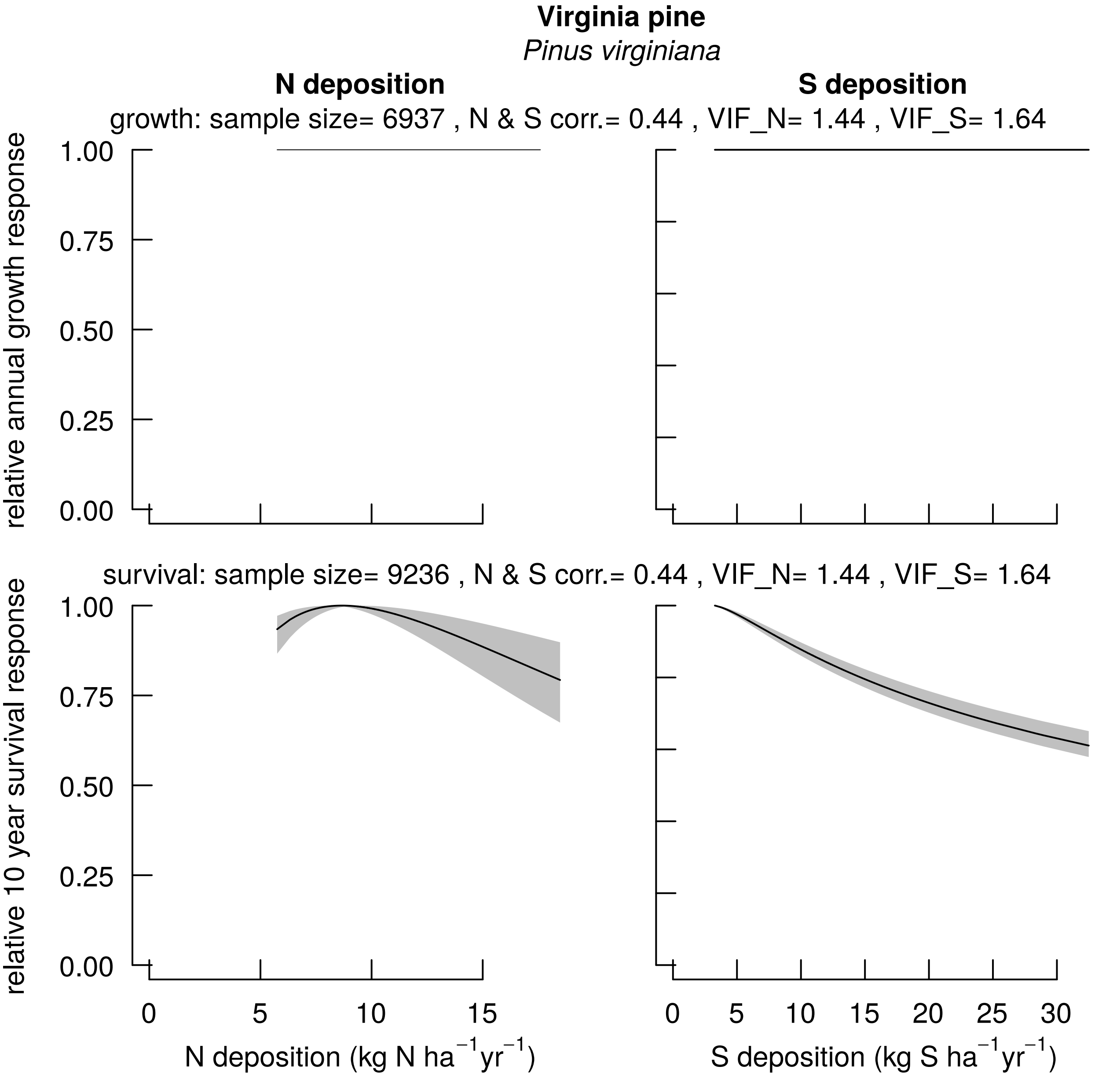

Virginia pine
Pinus virginiana
N deposition
S deposition
growth: sample size= 6937 , N & S corr.= 0.44 , VIF_N= 1.44 , VIF_S= 1.64
relative annual growth response
1.00
0.75
0.50
0.25
0.00
survival: sample size= 9236 , N & S corr.= 0.44 , VIF_N= 1.44 , VIF_S= 1.64
relative 10 year survival response
1.00
0.75
0.50
0.25
0.00
0
5
10
15
N deposition (kg N ha−1yr−1)
0
5
10
15
20
25
30
S deposition (kg S ha−1yr−1)

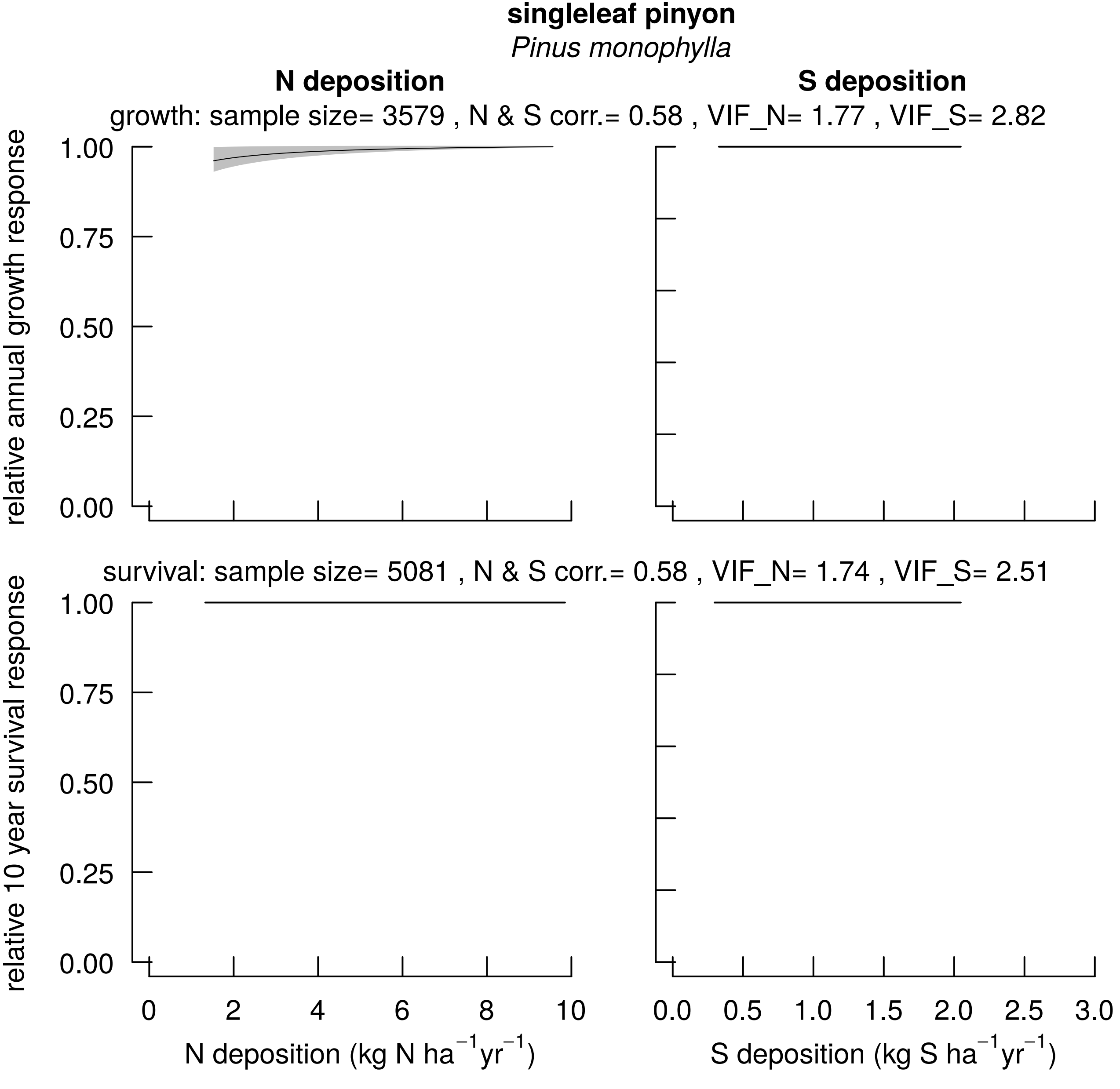

singleleaf pinyon
Pinus monophylla
N deposition
S deposition
growth: sample size= 3579 , N & S corr.= 0.58 , VIF_N= 1.77 , VIF_S= 2.82
relative annual growth response
1.00
0.75
0.50
0.25
0.00
survival: sample size= 5081 , N & S corr.= 0.58 , VIF_N= 1.74 , VIF_S= 2.51
relative 10 year survival response
0 2 4 6 8 10
0.0 0.5 1.0 1.5 2.0 2.5 3.0
N deposition (kg N ha^{-1}yr^{-1})
S deposition (kg S ha^{-1}yr^{-1})

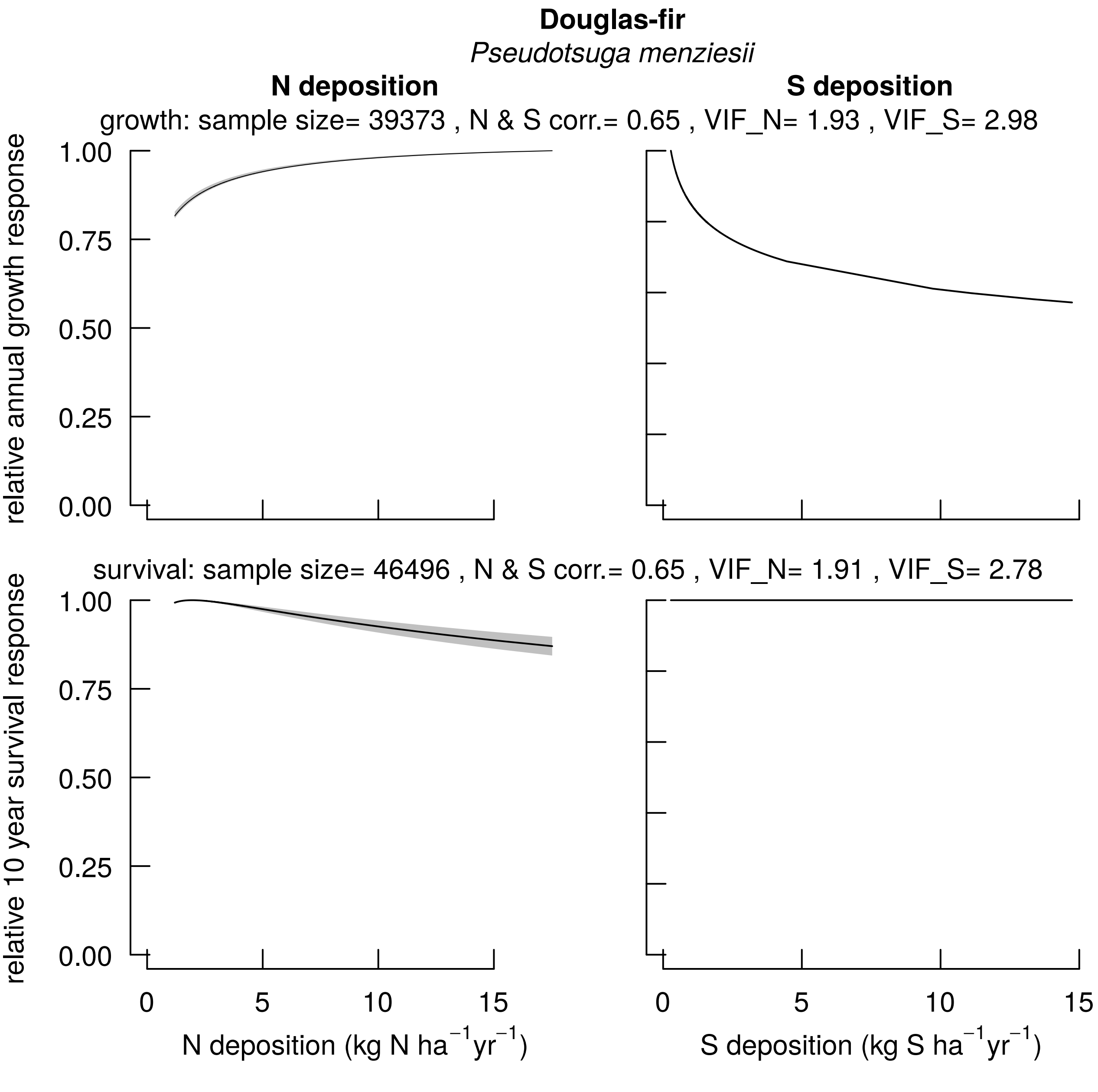

Douglas-fir
Pseudotsuga menziesii
N deposition
S deposition
growth: sample size= 39373 , N & S corr.= 0.65 , VIF_N= 1.93 , VIF_S= 2.98
survival: sample size= 46496 , N & S corr.= 0.65 , VIF_N= 1.91 , VIF_S= 2.78
relative annual growth response
relative 10 year survival response
1.00
0.75
0.50
0.25
0.00
0
5
10
15
N deposition (kg N ha^{-1}yr^{-1})
S deposition (kg S ha^{-1}yr^{-1})

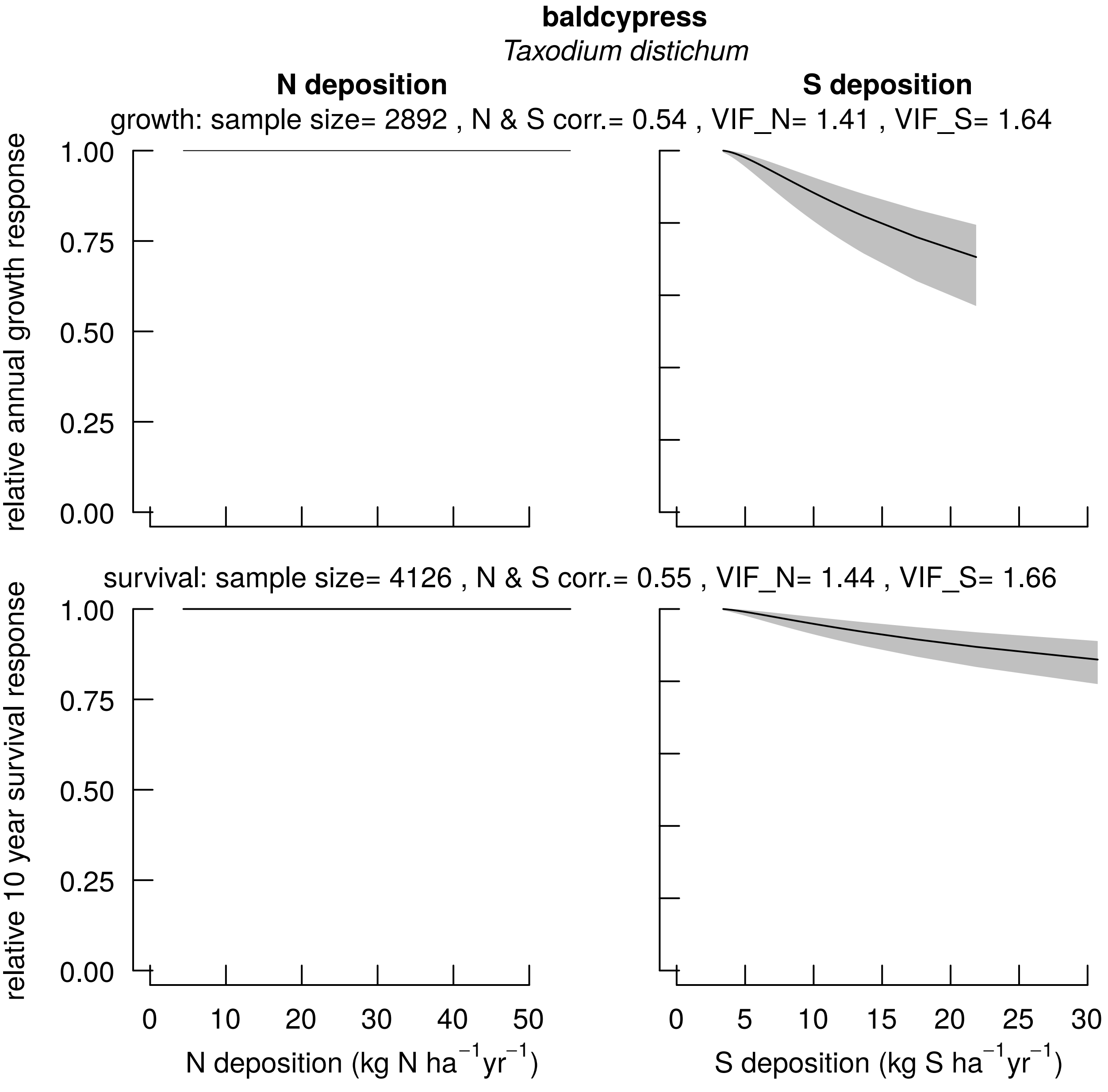
baldcypress
Taxodium distichum
N deposition
S deposition
growth: sample size= 2892 , N & S corr.= 0.54 , VIF_N= 1.41 , VIF_S= 1.64
relative annual growth response
survival: sample size= 4126 , N & S corr.= 0.55 , VIF_N= 1.44 , VIF_S= 1.66
relative 10 year survival response
1.00
0.75
0.50
0.25
0.00
0
10
20
30
40
50
0
5
10
15
20
25
30
N deposition (kg N ha−1yr−1)
S deposition (kg S ha−1yr−1)

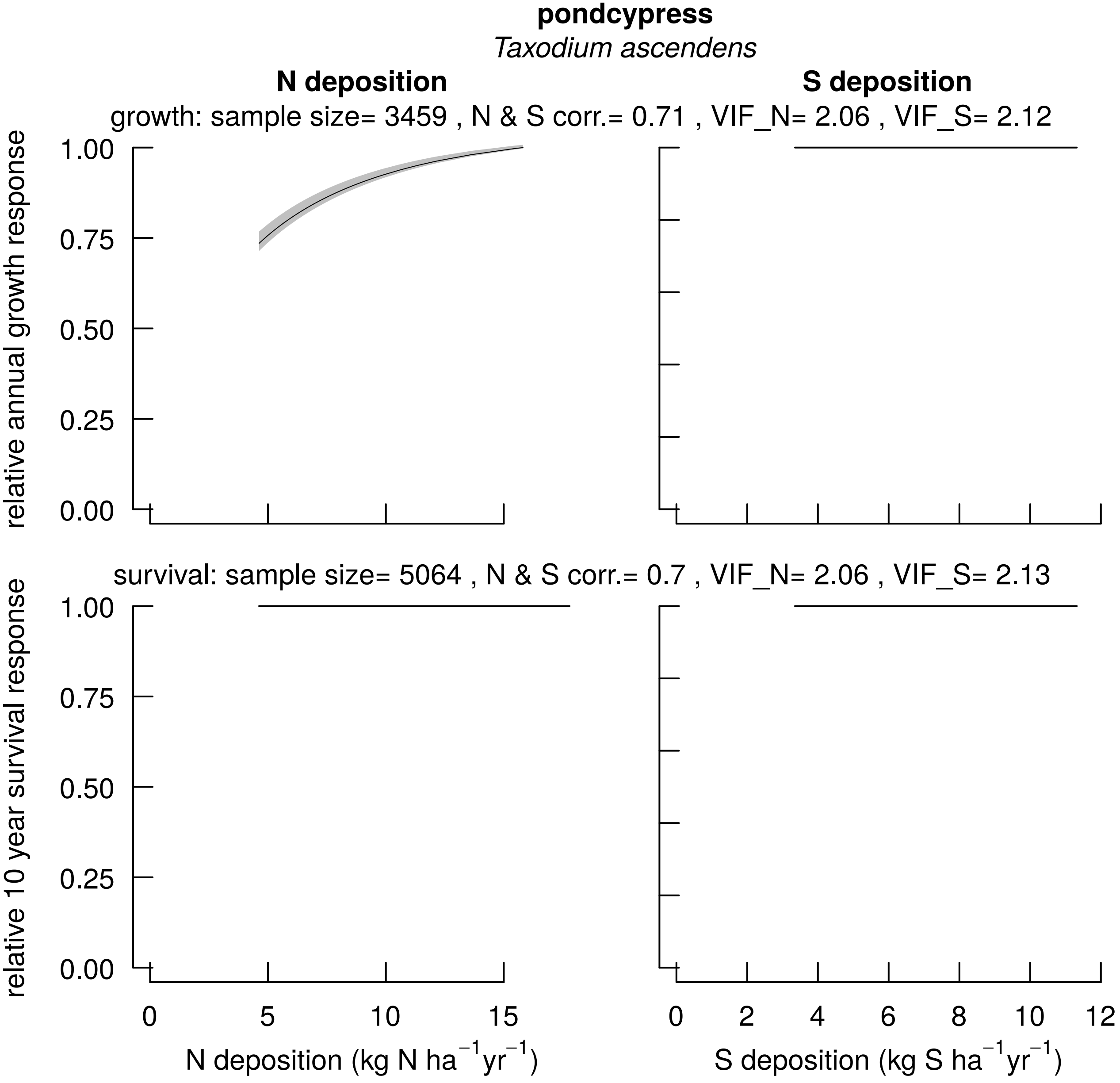
pondcypress
Taxodium ascendens
N deposition
S deposition
growth: sample size= 3459 , N & S corr.= 0.71 , VIF_N= 2.06 , VIF_S= 2.12
survival: sample size= 5064 , N & S corr.= 0.7 , VIF_N= 2.06 , VIF_S= 2.13
relative annual growth response
relative 10 year survival response
1.00
0.75
0.50
0.25
0.00
0
5
10
15
0
2
4
6
8
10
12
N deposition (kg N ha^{-1}yr^{-1})
S deposition (kg S ha^{-1}yr^{-1})

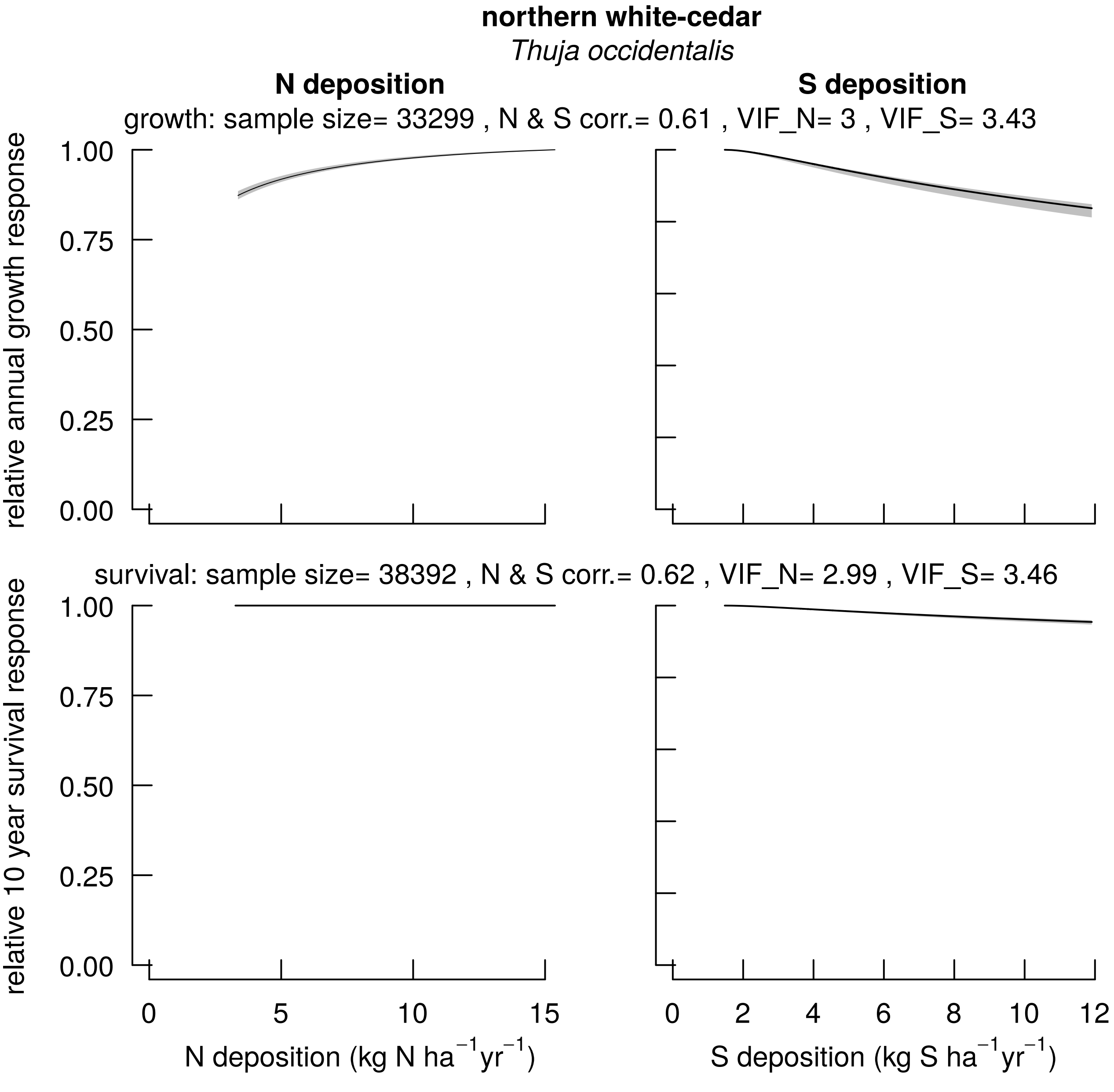
northern white-cedar
Thuja occidentalis
N deposition
S deposition
growth: sample size= 33299 , N & S corr.= 0.61 , VIF_N= 3 , VIF_S= 3.43
relative annual growth response
1.00
0.75
0.50
0.25
0.00
survival: sample size= 38392 , N & S corr.= 0.62 , VIF_N= 2.99 , VIF_S= 3.46
relative 10 year survival response
0
5
10
15
0
2
4
6
8
10
12
N deposition (kg N ha^{-1}yr^{-1})
S deposition (kg S ha^{-1}yr^{-1})

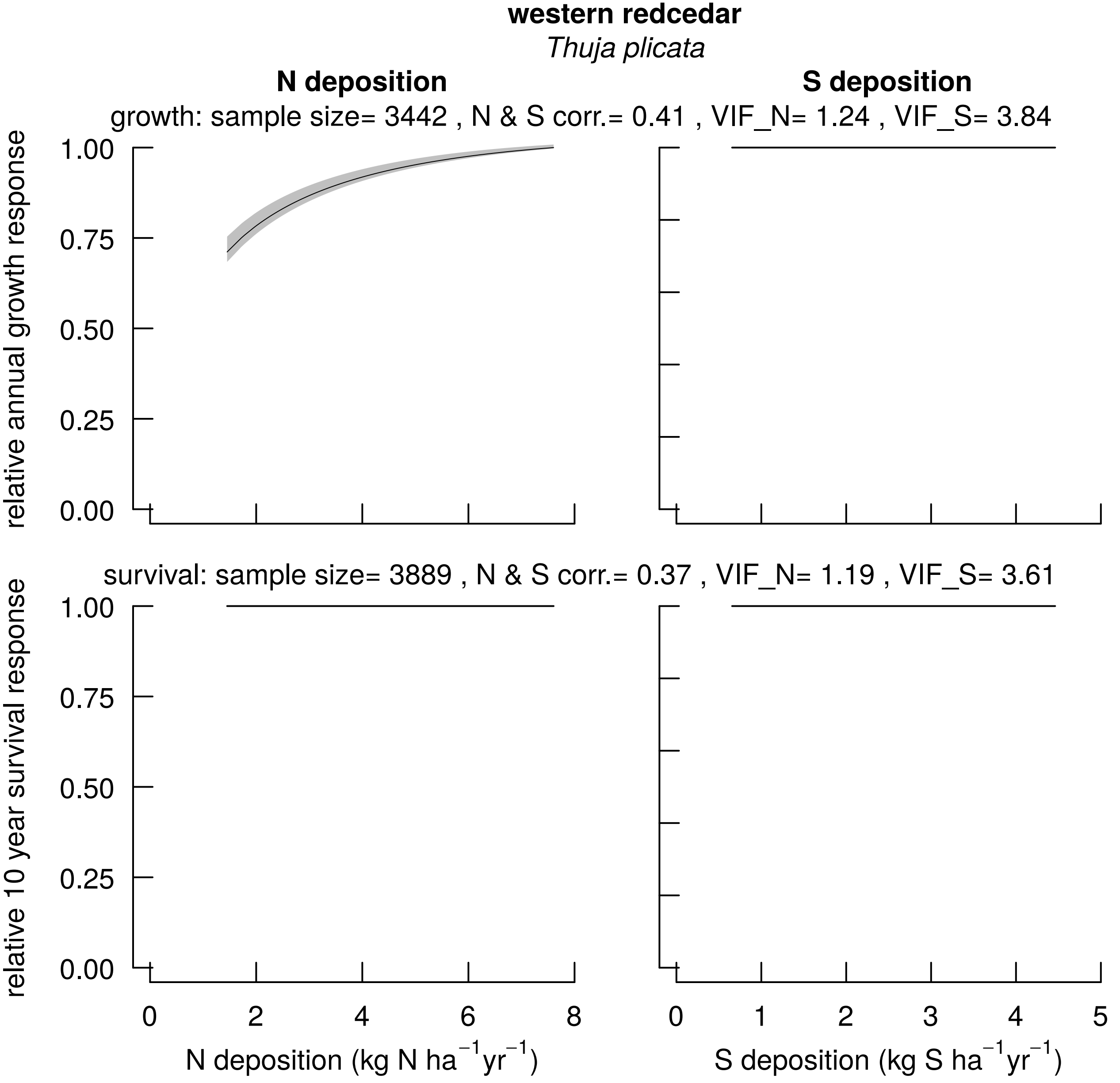
western redcedar
Thuja plicata
N deposition
S deposition
growth: sample size= 3442 , N & S corr.= 0.41 , VIF_N= 1.24 , VIF_S= 3.84
survival: sample size= 3889 , N & S corr.= 0.37 , VIF_N= 1.19 , VIF_S= 3.61
relative annual growth response
relative 10 year survival response
1.00
0.75
0.50
0.25
0.00
0
2
4
6
8
1
3
5
N deposition (kg N ha⁻¹yr⁻¹)
S deposition (kg S ha⁻¹yr⁻¹)

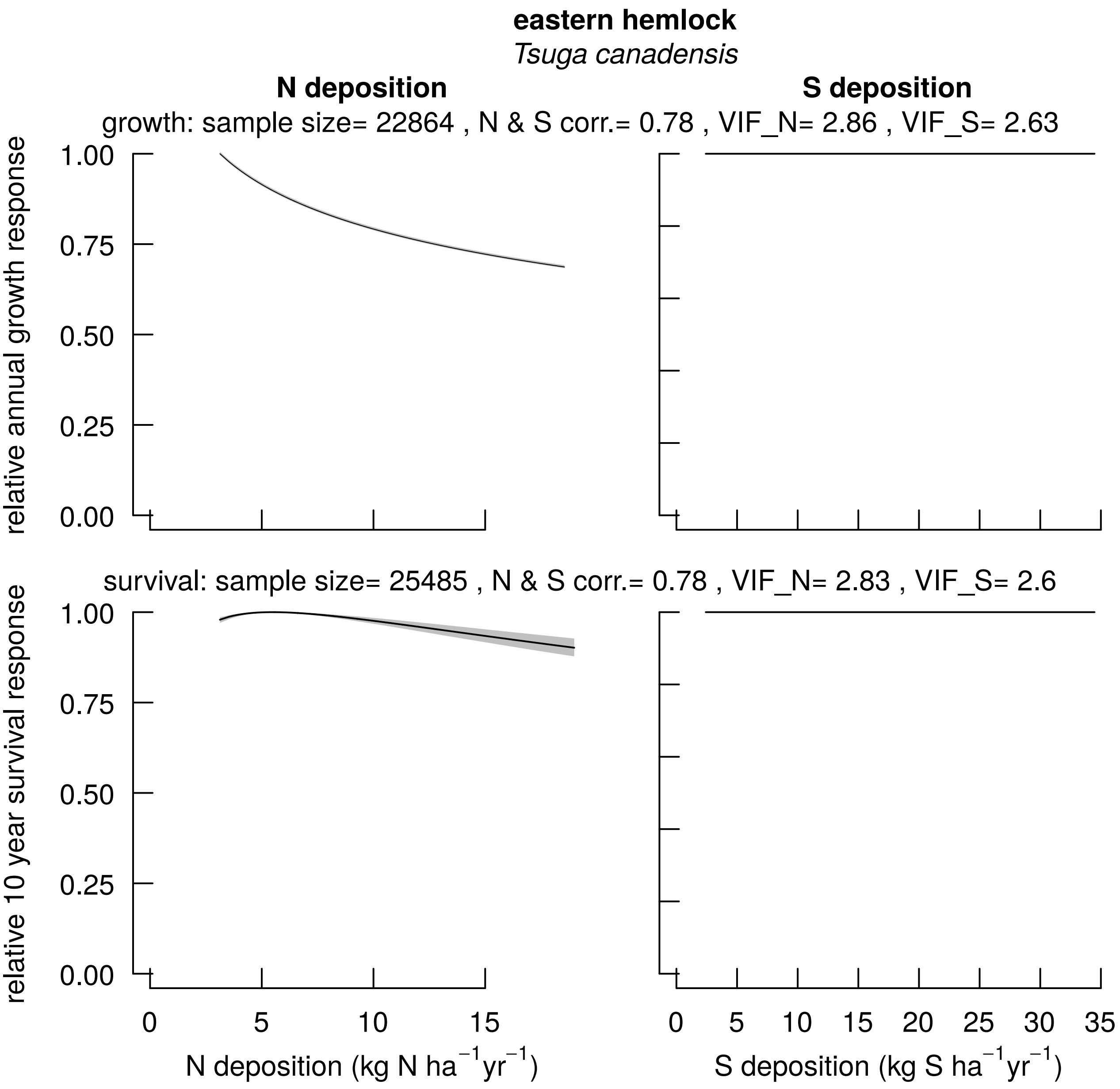

eastern hemlock
Tsuga canadensis
N deposition
S deposition
growth: sample size= 22864 , N & S corr.= 0.78 , VIF_N= 2.86 , VIF_S= 2.63
relative annual growth response
1.00
0.75
0.50
0.25
0.00
survival: sample size= 25485 , N & S corr.= 0.78 , VIF_N= 2.83 , VIF_S= 2.6
relative 10 year survival response
1.00
0.75
0.50
0.25
0.00
0
5
10
15
N deposition (kg N ha−1yr−1)
0
5
10
15
20
25
30
35
S deposition (kg S ha−1yr−1)

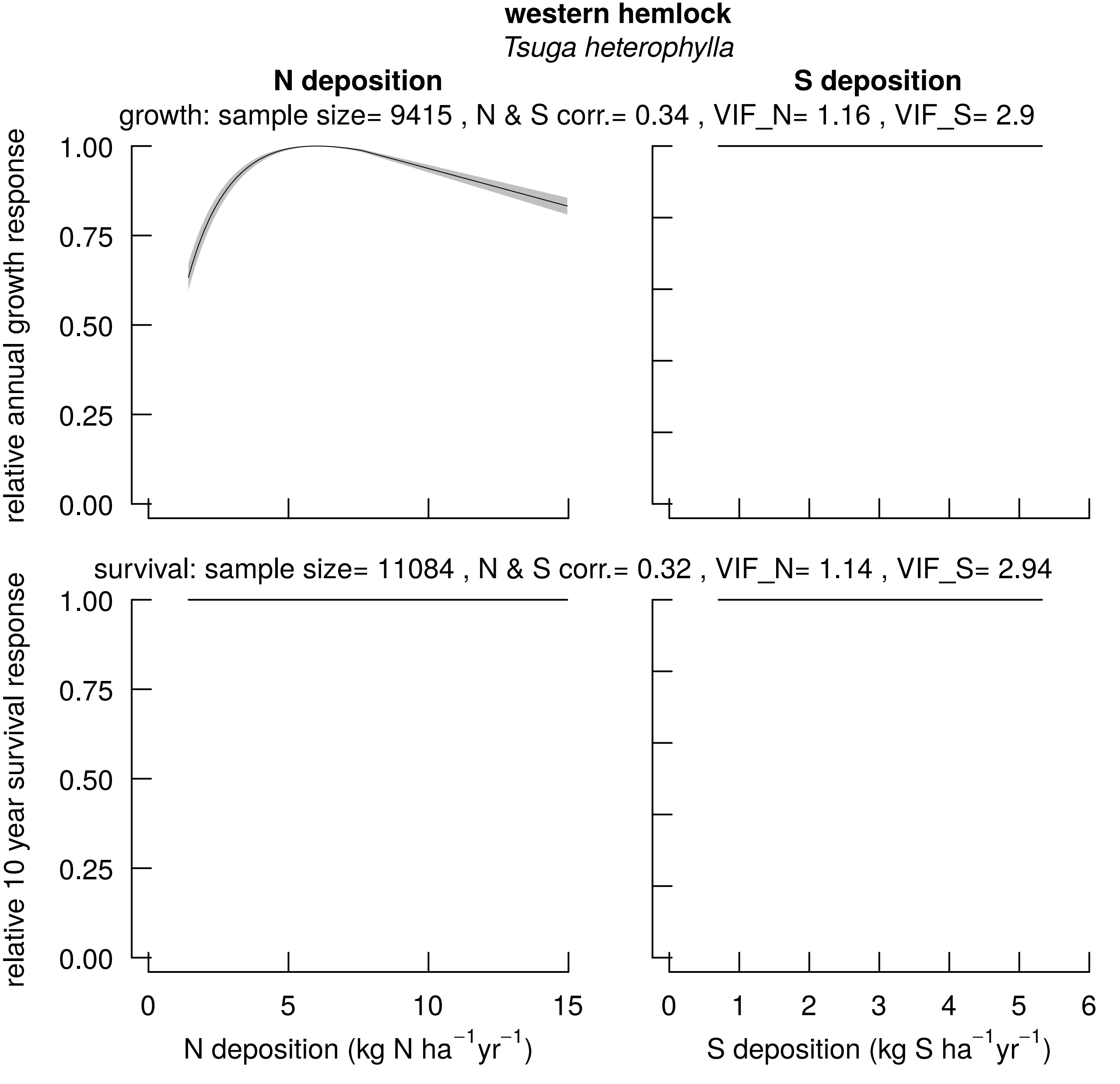
western hemlock
Tsuga heterophylla
N deposition
S deposition
growth: sample size= 9415 , N & S corr.= 0.34 , VIF_N= 1.16 , VIF_S= 2.9
relative annual growth response
1.00
0.75
0.50
0.25
0.00
survival: sample size= 11084 , N & S corr.= 0.32 , VIF_N= 1.14 , VIF_S= 2.94
relative 10 year survival response
1.00
0.75
0.50
0.25
0.00
0
5
10
15
0
1
2
3
4
5
6
N deposition (kg N ha^{-1}yr^{-1})
S deposition (kg S ha^{-1}yr^{-1})

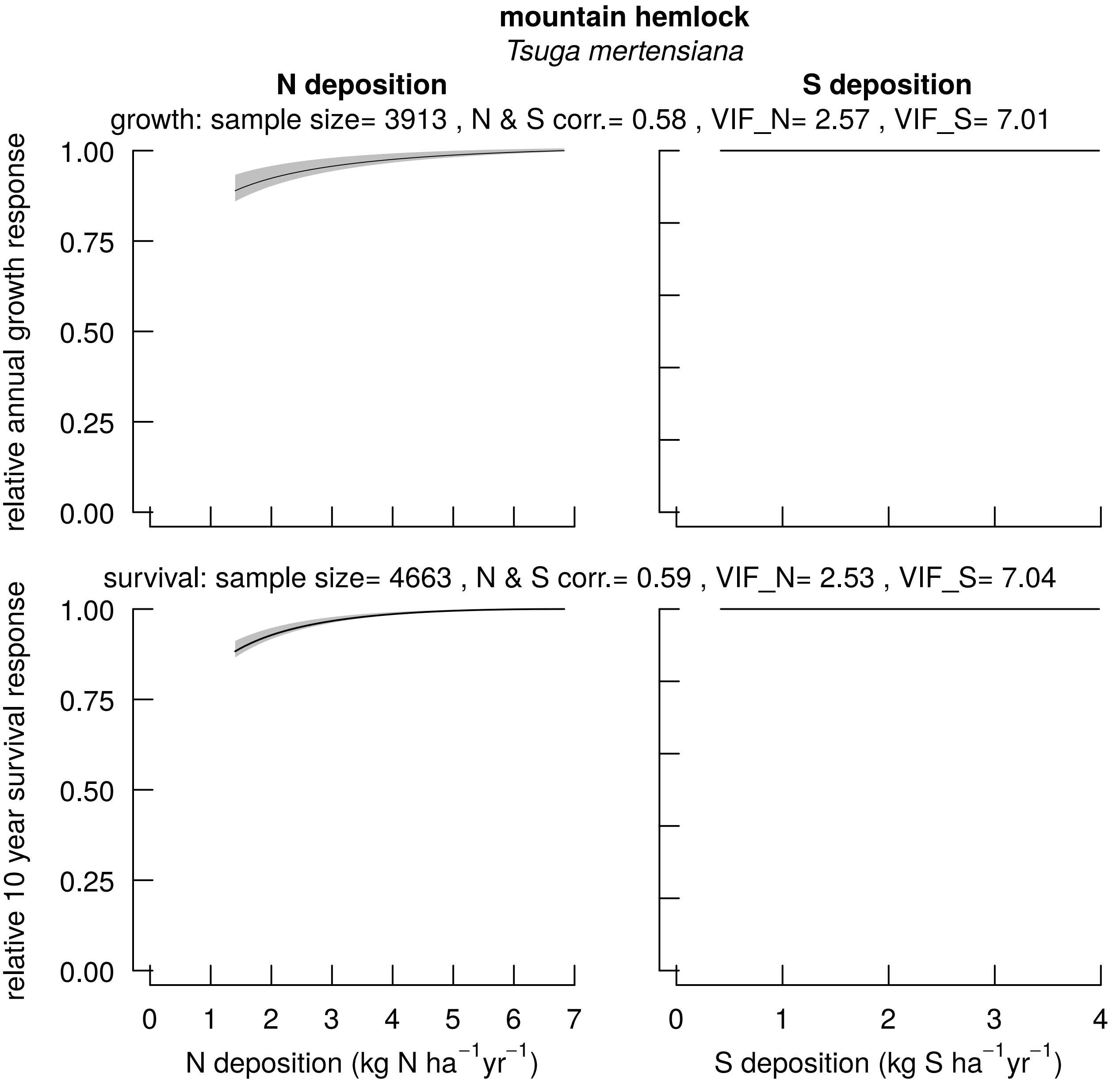

mountain hemlock
Tsuga mertensiana
N deposition
S deposition
growth: sample size= 3913 , N & S corr.= 0.58 , VIF_N= 2.57 , VIF_S= 7.01
relative annual growth response
1.00
0.75
0.50
0.25
0.00
survival: sample size= 4663 , N & S corr.= 0.59 , VIF_N= 2.53 , VIF_S= 7.04
relative 10 year survival response
1.00
0.75
0.50
0.25
0.00
0 1 2 3 4 5 6 7
0 1 2 3 4
N deposition (kg N ha−1yr−1)
S deposition (kg S ha−1yr−1)

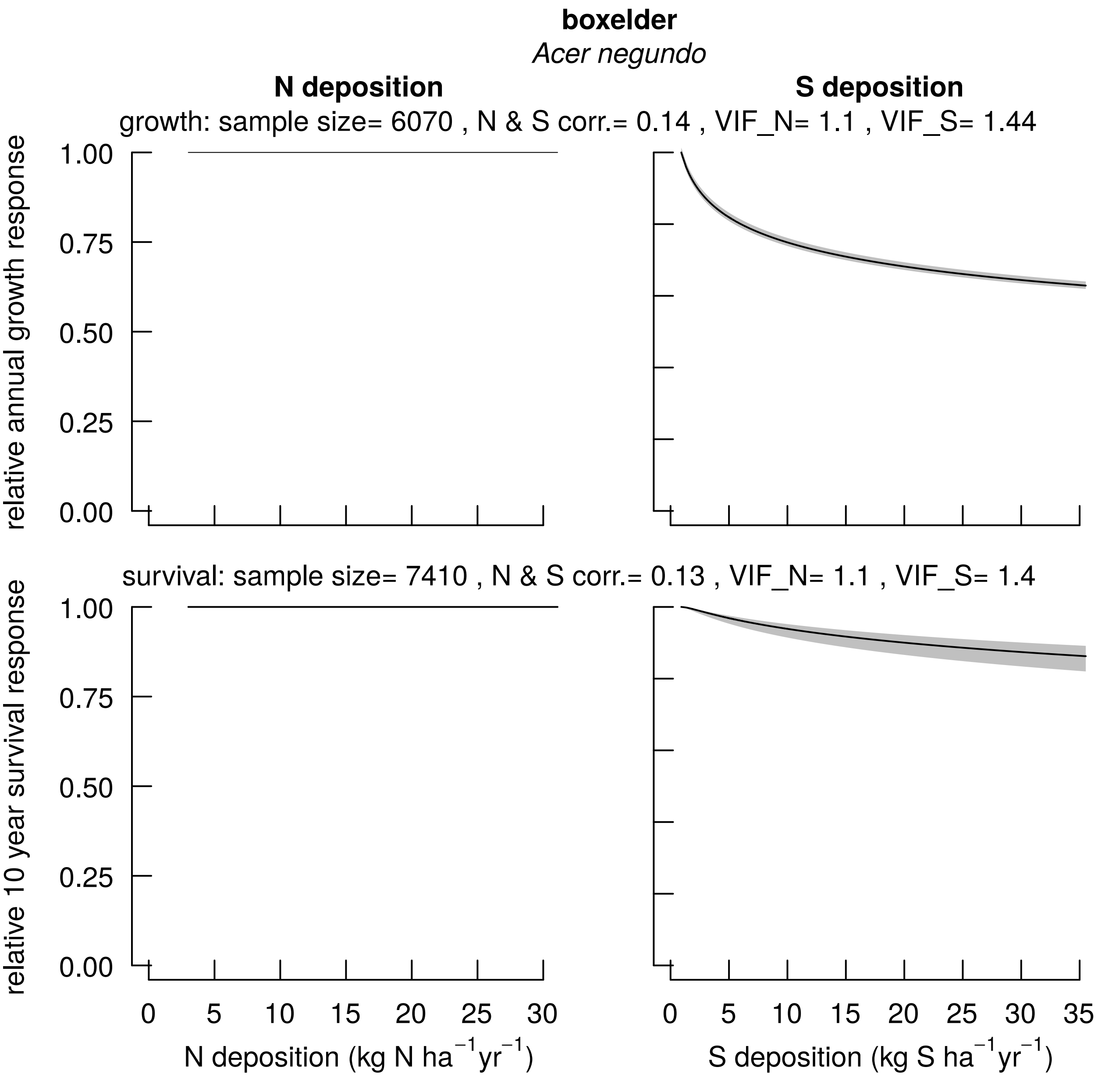

boxelder
Acer negundo
N deposition
S deposition
growth: sample size= 6070 , N & S corr.= 0.14 , VIF_N= 1.1 , VIF_S= 1.44
relative annual growth response
1.00
0.75
0.50
0.25
0.00
survival: sample size= 7410 , N & S corr.= 0.13 , VIF_N= 1.1 , VIF_S= 1.4
relative 10 year survival response
1.00
0.75
0.50
0.25
0.00
0 5 10 15 20 25 30
0 5 10 15 20 25 30 35
N deposition (kg N ha^{-1}yr^{-1})
S deposition (kg S ha^{-1}yr^{-1})

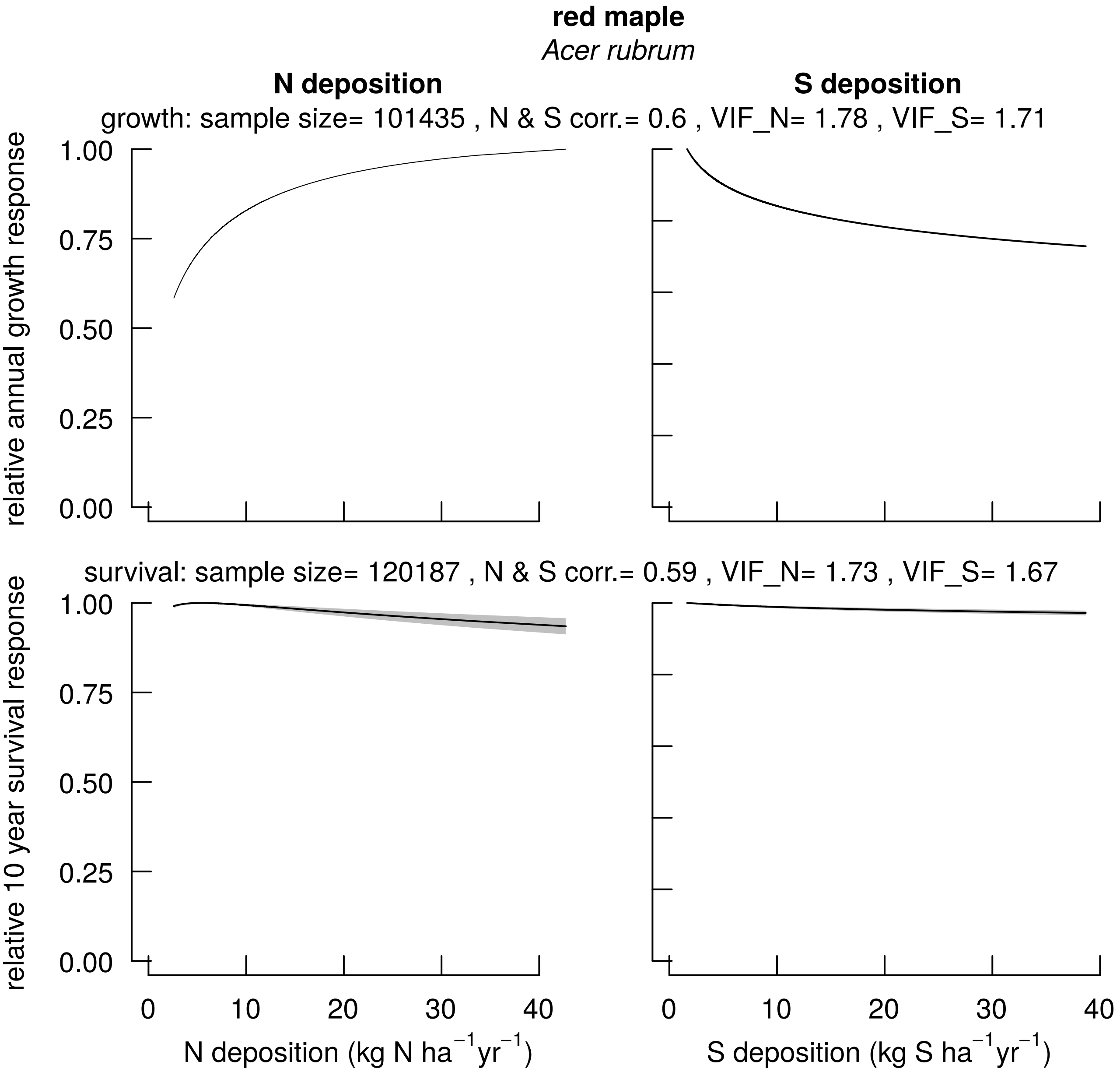

red maple
Acer rubrum
N deposition
S deposition
growth: sample size= 101435 , N & S corr.= 0.6 , VIF_N= 1.78 , VIF_S= 1.71
relative annual growth response
1.00
0.75
0.50
0.25
0.00
survival: sample size= 120187 , N & S corr.= 0.59 , VIF_N= 1.73 , VIF_S= 1.67
relative 10 year survival response
1.00
0.75
0.50
0.25
0.00
0
10
20
30
40
N deposition (kg N ha^{-1}yr^{-1})
0
10
20
30
40
S deposition (kg S ha^{-1}yr^{-1})

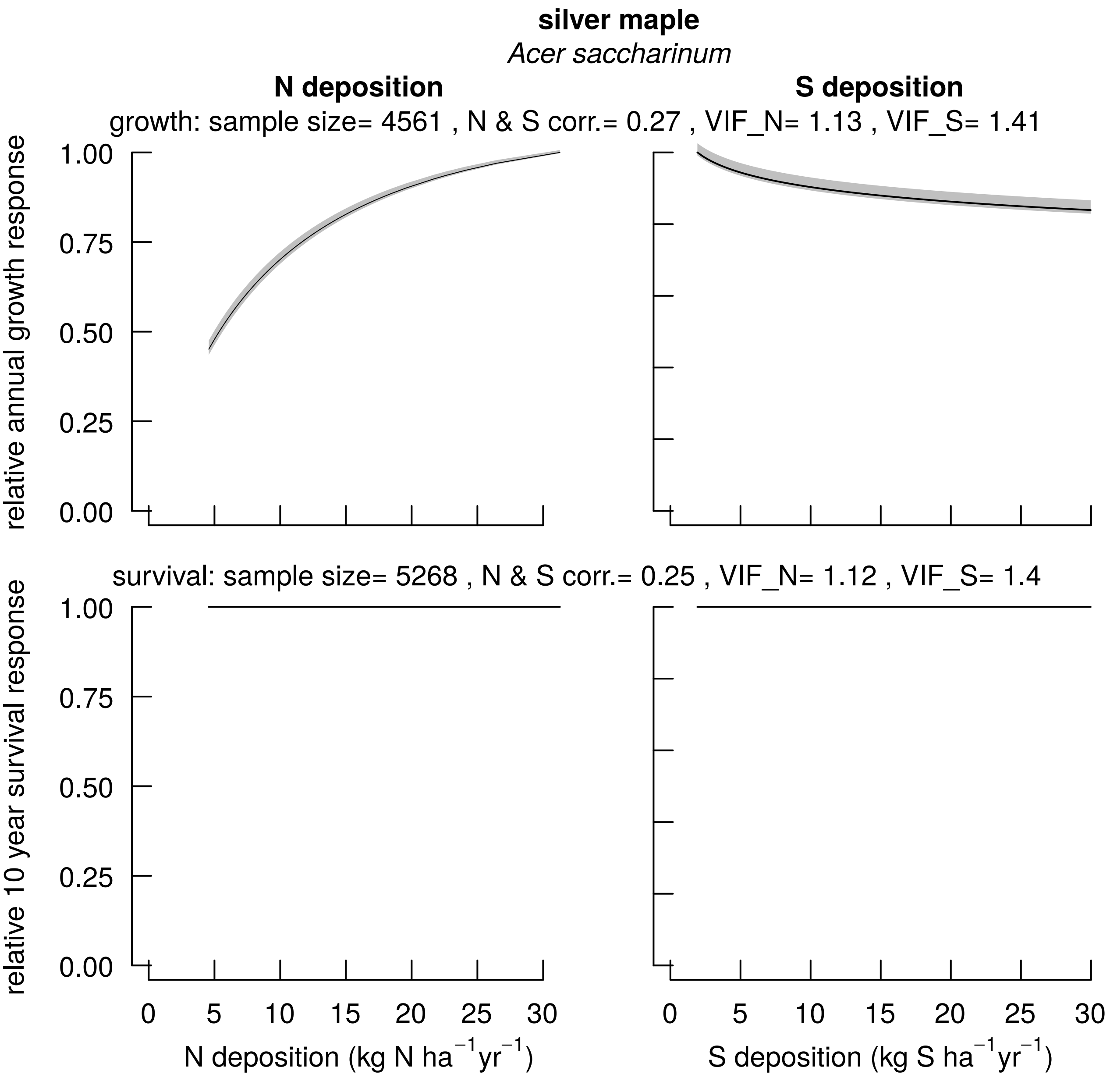

silver maple
Acer saccharinum
N deposition
S deposition
growth: sample size= 4561 , N & S corr.= 0.27 , VIF_N= 1.13 , VIF_S= 1.41
relative annual growth response
1.00
0.75
0.50
0.25
0.00
survival: sample size= 5268 , N & S corr.= 0.25 , VIF_N= 1.12 , VIF_S= 1.4
relative 10 year survival response
1.00
0.75
0.50
0.25
0.00
0
5
10
15
20
25
30
N deposition (kg N ha^{-1}yr^{-1})
0
5
10
15
20
25
30
S deposition (kg S ha^{-1}yr^{-1})

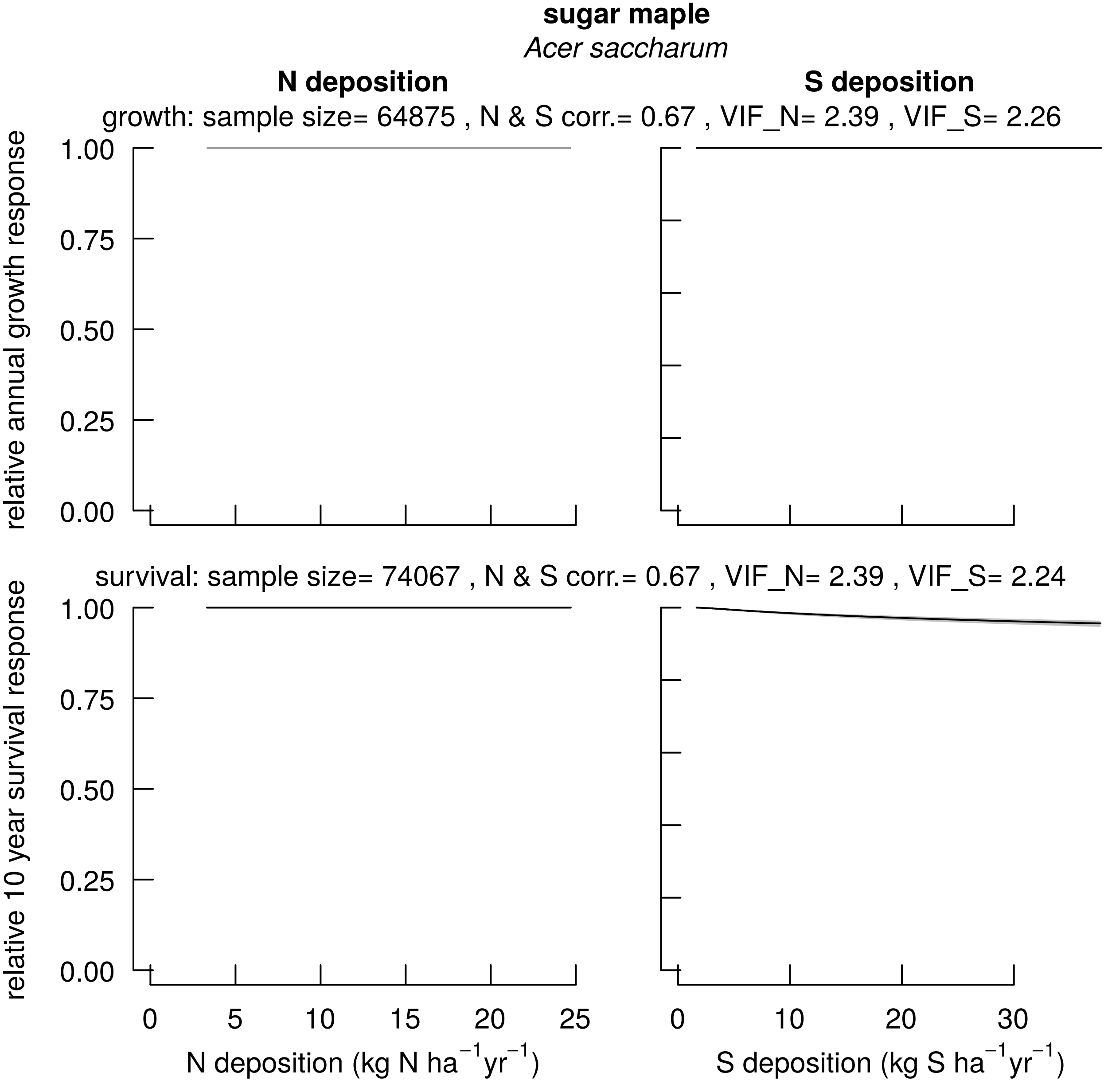

sugar maple
Acer saccharum
N deposition
S deposition
growth: sample size= 64875 , N & S corr.= 0.67 , VIF_N= 2.39 , VIF_S= 2.26
relative annual growth response
1.00
0.75
0.50
0.25
0.00
survival: sample size= 74067 , N & S corr.= 0.67 , VIF_N= 2.39 , VIF_S= 2.24
relative 10 year survival response
1.00
0.75
0.50
0.25
0.00
0
5
10
15
20
25
0
10
20
30
N deposition (kg N ha^{-1}yr^{-1})
S deposition (kg S ha^{-1}yr^{-1})

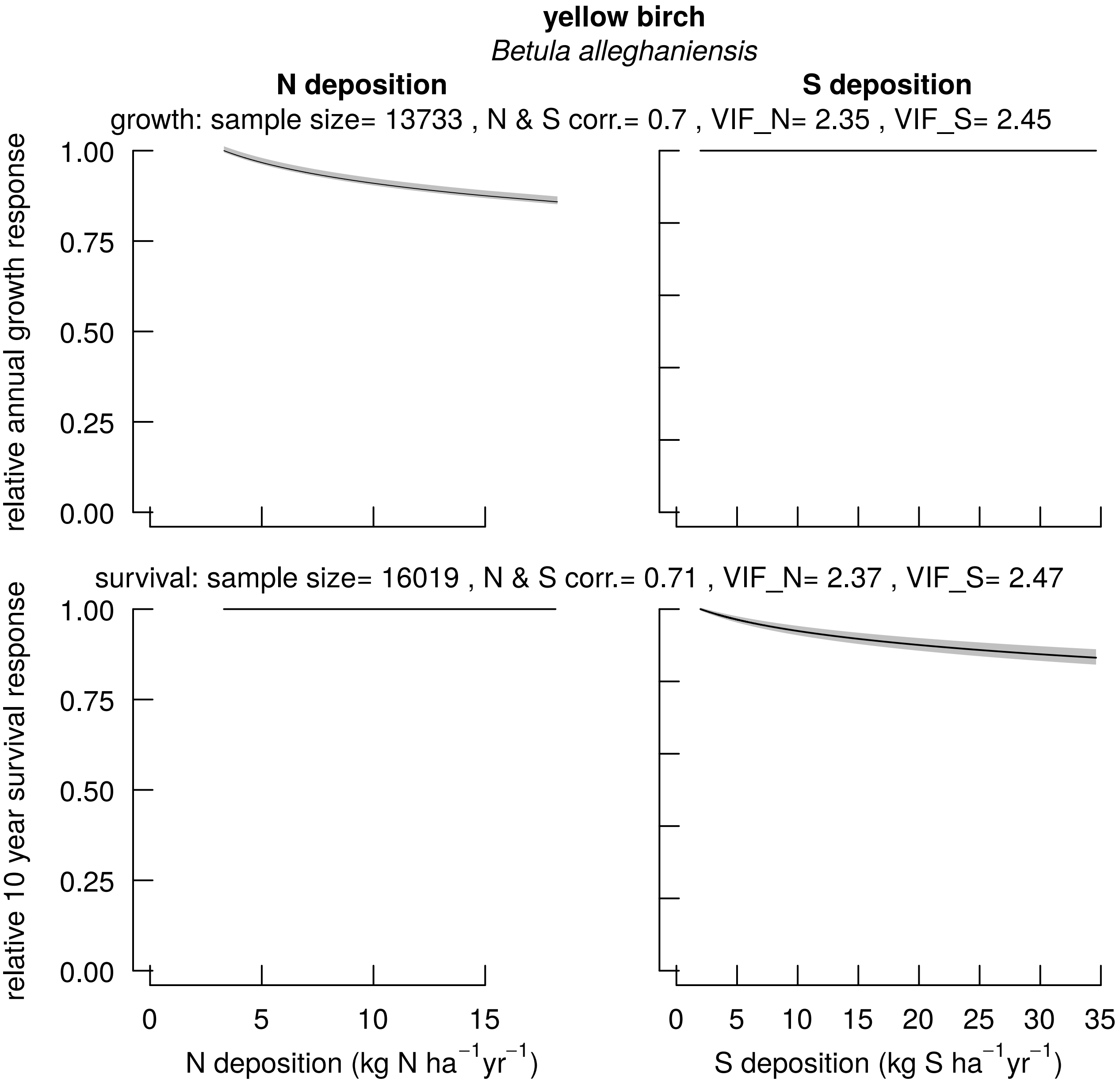

yellow birch
Betula alleghaniensis
N deposition
S deposition
growth: sample size= 13733 , N & S corr.= 0.7 , VIF_N= 2.35 , VIF_S= 2.45
relative annual growth response
1.00
0.75
0.50
0.25
0.00
survival: sample size= 16019 , N & S corr.= 0.71 , VIF_N= 2.37 , VIF_S= 2.47
relative 10 year survival response
1.00
0.75
0.50
0.25
0.00
0
5
10
15
N deposition (kg N ha^{-1}yr^{-1})
0
5
10
15
20
25
30
35
S deposition (kg S ha^{-1}yr^{-1})

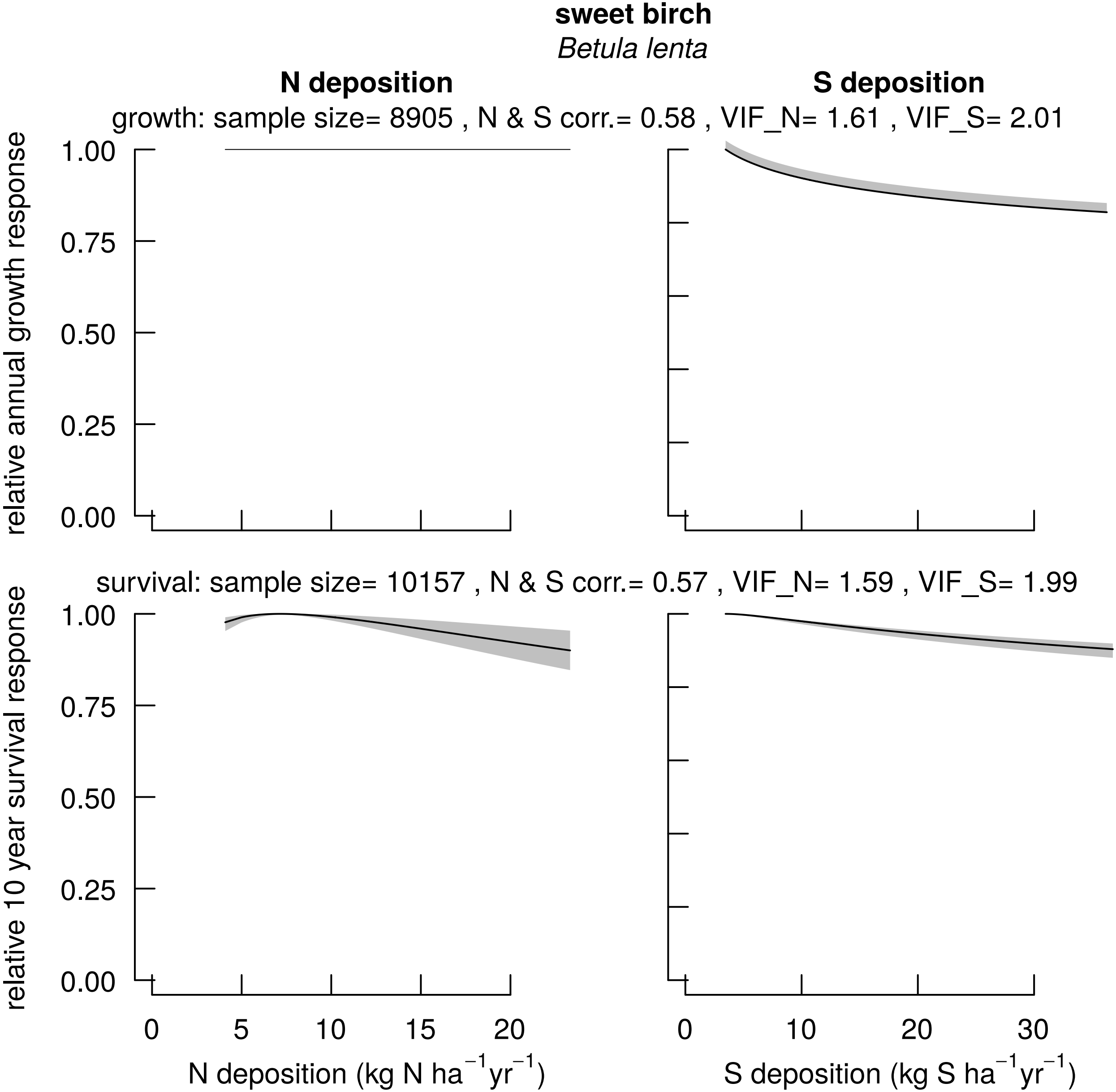
sweet birch
Betula lenta
N deposition
S deposition
growth: sample size= 8905 , N & S corr.= 0.58 , VIF_N= 1.61 , VIF_S= 2.01
survival: sample size= 10157 , N & S corr.= 0.57 , VIF_N= 1.59 , VIF_S= 1.99
relative annual growth response
relative 10 year survival response
1.00
0.75
0.50
0.25
0.00
0
5
10
15
20
30
N deposition (kg N ha^{-1}yr^{-1})
S deposition (kg S ha^{-1}yr^{-1})

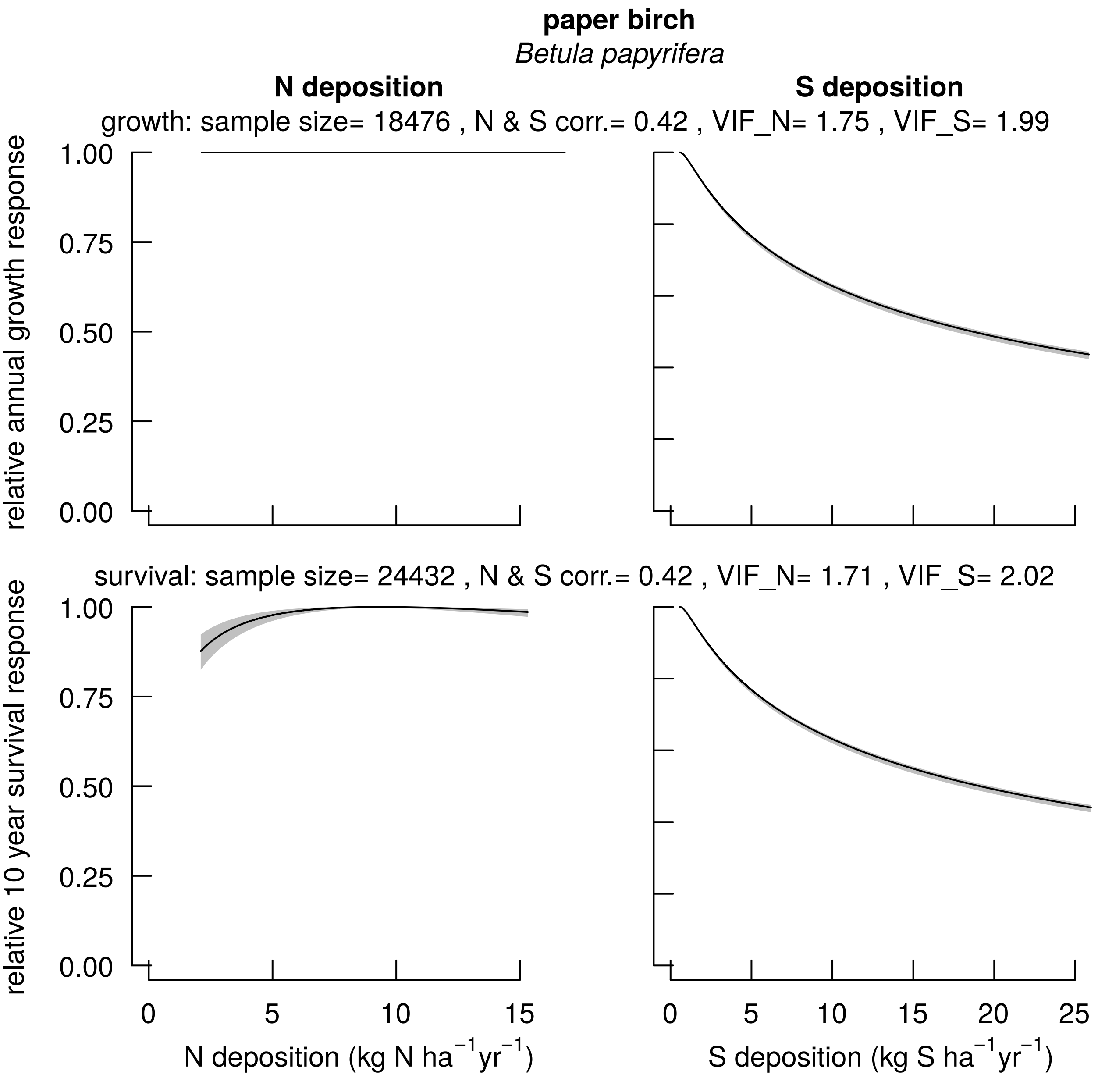
paper birch
Betula papyrifera
N deposition
S deposition
growth: sample size= 18476 , N & S corr.= 0.42 , VIF_N= 1.75 , VIF_S= 1.99
relative annual growth response
1.00
0.75
0.50
0.25
0.00
survival: sample size= 24432 , N & S corr.= 0.42 , VIF_N= 1.71 , VIF_S= 2.02
relative 10 year survival response
1.00
0.75
0.50
0.25
0.00
0
5
10
15
N deposition (kg N ha^{-1}yr^{-1})
0
5
10
15
20
25
S deposition (kg S ha^{-1}yr^{-1})

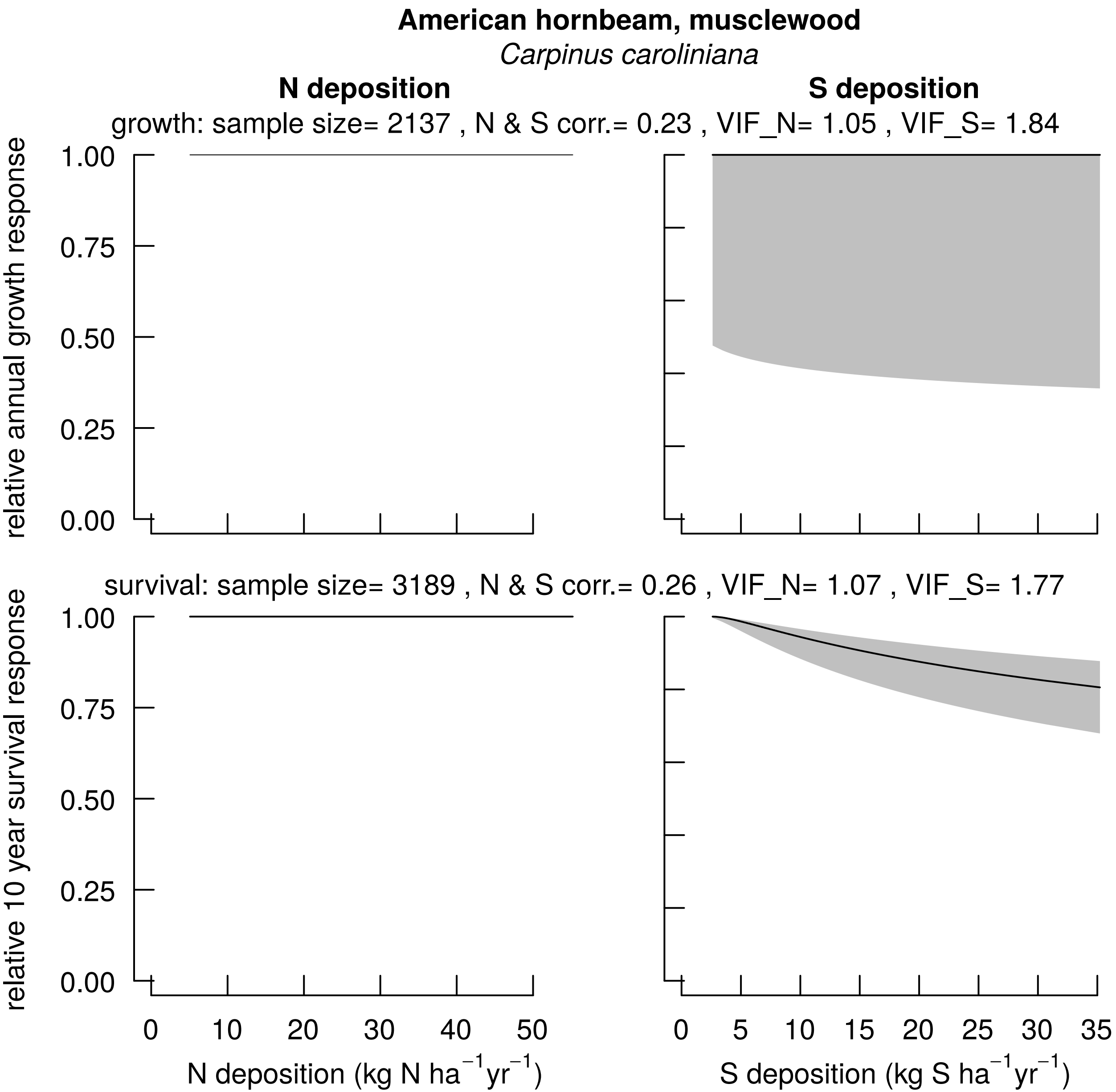

American hornbeam, musclewood
Carpinus caroliniana
N deposition
S deposition
growth: sample size= 2137 , N & S corr.= 0.23 , VIF_N= 1.05 , VIF_S= 1.84
relative annual growth response
1.00
0.75
0.50
0.25
0.00
survival: sample size= 3189 , N & S corr.= 0.26 , VIF_N= 1.07 , VIF_S= 1.77
relative 10 year survival response
1.00
0.75
0.50
0.25
0.00
0
10
20
30
40
50
N deposition (kg N ha−1yr−1)
0
5
10
15
20
25
30
35
S deposition (kg S ha−1yr−1)

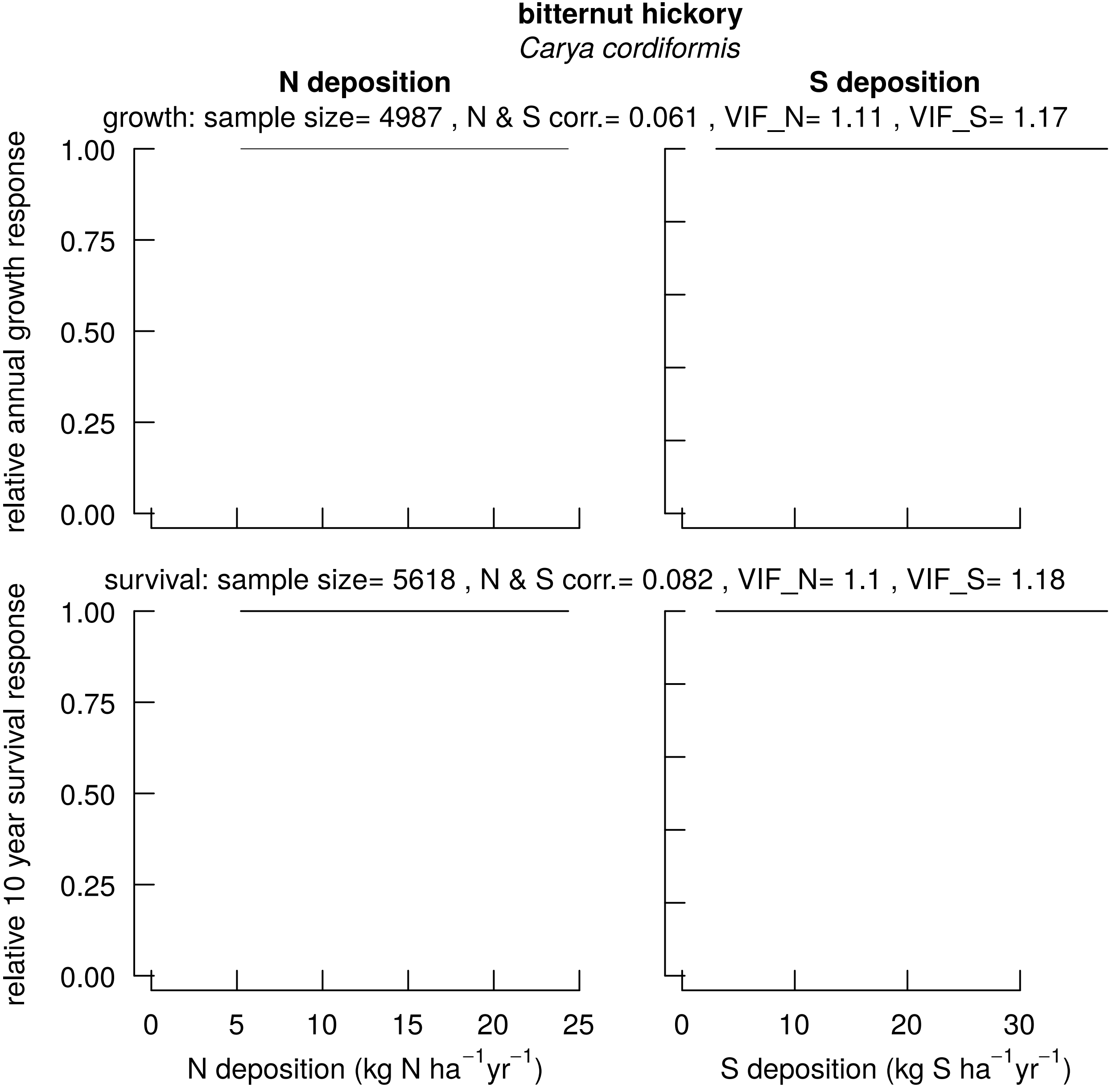

bitternut hickory
Carya cordiformis
N deposition
S deposition
growth: sample size= 4987 , N & S corr.= 0.061 , VIF_N= 1.11 , VIF_S= 1.17
relative annual growth response
1.00
0.75
0.50
0.25
0.00
survival: sample size= 5618 , N & S corr.= 0.082 , VIF_N= 1.1 , VIF_S= 1.18
relative 10 year survival response
1.00
0.75
0.50
0.25
0.00
0
5
10
15
20
25
0
10
20
30
N deposition (kg N ha^{-1}yr^{-1})
S deposition (kg S ha^{-1}yr^{-1})

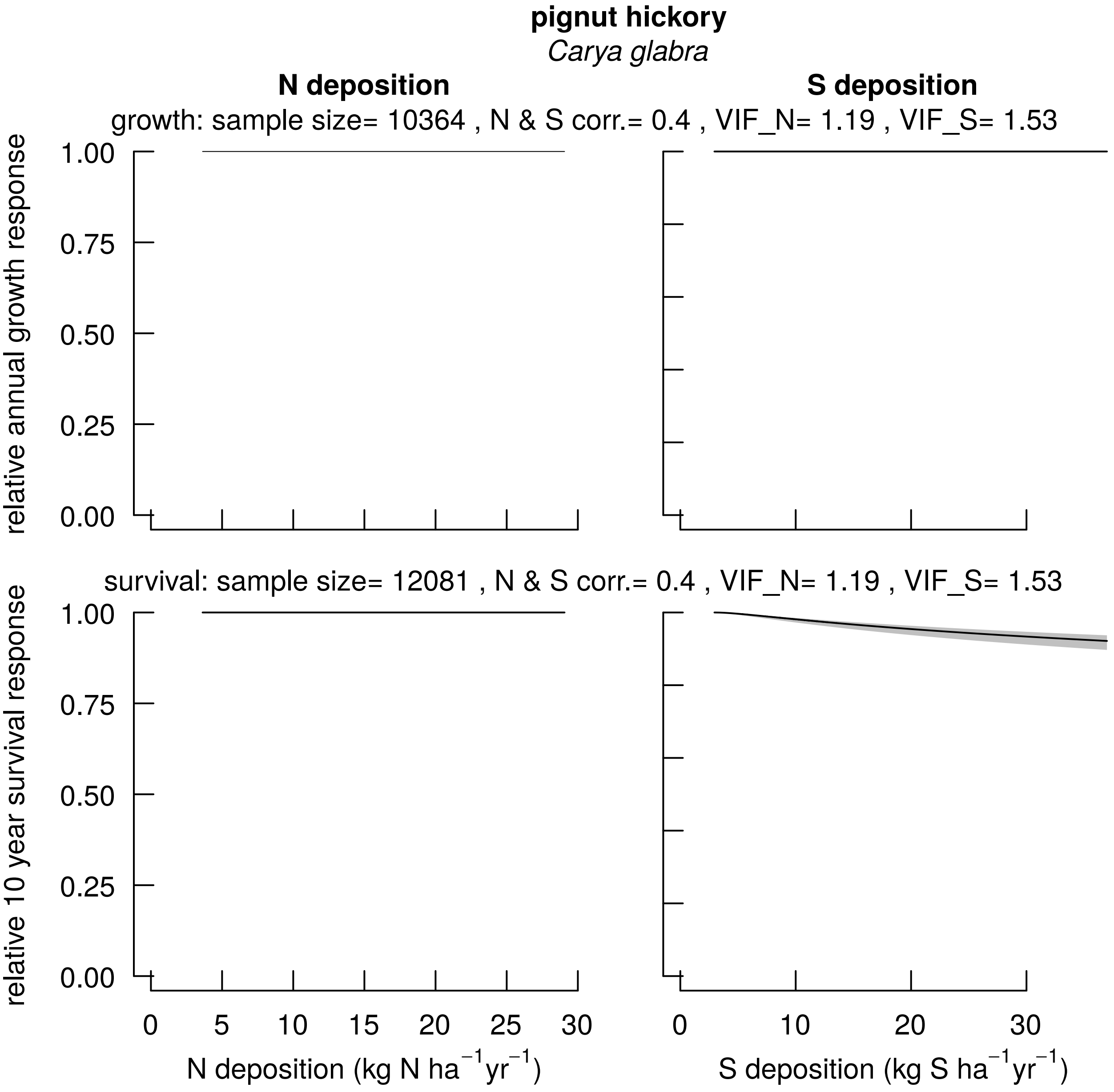
pignut hickory
Carya glabra
N deposition
S deposition
growth: sample size= 10364 , N & S corr.= 0.4 , VIF_N= 1.19 , VIF_S= 1.53
relative annual growth response
1.00
0.75
0.50
0.25
0.00
survival: sample size= 12081 , N & S corr.= 0.4 , VIF_N= 1.19 , VIF_S= 1.53
relative 10 year survival response
1.00
0.75
0.50
0.25
0.00
0
5
10
15
20
25
30
N deposition (kg N ha^{-1}yr^{-1})
0
10
20
30
S deposition (kg S ha^{-1}yr^{-1})

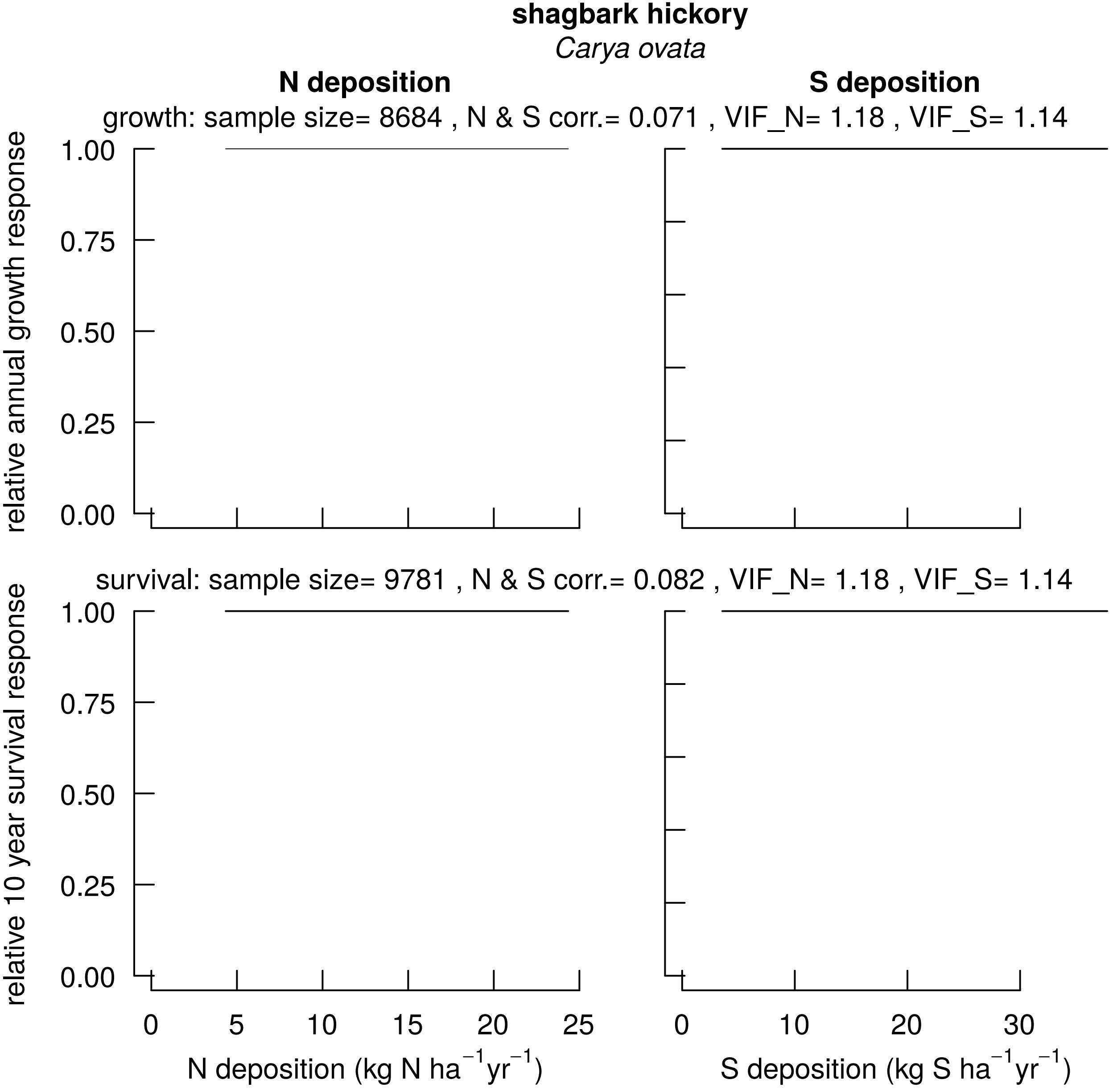

shagbark hickory
Carya ovata
N deposition
S deposition
growth: sample size= 8684 , N & S corr.= 0.071 , VIF_N= 1.18 , VIF_S= 1.14
relative annual growth response
1.00
0.75
0.50
0.25
0.00
survival: sample size= 9781 , N & S corr.= 0.082 , VIF_N= 1.18 , VIF_S= 1.14
relative 10 year survival response
1.00
0.75
0.50
0.25
0.00
0
5
10
15
20
25
0
10
20
30
N deposition (kg N ha⁻¹yr⁻¹)
S deposition (kg S ha⁻¹yr⁻¹)

black hickory
Carya texana

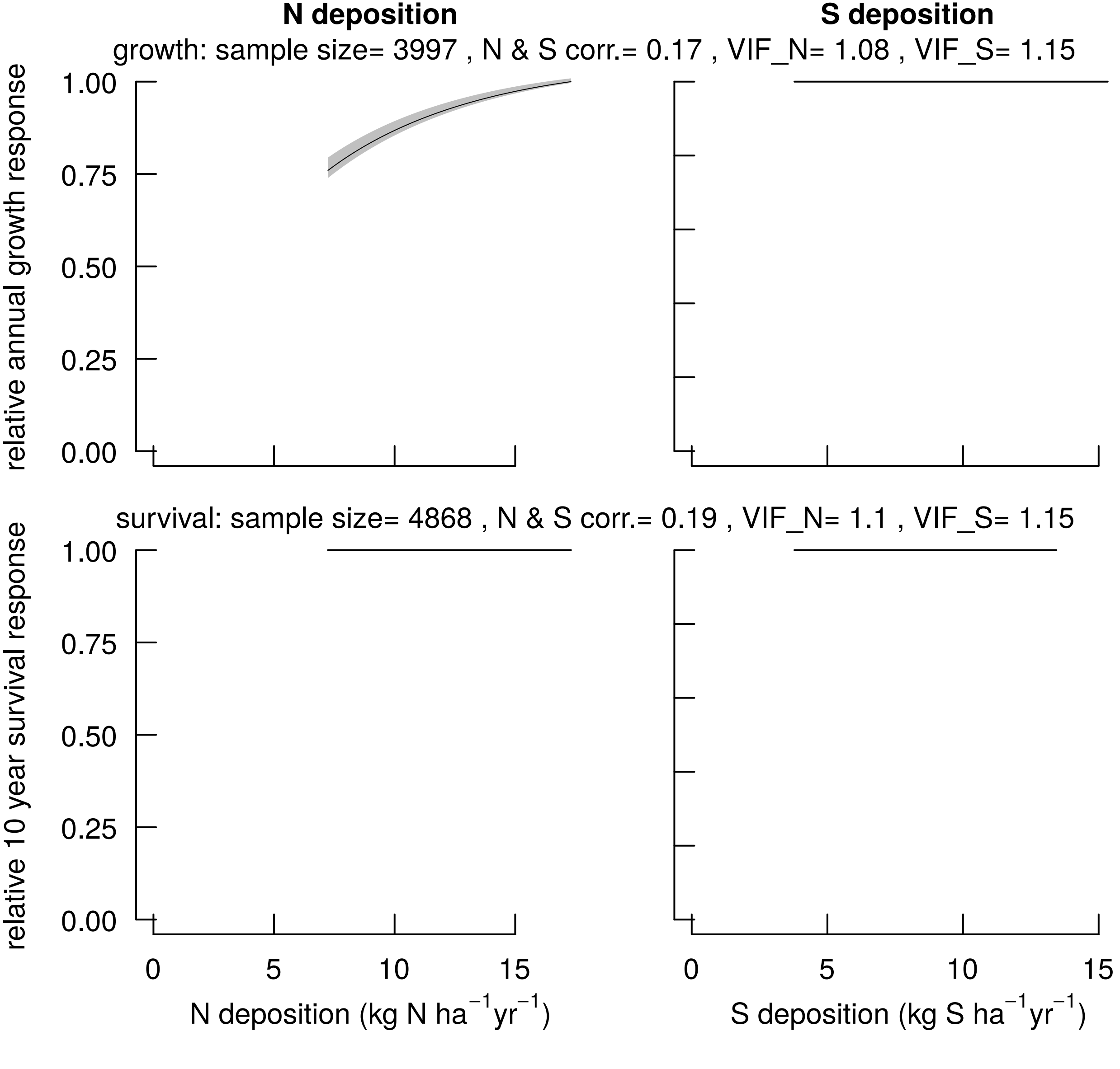

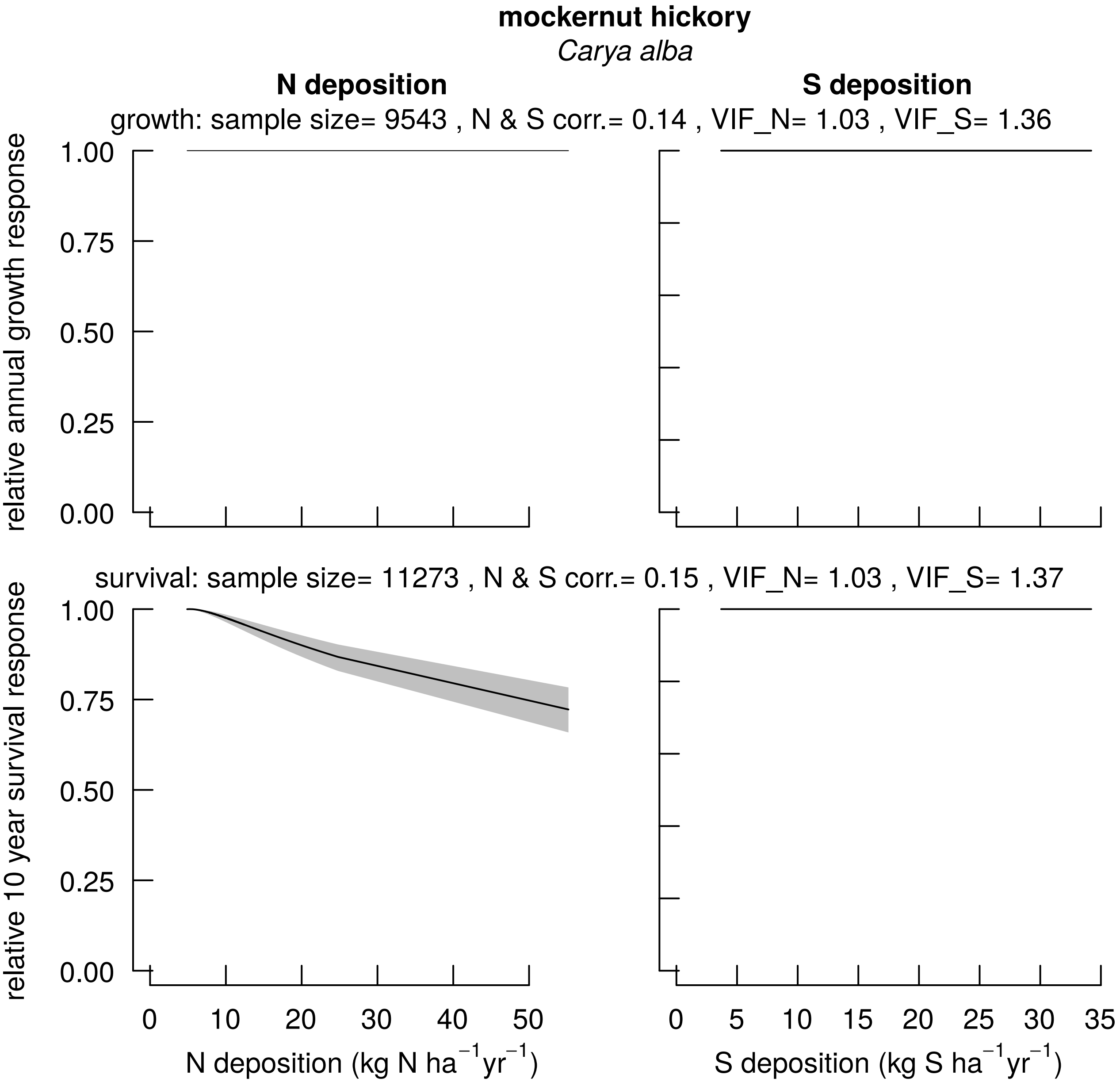
mockernut hickory
Carya alba
N deposition
S deposition
growth: sample size= 9543 , N & S corr.= 0.14 , VIF_N= 1.03 , VIF_S= 1.36
survival: sample size= 11273 , N & S corr.= 0.15 , VIF_N= 1.03 , VIF_S= 1.37
relative annual growth response
relative 10 year survival response
1.00
0.75
0.50
0.25
0.00
0
10
20
30
40
50
5
15
25
35
N deposition (kg N ha−1yr−1)
S deposition (kg S ha−1yr−1)

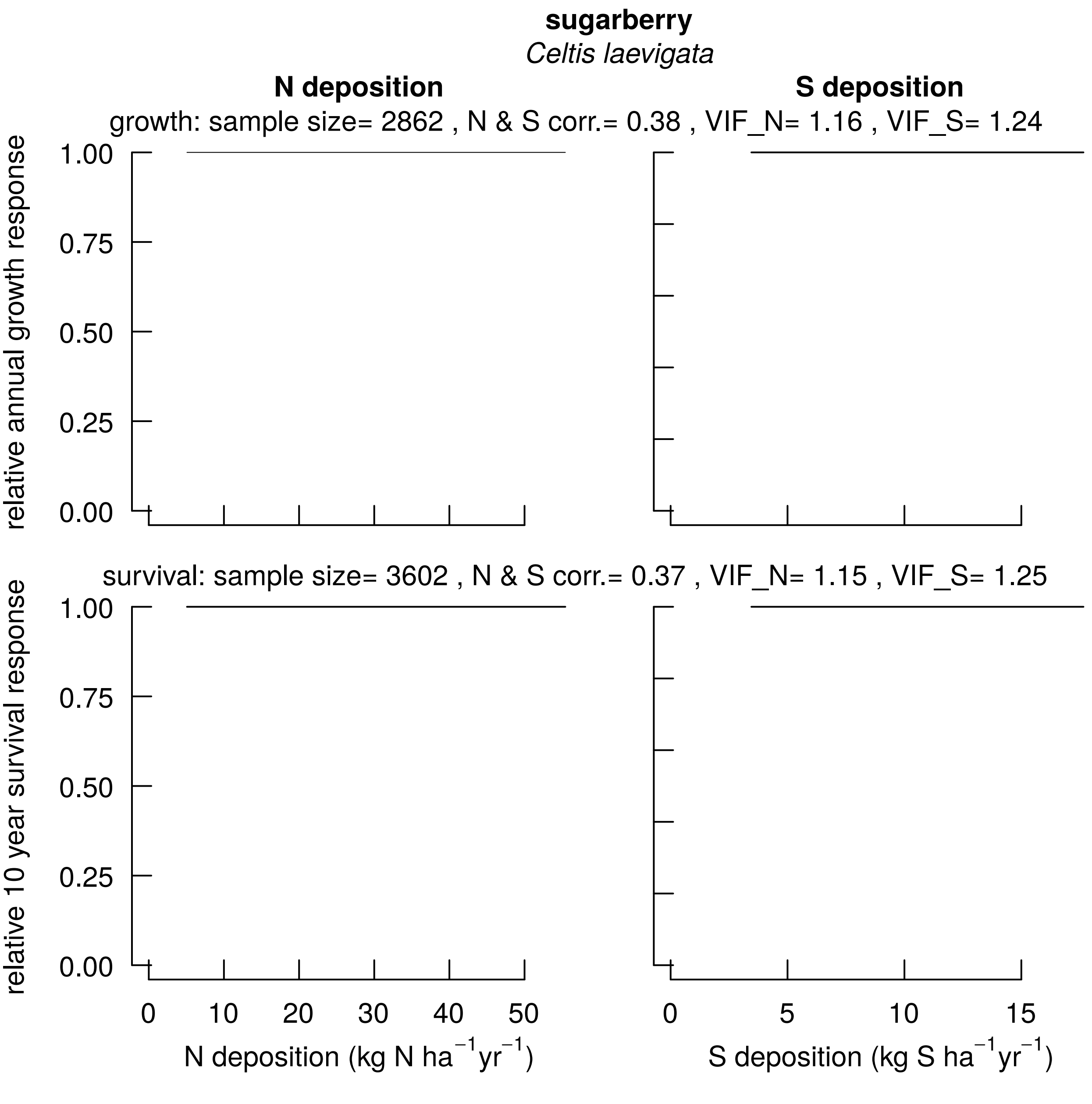

sugarberry
Celtis laevigata
N deposition
S deposition
growth: sample size= 2862 , N & S corr.= 0.38 , VIF_N= 1.16 , VIF_S= 1.24
survival: sample size= 3602 , N & S corr.= 0.37 , VIF_N= 1.15 , VIF_S= 1.25
relative annual growth response
relative 10 year survival response
1.00
0.75
0.50
0.25
0.00
0
10
20
30
40
50
5
15
N deposition (kg N ha^{-1}yr^{-1})
S deposition (kg S ha^{-1}yr^{-1})

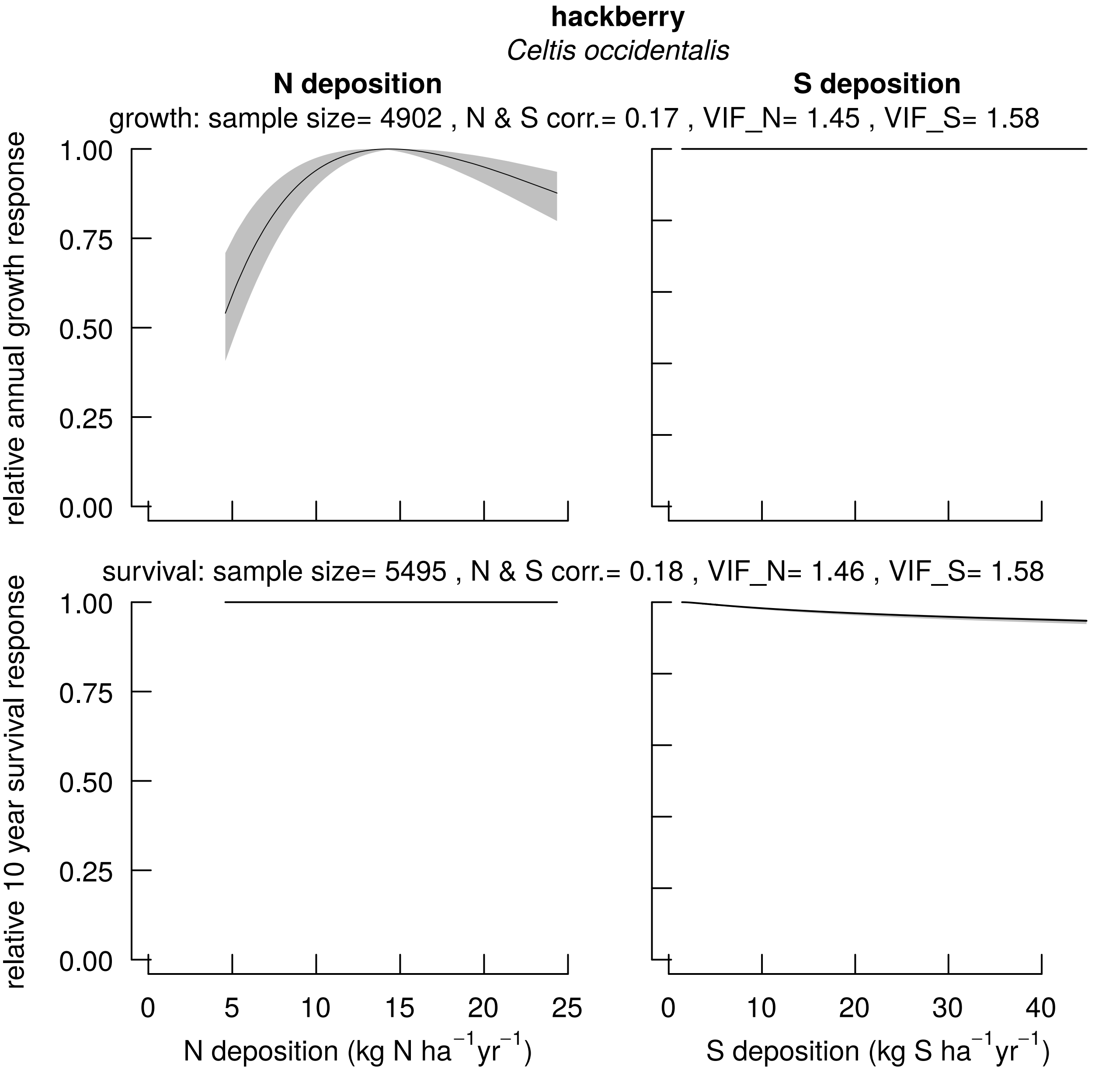
hackberry
Celtis occidentalis
N deposition
S deposition
growth: sample size= 4902 , N & S corr.= 0.17 , VIF_N= 1.45 , VIF_S= 1.58
relative annual growth response
1.00
0.75
0.50
0.25
0.00
survival: sample size= 5495 , N & S corr.= 0.18 , VIF_N= 1.46 , VIF_S= 1.58
relative 10 year survival response
1.00
0.75
0.50
0.25
0.00
0
5
10
15
20
25
N deposition (kg N ha^{-1}yr^{-1})
0
10
20
30
40
S deposition (kg S ha^{-1}yr^{-1})

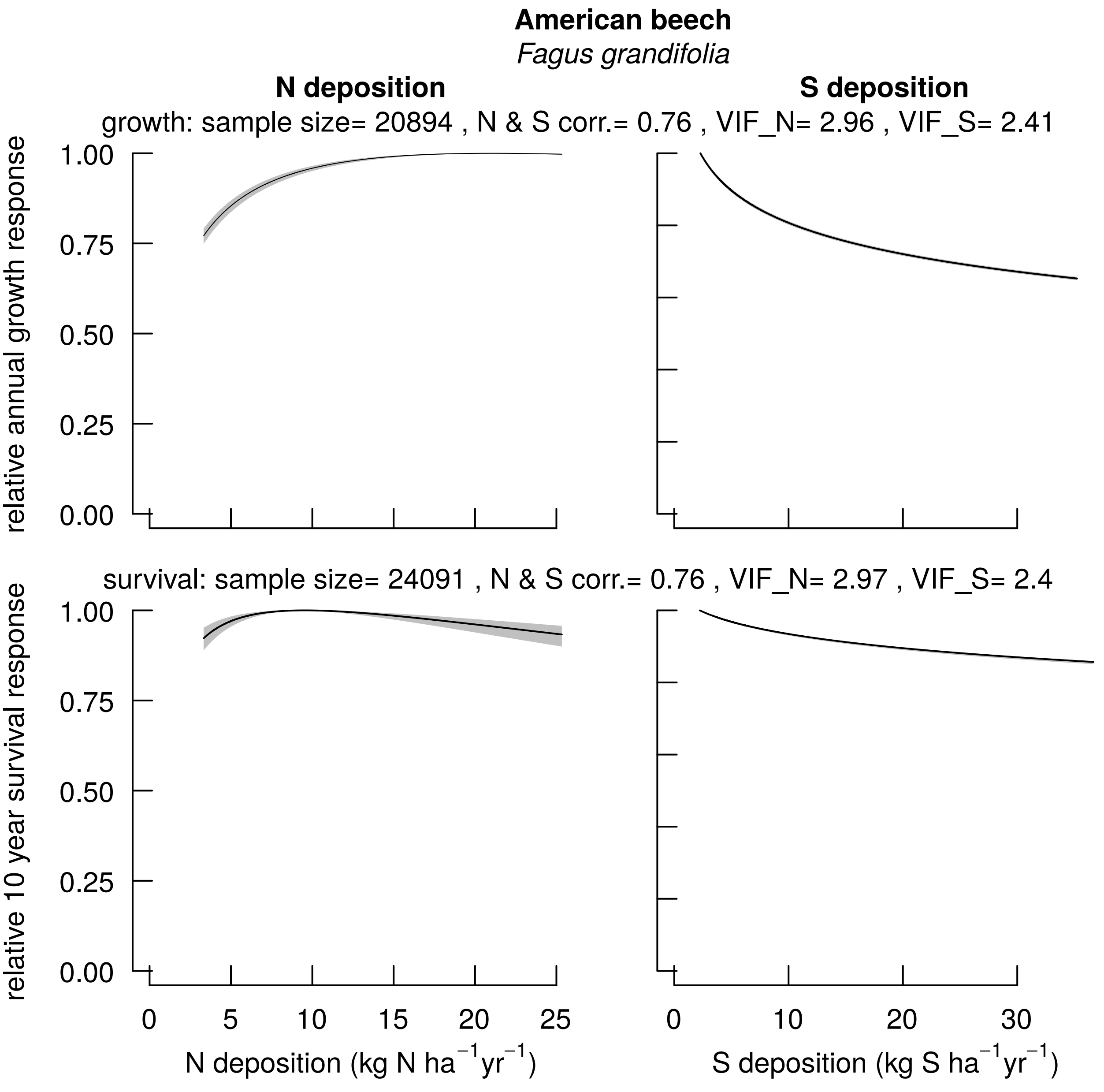
American beech
Fagus grandifolia
N deposition
S deposition
growth: sample size= 20894 , N & S corr.= 0.76 , VIF_N= 2.96 , VIF_S= 2.41
relative annual growth response
1.00
0.75
0.50
0.25
0.00
survival: sample size= 24091 , N & S corr.= 0.76 , VIF_N= 2.97 , VIF_S= 2.4
relative 10 year survival response
1.00
0.75
0.50
0.25
0.00
0 5 10 15 20 25
N deposition (kg N ha^{-1}yr^{-1})
0 10 20 30
S deposition (kg S ha^{-1}yr^{-1})

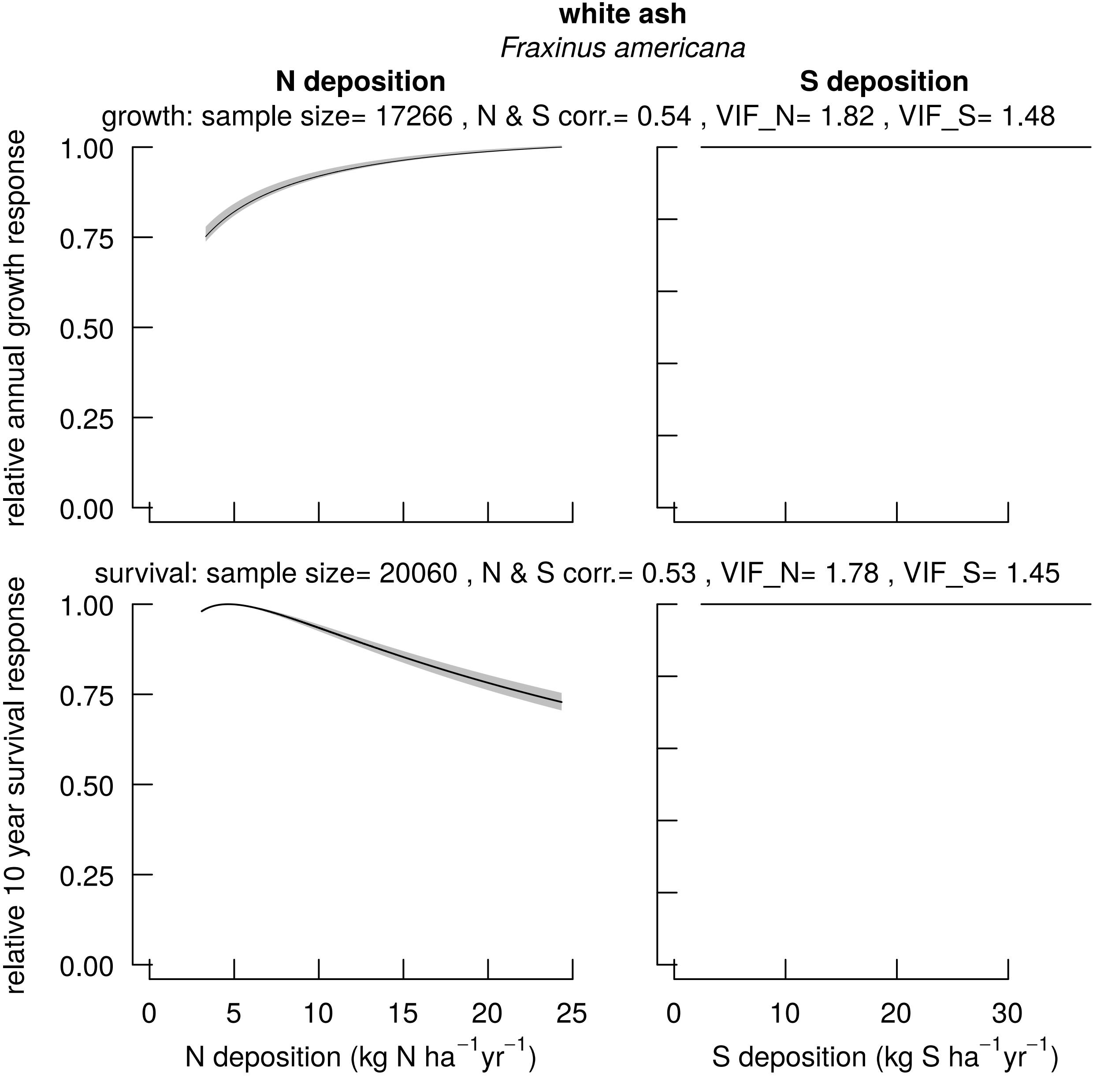

white ash
Fraxinus americana
N deposition
S deposition
growth: sample size= 17266 , N & S corr.= 0.54 , VIF_N= 1.82 , VIF_S= 1.48
survival: sample size= 20060 , N & S corr.= 0.53 , VIF_N= 1.78 , VIF_S= 1.45
relative annual growth response
relative 10 year survival response
1.00
0.75
0.50
0.25
0.00
0
5
10
15
20
25
30
N deposition (kg N $ha^{-1}yr^{-1}$)
S deposition (kg S $ha^{-1}yr^{-1}$)

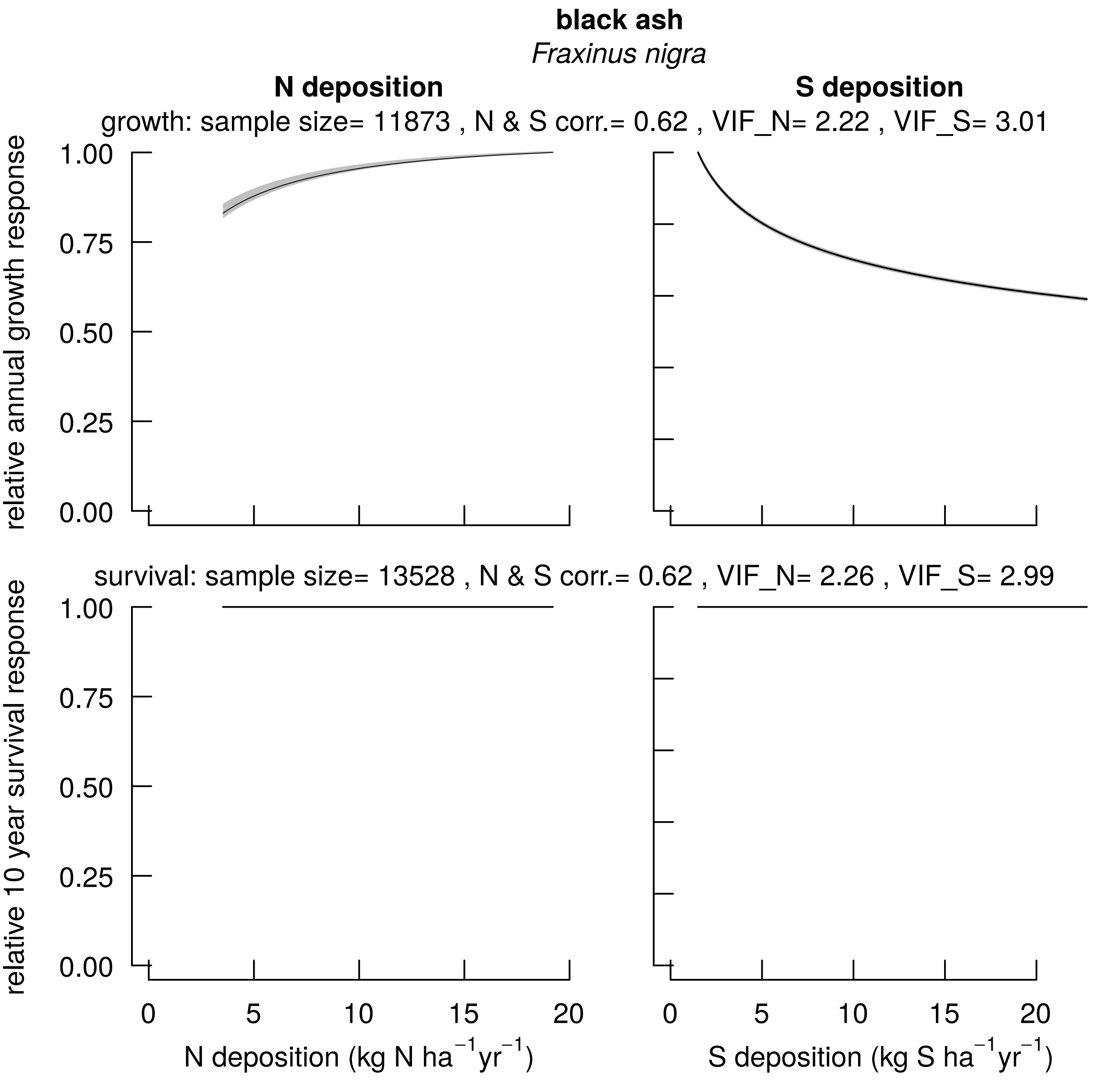
black ash
Fraxinus nigra
N deposition
S deposition
growth: sample size= 11873 , N & S corr.= 0.62 , VIF_N= 2.22 , VIF_S= 3.01
relative annual growth response
1.00
0.75
0.50
0.25
0.00
survival: sample size= 13528 , N & S corr.= 0.62 , VIF_N= 2.26 , VIF_S= 2.99
relative 10 year survival response
1.00
0.75
0.50
0.25
0.00
0
5
10
15
20
0
5
10
15
20
N deposition (kg N ha^{-1}yr^{-1})
S deposition (kg S ha^{-1}yr^{-1})

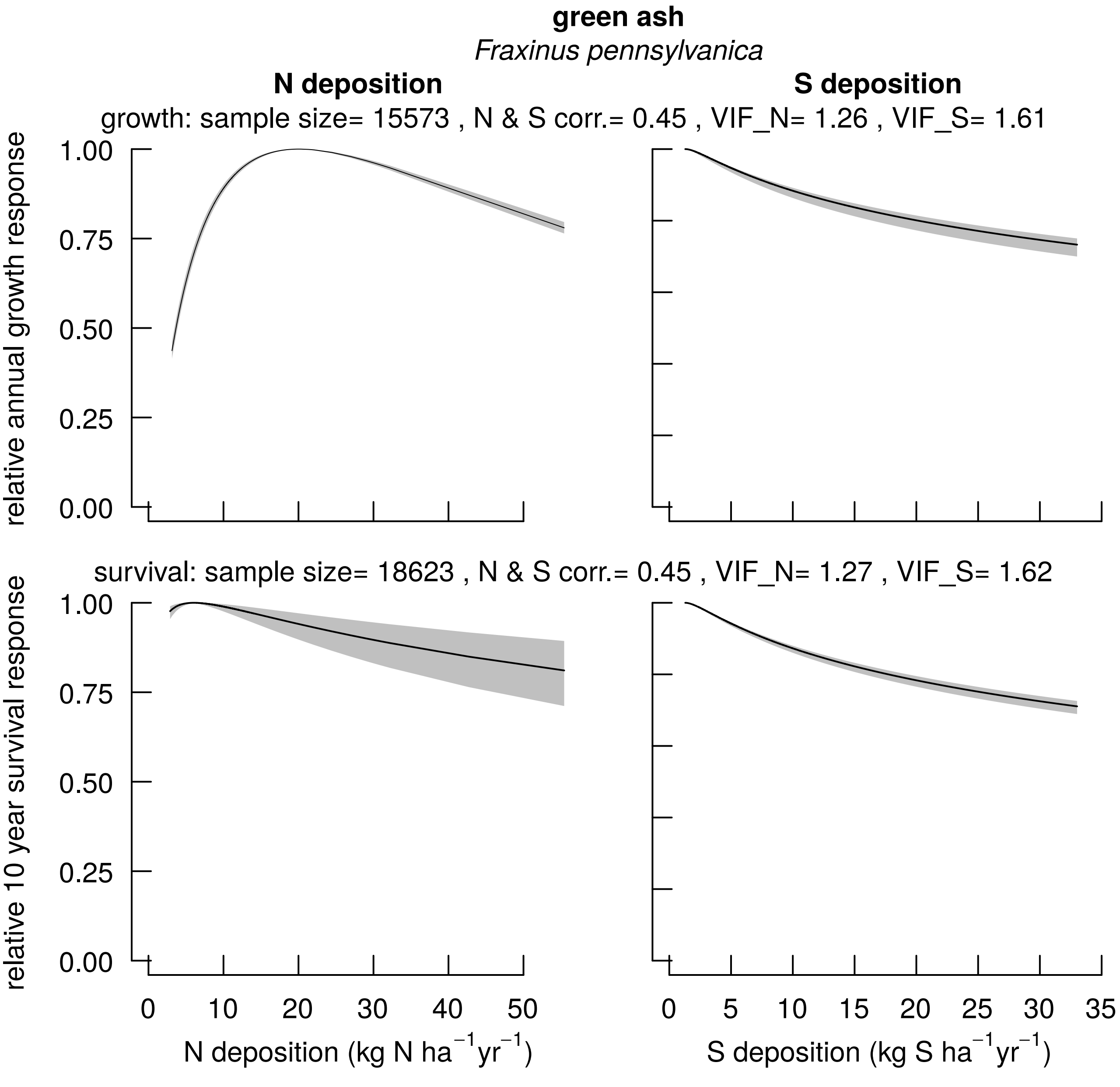

green ash
Fraxinus pennsylvanica
N deposition
S deposition
growth: sample size= 15573 , N & S corr.= 0.45 , VIF_N= 1.26 , VIF_S= 1.61
relative annual growth response
1.00
0.75
0.50
0.25
0.00
survival: sample size= 18623 , N & S corr.= 0.45 , VIF_N= 1.27 , VIF_S= 1.62
relative 10 year survival response
1.00
0.75
0.50
0.25
0.00
0
10
20
30
40
50
N deposition (kg N ha^{-1}yr^{-1})
0
5
10
15
20
25
30
35
S deposition (kg S ha^{-1}yr^{-1})

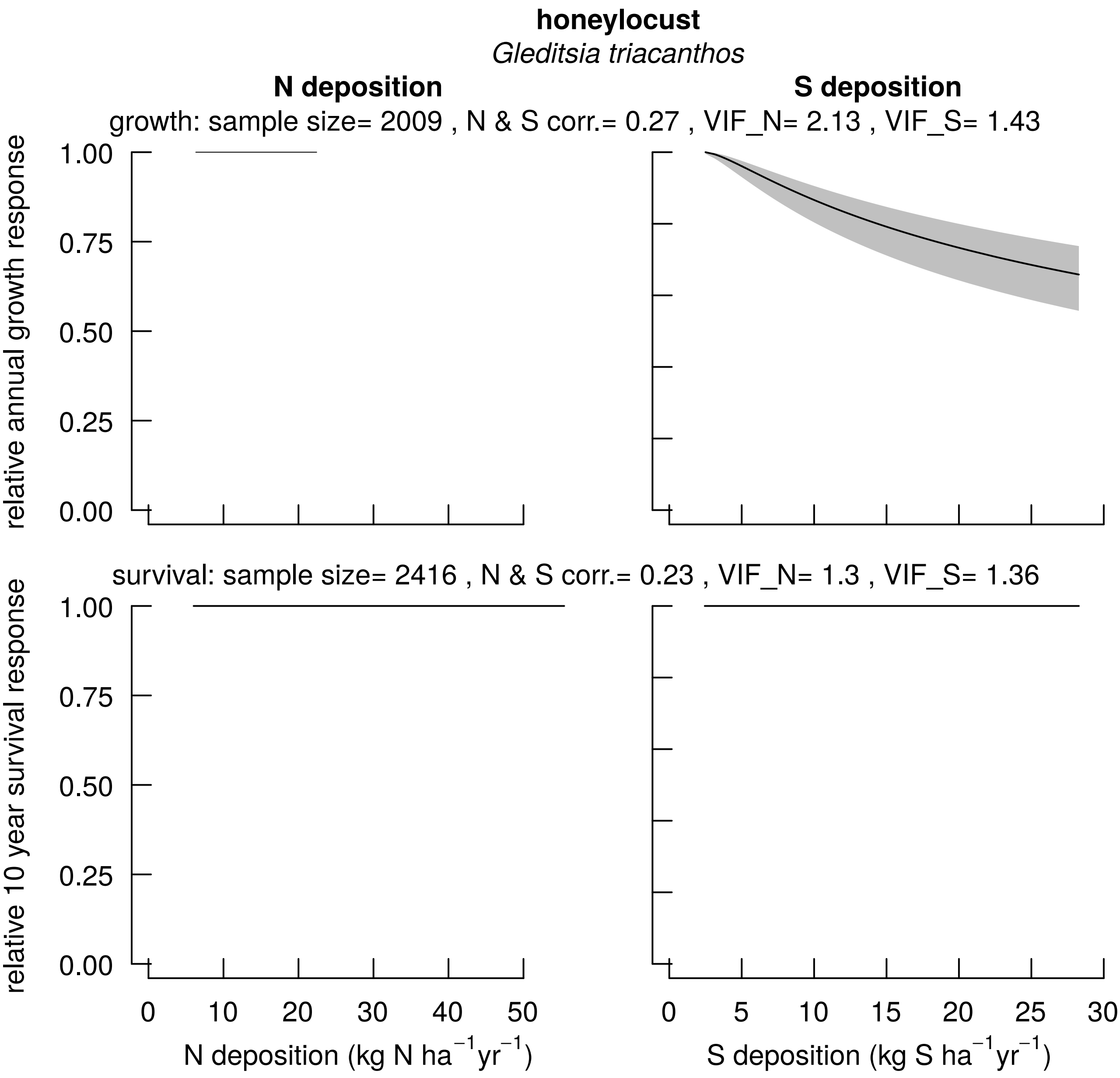
honeylocust
Gleditsia triacanthos
N deposition
S deposition
growth: sample size= 2009 , N & S corr.= 0.27 , VIF_N= 2.13 , VIF_S= 1.43
relative annual growth response
1.00
0.75
0.50
0.25
0.00
survival: sample size= 2416 , N & S corr.= 0.23 , VIF_N= 1.3 , VIF_S= 1.36
relative 10 year survival response
1.00
0.75
0.50
0.25
0.00
0
10
20
30
40
50
0
5
10
15
20
25
30
N deposition (kg N ha^{-1}yr^{-1})
S deposition (kg S ha^{-1}yr^{-1})

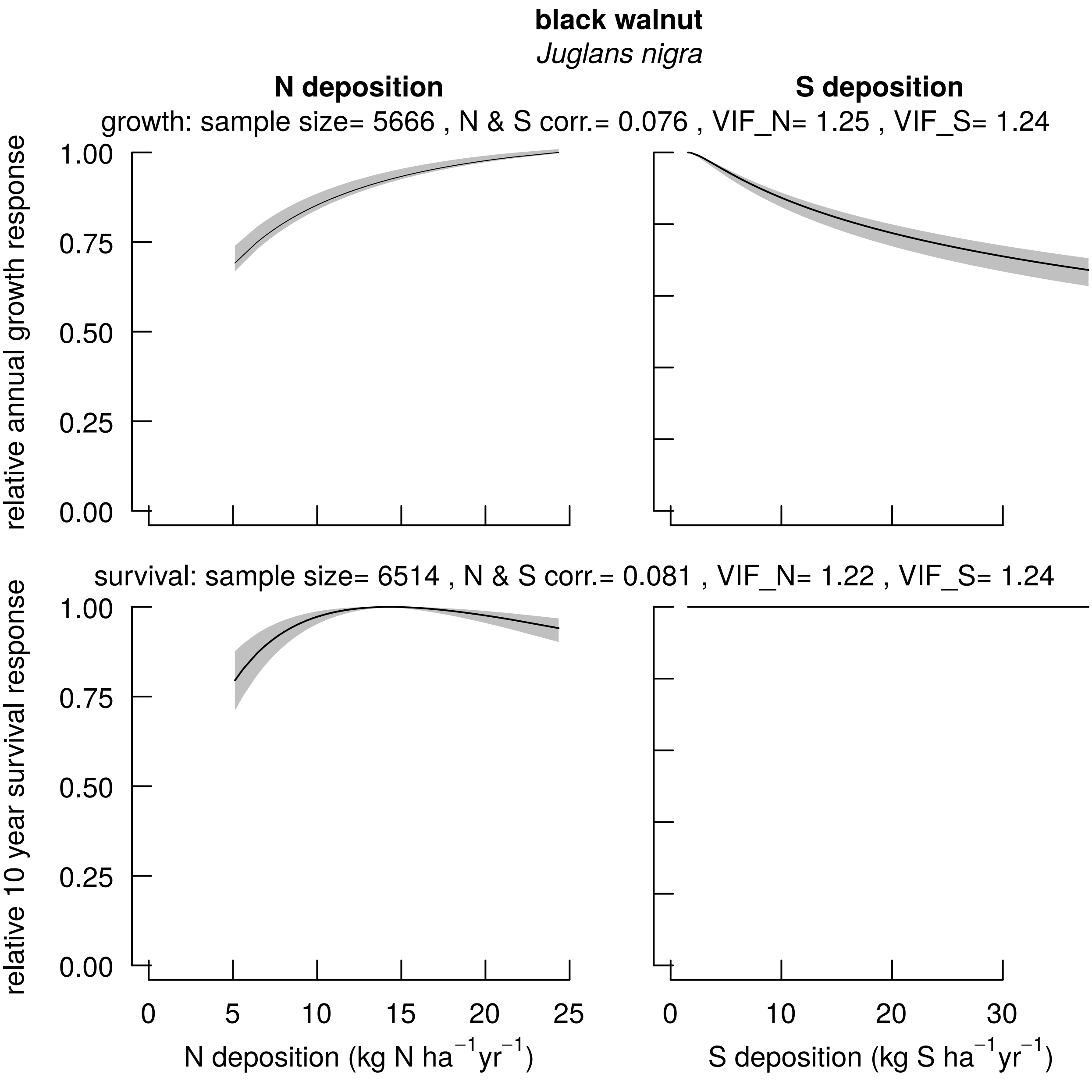

black walnut
Juglans nigra
N deposition
S deposition
growth: sample size= 5666 , N & S corr.= 0.076 , VIF_N= 1.25 , VIF_S= 1.24
relative annual growth response
1.00
0.75
0.50
0.25
0.00
survival: sample size= 6514 , N & S corr.= 0.081 , VIF_N= 1.22 , VIF_S= 1.24
relative 10 year survival response
1.00
0.75
0.50
0.25
0.00
0
5
10
15
20
25
N deposition (kg N ha⁻¹yr⁻¹)
0
10
20
30
S deposition (kg S ha⁻¹yr⁻¹)

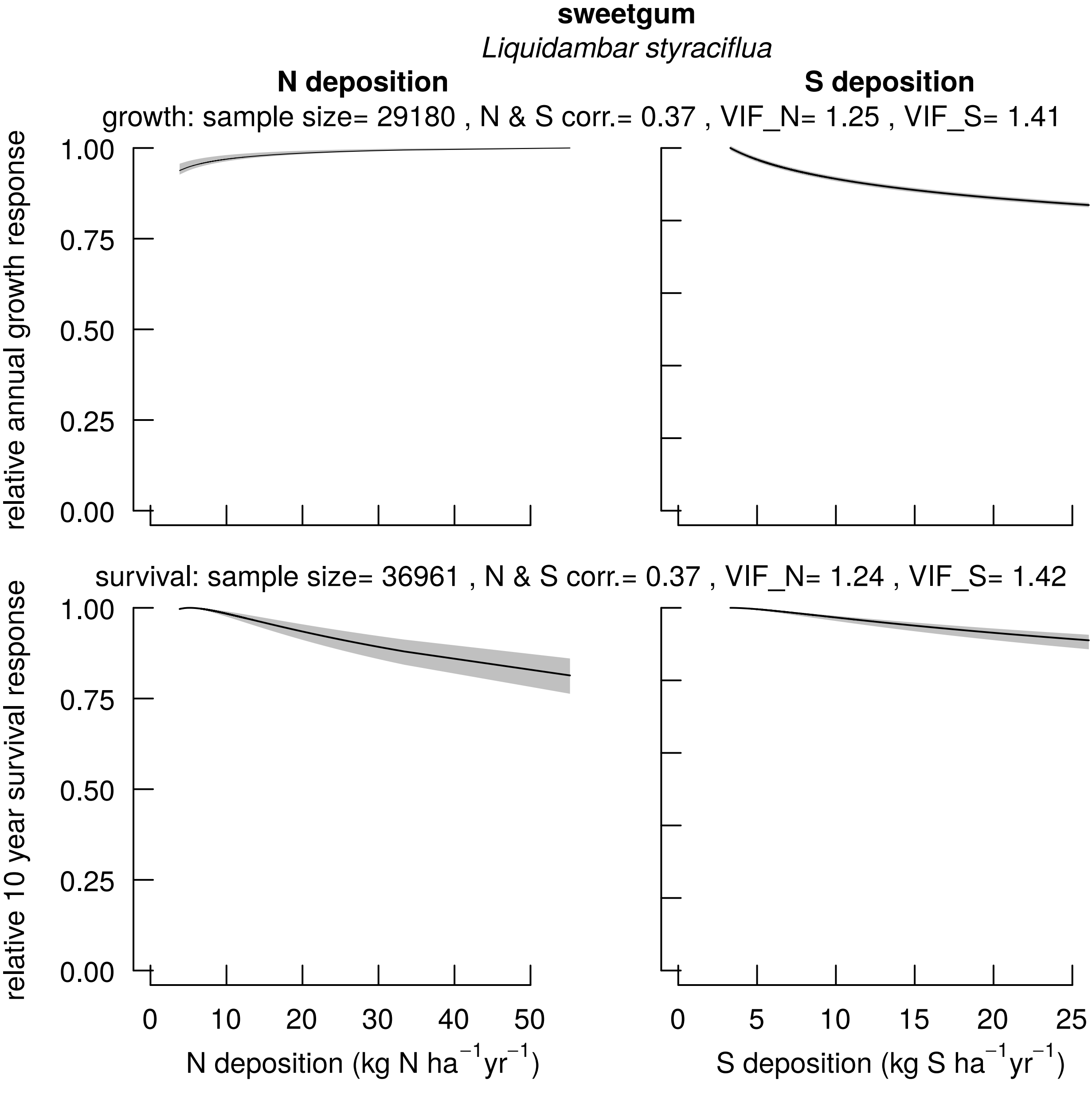
sweetgum
Liquidambar styraciflua
N deposition
S deposition
growth: sample size= 29180 , N & S corr.= 0.37 , VIF_N= 1.25 , VIF_S= 1.41
relative annual growth response
1.00
0.75
0.50
0.25
0.00
survival: sample size= 36961 , N & S corr.= 0.37 , VIF_N= 1.24 , VIF_S= 1.42
relative 10 year survival response
1.00
0.75
0.50
0.25
0.00
0
10
20
30
40
50
N deposition (kg N ha−1yr−1)
0
5
10
15
20
25
S deposition (kg S ha−1yr−1)

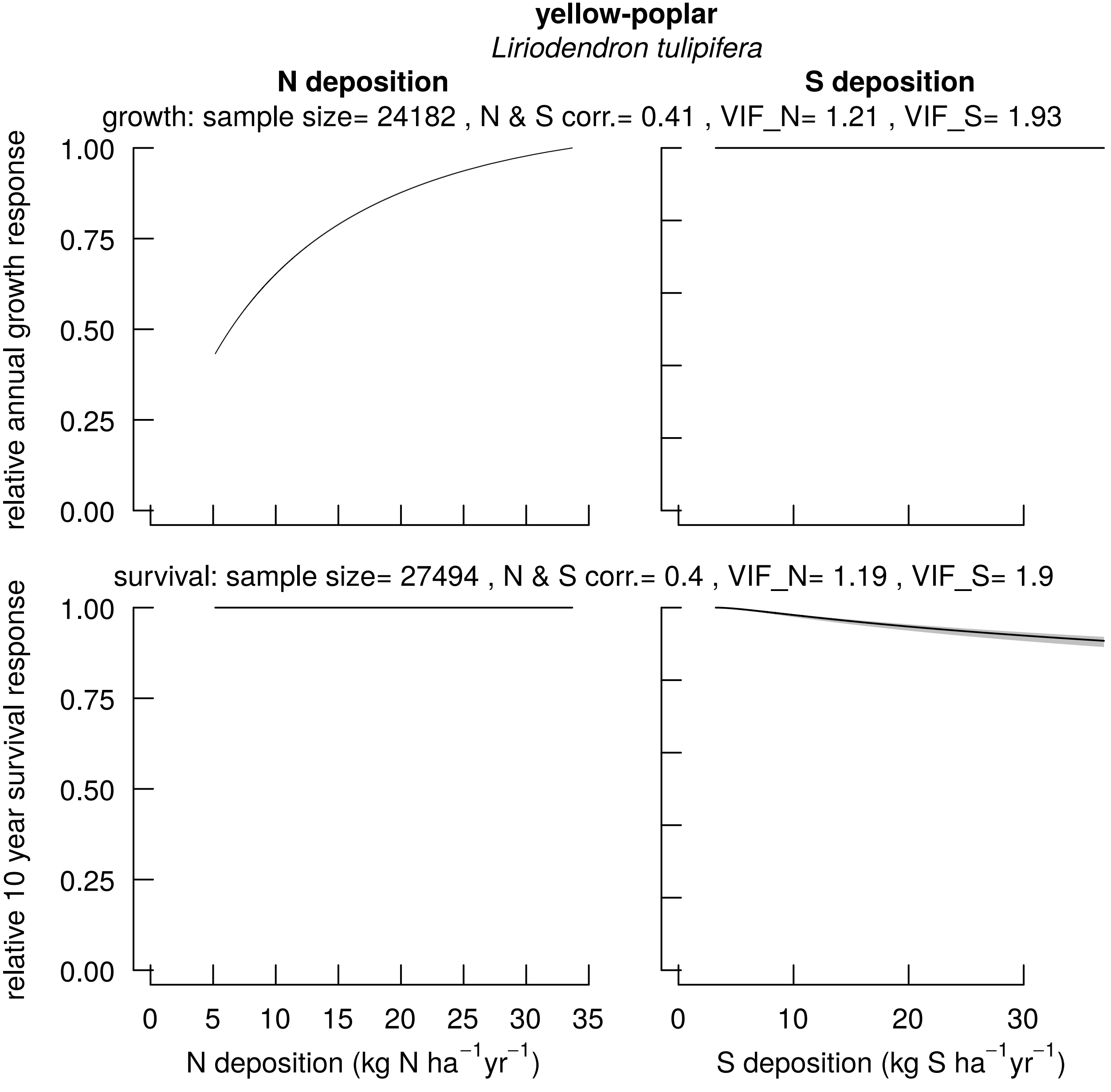

yellow-poplar
Liriodendron tulipifera
N deposition
S deposition
growth: sample size= 24182 , N & S corr.= 0.41 , VIF_N= 1.21 , VIF_S= 1.93
relative annual growth response
1.00
0.75
0.50
0.25
0.00
survival: sample size= 27494 , N & S corr.= 0.4 , VIF_N= 1.19 , VIF_S= 1.9
relative 10 year survival response
1.00
0.75
0.50
0.25
0.00
0 5 10 15 20 25 30 35
0 10 20 30
N deposition (kg N $ha^{-1}yr^{-1}$)
S deposition (kg S $ha^{-1}yr^{-1}$)

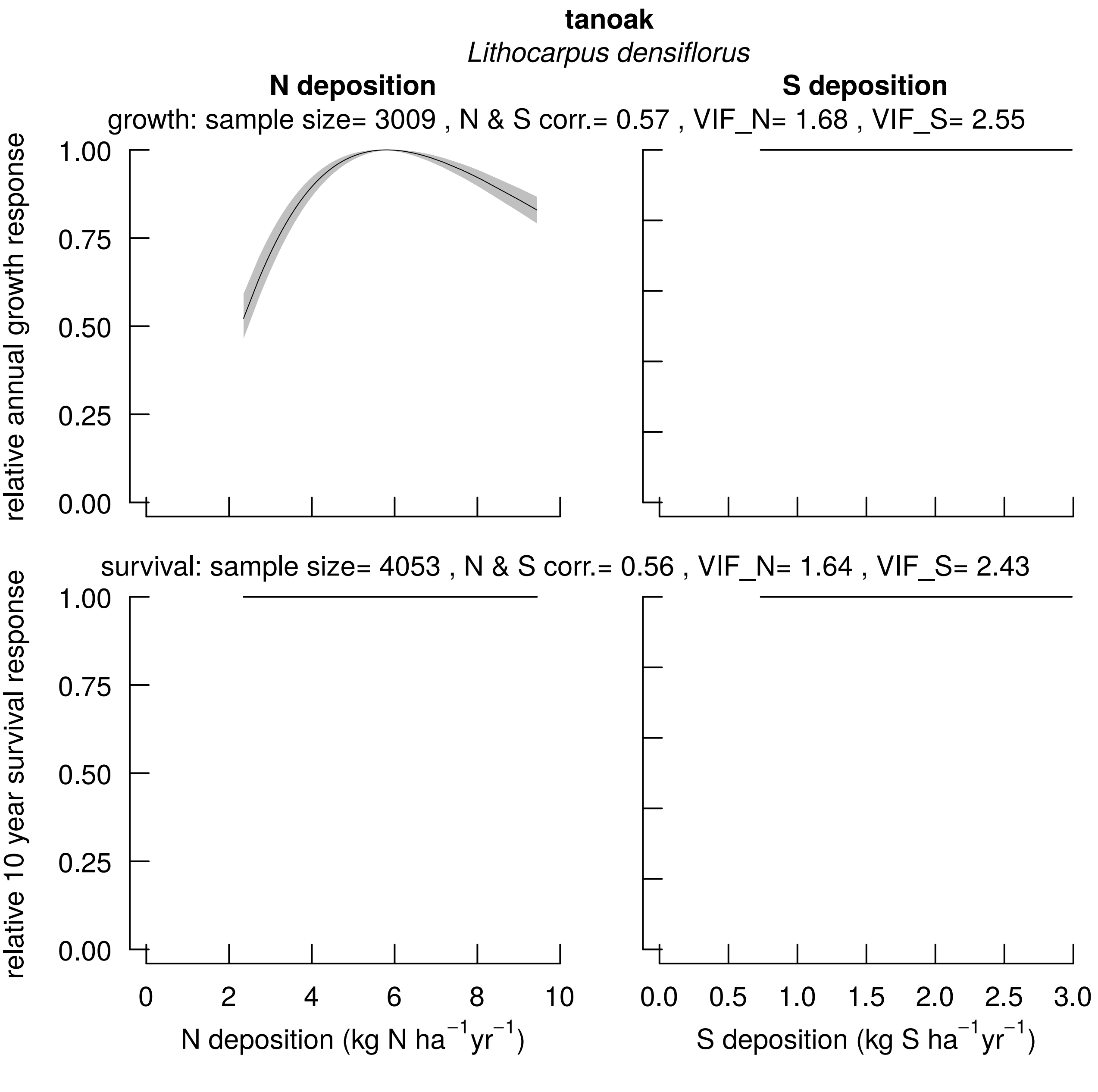
tanoak
Lithocarpus densiflorus
N deposition
S deposition
growth: sample size= 3009 , N & S corr.= 0.57 , VIF_N= 1.68 , VIF_S= 2.55
survival: sample size= 4053 , N & S corr.= 0.56 , VIF_N= 1.64 , VIF_S= 2.43
relative annual growth response
relative 10 year survival response
1.00
0.75
0.50
0.25
0.00
0
2
4
6
8
10
0.0
0.5
1.0
1.5
2.0
2.5
3.0
N deposition (kg N ha⁻¹yr⁻¹)
S deposition (kg S ha⁻¹yr⁻¹)

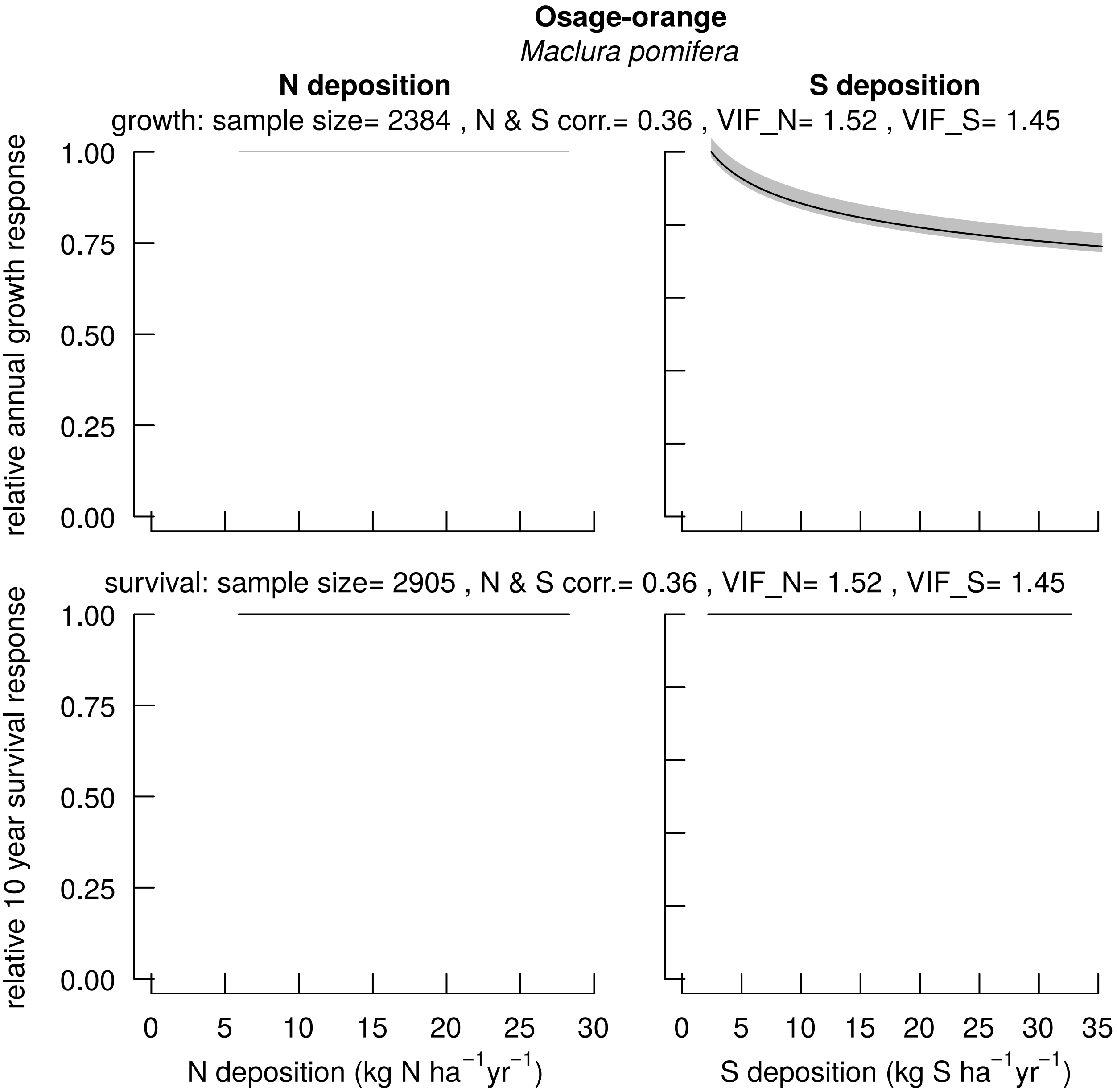

Osage-orange
Maclura pomifera
N deposition
S deposition
growth: sample size= 2384 , N & S corr.= 0.36 , VIF_N= 1.52 , VIF_S= 1.45
relative annual growth response
1.00
0.75
0.50
0.25
0.00
survival: sample size= 2905 , N & S corr.= 0.36 , VIF_N= 1.52 , VIF_S= 1.45
relative 10 year survival response
0 5 10 15 20 25 30
0 5 10 15 20 25 30 35
N deposition (kg N ha−1yr−1)
S deposition (kg S ha−1yr−1)

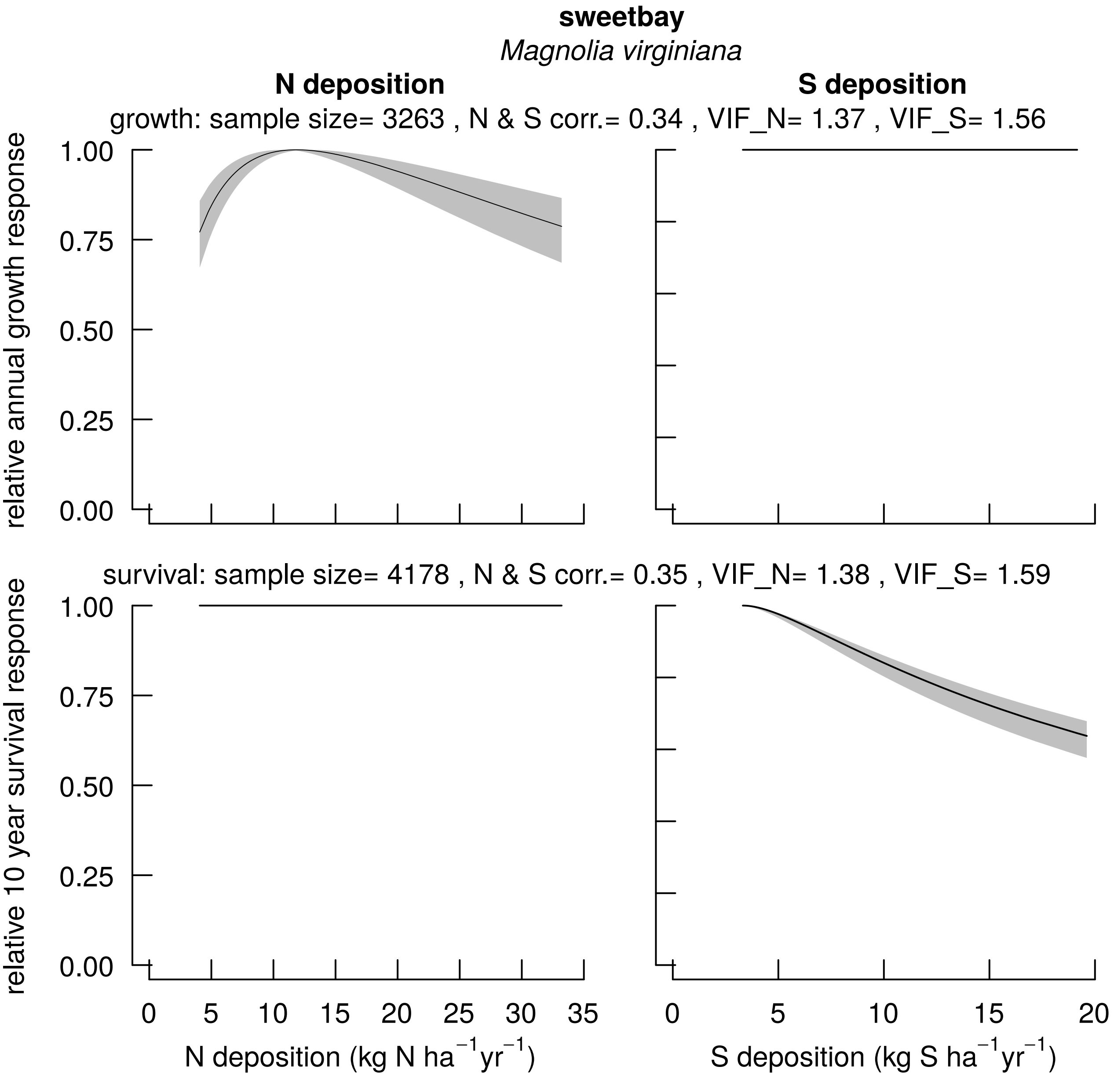
sweetbay
Magnolia virginiana
N deposition
S deposition
growth: sample size= 3263 , N & S corr.= 0.34 , VIF_N= 1.37 , VIF_S= 1.56
relative annual growth response
1.00
0.75
0.50
0.25
0.00
survival: sample size= 4178 , N & S corr.= 0.35 , VIF_N= 1.38 , VIF_S= 1.59
relative 10 year survival response
1.00
0.75
0.50
0.25
0.00
0
5
10
15
20
25
30
35
N deposition (kg N ha^{-1}yr^{-1})
0
5
10
15
20
S deposition (kg S ha^{-1}yr^{-1})

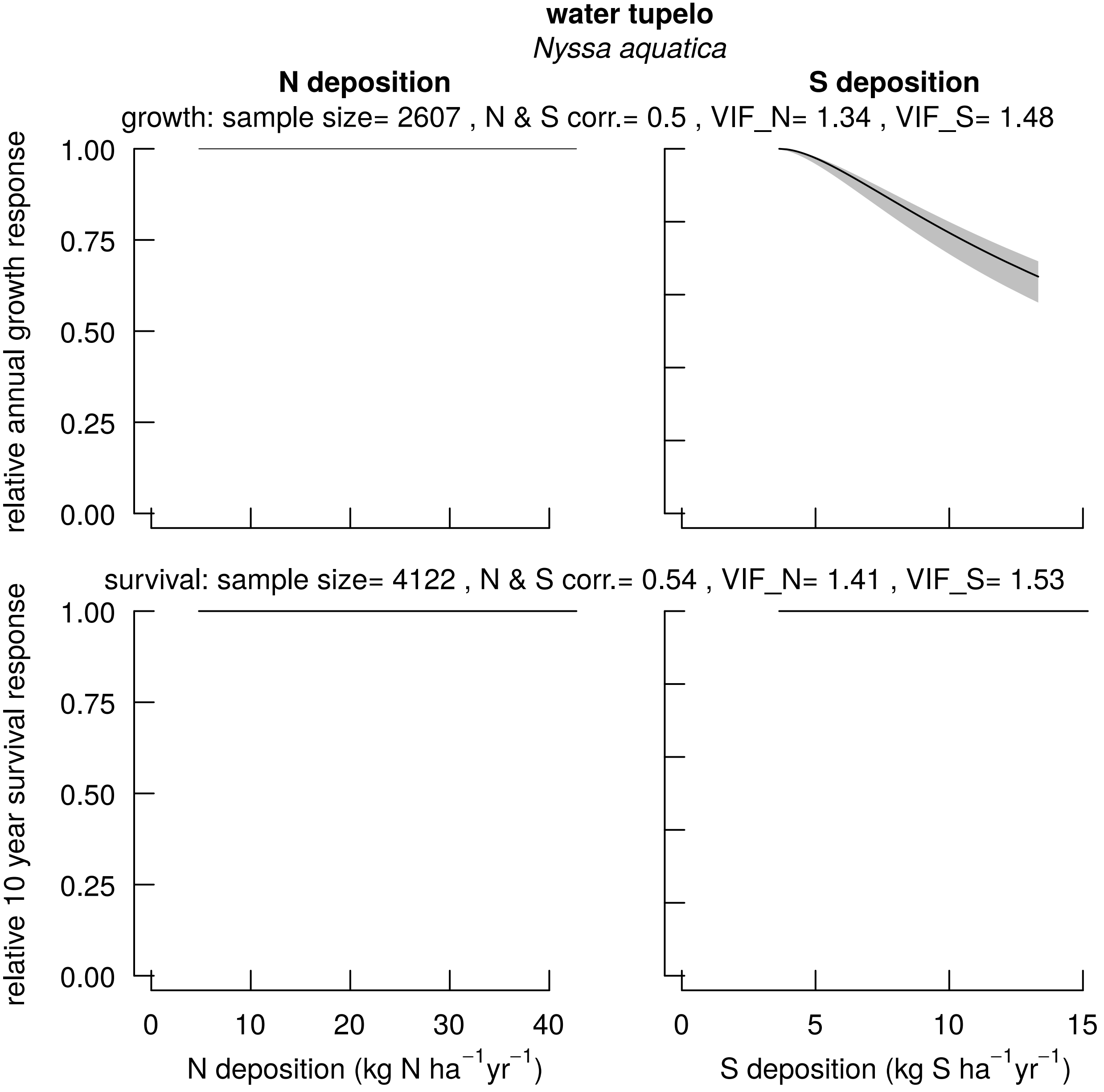

water tupelo
Nyssa aquatica
N deposition
S deposition
growth: sample size= 2607 , N & S corr.= 0.5 , VIF_N= 1.34 , VIF_S= 1.48
relative annual growth response
1.00
0.75
0.50
0.25
0.00
survival: sample size= 4122 , N & S corr.= 0.54 , VIF_N= 1.41 , VIF_S= 1.53
relative 10 year survival response
1.00
0.75
0.50
0.25
0.00
0
10
20
30
40
N deposition (kg N $ha^{-1}yr^{-1}$)
0
5
10
15
S deposition (kg S $ha^{-1}yr^{-1}$)

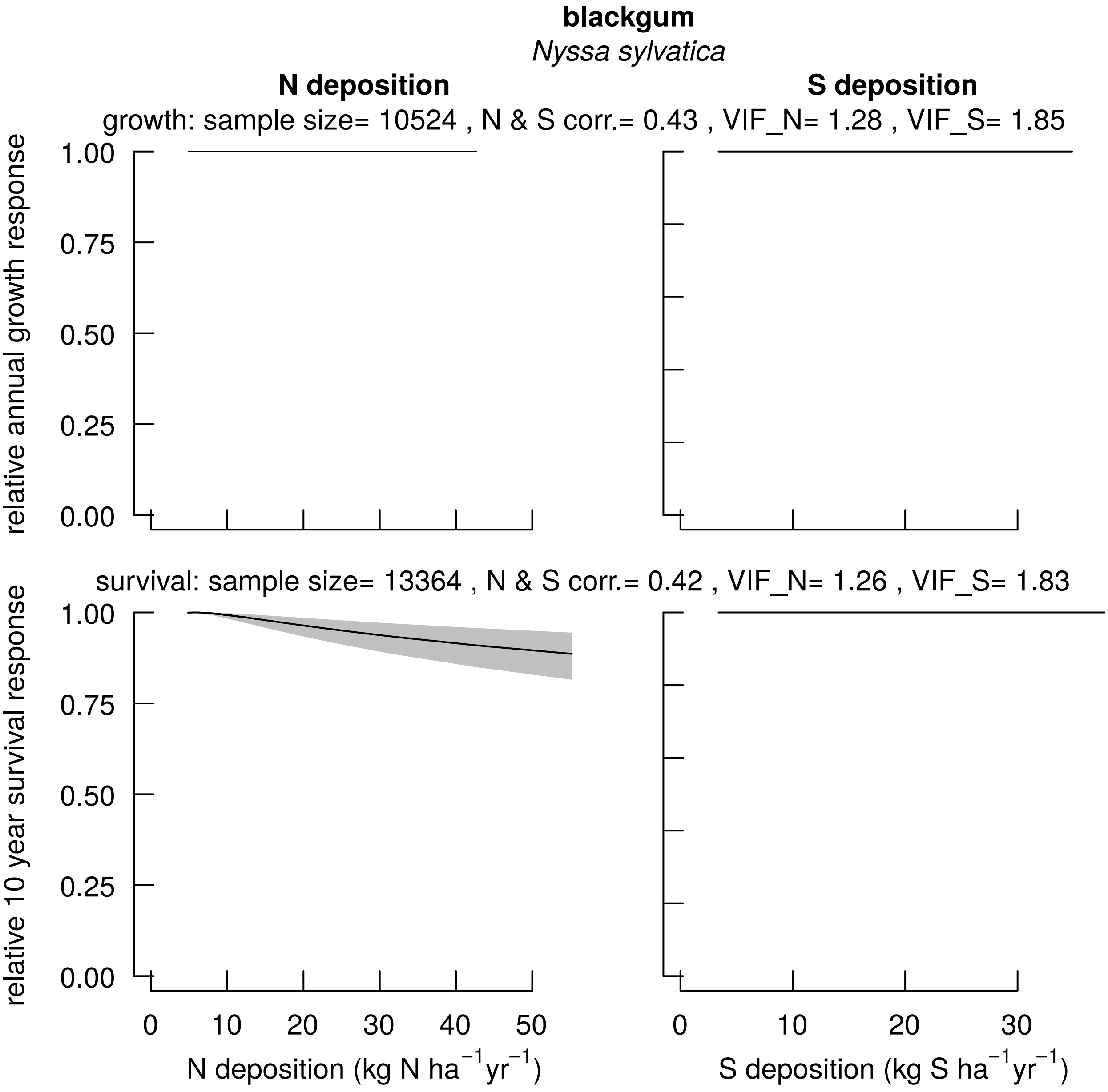

blackgum
Nyssa sylvatica
N deposition
S deposition
growth: sample size= 10524 , N & S corr.= 0.43 , VIF_N= 1.28 , VIF_S= 1.85
relative annual growth response
1.00
0.75
0.50
0.25
0.00
survival: sample size= 13364 , N & S corr.= 0.42 , VIF_N= 1.26 , VIF_S= 1.83
relative 10 year survival response
0
10
20
30
40
50
N deposition (kg N ha−1yr−1)
S deposition (kg S ha−1yr−1)

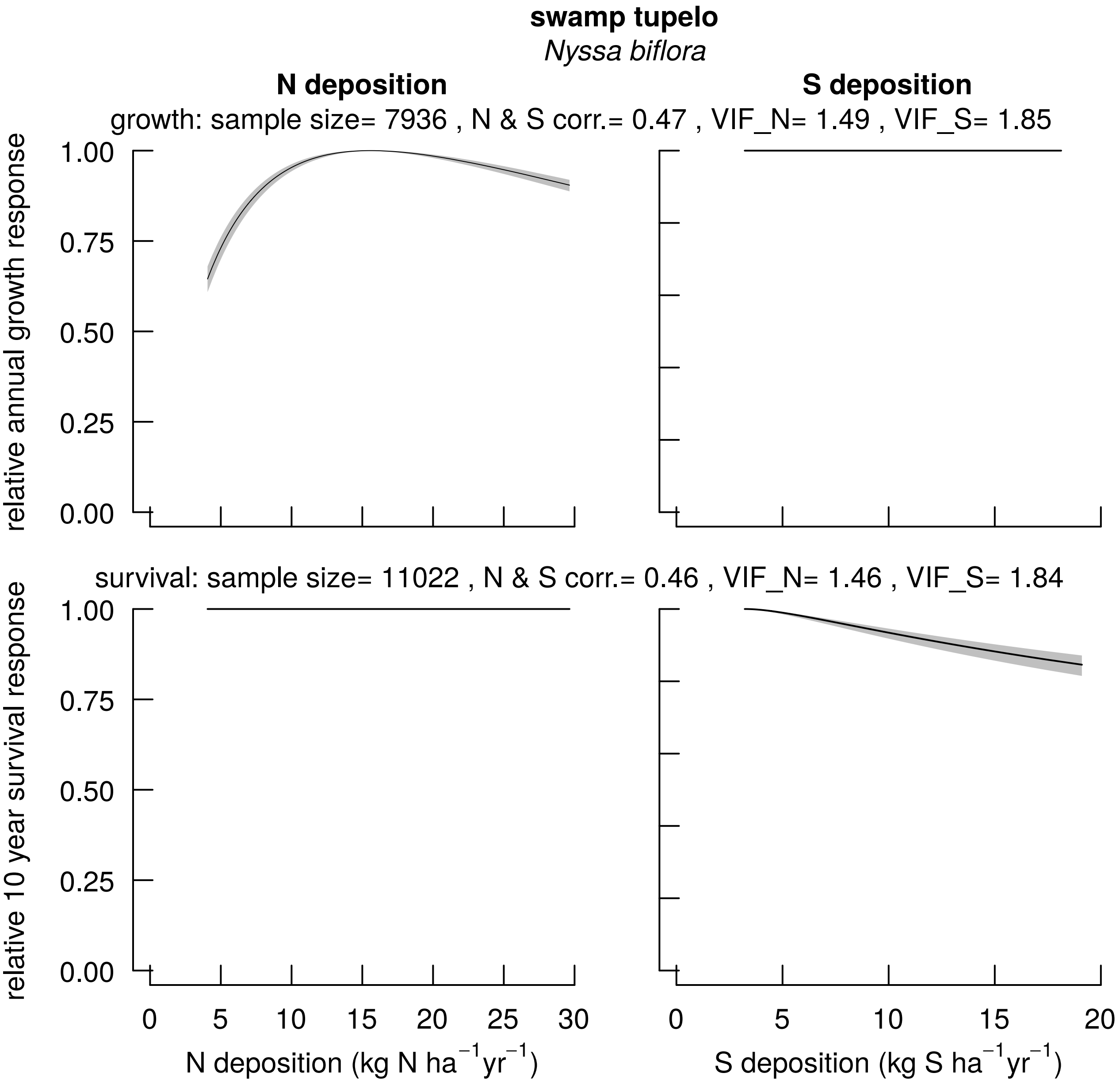
swamp tupelo
Nyssa biflora
N deposition
S deposition
growth: sample size= 7936 , N & S corr.= 0.47 , VIF_N= 1.49 , VIF_S= 1.85
survival: sample size= 11022 , N & S corr.= 0.46 , VIF_N= 1.46 , VIF_S= 1.84
relative annual growth response
relative 10 year survival response
1.00
0.75
0.50
0.25
0.00
0 5 10 15 20 25 30
0 5 10 15 20
N deposition (kg N ha^{-1}yr^{-1})
S deposition (kg S ha^{-1}yr^{-1})

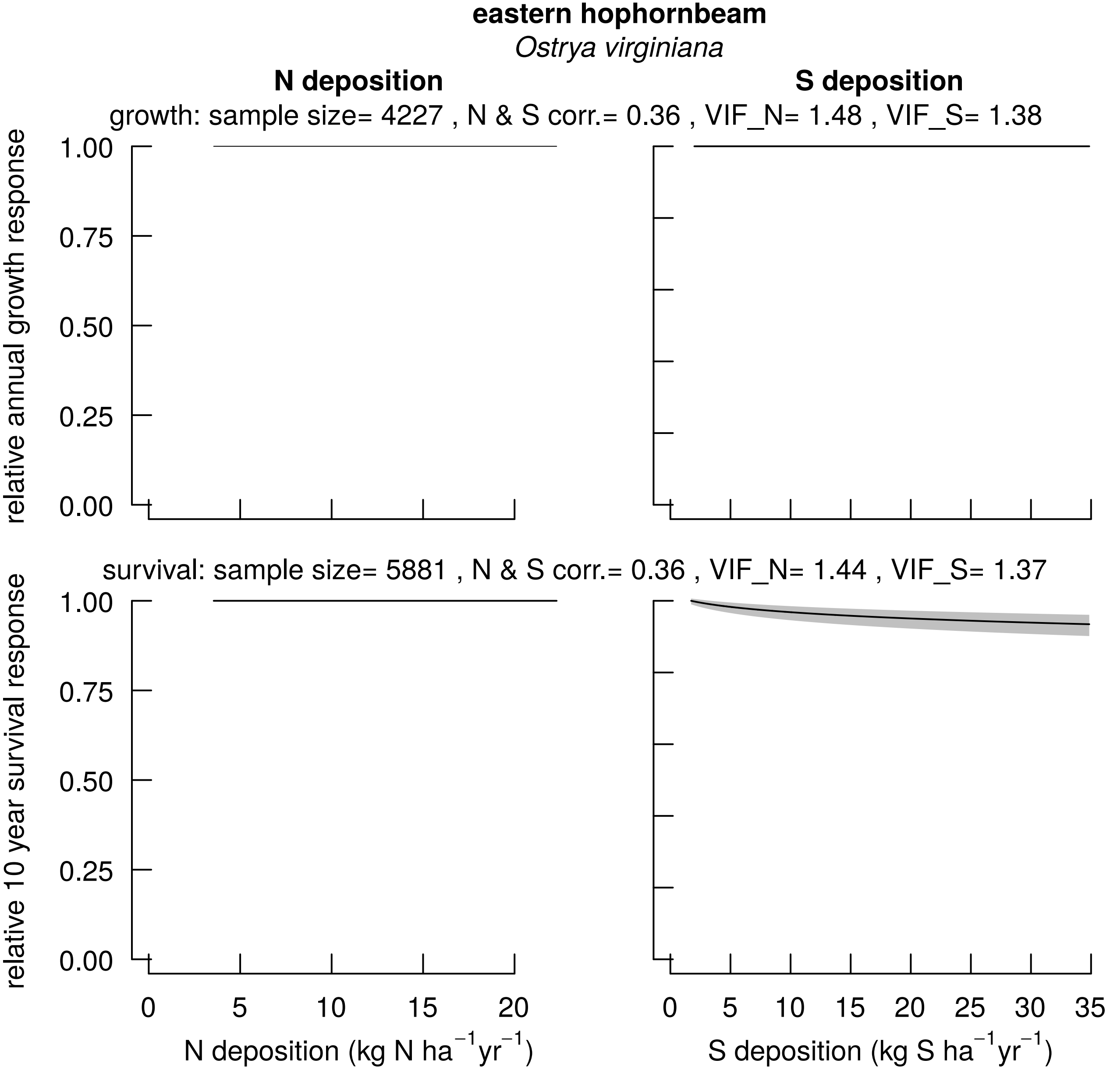

eastern hophornbeam
Ostrya virginiana
N deposition
S deposition
growth: sample size= 4227 , N & S corr.= 0.36 , VIF_N= 1.48 , VIF_S= 1.38
relative annual growth response
1.00
0.75
0.50
0.25
0.00
survival: sample size= 5881 , N & S corr.= 0.36 , VIF_N= 1.44 , VIF_S= 1.37
relative 10 year survival response
1.00
0.75
0.50
0.25
0.00
0 5 10 15 20
0 5 10 15 20 25 30 35
N deposition (kg N ha−1yr−1)
S deposition (kg S ha−1yr−1)

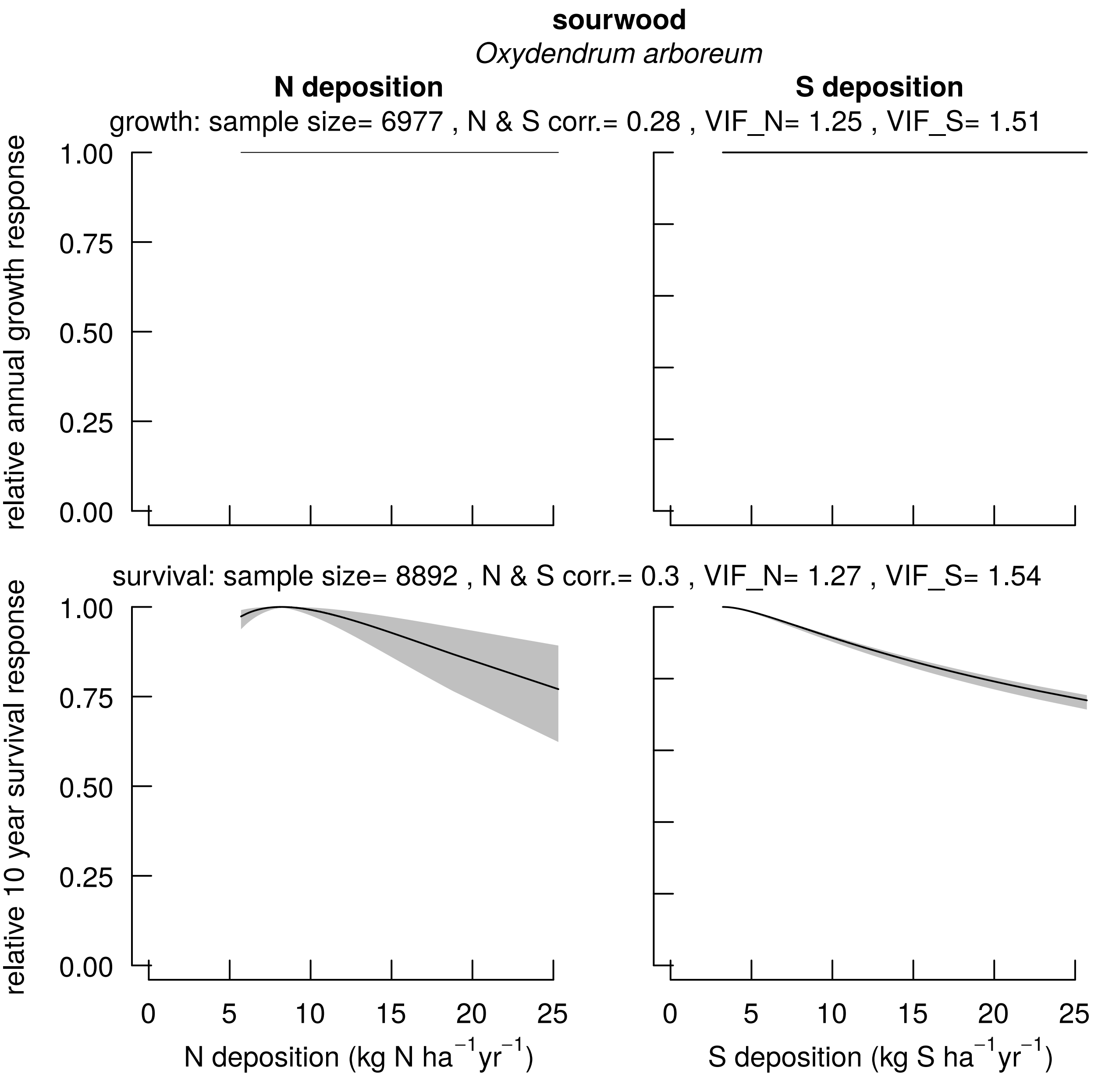

sourwood
Oxydendrum arboreum
N deposition
S deposition
growth: sample size= 6977 , N & S corr.= 0.28 , VIF_N= 1.25 , VIF_S= 1.51
relative annual growth response
1.00
0.75
0.50
0.25
0.00
survival: sample size= 8892 , N & S corr.= 0.3 , VIF_N= 1.27 , VIF_S= 1.54
relative 10 year survival response
0
5
10
15
20
25
N deposition (kg N ha^{-1}yr^{-1})
S deposition (kg S ha^{-1}yr^{-1})

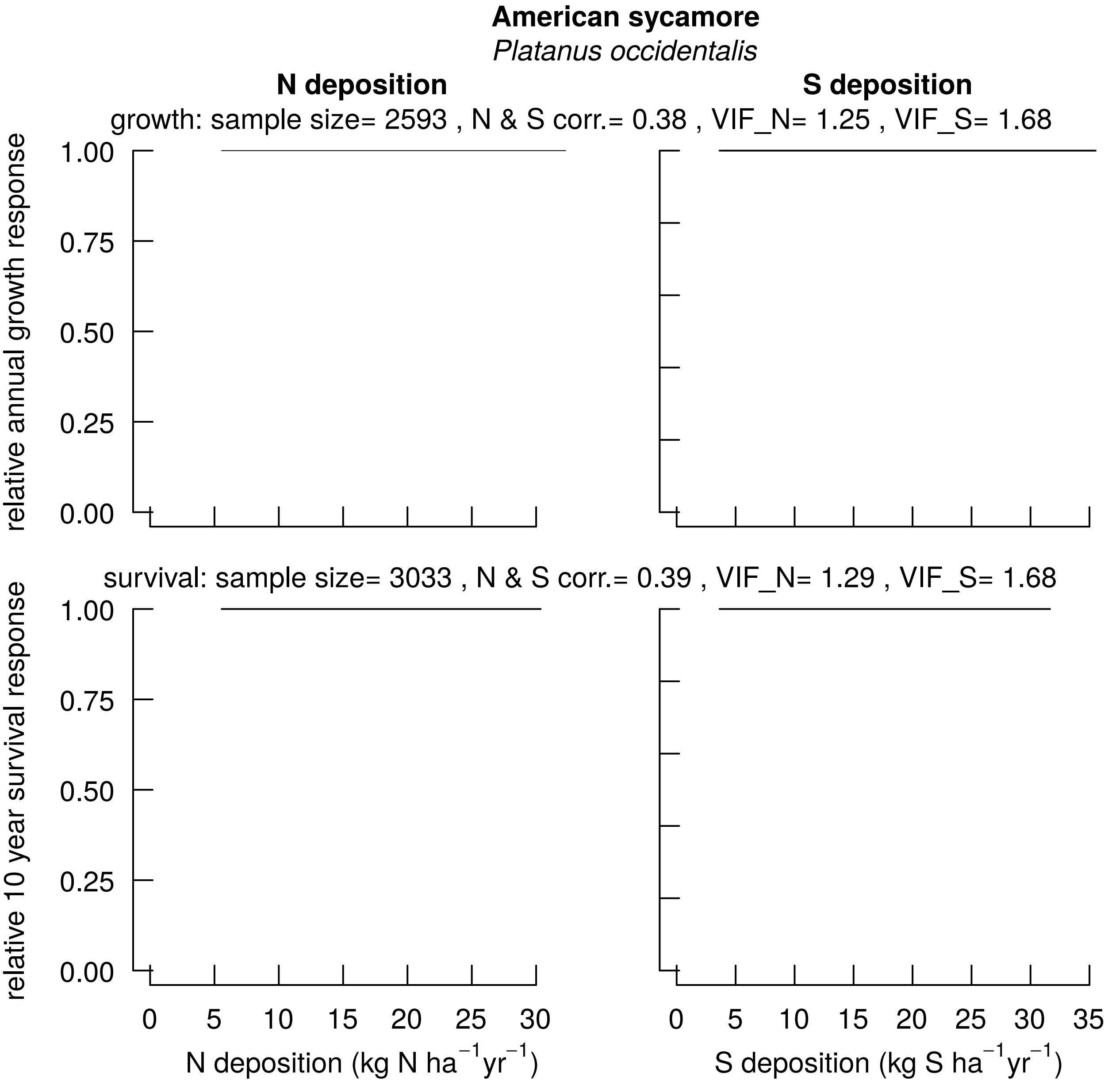

American sycamore
Platanus occidentalis
N deposition
S deposition
growth: sample size= 2593 , N & S corr.= 0.38 , VIF_N= 1.25 , VIF_S= 1.68
relative annual growth response
1.00
0.75
0.50
0.25
0.00
survival: sample size= 3033 , N & S corr.= 0.39 , VIF_N= 1.29 , VIF_S= 1.68
relative 10 year survival response
1.00
0.75
0.50
0.25
0.00
0 5 10 15 20 25 30
N deposition (kg N ha^{-1}yr^{-1})
0 5 10 15 20 25 30 35
S deposition (kg S ha^{-1}yr^{-1})

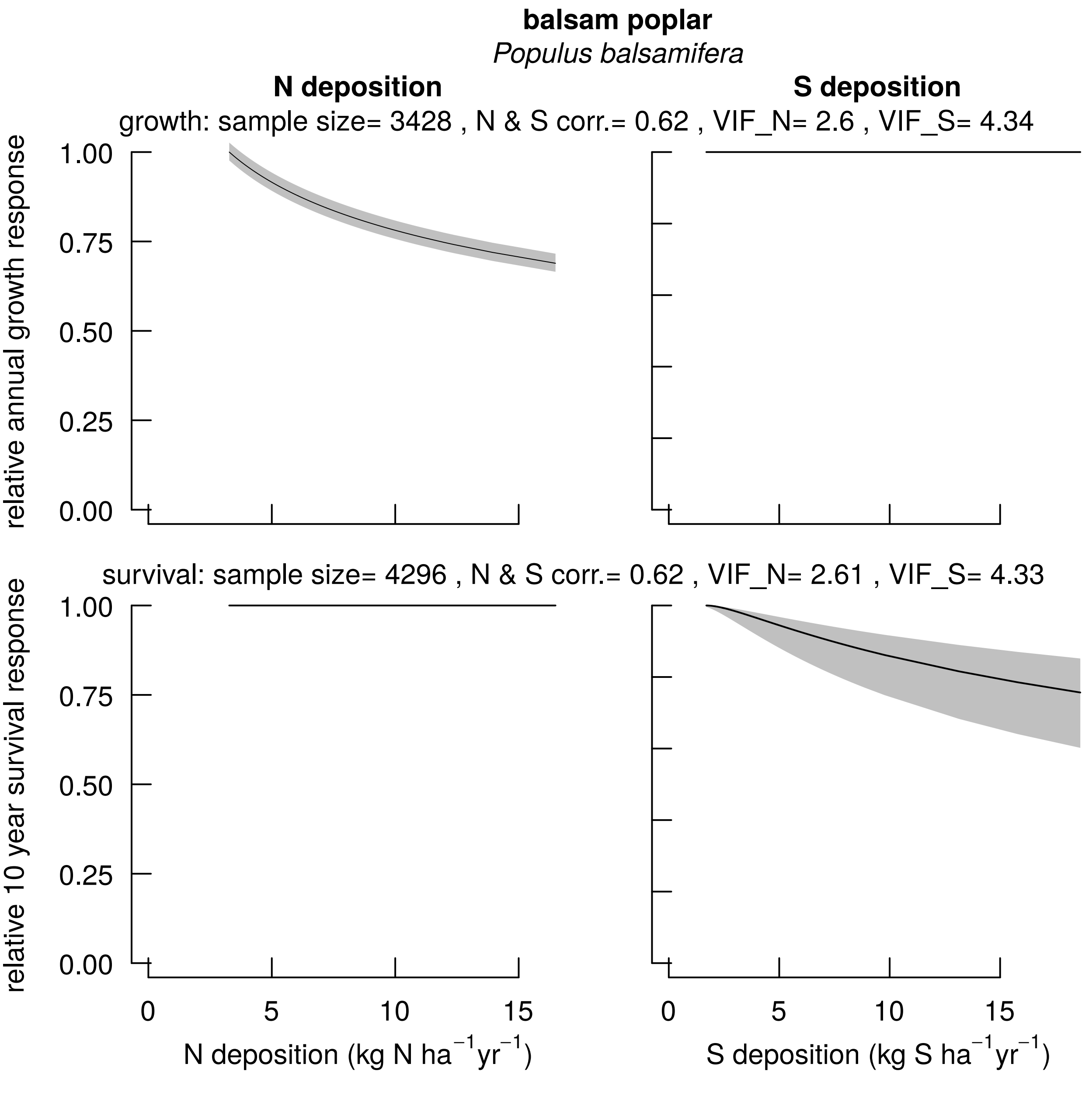

balsam poplar
Populus balsamifera
N deposition
S deposition
growth: sample size= 3428 , N & S corr.= 0.62 , VIF_N= 2.6 , VIF_S= 4.34
survival: sample size= 4296 , N & S corr.= 0.62 , VIF_N= 2.61 , VIF_S= 4.33
relative annual growth response
relative 10 year survival response
1.00
0.75
0.50
0.25
0.00
0
5
10
15
N deposition (kg N ha^{-1}yr^{-1})
S deposition (kg S ha^{-1}yr^{-1})

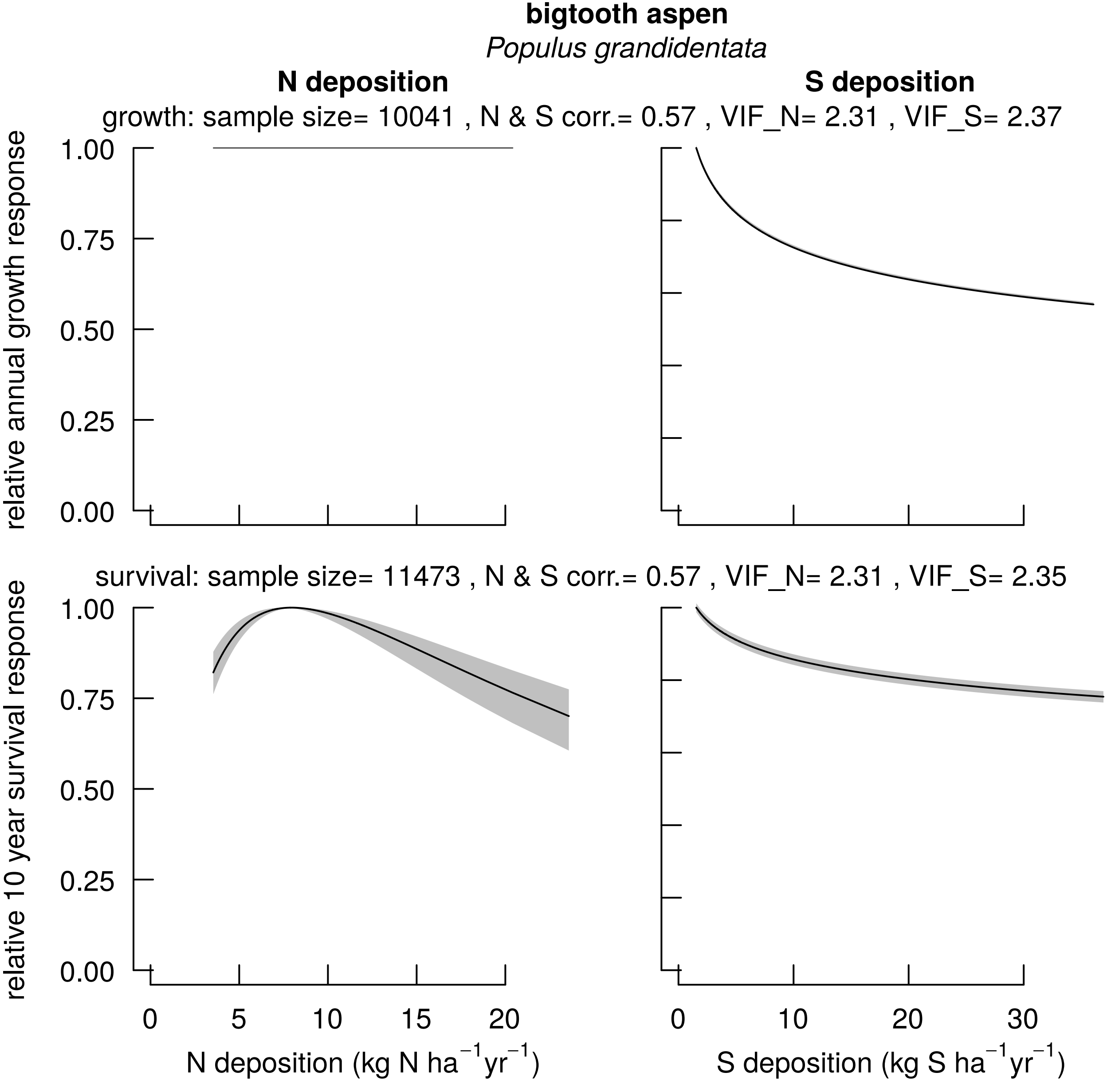
bigtooth aspen
Populus grandidentata
N deposition
S deposition
growth: sample size= 10041 , N & S corr.= 0.57 , VIF_N= 2.31 , VIF_S= 2.37
survival: sample size= 11473 , N & S corr.= 0.57 , VIF_N= 2.31 , VIF_S= 2.35
relative annual growth response
relative 10 year survival response
1.00
0.75
0.50
0.25
0.00
0
5
10
15
20
30
N deposition (kg N ha^{-1}yr^{-1})
S deposition (kg S ha^{-1}yr^{-1})

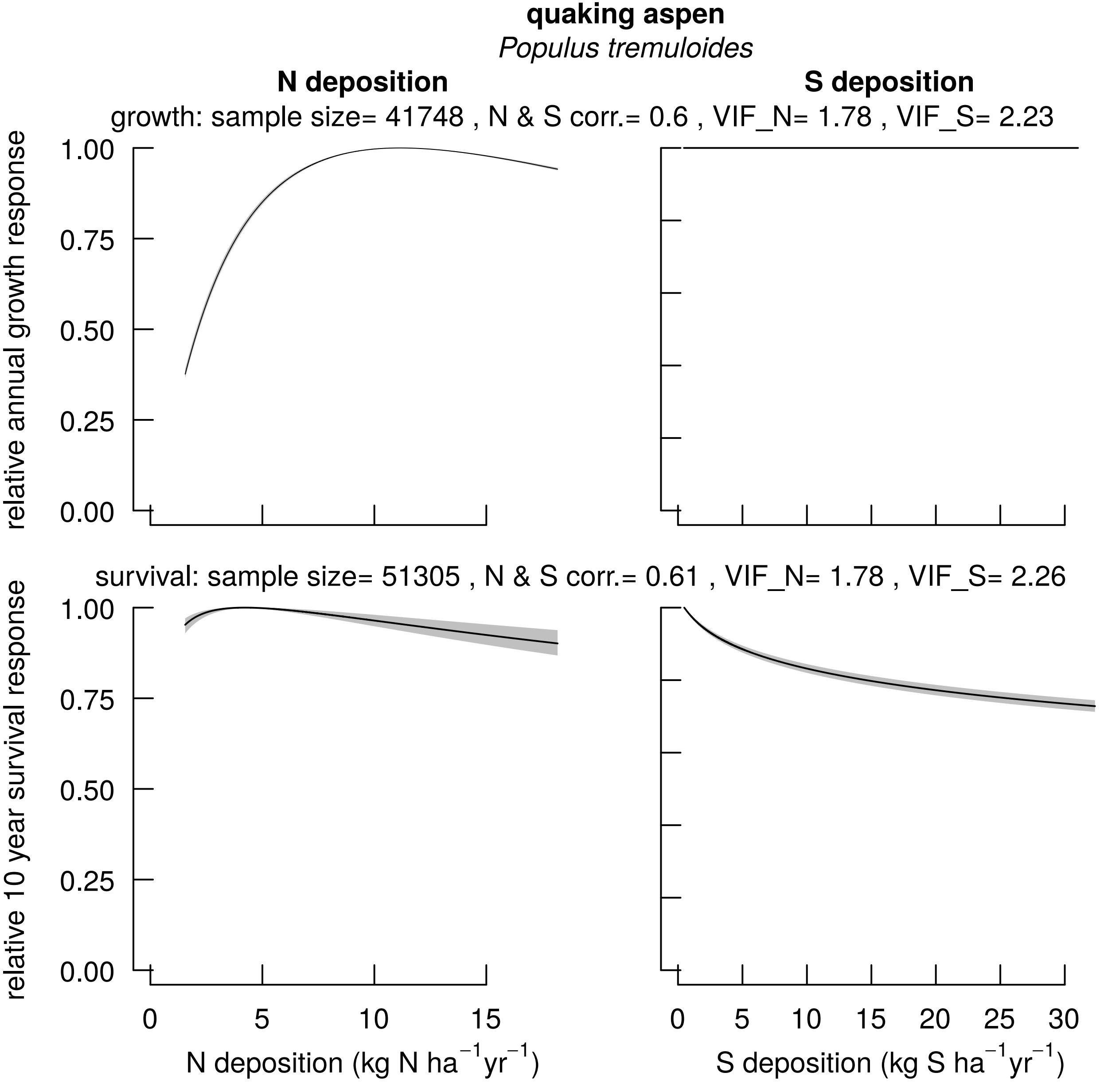
quaking aspen
Populus tremuloides
N deposition
S deposition
growth: sample size= 41748 , N & S corr.= 0.6 , VIF_N= 1.78 , VIF_S= 2.23
survival: sample size= 51305 , N & S corr.= 0.61 , VIF_N= 1.78 , VIF_S= 2.26
relative annual growth response
relative 10 year survival response
1.00
0.75
0.50
0.25
0.00
0
5
10
15
20
25
30
N deposition (kg N ha^{-1}yr^{-1})
S deposition (kg S ha^{-1}yr^{-1})

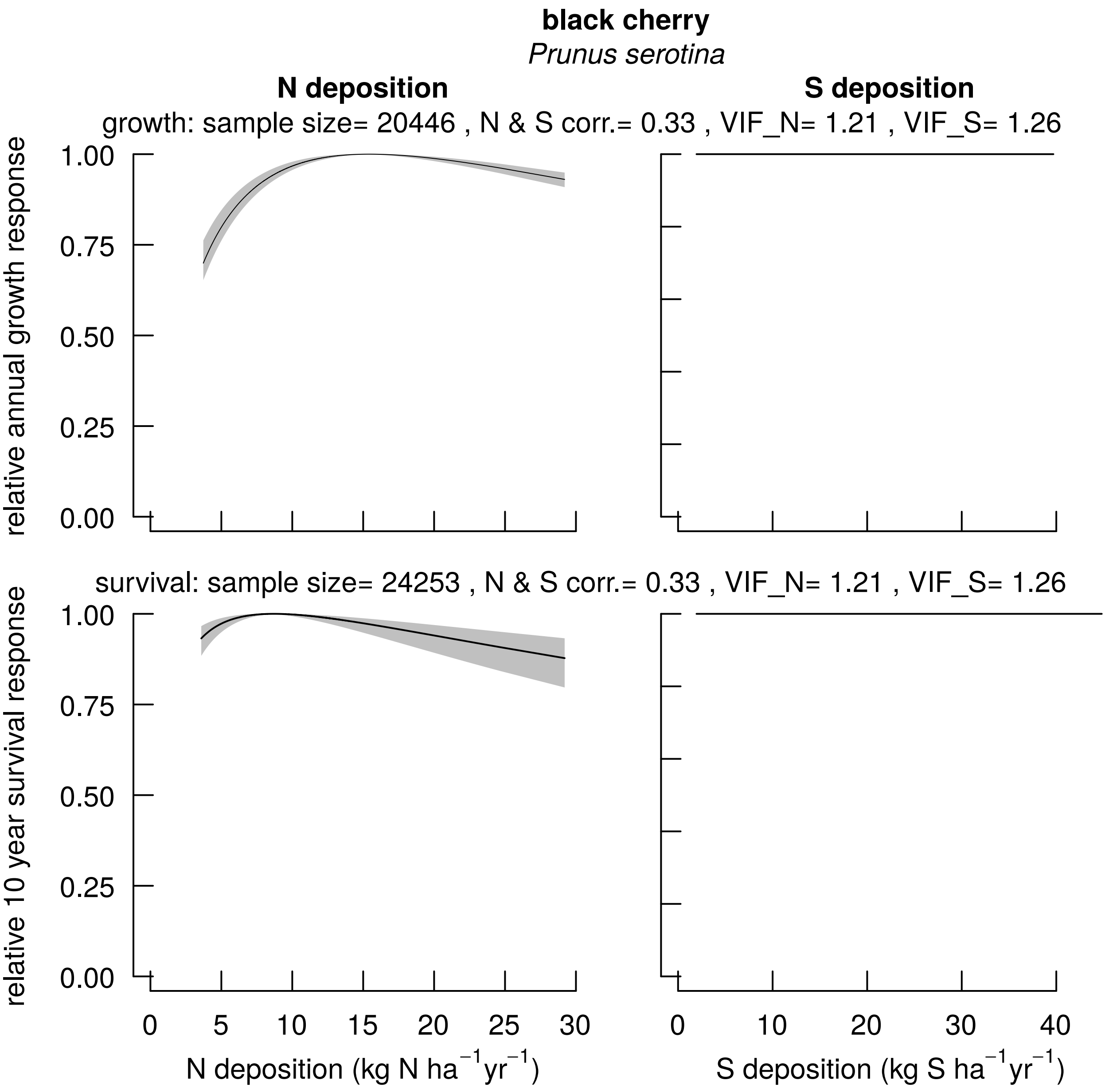

black cherry
Prunus serotina
N deposition
S deposition
growth: sample size= 20446 , N & S corr.= 0.33 , VIF_N= 1.21 , VIF_S= 1.26
relative annual growth response
1.00
0.75
0.50
0.25
0.00
survival: sample size= 24253 , N & S corr.= 0.33 , VIF_N= 1.21 , VIF_S= 1.26
relative 10 year survival response
1.00
0.75
0.50
0.25
0.00
0
5
10
15
20
25
30
0
10
20
30
40
N deposition (kg N ha^{-1}yr^{-1})
S deposition (kg S ha^{-1}yr^{-1})

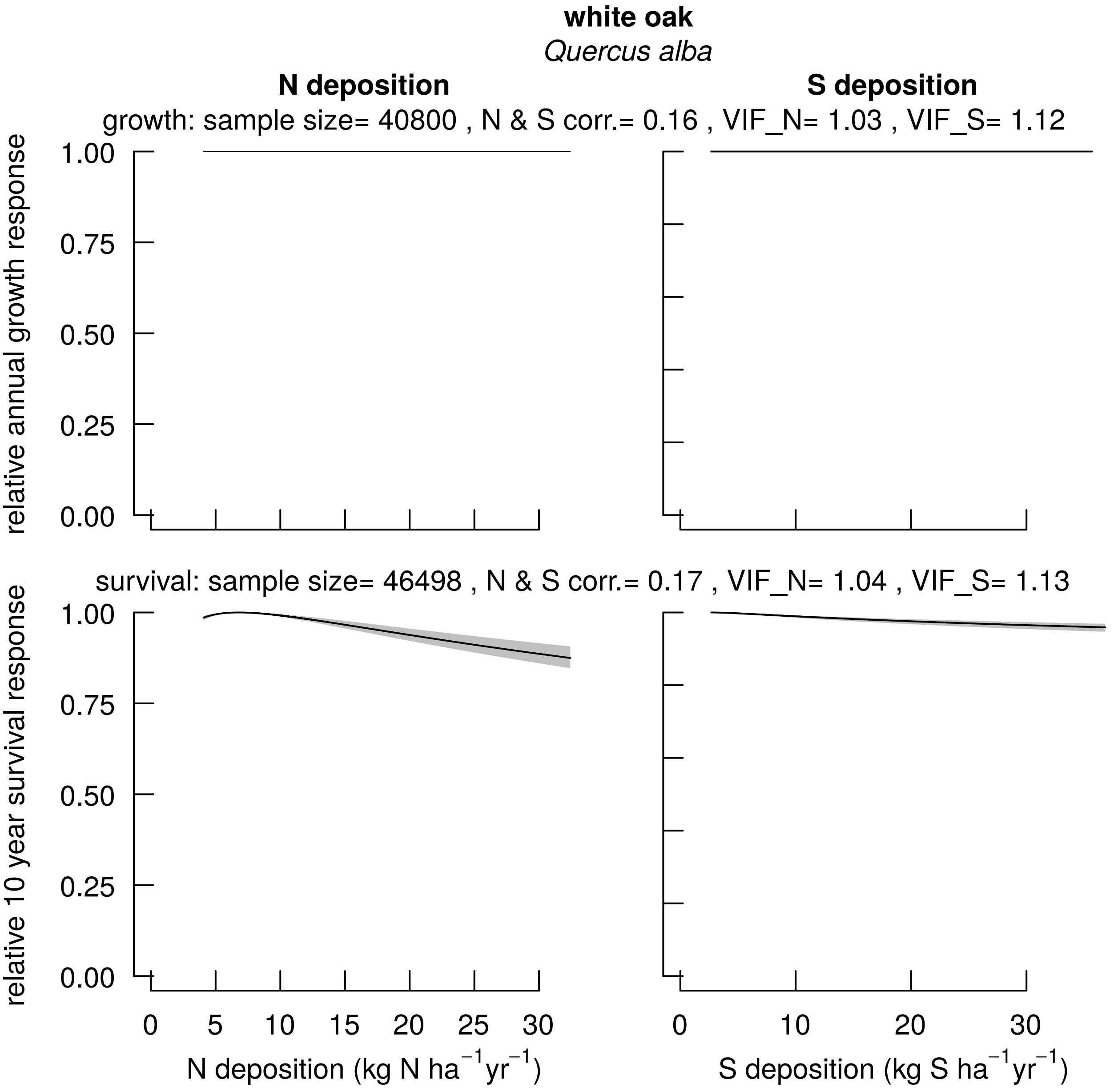

white oak
Quercus alba
N deposition
S deposition
growth: sample size= 40800 , N & S corr.= 0.16 , VIF_N= 1.03 , VIF_S= 1.12
relative annual growth response
1.00
0.75
0.50
0.25
0.00
survival: sample size= 46498 , N & S corr.= 0.17 , VIF_N= 1.04 , VIF_S= 1.13
relative 10 year survival response
1.00
0.75
0.50
0.25
0.00
0
5
10
15
20
25
30
N deposition (kg N ha^{-1}yr^{-1})
0
10
20
30
S deposition (kg S ha^{-1}yr^{-1})

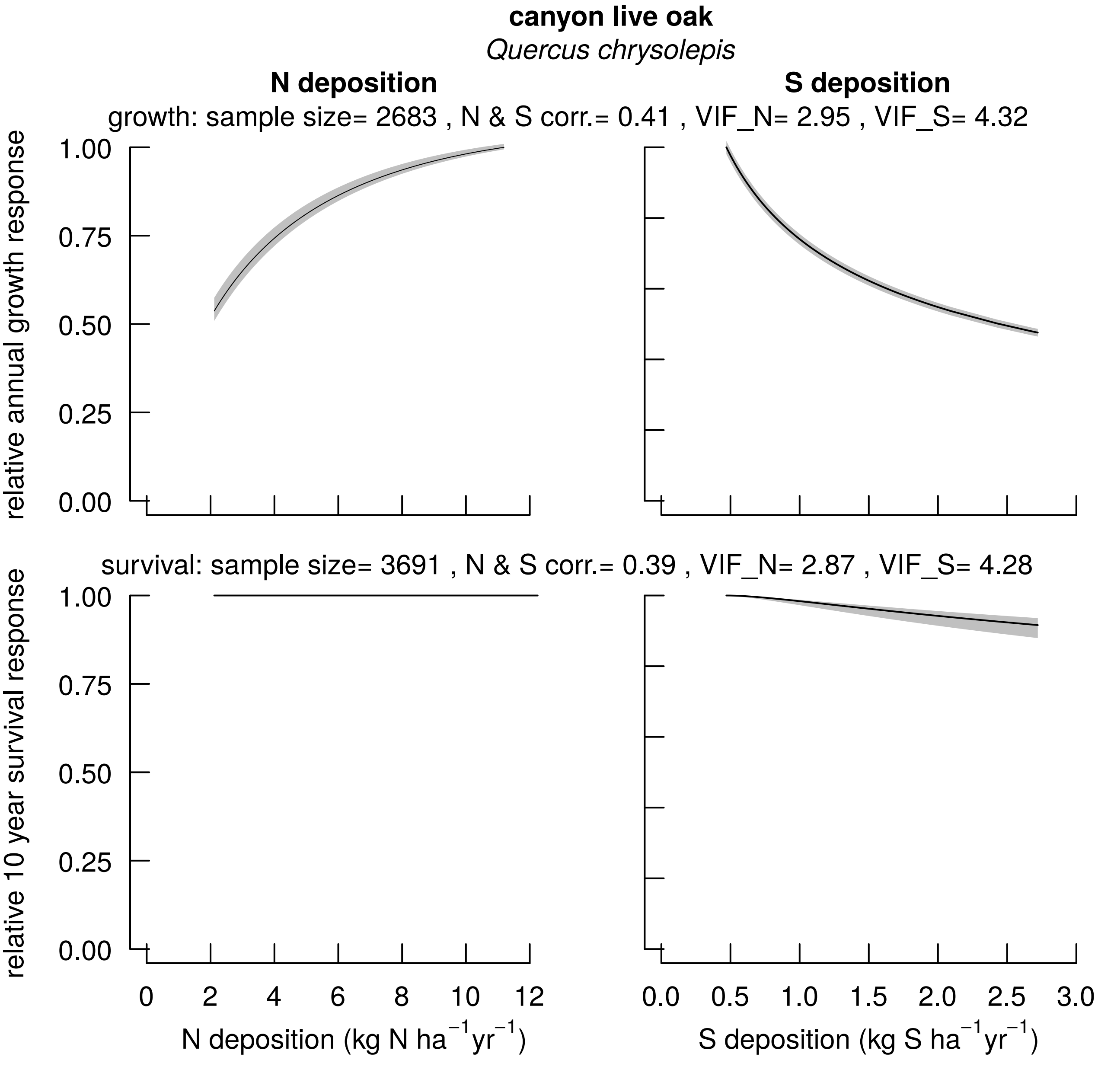

canyon live oak
Quercus chrysolepis
N deposition
S deposition
growth: sample size= 2683 , N & S corr.= 0.41 , VIF_N= 2.95 , VIF_S= 4.32
relative annual growth response
1.00
0.75
0.50
0.25
0.00
survival: sample size= 3691 , N & S corr.= 0.39 , VIF_N= 2.87 , VIF_S= 4.28
relative 10 year survival response
1.00
0.75
0.50
0.25
0.00
0 2 4 6 8 10 12
N deposition (kg N ha^{-1}yr^{-1})
0.0 0.5 1.0 1.5 2.0 2.5 3.0
S deposition (kg S ha^{-1}yr^{-1})

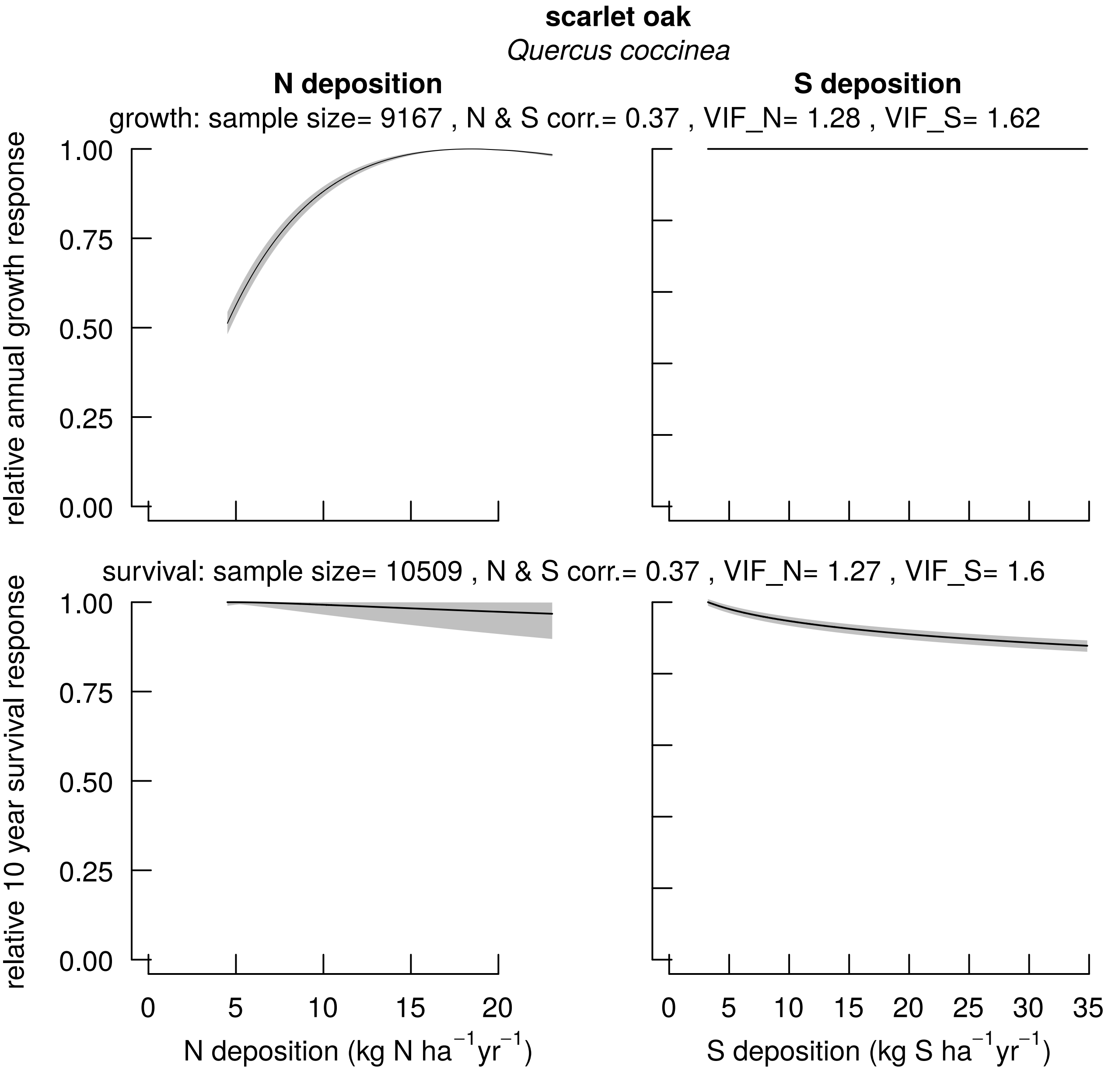

scarlet oak
Quercus coccinea
N deposition
S deposition
growth: sample size= 9167 , N & S corr.= 0.37 , VIF_N= 1.28 , VIF_S= 1.62
relative annual growth response
1.00
0.75
0.50
0.25
0.00
survival: sample size= 10509 , N & S corr.= 0.37 , VIF_N= 1.27 , VIF_S= 1.6
relative 10 year survival response
1.00
0.75
0.50
0.25
0.00
0 5 10 15 20
0 5 10 15 20 25 30 35
N deposition (kg N $ha^{-1}yr^{-1}$)
S deposition (kg S $ha^{-1}yr^{-1}$)

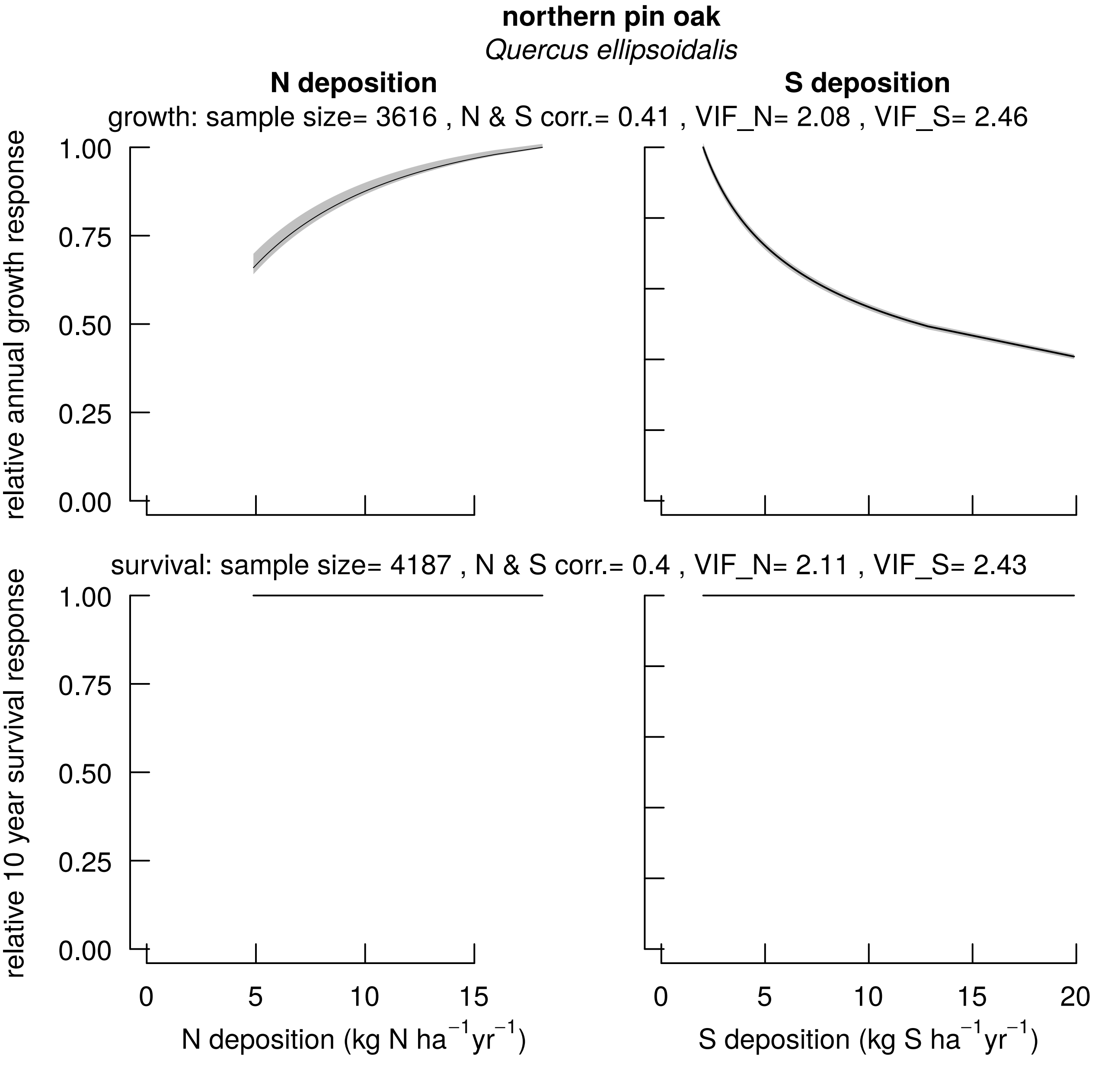

northern pin oak
Quercus ellipsoidalis
N deposition
S deposition
growth: sample size= 3616 , N & S corr.= 0.41 , VIF_N= 2.08 , VIF_S= 2.46
relative annual growth response
1.00
0.75
0.50
0.25
0.00
survival: sample size= 4187 , N & S corr.= 0.4 , VIF_N= 2.11 , VIF_S= 2.43
relative 10 year survival response
1.00
0.75
0.50
0.25
0.00
0
5
10
15
N deposition (kg N ha^{-1}yr^{-1})
0
5
10
15
20
S deposition (kg S ha^{-1}yr^{-1})

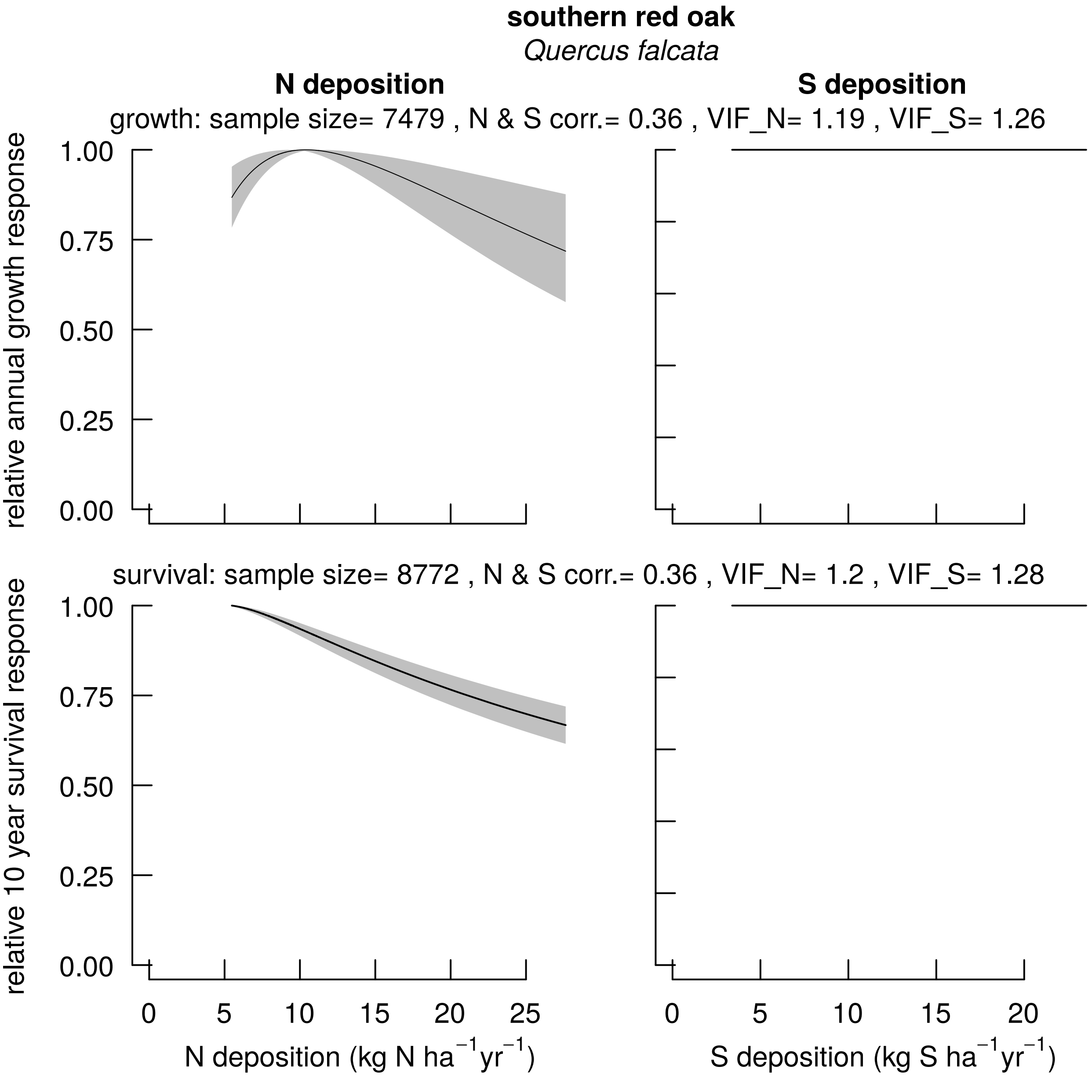
southern red oak
Quercus falcata
N deposition
S deposition
growth: sample size= 7479 , N & S corr.= 0.36 , VIF_N= 1.19 , VIF_S= 1.26
relative annual growth response
1.00
0.75
0.50
0.25
0.00
survival: sample size= 8772 , N & S corr.= 0.36 , VIF_N= 1.2 , VIF_S= 1.28
relative 10 year survival response
1.00
0.75
0.50
0.25
0.00
0
5
10
15
20
25
N deposition (kg N ha^{-1}yr^{-1})
0
5
10
15
20
S deposition (kg S ha^{-1}yr^{-1})

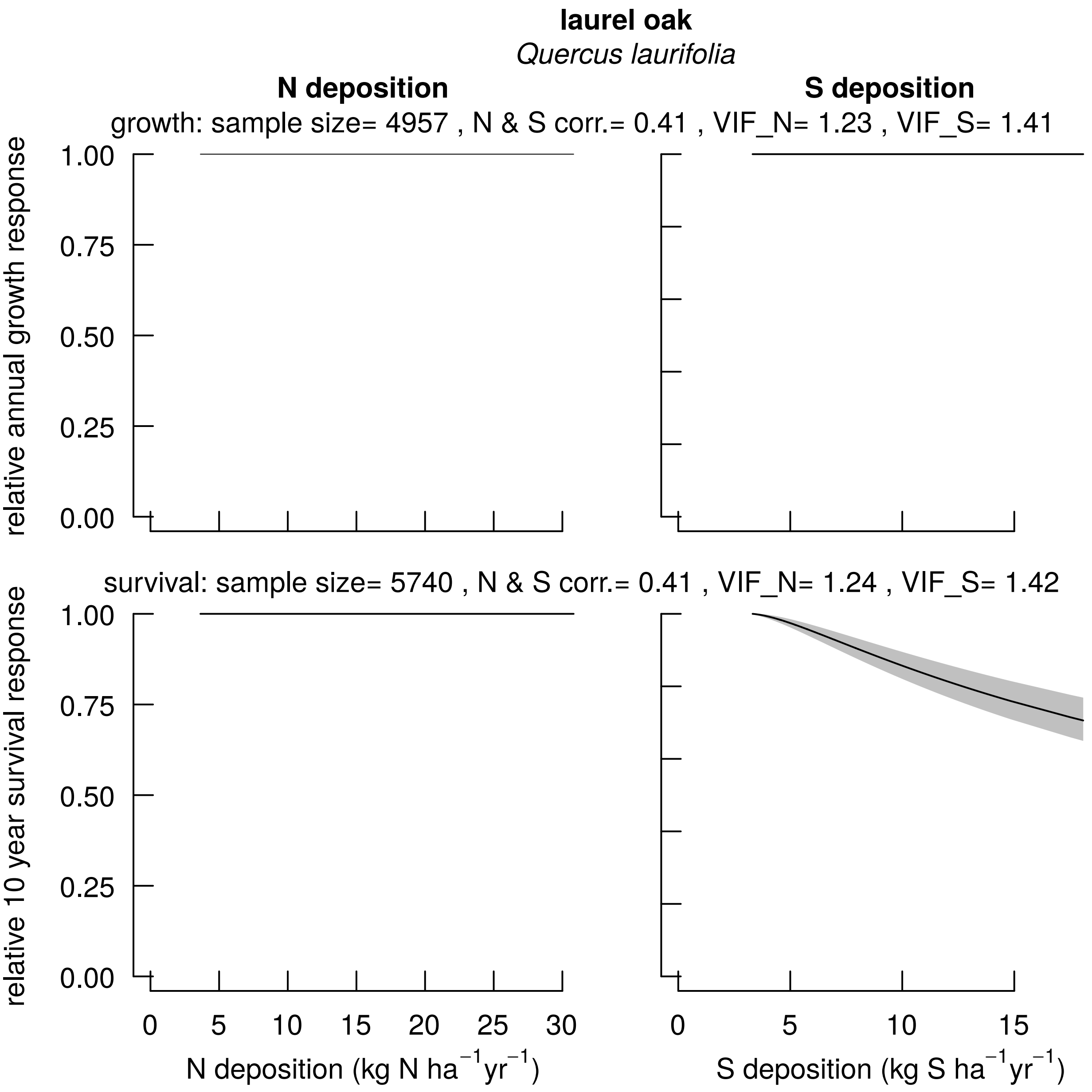
laurel oak
Quercus laurifolia
N deposition
S deposition
growth: sample size= 4957 , N & S corr.= 0.41 , VIF_N= 1.23 , VIF_S= 1.41
relative annual growth response
1.00
0.75
0.50
0.25
0.00
survival: sample size= 5740 , N & S corr.= 0.41 , VIF_N= 1.24 , VIF_S= 1.42
relative 10 year survival response
1.00
0.75
0.50
0.25
0.00
0 5 10 15 20 25 30
0 5 10 15
N deposition (kg N ha^{-1}yr^{-1})
S deposition (kg S ha^{-1}yr^{-1})

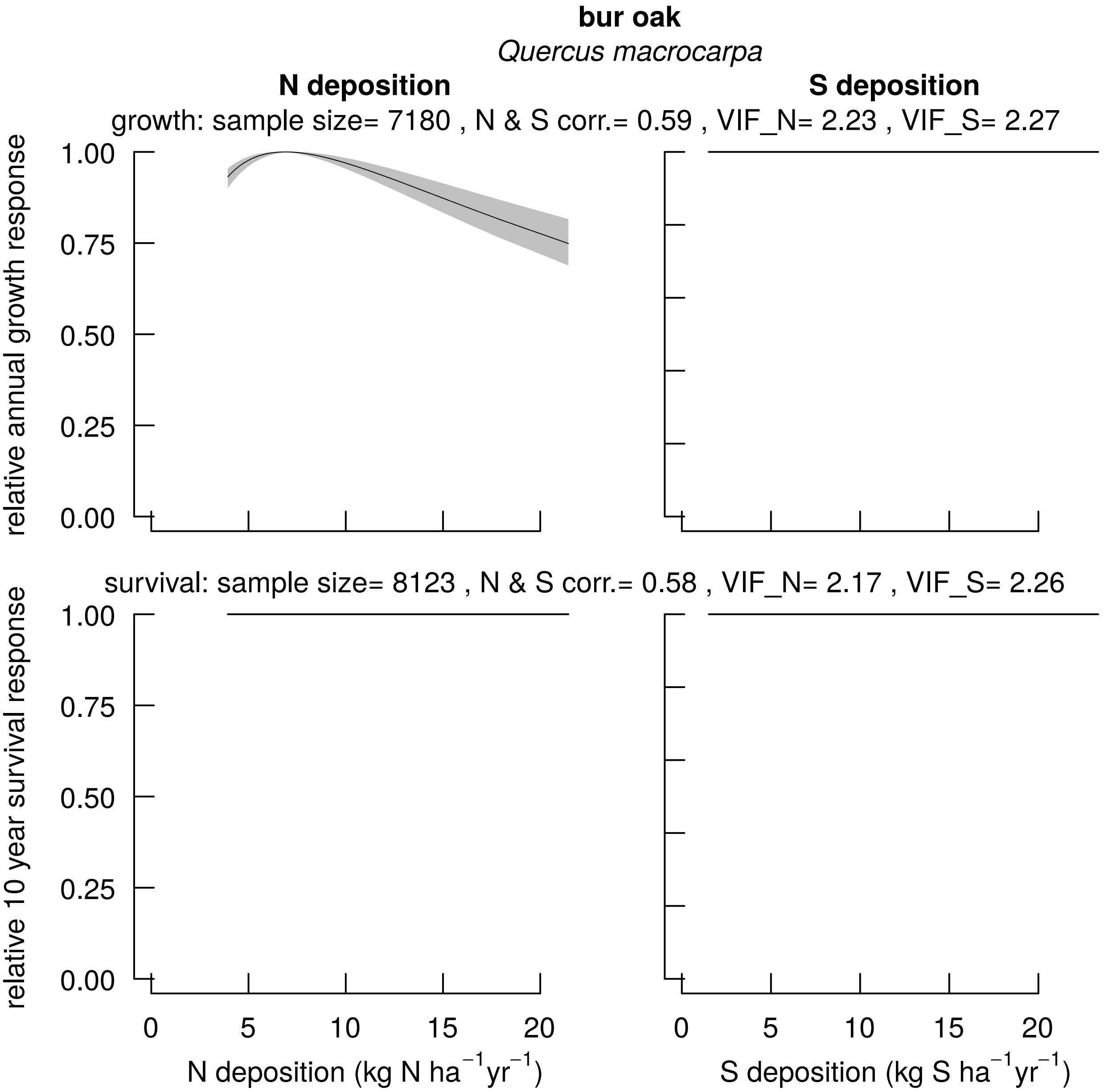

bur oak
Quercus macrocarpa
N deposition
S deposition
growth: sample size= 7180 , N & S corr.= 0.59 , VIF_N= 2.23 , VIF_S= 2.27
relative annual growth response
1.00
0.75
0.50
0.25
0.00
survival: sample size= 8123 , N & S corr.= 0.58 , VIF_N= 2.17 , VIF_S= 2.26
relative 10 year survival response
1.00
0.75
0.50
0.25
0.00
0
5
10
15
20
N deposition (kg N ha⁻¹yr⁻¹)
0
5
10
15
20
S deposition (kg S ha⁻¹yr⁻¹)

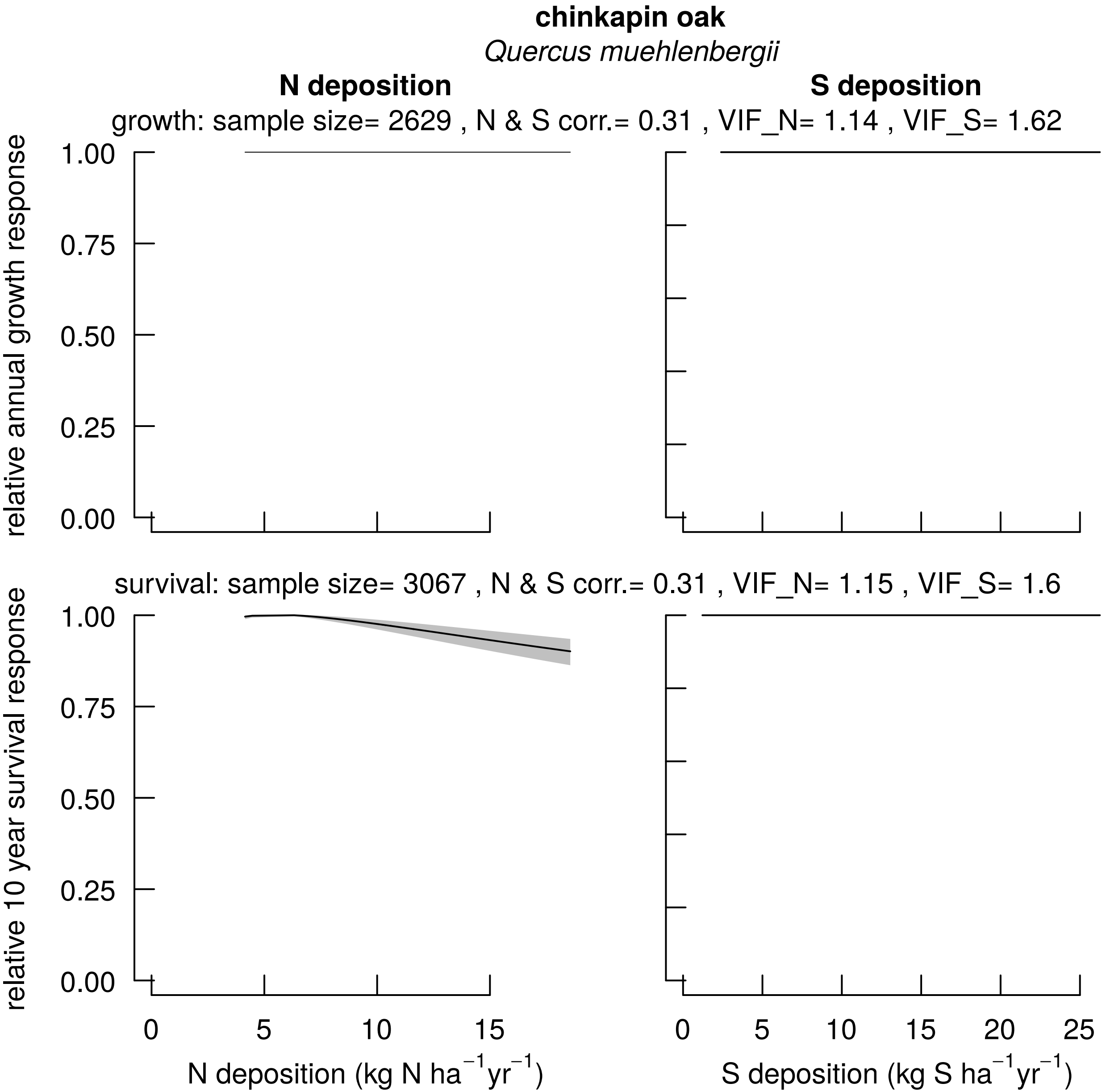

chinkapin oak
Quercus muehlenbergii
N deposition
S deposition
growth: sample size= 2629 , N & S corr.= 0.31 , VIF_N= 1.14 , VIF_S= 1.62
relative annual growth response
1.00
0.75
0.50
0.25
0.00
survival: sample size= 3067 , N & S corr.= 0.31 , VIF_N= 1.15 , VIF_S= 1.6
relative 10 year survival response
1.00
0.75
0.50
0.25
0.00
0
5
10
15
0
5
10
15
20
25
N deposition (kg N ha−1yr−1)
S deposition (kg S ha−1yr−1)

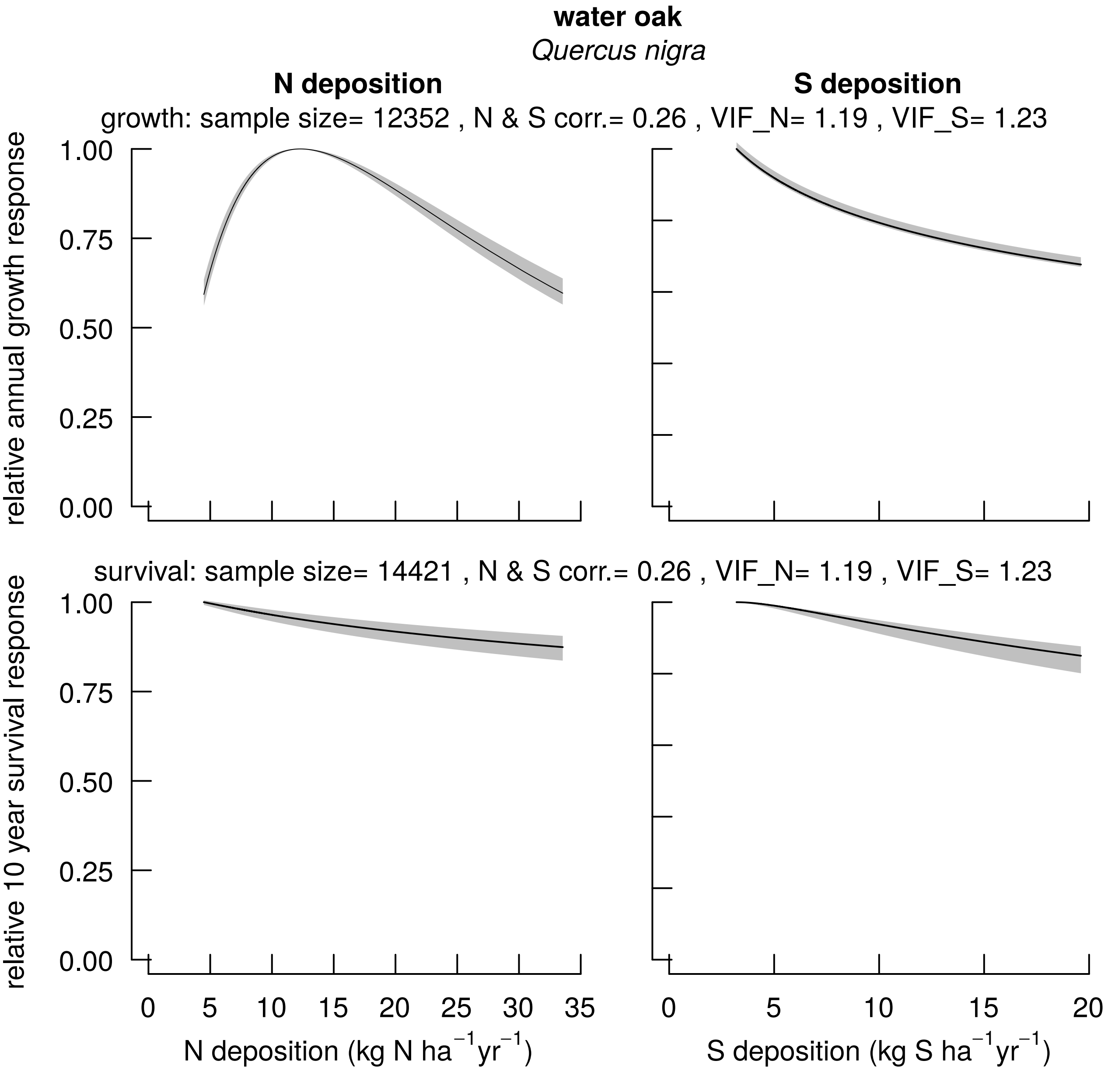
water oak
Quercus nigra
N deposition
S deposition
growth: sample size= 12352 , N & S corr.= 0.26 , VIF_N= 1.19 , VIF_S= 1.23
survival: sample size= 14421 , N & S corr.= 0.26 , VIF_N= 1.19 , VIF_S= 1.23
relative annual growth response
relative 10 year survival response
1.00
0.75
0.50
0.25
0.00
0
5
10
15
20
25
30
35
N deposition (kg N ha⁻¹yr⁻¹)
S deposition (kg S ha⁻¹yr⁻¹)

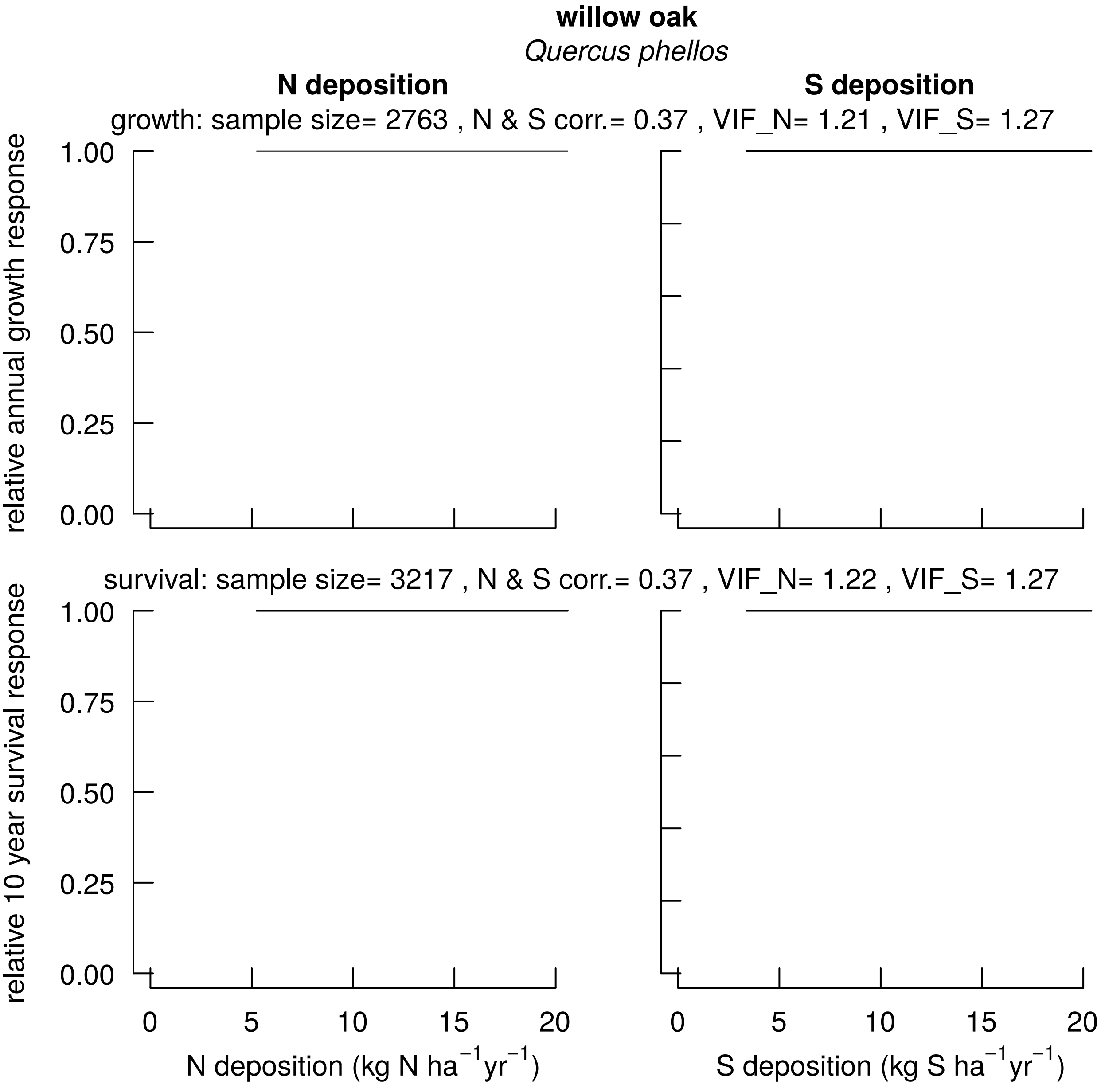

willow oak
Quercus phellos
N deposition
S deposition
growth: sample size= 2763 , N & S corr.= 0.37 , VIF_N= 1.21 , VIF_S= 1.27
relative annual growth response
1.00
0.75
0.50
0.25
0.00
survival: sample size= 3217 , N & S corr.= 0.37 , VIF_N= 1.22 , VIF_S= 1.27
relative 10 year survival response
0
5
10
15
20
N deposition (kg N ha^{-1}yr^{-1})
S deposition (kg S ha^{-1}yr^{-1})

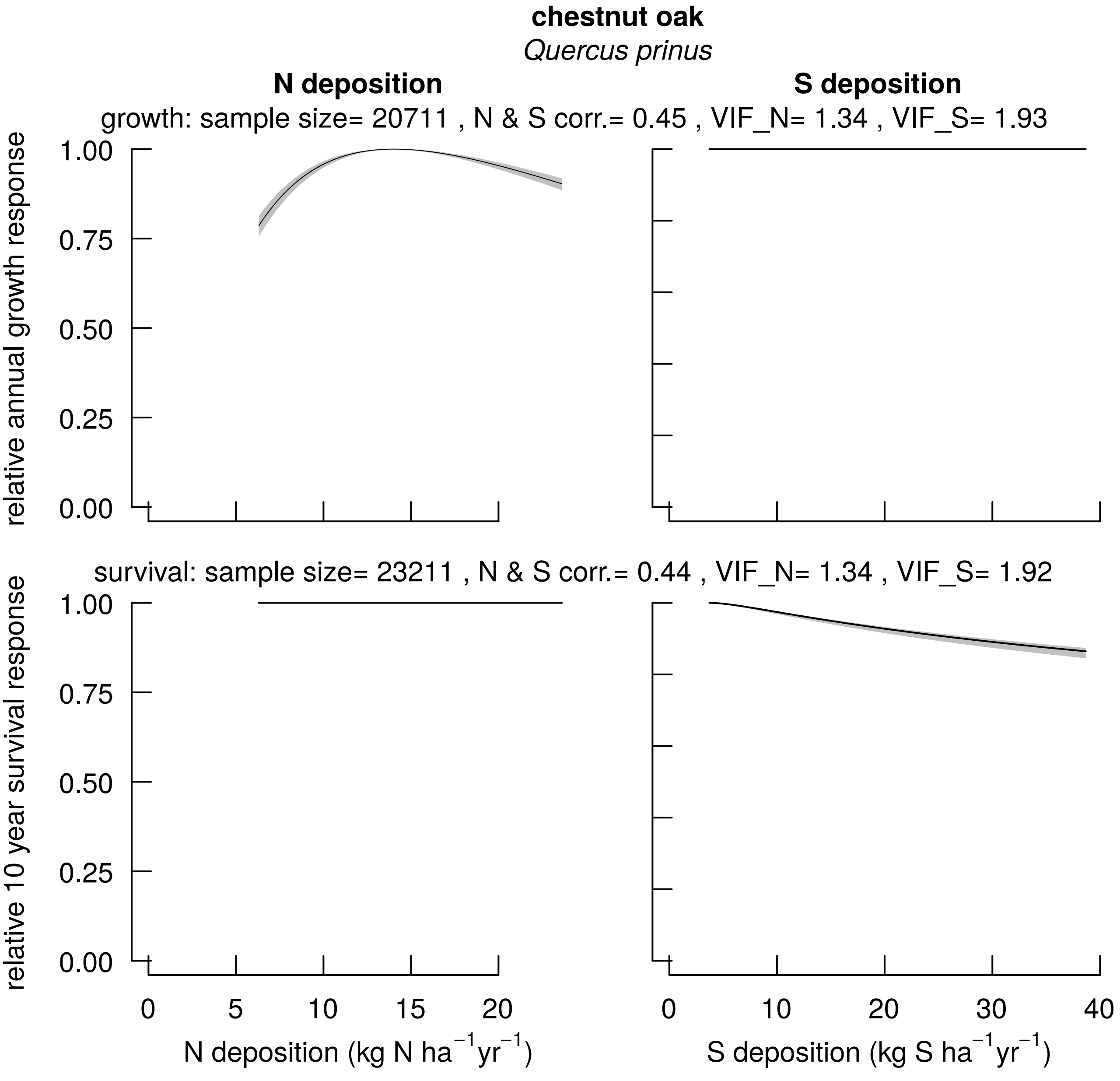

chestnut oak
Quercus prinus
N deposition
S deposition
growth: sample size= 20711 , N & S corr.= 0.45 , VIF_N= 1.34 , VIF_S= 1.93
relative annual growth response
1.00
0.75
0.50
0.25
0.00
survival: sample size= 23211 , N & S corr.= 0.44 , VIF_N= 1.34 , VIF_S= 1.92
relative 10 year survival response
0
5
10
15
20
30
40
N deposition (kg N ha^{-1}yr^{-1})
S deposition (kg S ha^{-1}yr^{-1})

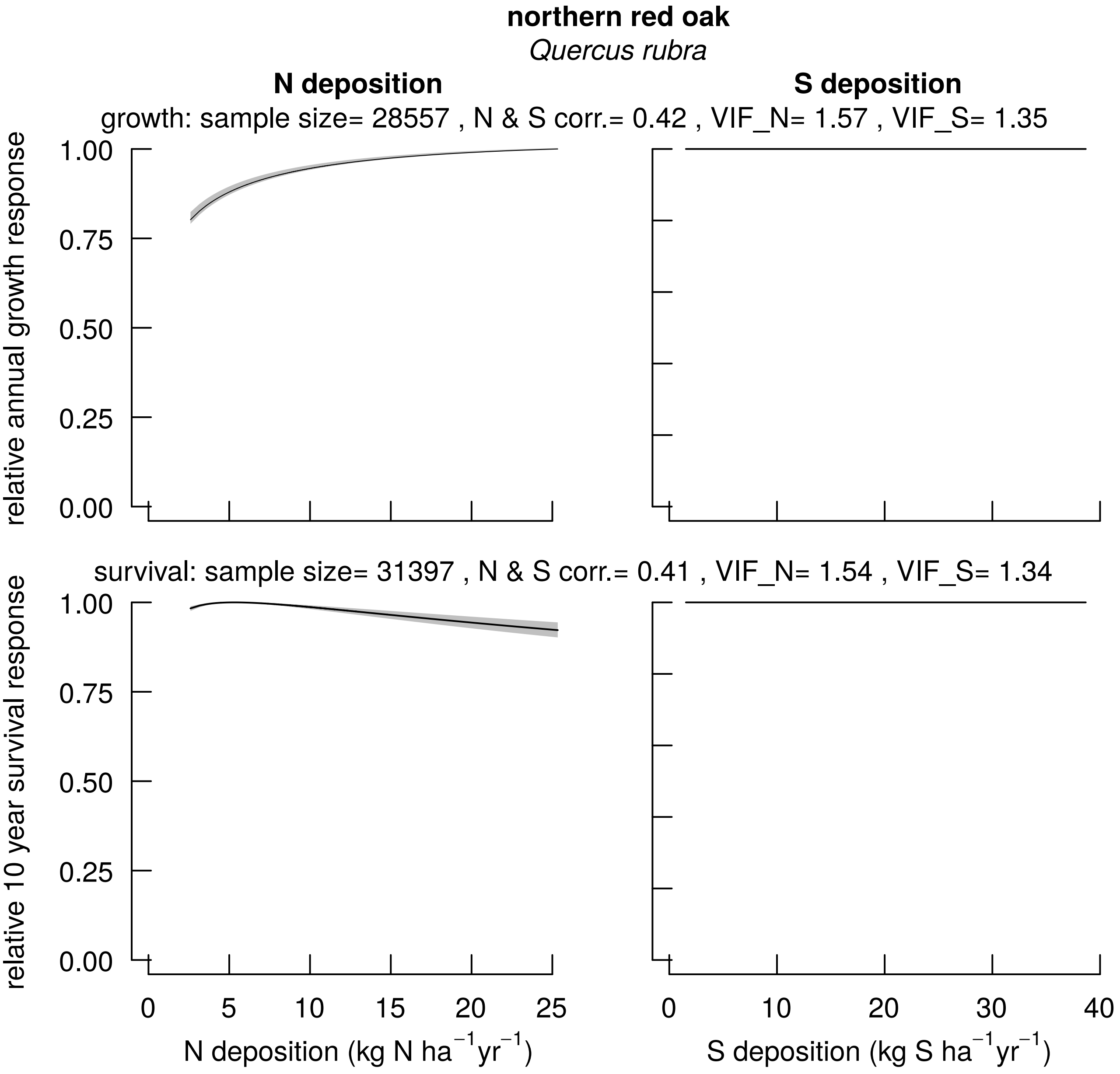
northern red oak
Quercus rubra
N deposition
S deposition
growth: sample size= 28557 , N & S corr.= 0.42 , VIF_N= 1.57 , VIF_S= 1.35
relative annual growth response
1.00
0.75
0.50
0.25
0.00
survival: sample size= 31397 , N & S corr.= 0.41 , VIF_N= 1.54 , VIF_S= 1.34
relative 10 year survival response
0 5 10 15 20 25
0 10 20 30 40
N deposition (kg N ha^{-1}yr^{-1})
S deposition (kg S ha^{-1}yr^{-1})

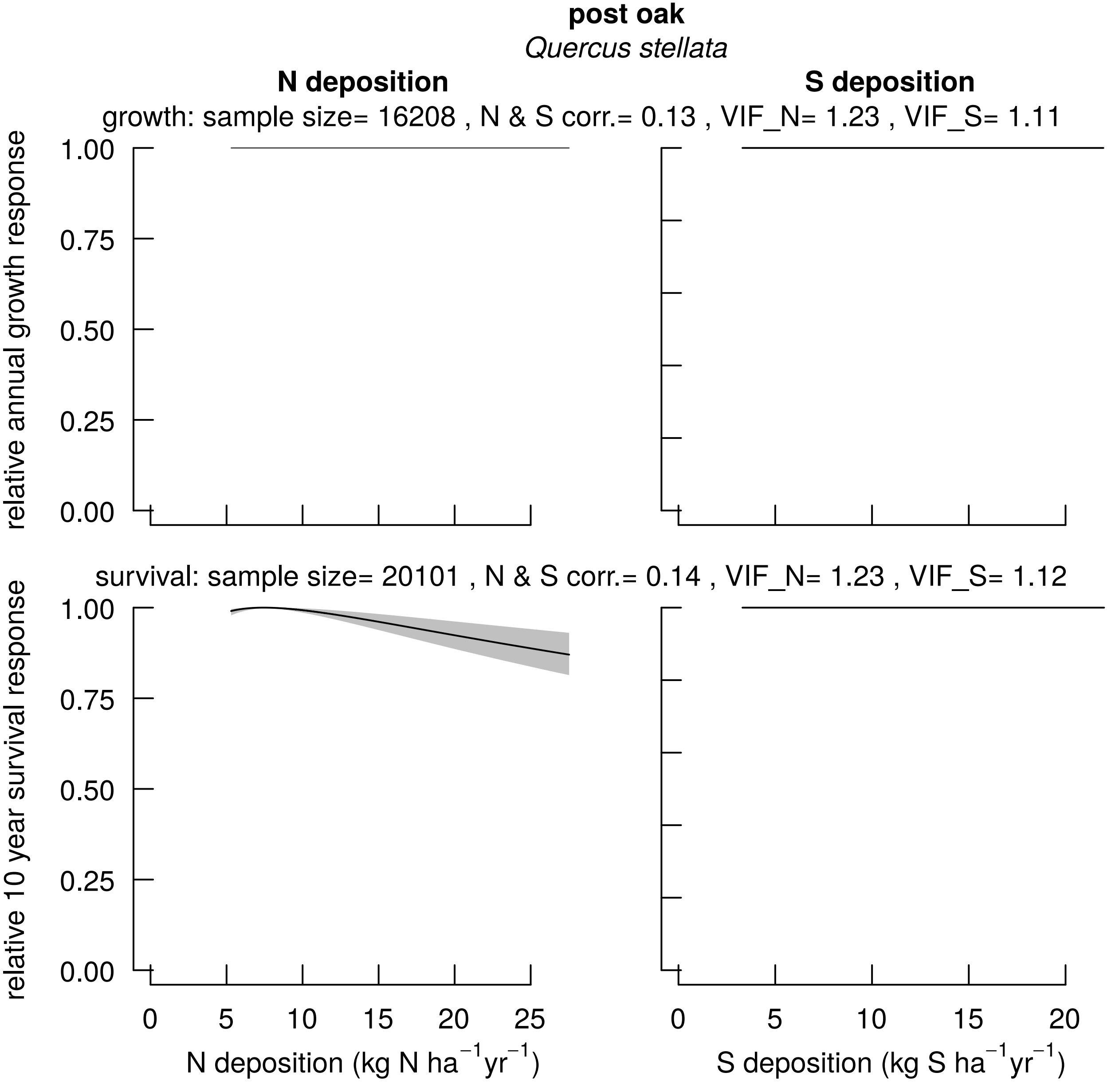

post oak
Quercus stellata
N deposition
S deposition
growth: sample size= 16208 , N & S corr.= 0.13 , VIF_N= 1.23 , VIF_S= 1.11
relative annual growth response
1.00
0.75
0.50
0.25
0.00
survival: sample size= 20101 , N & S corr.= 0.14 , VIF_N= 1.23 , VIF_S= 1.12
relative 10 year survival response
1.00
0.75
0.50
0.25
0.00
0
5
10
15
20
25
N deposition (kg N ha−1yr−1)
0
5
10
15
20
S deposition (kg S ha−1yr−1)

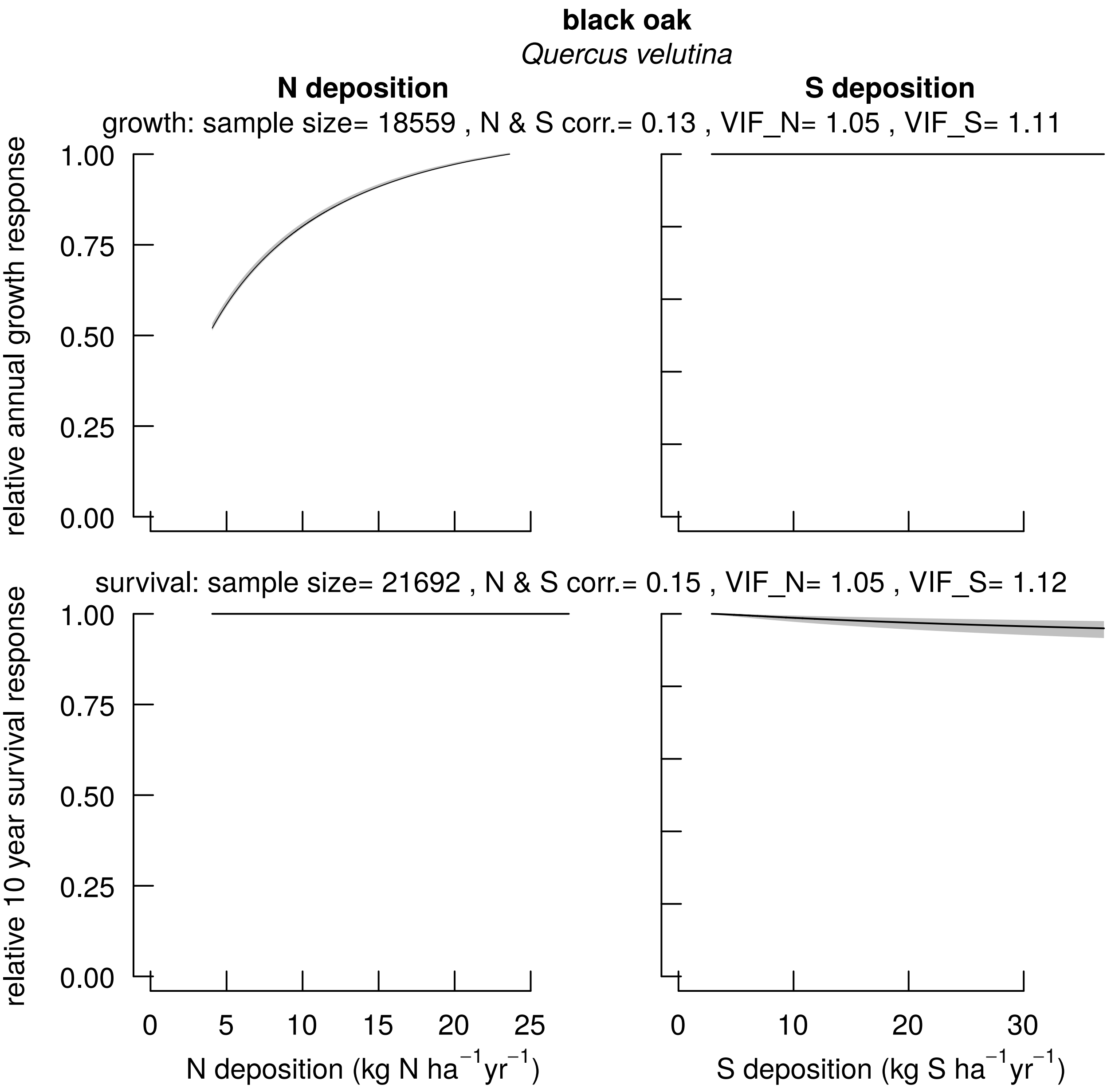

black oak
Quercus velutina
N deposition
S deposition
growth: sample size= 18559 , N & S corr.= 0.13 , VIF_N= 1.05 , VIF_S= 1.11
relative annual growth response
1.00
0.75
0.50
0.25
0.00
survival: sample size= 21692 , N & S corr.= 0.15 , VIF_N= 1.05 , VIF_S= 1.12
relative 10 year survival response
1.00
0.75
0.50
0.25
0.00
0
5
10
15
20
25
0
10
20
30
N deposition (kg N ha⁻¹yr⁻¹)
S deposition (kg S ha⁻¹yr⁻¹)

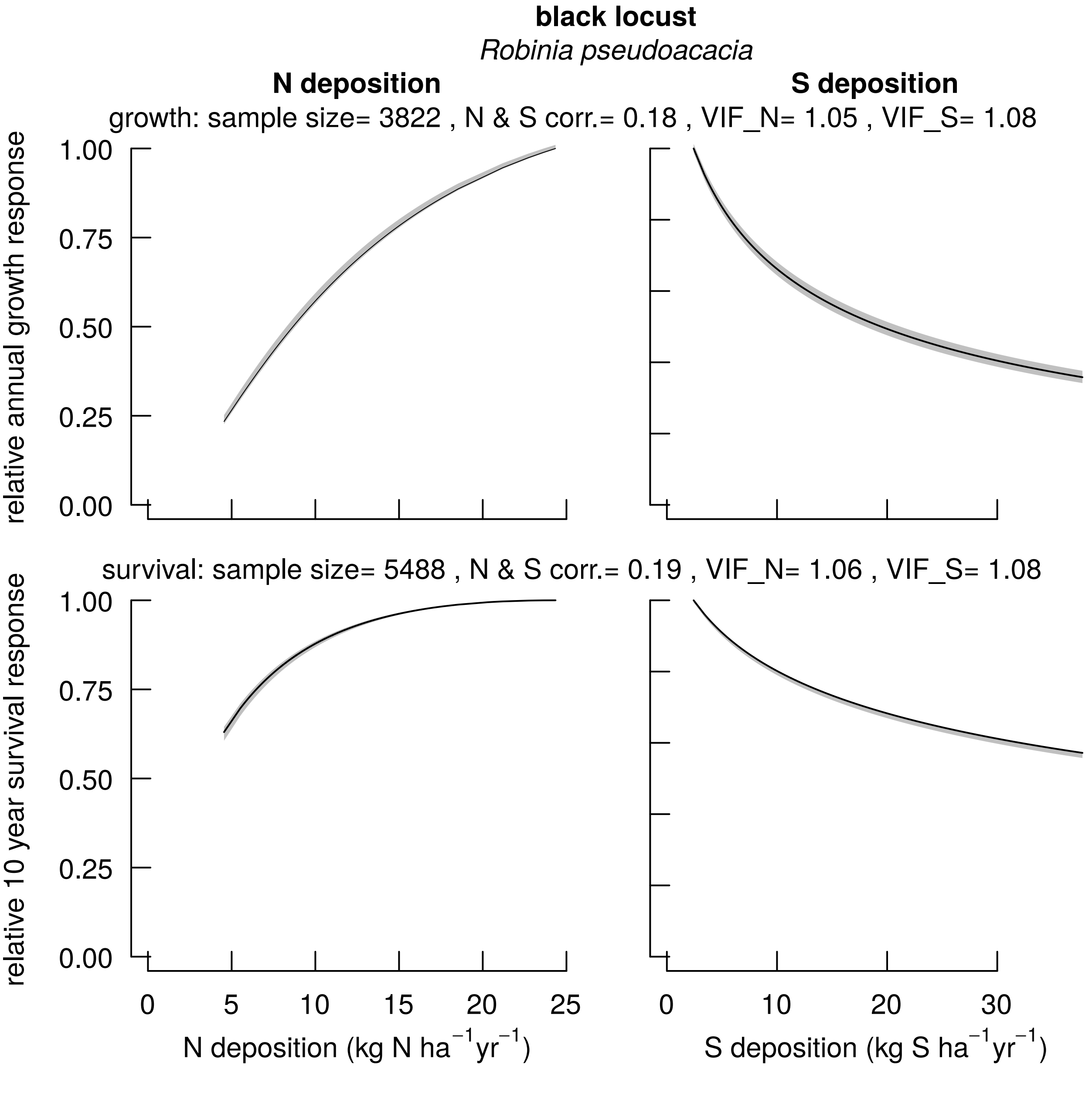

black locust
Robinia pseudoacacia
N deposition
S deposition
growth: sample size= 3822 , N & S corr.= 0.18 , VIF_N= 1.05 , VIF_S= 1.08
relative annual growth response
1.00
0.75
0.50
0.25
0.00
survival: sample size= 5488 , N & S corr.= 0.19 , VIF_N= 1.06 , VIF_S= 1.08
relative 10 year survival response
1.00
0.75
0.50
0.25
0.00
0
5
10
15
20
25
0
10
20
30
N deposition (kg N ha^{-1}yr^{-1})
S deposition (kg S ha^{-1}yr^{-1})

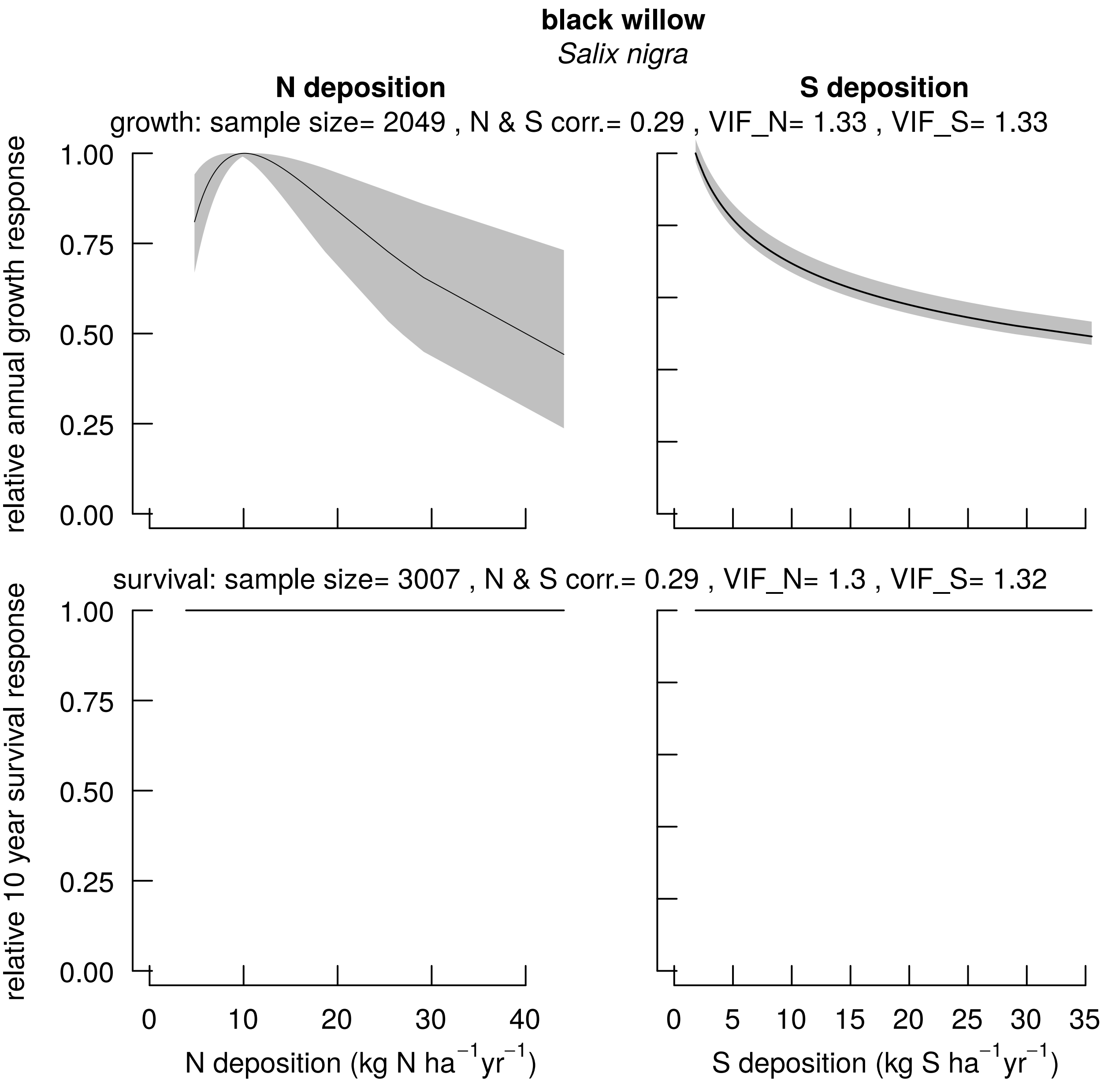

black willow
Salix nigra
N deposition
S deposition
growth: sample size= 2049 , N & S corr.= 0.29 , VIF_N= 1.33 , VIF_S= 1.33
relative annual growth response
1.00
0.75
0.50
0.25
0.00
survival: sample size= 3007 , N & S corr.= 0.29 , VIF_N= 1.3 , VIF_S= 1.32
relative 10 year survival response
0 10 20 30 40
0 5 10 15 20 25 30 35
N deposition (kg N $ha^{-1}yr^{-1}$)
S deposition (kg S $ha^{-1}yr^{-1}$)

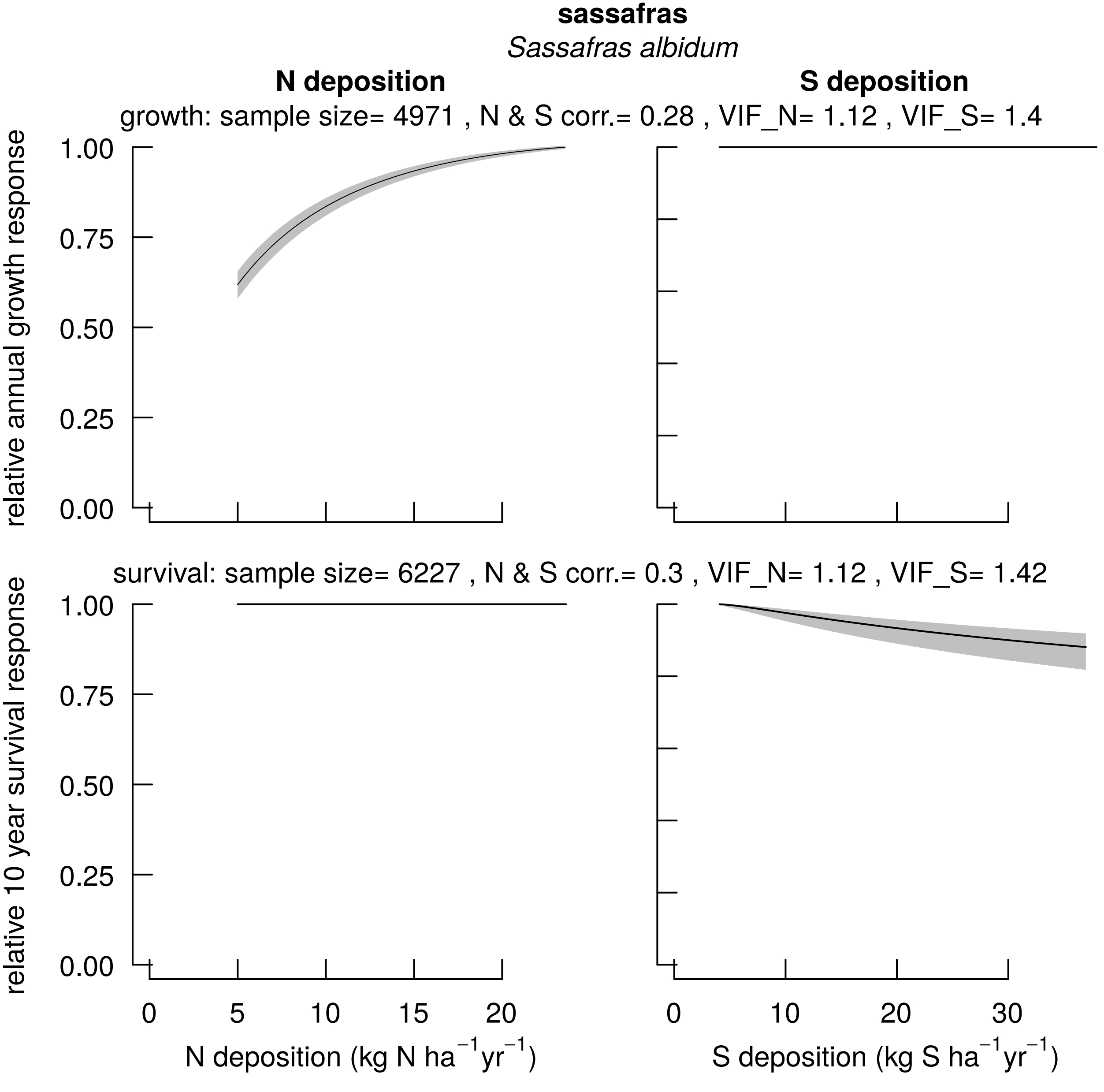

sassafras
Sassafras albidum
N deposition
S deposition
growth: sample size= 4971 , N & S corr.= 0.28 , VIF_N= 1.12 , VIF_S= 1.4
survival: sample size= 6227 , N & S corr.= 0.3 , VIF_N= 1.12 , VIF_S= 1.42
relative annual growth response
relative 10 year survival response
1.00
0.75
0.50
0.25
0.00
0
5
10
15
20
0
10
20
30
N deposition (kg N $ha^{-1}yr^{-1}$)
S deposition (kg S $ha^{-1}yr^{-1}$)

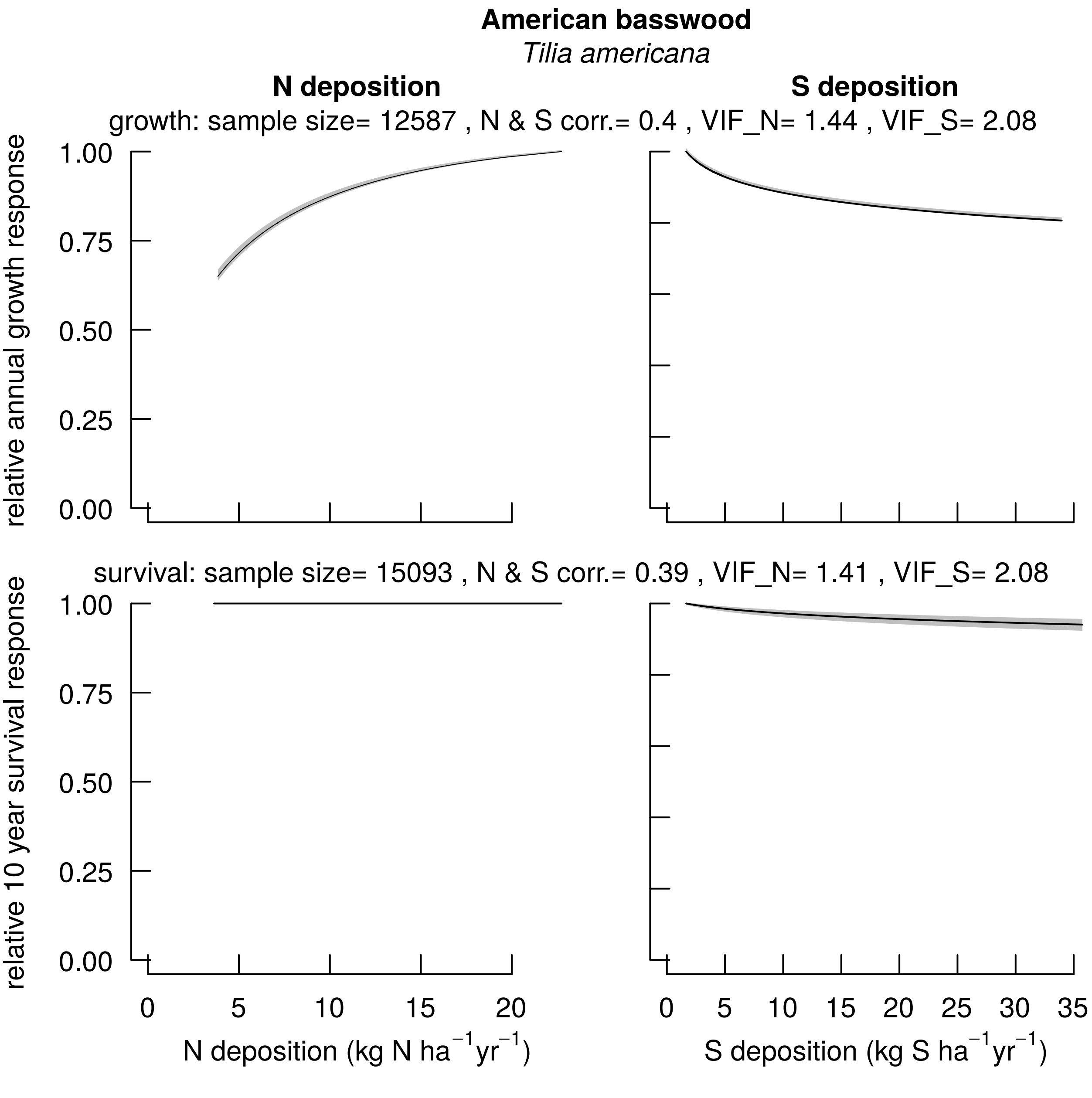

American basswood
Tilia americana
N deposition
S deposition
growth: sample size= 12587 , N & S corr.= 0.4 , VIF_N= 1.44 , VIF_S= 2.08
relative annual growth response
1.00
0.75
0.50
0.25
0.00
survival: sample size= 15093 , N & S corr.= 0.39 , VIF_N= 1.41 , VIF_S= 2.08
relative 10 year survival response
1.00
0.75
0.50
0.25
0.00
0
5
10
15
20
0
5
10
15
20
25
30
35
N deposition (kg N ha^{-1}yr^{-1})
S deposition (kg S ha^{-1}yr^{-1})

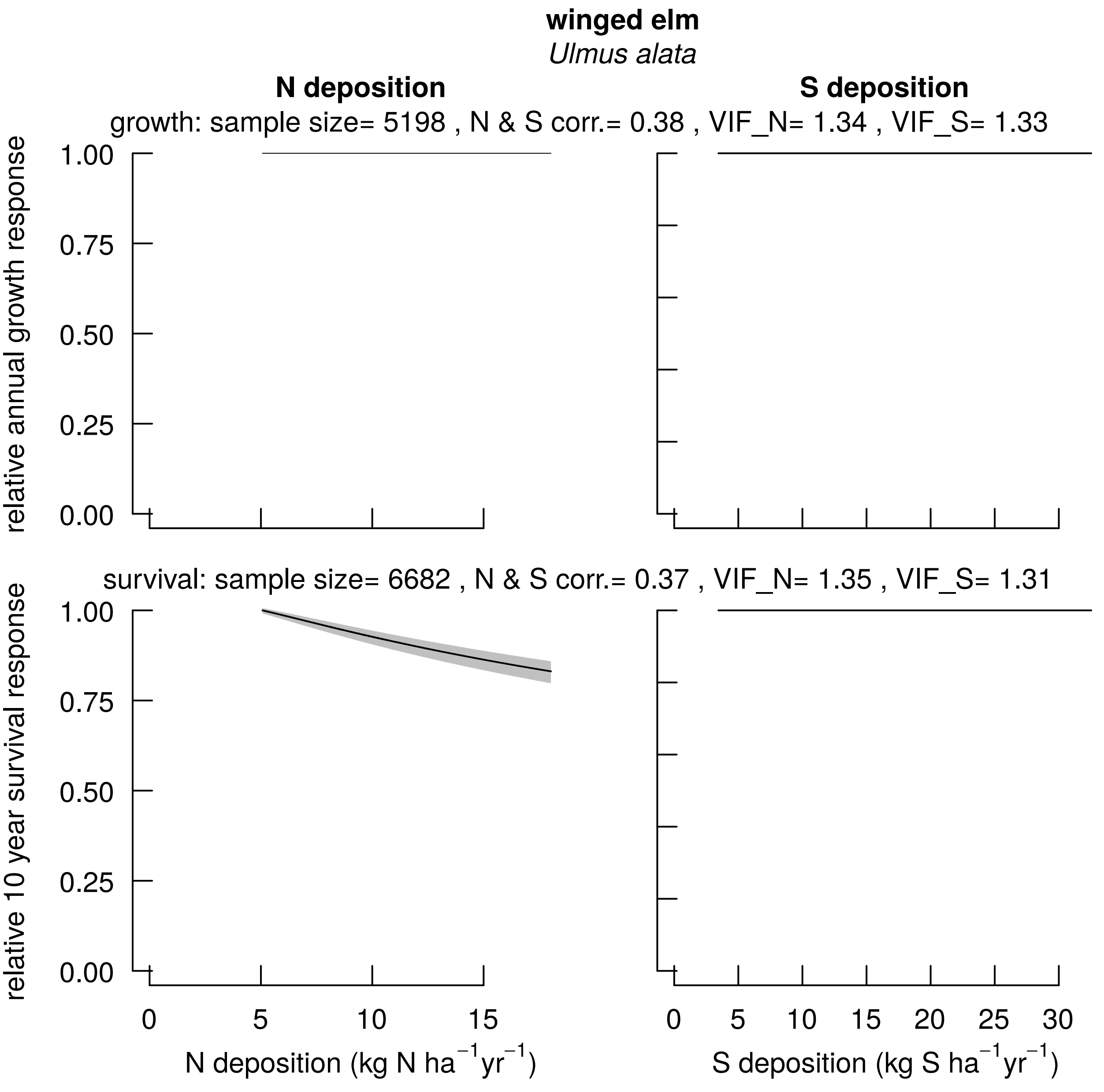

winged elm
Ulmus alata
N deposition
S deposition
growth: sample size= 5198 , N & S corr.= 0.38 , VIF_N= 1.34 , VIF_S= 1.33
relative annual growth response
1.00
0.75
0.50
0.25
0.00
survival: sample size= 6682 , N & S corr.= 0.37 , VIF_N= 1.35 , VIF_S= 1.31
relative 10 year survival response
1.00
0.75
0.50
0.25
0.00
0
5
10
15
N deposition (kg N ha−1yr−1)
0
5
10
15
20
25
30
S deposition (kg S ha−1yr−1)

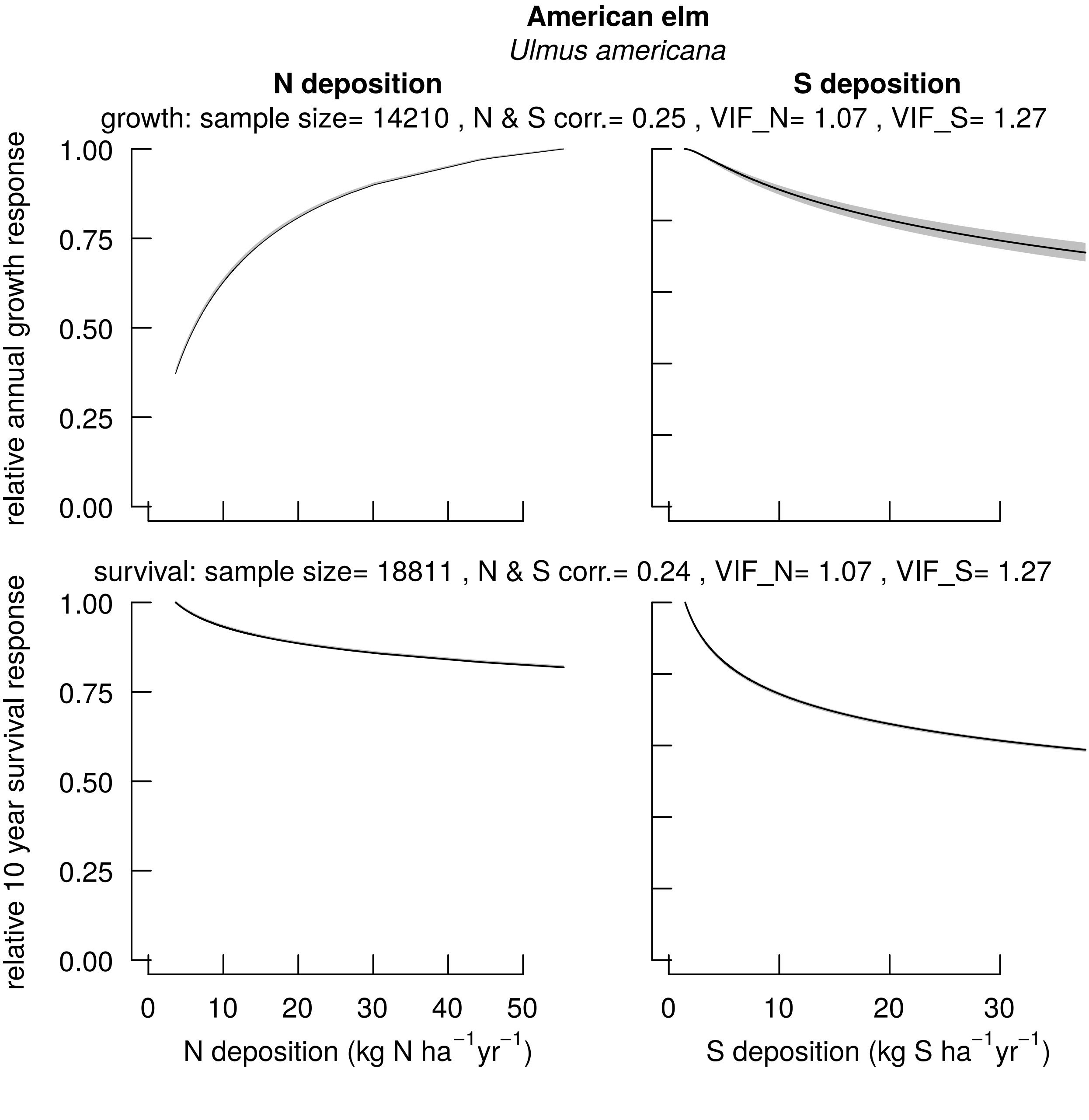
American elm
Ulmus americana
N deposition
S deposition
growth: sample size= 14210 , N & S corr.= 0.25 , VIF_N= 1.07 , VIF_S= 1.27
survival: sample size= 18811 , N & S corr.= 0.24 , VIF_N= 1.07 , VIF_S= 1.27
relative annual growth response
relative 10 year survival response
1.00
0.75
0.50
0.25
0.00
0
10
20
30
40
50
N deposition (kg N ha^{-1}yr^{-1})
S deposition (kg S ha^{-1}yr^{-1})

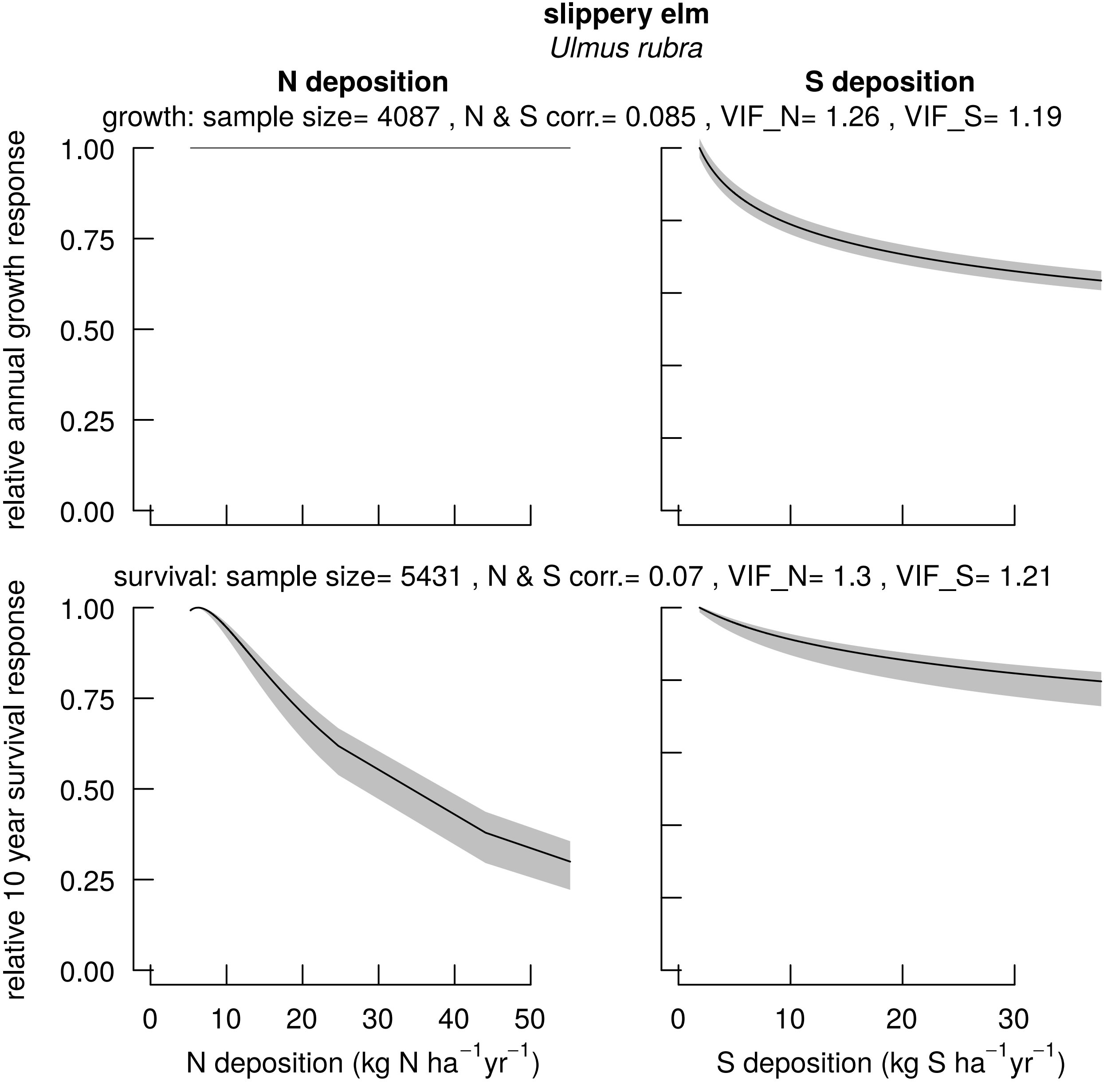

slippery elm
Ulmus rubra
N deposition
S deposition
growth: sample size= 4087 , N & S corr.= 0.085 , VIF_N= 1.26 , VIF_S= 1.19
relative annual growth response
1.00
0.75
0.50
0.25
0.00
survival: sample size= 5431 , N & S corr.= 0.07 , VIF_N= 1.3 , VIF_S= 1.21
relative 10 year survival response
1.00
0.75
0.50
0.25
0.00
0
10
20
30
40
50
0
10
20
30
N deposition (kg N ha^{-1}yr^{-1})
S deposition (kg S ha^{-1}yr^{-1})
